# Synthesis and hydrogenation of polycyclic aromatic hydrocarbon-substituted diborenes *via* uncatalysed hydrogenative B–C bond cleavage†

**DOI:** 10.1039/d2sc02515a

**Published:** 2022-06-14

**Authors:** Alexander Okorn, Arumugam Jayaraman, Lukas Englert, Merle Arrowsmith, Theresa Swoboda, Jeanette Weigelt, Carina Brunecker, Merlin Hess, Anna Lamprecht, Carsten Lenczyk, Maximilian Rang, Holger Braunschweig

**Affiliations:** Institute for Inorganic Chemistry, Julius-Maximilians-Universität Würzburg Am Hubland 97074 Würzburg Germany h.braunschweig@uni-wuerzburg.de; Institute for Sustainable Chemistry & Catalysis with Boron, Julius-Maximilians-Universität Würzburg Am Hubland 97074 Würzburg Germany

## Abstract

The classical route to the PMe_3_-stabilised polycyclic aromatic hydrocarbon (PAH)-substituted diborenes B_2_Ar_2_(PMe_3_)_2_ (Ar = 9-phenanthryl 7-Phen; Ar = 1-pyrenyl 7-Pyr) *via* the corresponding 1,2-diaryl-1,2-dimethoxydiborane(4) precursors, B_2_Ar_2_(OMe)_2_, is marred by the systematic decomposition of the latter to BAr(OMe)_2_ during reaction workup. Calculations suggest this results from the absence of a second *ortho*-substituent on the boron-bound aryl rings, which enables their free rotation and exposes the B–B bond to nucleophilic attack. 7-Phen and 7-Pyr are obtained by the reduction of the corresponding 1,2-diaryl-1,2-dichlorodiborane precursors, B_2_Ar_2_Cl_2_(PMe_3_)_2_, obtained from the SMe_2_ adducts, which are synthesised by direct NMe_2_–Cl exchange at B_2_Ar_2_(NMe_2_)_2_ with (Me_2_S)BCl_3_. The low-lying π* molecular orbitals (MOs) located on the PAH substituents of 7-Phen and 7-Pyr intercalate between the B–B-based π and π* MOs, leading to a relatively small HOMO–LUMO gap of 3.20 and 2.72 eV, respectively. Under vacuum or at high temperature 7-Phen and 7-Pyr undergo intramolecular hydroarylation of the B=B bond to yield 1,2-dihydronaphtho[1,8-*cd*][1,2]diborole derivatives. Hydrogenation of 7-Phen, 7-Pyr and their 9-anthryl and mesityl analogues III and II, respectively, results in all cases in splitting of the B–B bond and isolation of the monoboranes (Me_3_P)BArH_2_. NMR-spectroscopic monitoring of the reactions, solid-state structures of isolated reaction intermediates and computational mechanistic analyses show that the hydrogenation of the three PAH-substituted diborenes proceeds *via* a different pathway to that of the dimesityldiborene. Rather than occurring exclusively at the B–B bond, hydrogenation of 7-Ar and III proceeds *via* a hydroarylated intermediate, which undergoes one B–B bond-centered H_2_ addition, followed by hydrogenation of the endocyclic B–C bond resulting from hydroarylation, making the latter effectively reversible.

## Abstract 2

In contrast to classical B–B bond-centred diborene hydrogenation, polycyclic aromatic hydrocarbon-substituted diborenes first undergo thermal intramolecular hydroarylation, followed by hydrogenation of the remaining B–B and endocyclic B–C bonds.

## Graphical abstract

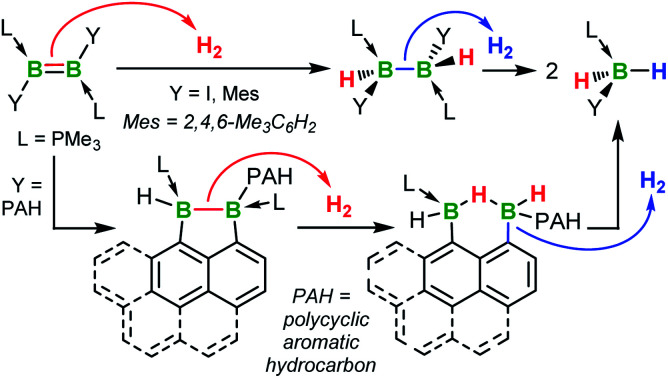

## Introduction

Since the isolation of the first neutral diborenes stabilised by two Lewis bases by Robinson and coworkers,^1,2^ the synthesis of these very electron-rich boron-based alkene analogues and the exploration of their versatile reactivity has advanced greatly.^3,4^ Symmetrical diborenes of the form L_2_B_2_Y_2_ (where L is a neutral donor (*e.g.* N-heterocyclic carbene (NHC), cyclic alkyl(amino)carbene (CAAC), phosphine) and Y an anionic substituent (*e.g.* aryl, heteroaryl, alkyl, vinyl, halide, cyanide, hydride)) are most often synthesised either by the reductive coupling of two LBYX_2_ precursors (X = halide),^3–10^ or the reduction of 1,2-dihalodiboranes of the form L_2_B_2_Y_2_X_2_.^3,11–14^ Since the apolar B–B bond of symmetrical diborenes often makes them relatively unreactive, recent efforts have also focused on synthesising polar, unsymmetrical diborenes by the reduction of unsymmetrically substituted 1,2-dihalodiboranes of the form LL′B_2_YY′X_2_.^15–20^

**Fig. 1:**
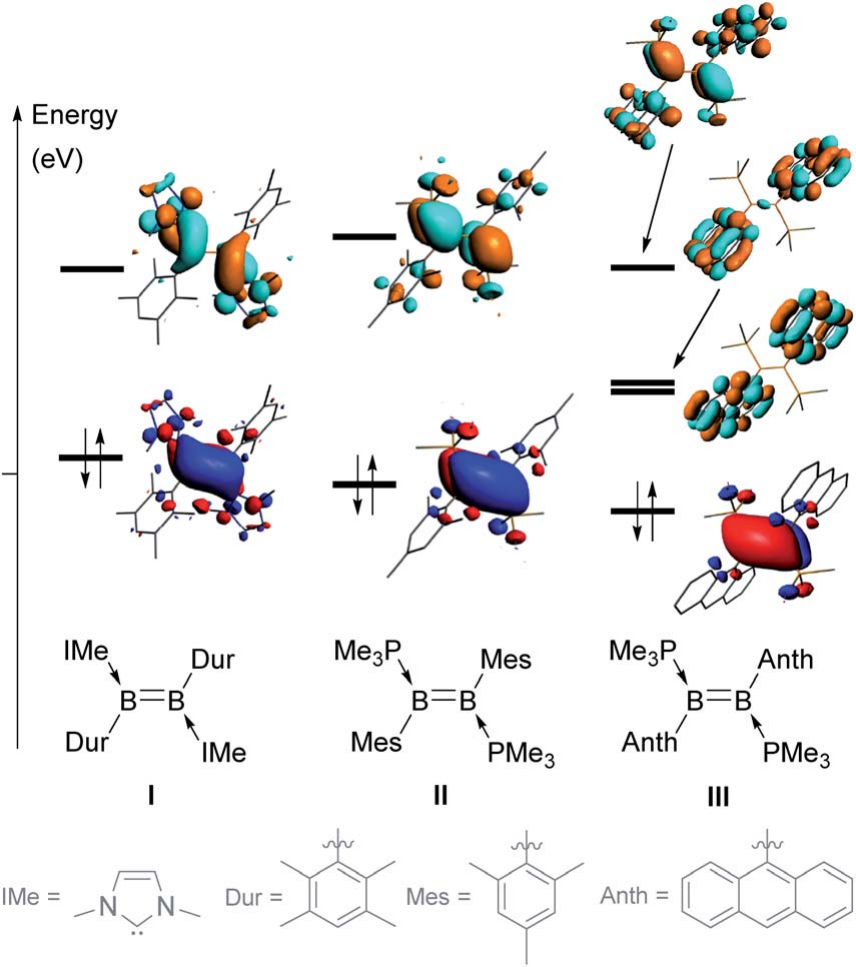
Comparison of the frontier molecular orbitals (MOs) of diborenes I, II and III.

The stereoelectronic nature of the substituents of the B=B double bond greatly influences its reactivity by tuning the energies (and sometimes localisation) of the highest occupied and lowest unoccupied molecular orbitals (HOMO and LUMO). For example, changing the NHC and duryl ligands in diborene I (Fig. 1)^21^ to PMe_3_ and mesityl ligands, respectively, in II,^22^ causes a decrease in the energy of the HOMO and an increase in the energy of the LUMO, thereby widening the HOMO–LUMO gap by *ca.* 0.7 eV.^23^ In both I and II, the HOMO is localised on the B=B π bond, while the LUMO corresponds to the B–B π* orbital. When the mesityl substituents of II are changed to the polycyclic 9-anthryl (Anth) substituents of III,^12^ the energy of the HOMO is slightly lowered but, most importantly, two of the π* orbitals of the anthryl fragments (in-phase and anti-phase) intercalate between the B–B π and π* orbitals (usually the frontier orbitals), thereby lowering the HOMO–LUMO gap to only 1.07 eV. This orbital intercalation adds to the usual B–B bond-centred reactivity of diborenes the possibility of aryl-centred reactivity, as borne out by the intramolecular C–H borylation of the anthryl moiety in reactions with selenium and copper triflate.^12,24^

**Scheme 1:**
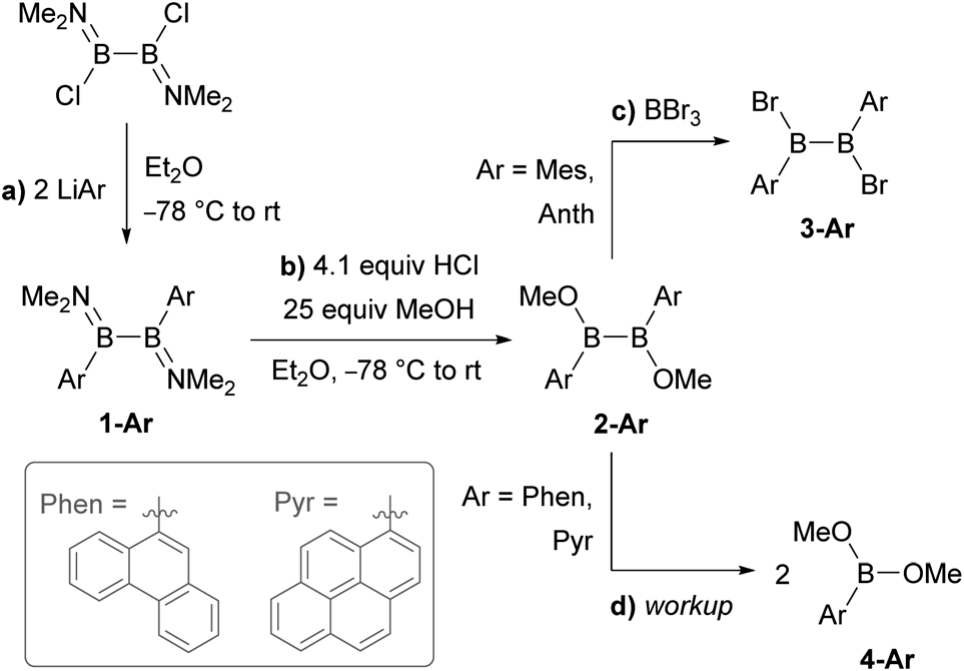
Attempted synthesis of B_2_Ar_2_Br_2_ (3-Ar, Ar = Phen, Pyr) *via* B_2_Ar_2_(OMe)_2_ (2-Ar).

Thus far, all known diaryldiborenes bear very sterically demanding aryl substituents, substituted in both *ortho* positions, which locks these substituents in a position orthogonal to the plane of the diborene core. In order to increase the reactivity of diaryldiborenes even further we aimed to synthesise new polycyclic aromatic hydrocarbon (PAH)-substituted diborenes with a similar frontier orbital arrangement as III, but in which the boron-bound aryl ring bears only one *ortho* substituent, such as the 9-phenanthryl and 1-pyrenyl groups displayed in Scheme 1. In this study, we show that the unsymmetrical nature and diminished steric profiles of the 9-phenanthryl and 1-pyrenyl substituents first of all necessitate a different synthetic route to the desired PMe_3_-stabilised diphenanthryl- and dipyrenyldiborenes, and secondly make the latter thermally unstable towards intramolecular C–H borylation. Furthermore, we examine the hydrogenation mechanism of PAH-substituted diborenes relative to that of the dimesityldiborene III, both experimentally and computationally.

## Results and discussion

### Synthesis of PAH-substituted diborane precursors

Literature-known phosphine-stabilised 1,2-diaryldiborenes such as II and III are synthesized by twofold reduction of the 1,2-diaryl-1,2-dibromodiborane precursors, B_2_Ar_2_Br_2_ (3-Ar), in the presence of PMe_3_.^12,22^ The precursors 3-Ar are obtained *via* a three-step procedure by (a) arylation of B_2_X_2_(NMe_2_)_2_ (X = Cl, Br) with LiAr to yield 1-Ar, followed by (b) acidic methanol quenching to yield 2-Ar, and finally (c) bromination to yield 3-Ar (Scheme 1).^25–27^ Attempts to synthesise the corresponding diphenanthryl- and dipyrenyldiboranes(4) following the same procedure failed as step (b) was systematically followed by B–B bond cleavage of 2-Ar during workup. Although 2-Ar was detected in the ^11^B NMR spectrum of the reaction mixture at *ca.* 59 ppm (2-Mes: *δ*_11B_ = 61 ppm),^26^ workup systematically led to the isolation of the dimethoxyboranes 4-Ar (*δ*_11B_ = *ca.* 31 ppm, *cf.*4-Ph: *δ*_11B_ = 28.1 ppm)^28^ as the sole isolable products (Scheme 1d). Multiple attempts to isolate 2-Ar using different stoichiometries (2.1 equiv. MeOH and HCl), HCl solutions (in Et_2_O or toluene), solvents (Et_2_O, toluene, benzene, hexane), workup temperatures (0 °C, rt) and procedures (extraction, precipitation, neutralisation of ammonium salt by-product) all led to the isolation of the monoboranes 4-Ar as the major product in up to 77% yield (see ESI† for details).

**Fig. 2:**
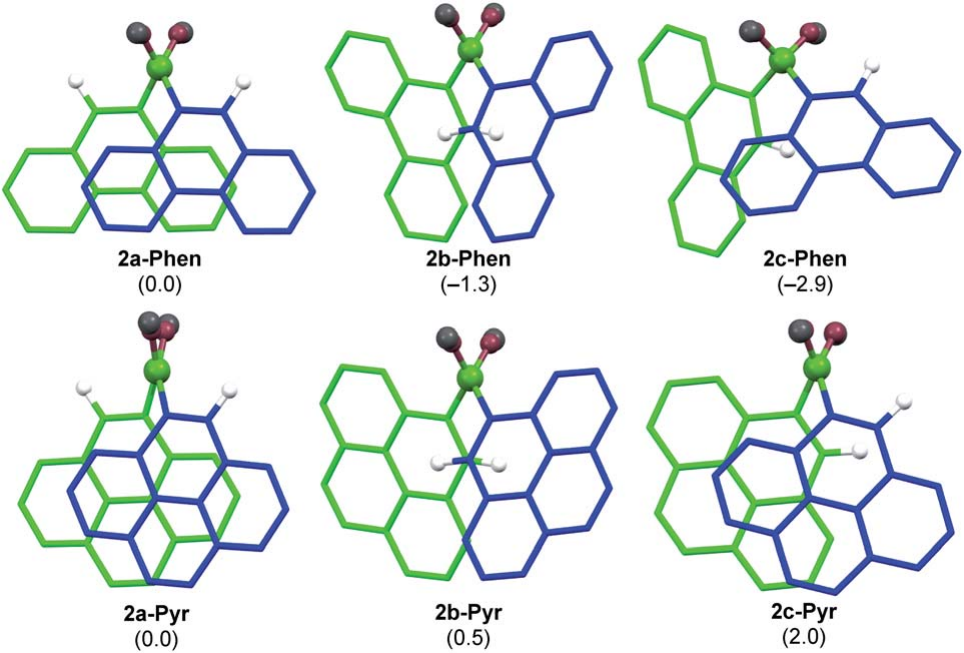
Calculated rotational isomers of 2-Phen and 2-Pyr optimised at the M06/6-31g(d,p) level of theory, viewed along the B–B axis. Hydrogen atoms omitted for clarity, except for the *ortho* protons. Blue framework: front aryl group; green framework: back aryl group. Atom colours: white, H; fir green, B; dark red, O; dark grey, C. Relative free energies in parentheses in kcal mol^−1^.

Since **2-Mes** and **2-Anth**, in which the boron-bound aryl rings are substituted in both *ortho* positions, are stable, the origin of the instability of 2-Phen and 2-Pyr must lie in the absence of a second *ortho* substituent. This results in a lower barrier to rotation of the aryl substituents. Calculations at the M06/6-31g(d,p) level of theory show that 2-Phen and 2-Pyr each have three stable rotational isomers, depending on the relative orientation of the two unsymmetrical aryl substituents (Fig. 2). For the pyrenyl derivative the most stable isomer is 2a-Pyr, in which the *ortho* protons point in opposite directions, maximizing π overlap between the parallel polycyclic aromatic groups by minimising the (C–B–B–C) torsion angle (2a-Pyr 22.2°). For comparison, the (C–B–B–C) torsion angles in the solid-state structures of 2-Mes and 2-Anth are *ca.* 104° and 125°, respectively.^12,26^ The isomer in which both *ortho* protons point towards each other, 2b-Pyr, is of similar energy to 2a-Pyr. The third isomer, in which both *ortho* protons point in the same direction, 2c-Pyr, lies only 2.0 kcal mol^−1^ higher in energy than 2a-Pyr. For the phenanthryl derivative the trend is reversed, 2c-Phen being 2.9 kcal mol^−1^ more stable than 2a-Phen, in which the π overlap is far less pronounced than in 2a-Pyr, due to a much larger (C–B–B–C) torsion angle of 65°. In both cases, however, the asymmetry of the energetically accessible 2c-Ar conformation, combined with the much lower steric protection afforded by the 9-phenanthryl and 1-pyrenyl ligands when compared to the mesityl and 9-anthryl ligands, may be the cause of the increased instability of 2-Phen and 2-Pyr, presumably promoting nucleophilic attack of unreacted MeOH at the B–B bond. The exact mechanism of the conversion from the diboranes 2-Ar to the monoboranes 4-Ar, however, remains unclear.

**Scheme 2:**
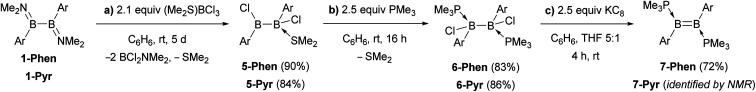
Synthetic route to the diborenes B_2_Ar_2_(PMe_3_)_2_ (7-Ar) *via* the diaryl(dichloro)diborane precursors 5-Ar and 6-Ar.

An alternative route was envisaged by direct NMe_2_–Br exchange using (Me_2_S)BBr_3_ as the bromide source.^29^ While 1-Ar (Ar = Phen, Pyr) underwent NMe_2_–Br exchange with (Me_2_S)BBr_3_ as shown by the formation of BBr_2_(NMe_2_) (*δ*_11B_ = 26 ppm),^30^ the reaction did not yield the desired 1,2-diaryl-1,2-dibromodiborane, but instead a complex mixture of products, including some resulting from B–B bond splitting. In contrast, the reaction of 1-Ar (Ar = Phen, Pyr) with 2.1 equiv. (Me_2_S)BCl_3_ in benzene proceeded cleanly over a period of 5 days at room temperature to afford the SMe_2_-stabilised precursors 5-Ar in 90% (Ar = Phen) and 84% (Ar = Pyr) isolated yields (Scheme 2a). The addition of 2 equiv. PMe_3_ to 5-Ar resulted in quantitative conversion to the doubly PMe_3_-stabilised 1,2-diaryl-1,2-dichlorodiboranes, 6-Ar (*δ*_11B_ = 1.3 ppm), isolated in 83% (Ar = Phen) and 86% (Ar = Pyr) yields (Scheme 2b).

**Fig. 3:**
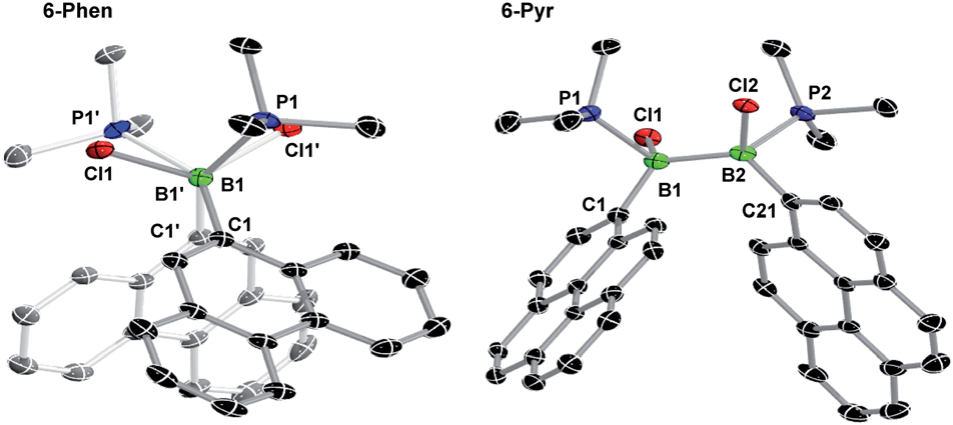
Crystallographically-derived solid-state structures of 6-Phen (view along the B–B axis showing the eclipsed conformation) and 6-Pyr. Atomic displacement ellipsoids at 50%. Hydrogen atoms omitted for clarity. Selected bond lengths (Å) and angles (°) for 6-Phen: B1–B1′ 1.776(4), B1–P1 1.9950(19), B1–Cl1 1.9719(19), B1–C1 1.629(2), torsion C1–B1–B1′–C1′ 14.4(2); for 6-Pyr: B1–B2 1.779(7), B1–P1 1.997(5), B1–Cl1 1.974(5), B1–C1 1.637(7), B2–P2 2.005(5), B2–Cl2 1.969(5), B1–C21 1.634(6), torsion C1–B1–B2–C21 13.3(6).

The solid-state structures of 6-Phen and 6-Pyr show an unexpected eclipsed conformation of the substituents at the two boron atoms (C–B–B–C and P–B–B–Cl torsion angles 10.1–14.8°, Fig. 3). This contrasts with the staggered conformation usually observed in doubly base-stabilized di- and tetrahalodiboranes, which minimises steric repulsion.^31–34^ The eclipsed conformation might be favoured in this case by attractive interactions between the two PAH groups. The B–B bond lengths of 1.776(4) and 1.779(7) Å for 6-Phen and 6-Pyr, respectively, are within the range typical for acyclic doubly base-stabilised diboranes.^31–34^

### Synthesis of PAH-substituted diborenes

The reduction of 6-Ar with 2.5 equiv. KC_8_ in a 5 : 1 benzene–THF solvent mixture at room temperature over a period of 4 h resulted in a colour change to red for 7-Phen and blue for 7-Pyr (Scheme 2c). NMR spectra of the filtered reaction mixtures showed 60–80% conversion to the diborenes 7-Ar, which display a broad ^11^B NMR resonance at *ca.* 21 ppm and a broad ^31^P NMR singlet at *ca.* −22 ppm, similar to the dianthryldiborene III (*δ*_11B_ = 22 ppm, *δ*_31P_ = −21.3 ppm).^12^ Interestingly, the ^1^H NMR spectrum of 7-Phen showed the presence of two isomers, which were identified as two rotamers of 7-Phen, the centrosymmetric species 7a-Phen and its isomer 7b-Phen, in which one of the phenanthryl substituents is rotated by 180° compared to 7a-Phen (Fig. 5). Calculations showed that these two rotamers are only 0.4 kcal mol^−1^ apart in energy (see ESI† for details), which is in agreement with their experimentally observed formation in a nearly 1 : 1 ratio. The UV-vis spectrum of 7-Phen showed several strong, overlapping absorptions in the 350–400 nm range and a low-intensity broad absorption band centred around 480 nm, accounting for its red colour. Unfortunately, 7-Pyr was too unstable in solution to acquire meaningful UV-vis data.

**Fig. 4:**
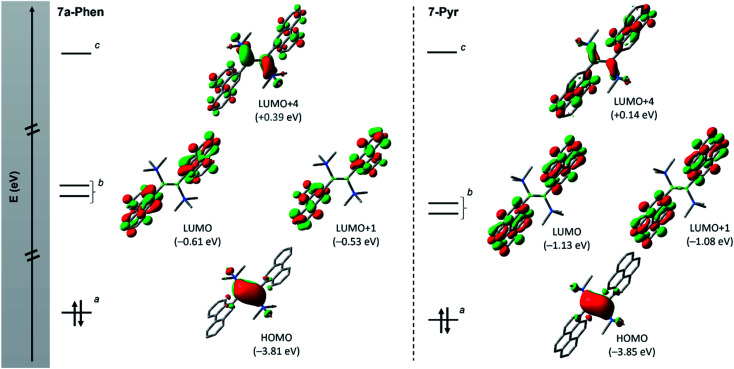
Frontier orbitals (isovalue: ±0.04) of diborenes 7a-Phen (left) and 7-Pyr (right) showing the orbital intercalation between PAH and diborene units. ^*a*^ π orbital of the B=B bond; ^*b*^ π* orbitals of PAH units; and ^*c*^ π* orbital of the B=B bond. Note: molecular orbitals LUMO+2 and LUMO+3 corresponding to other combination of π* orbitals of PAH units are not shown (see ESI†).

**Fig. 5:**
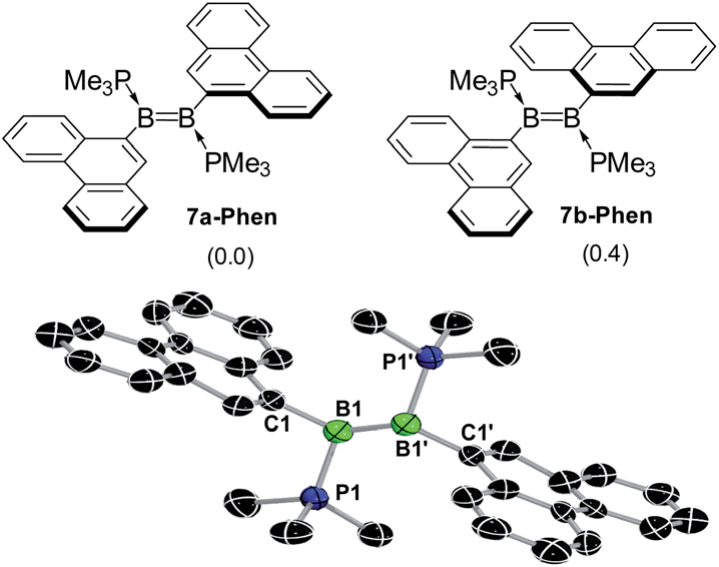
Top: the two rotamers of 7-Phen, 7a-Phen and 7b-Phen. Energies (kcal mol^−1^) in parentheses calculated at the M06/6-31g(d,p)/SMD(THF) level of theory. Bottom: crystallographically-derived solid-state structure of 7a-Phen. Atomic displacement ellipsoids at 50%. Ellipsoids of ligand periphery and hydrogen atoms omitted for clarity. Structural parameters cannot be discussed due to heavy structural disorder and bonding restraints applied during refinement.

A single-crystal X-ray diffraction analysis of 7a-Phen confirmed the formation of the diborene (Fig. 5). The structure was entirely disordered in a 1 : 1 ratio *via* a mirror plane orthogonal to the plane of the B=B unit and bisecting the B–B bond, thus precluding a detailed analysis of bonding parameters (see ESI† for details). The same disorder was also found in the solid-state structure of the anthryl analogue III.^12^ The B–B bond length of *ca.* 1.56 Å (average of the two overlapping diborenes) is within the range usually observed in diborenes (1.52–1.62 Å).^1,12,21,35^

The computed structure of 7a-Phen also displays a very similar B–B bond length (1.57 Å, see ESI†). Additionally, the π core of the B=B double bond and that of the phenanthryl units are likely unconjugated, as the C2–C1–B1–B1′ torsion angle is *ca.* 78.7°. The computed structure of 7-Pyr also shows a very similar B–B bond length (1.575 Å) and a larger C2–C1–B1–B1′ twist of 105.4°.

Fig. 4 depicts the frontier molecular orbitals (MOs) of 7a-Phen and 7-Pyr. For comparison, such details were also computed for diborene III at the same level of theory (see ESI†). Diborenes 7a-Phen and 7-Pyr exhibit a similar arrangement of frontier orbitals and orbital energies as III, with the low-lying unoccupied π* orbitals of the phenanthryl and pyrenyl residues intercalated between the π and π* orbitals of the B–B bond to furnish a small HOMO–LUMO gap (HLG). As in III,^12^ the HOMO of 7a-Phen and 7-Pyr is a B–B π-bonding orbital, and the LUMO and LUMO+1 are in-phase and anti-phase π* orbitals, respectively, exclusively spread over both PAH units. Energetically, the frontier MOs HOMO to LUMO+4 in all three diborenes lie in a similar range (−3.85 eV to +0.39 eV). In terms of engineering a small HLG in diborenes by incorporating PAH substituents, phenanthryl and pyrenyl groups show no advantage over anthryl groups, even if the HLG of 7-Pyr is close to that of III (HLGs for 7a-Phen: 3.20 eV; 7-Pyr: 2.72 eV; III: 2.53 eV).^12^

**Scheme 3:**
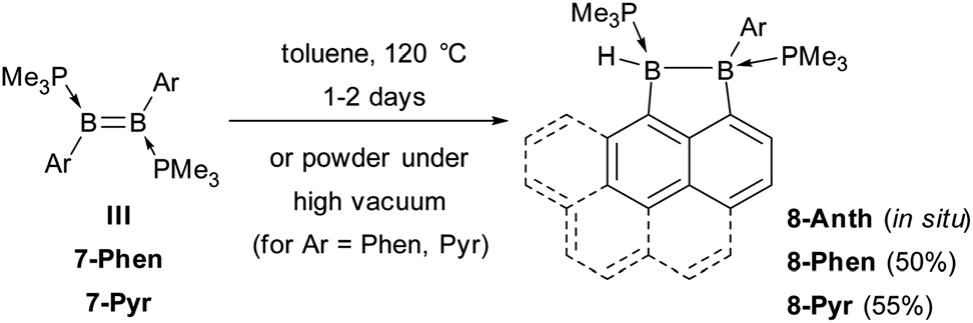
Intramolecular C–H activation in diborenes III and 7-Ar.

During the reduction of 6-Ar to 7-Ar, a small amount of by-product 8-Ar was formed, which displays two broad ^11^B NMR resonances at −13 and −22 ppm, and two broad ^31^P NMR doublets at −5 and −9 ppm (^3^*J*_PP_ = 84 Hz), indicative of an unsymmetrical doubly PMe_3_-stabilised diborane. Upon removing all volatiles *in vacuo*, the diborene 7-Ar converted entirely to 8-Ar, isolated as a colourless solid in 50% yield for Ar = Phen and as a yellow solid in 55% yield for Ar = Pyr (Scheme 3). The ^1^H NMR spectrum of 8-Phen showed a very broad B*H* resonance in the 2.4–3.2 ppm region, which resolved to an apparent triplet upon ^11^B decoupling (^2/3^*J*_HP_ = 17.0 Hz).

**Fig. 6:**
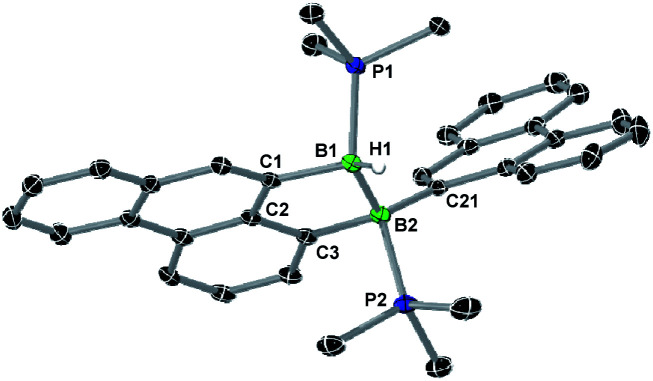
Crystallographically-derived solid-state structure of 8-Phen. Atomic displacement ellipsoids at 50%. Hydrogen atoms omitted for clarity, except boron-bound H1.‡ Selected bond lengths (Å) and angles (°): B1–B2 1.795(2), B1–P1 1.9348(17), B1–C1 1.6221(18), B1–H1 1.148(17), B2–P2 1.9806(17), B2–C21 1.6230(19), B2–C3 1.6381(18).

A single-crystal X-ray analysis showed that 8-Phen is the product of the intramolecular hydroarylation of diborene 7-Phen, which generates a doubly PMe_3_-stabilised 4-(9-phenanthryl)-5-hydro-phenanthro[1,10-*cd*][1,2]diborole (Fig. 6). A similar intramolecular hydroarylation reaction has been observed for III, triggered by the coordination of copper triflate to the B=B double bond and followed by hydride abstraction and PMe_3_ migration,^36^ as well as for a 9-anthryl-substituted disilane under photolytic conditions.^37^ In the case of 7-Phen and 7-Pyr this reaction can be triggered either by removal of all volatiles from the reaction mixture (free PMe_3_ in the reaction mixture seems to stabilise 7-Ar) or by heating the reaction mixture for 2 days at 120 °C for complete conversion. The anthryl derivative III could also be partially converted to 8-Anth over a period of several days in refluxing toluene but was stable under vacuum. This increased reactivity of 7-Phen and 7-Pyr compared to III is likely due to the lower steric shielding of the boron centres provided by the unsubstituted *ortho* position of the 9-phenanthryl and 1-pyrenyl ligands. The B–B bond in 8-Phen (1.795(2) Å) is slightly longer than in the hydroarylated anthryl derivative (1.782(2) Å)^36^ due to the additional presence of the hydride ligand at B1. The phenanthro[1,10-*cd*][1,2]diborole ring is not fully planar and shows a slight curvature of the PAH backbone (the mean planes of the three C_6_ rings form angles of 1.9 and 4.1° between each other) and a slight twist in the 1,2-diborole ring (torsion angles C1–B1–B2–C3 11.35(11), C2–C3–B2–B1 −9.43(12), C2–C1–B1–B2 −10.70(12)°) caused by the ring strain of the C_3_B_2_ ring.‡The boron-bound hydrides were detected in the inverse Fourier map and freely refined.

### Hydrogenation of B_2_Ar_2_(PMe_3_)_2_

**Scheme 4:**
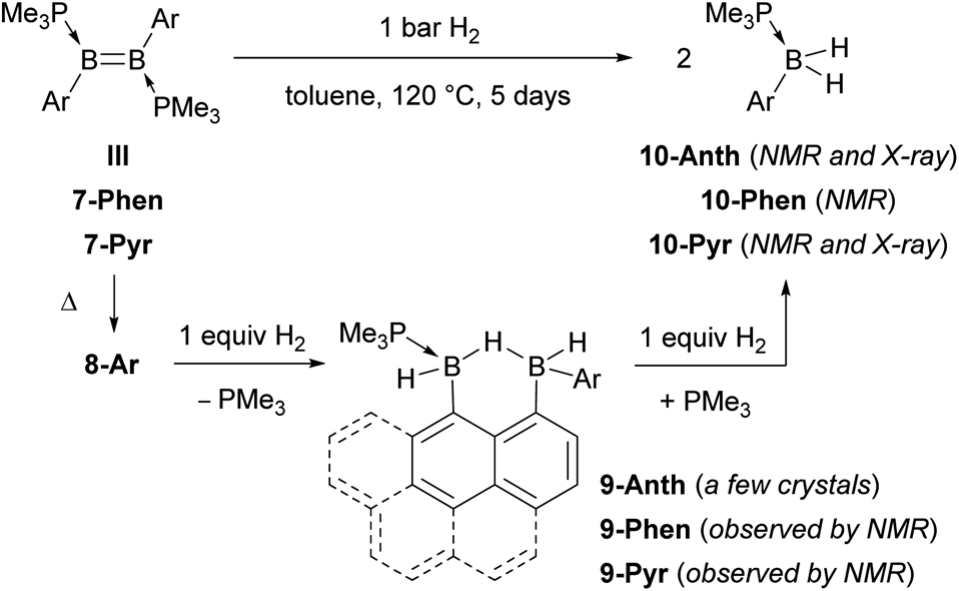
Hydrogenation of III and 7-Ar.

**Fig. 7:**
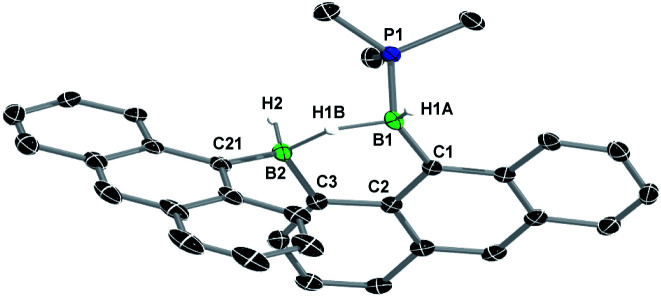
Crystallographically-derived solid-state structure of 9-Anth. Atomic displacement ellipsoids at 50%. Hydrogen atoms omitted for clarity, except boron-bound hydrides.‡ Selected bond lengths (Å) and angles (°): B1–P1 1.948(3), B1–C1 1.597(4), B1–H1A 1.11(4), B1–H1B 1.26(4), B2–H1B 1.41(4), B2–H2 1.12(3), B2–C21 1.611(13), B2–C3 1.600(4).

Heating a toluene solution of III or 7-Ar at 120 °C for 5 days under 1 bar of H_2_ resulted in twofold hydrogenation and splitting of the B–B bond to yield the phosphine-stabilised arylboranes 10-Ar, characterised by an ^11^B{^1^H} NMR doublet at *ca.* −25 ppm (^2^*J*_BP_ ≈ 50 Hz) and a broad ^31^P{^1^H} NMR multiplet around −8 ppm (Scheme 4). During the course of these reactions an intermediate, 9-Ar, with two ^11^B NMR resonances in a 1 : 1 ratio at around −23 and −27 ppm, was observed but could not be isolated. The hydrogenation of the known diborene III in toluene at 120 °C under 1 bar of H_2_, however, proceeded more slowly, leading to the isolation of a few crystals of the intermediate 9-Anth suitable for X-ray diffraction analysis after 2 days. The solid-state structure of 9-Anth shows a hydride-bridged 9-boranylanthracene-1-yl(anthrylborane), stabilised by a single PMe_3_ ligand, which results from the single hydrogenation of the B–B bond of 8-Anth with loss of one PMe_3_ ligand (Fig. 7). In the reaction shown in Scheme 4, this first step is followed by the addition of a second equivalent of H_2_ across the B2–C3 bond of 9-Ar, yielding 10-Ar and ArBH_2_, the latter forming an adduct with the remaining PMe_3_ ligand to yield the second equivalent of 10-Ar.

The reaction is remarkable in that it involves the uncatalysed hydrogenative cleavage of a strong B–C bond, thus effectively rendering the intramolecular hydroarylation process reversible. To our knowledge, the only other example of B–C bond-cleaving hydrogenation is the palladium-catalysed hydrogenation of a boratirene, resulting in ring-opening of the strained BC_2_ heterocycle.^38^ Interestingly, Piers proposed that the formation of *cis*- and *trans*-isomers in the 2,5-hydrogenation of pentaarylboroles can be explained by the first reaction step being the 1,2-hydrogenation of the endocyclic B–C bond, followed by rotation of the remaining B–C bond and finally cyclisation to the *cis*/*trans*-boracyclopent-3-ene. The putative ring-opened 1,3-butadienylborane intermediate of this reversible B–C bond-cleaving hydrogenation, however, was not detected.^39^

**Scheme 5:**
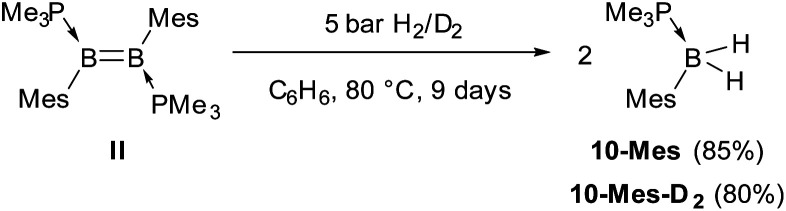
Hydrogenation of II.

In comparison, the hydrogenation of the related dimesityldiborene II required heating in benzene for 9 days at 80 °C under 5 bar of H_2_ (Scheme 5). Although the final product of the hydrogenation was also the phosphine-stabilised arylborane 10-Mes, NMR-spectroscopic monitoring during the course of the reaction did not show any intermediates. In contrast, the hydrogenation of the related diiododiborene B_2_I_2_(PMe_3_)_2_ proceeded stepwise, enabling the isolation of the single hydrogenation intermediate B_2_I_2_H_2_(PMe_3_)_2_.^40^

### Mechanistic computational analyses

**Fig. 8:**
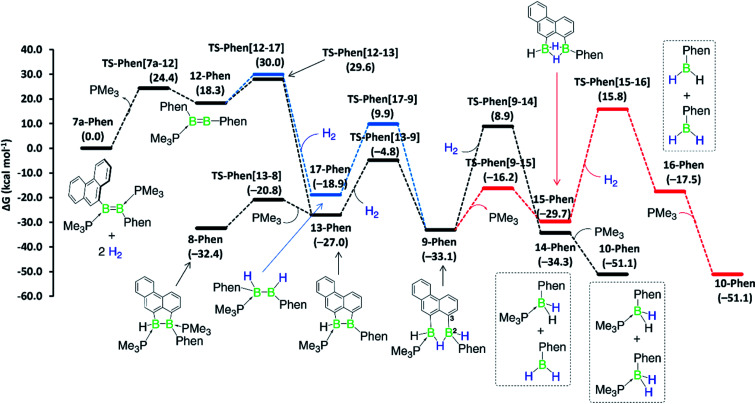
Mechanisms computed for the hydrogenation of diborene 7-Phen at the M06/6-31g(d,p)/SMD(toluene) level of theory. Black profile: hydrogenation *via* initial H–H activation pathway; blue profile: hydrogenation *via* initial C–H activation pathway; and red profile: second hydrogenation entailing the bis(monoborane) intermediate 15-Phen. Free energies in parentheses.

**Fig. 9:**
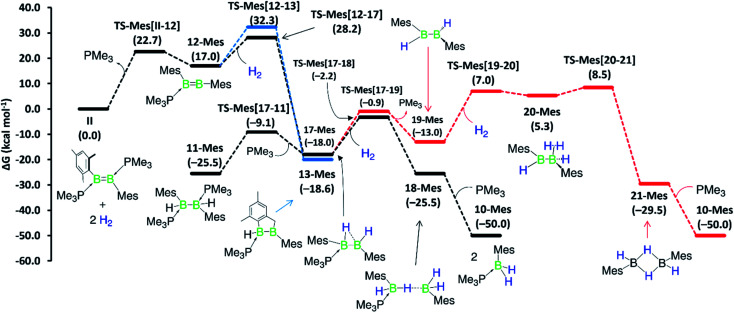
Mechanisms computed for the hydrogenation of diborene II at the M06/6-31g(d,p)/SMD(benzene) level of theory. Black profile: hydrogenation *via* initial H–H activation pathway and the more likely pathway; blue profile: hydrogenation *via* initial C–H activation pathway; and red profile: second hydrogenation involving the diborane(4) intermediate 19-Mes. Free energies in parentheses.

Although a similar hydrogenation mechanism to that of III, 7-Phen and 7-Pyr may be envisaged for diborene II, since mesityl-substituted diboron compounds are known to undergo intramolecular C–H activation at one *ortho*-methyl group to form a 2-benzyl unit bridging two boron centres,^41,42^ the known hydrogenation mechanism for B_2_I_2_(PMe_3_)_2_*via* B_2_I_2_H_2_(PMe_3_)_2_^40^  makes a similar stepwise hydrogenation of II*via* B_2_Mes_2_H_2_(PMe_3_)_2_ more likely. We envisage that the initial competition for activating the intramolecular aryl C–H bond of fused arenes or the H–H bond of H_2_ by the diborenes, after PMe_3_ dissociation, decides the fate of the mechanism. This view puts forward that diborenes 7-Phen, 7-Pyr and III perhaps follow the C–H activation route, while diborene II, lacking a nearby aryl C–H bond, may follow the H–H activation route. To ascertain this, computations were performed on both pathways for all diborenes. The pathways computed for 7a-Phen and II are shown in Fig. 8 and 9, respectively. Since diborenes 7-Pyr and III pose similar mechanistic steps and energetics to 7a-Phen, their free energy profiles are provided in the ESI (Fig. S80 and S81,† respectively). The most likely reaction pathway with the lowest barriers is provided as a black profile.

The twofold hydrogenation of all diborenes to the boranes **10-Ar** is substantially exergonic, with Δ*G* ranging from −47.6 to −51.1 kcal mol^−1^. Although the initial step of PMe_3_ dissociation from all diborenes, leading to intermediate 12-Ar, is significantly endergonic, it has a relatively low energy barrier of 22.7 to 24.4 kcal mol^−1^. In the next step, intermediates 12-Phen, 12-Pyr and 12-Anth all show almost an equal propensity towards C–H (black profile) and H–H activation (blue profile), with the overall energy barriers for these two processes from 7-Ar and III separated by only 0.4 to 2.1 kcal mol^−1^ (Fig. 8, S80 and S81†). For these PAH-substituted diborenes the formation of both 13-Ar and 17-Ar, and eventually both mechanisms, are therefore energetically accessible. Nevertheless, the experimentally observed intermediate 8-Ar, formed by reassociation of PMe_3_ to 13-Ar, is in all cases thermodynamically favoured over the direct hydrogenation intermediate 17-Ar by 5.5 to 13.5 kcal mol^−1^. Both pathways lead to intermediate 9-Ar, in one case by C–H activation of one aryl moiety at 17-Ar, in the other by hydrogenation of 13-Ar. As mentioned above, 9-Ar forms the product 10-Ar by consuming the second equivalent of H_2_*via* direct H–H activation across the B2–C3 bond in 9-Ar, followed by PMe_3_ association. If we consider 9-Ar as the major resting species, the activation of the second H_2_ equivalent by 9-Ar, leading to 14-Ar, *i.e.* the hydrogenative cleavage of the endocyclic B–C bond, becomes rate-limiting (Δ*G*^‡^ ≈ 42 kcal mol^−1^, TS-Ar(9–14)) and explains the high reaction temperature required for full conversion. The alternative pathway, *via* loss of PMe_3_ to generate 15-Ar, followed by H_2_ activation (red profile), is energetically less favourable by 3 to 5 kcal mol^−1^.

Competition for the initial C–H or H–H activation is not seen with diborene **II**, as activating the *ortho*-methyl C–H bond of the mesityl substituent is energetically less favoured (Δ*G*^‡^ = 32.3 kcal mol^−1^, TS-Mes(12–13)) than the activation of H_2_ (Δ*G*^‡^ = 28.2 kcal mol^−1^, TS-Mes(12–17), Fig. 9). Thus, intermediate 12-Mes undergoes H_2_ activation across the B=B bond, leading to the resting species, the doubly PMe_3_-stabilised 1,2-dihydro-1,2-dimesityldiborane 11-Mes. This species loses one PMe_3_, leading to the hydride-bridged diborane 17-Mes, which activates the second equivalent of H_2_ across the B–B single bond, ultimately forming the product 10-Mes. Following previous reports of symmetrical di- and tetraaryldiboranes(4) spontaneously activating dihydrogen across their B–B bond,^43,44^ this pathway was also computed using the diborane(4) intermediate 19-Mes, generated from 17-Mes through PMe_3_ dissociation, but proved less competitive for the formation of 10-Mes (red profile). For the whole hydrogenation reaction of diborene II, the first H_2_ activation step (II-to-17-Mes) is rate-limiting (Δ*G*^‡^ = 28.2 kcal mol^−1^).

Overall, computations reveal that for diborenes 7-Phen, 7-Pyr and III, both C–H and H–H activation pathways initially compete almost equally to establish the observed resting species 9-Ar, while the second H_2_ addition proceeds directly across the B–C bond of 9-Ar. In contrast, for diborene II, it is clear-cut that both the first and second hydrogenation occur essentially across the B–B core.

## Conclusions

Unlike the straightforward syntheses of the PMe_3_-stabilised dimesityl- and dianthryldiborenes II and III, the synthesis of the analogous 9-phenanthryl and 1-pyrenyl-substituted diborenes 7-Phen and 7-Pyr is fraught with complications owed to the absence of a second *ortho* substituent at the boron-bound aryl rings. The reduced steric bulk of these PAH substituents enables their free rotation, thus exposing the B–B bond of both diborane precursors and diborenes to decomposition. Systematic B–B bond cleavage during the usual synthetic route *via* B_2_Ar_2_(OMe)_2_ precursors can be circumvented by direct NMe_2_–Cl exchange at B_2_Ar_2_(NMe_2_)_2_ precursors with (Me_2_S)BCl_3_. The resulting isolable SMe_2_ adducts of B_2_Ar_2_Cl_2_ can be converted to the PMe_3_ adducts, which can in turn be reduced to the desired diborenes 7-Phen and 7-Pyr. The facile rotation of the 9-phenanthryl substituent is evidenced by the existence of two energetically equivalent rotational isomers of 7-Phen in solution. DFT calculations show that, as in III, the low-lying unoccupied π* orbitals of the PAH residues of 7-Phen and 7-Pyr are intercalated between the π and π* orbitals of the B–B bond to furnish a small HOMO–LUMO gap, albeit a slightly larger one than in III. Both 7-Phen and 7-Pyr were only stable in solution at rt in the presence of PMe_3_, thus marring their clean isolation in the solid state. At high temperature in solution or under vacuum 7-Phen and 7-Pyr underwent intramolecular hydroarylation of the B=B bond to yield the 1,2-dihydronaphtho[1,8-*cd*][1,2]diborole derivatives 8-Ar. The analogous 8-Anth could also be obtained, albeit in much lower yield, by refluxing III in toluene for several days.

The twofold hydrogenation of the PAH-substituted diborenes with 1 bar H_2_ required prolonged reaction times and high temperatures. NMR-spectroscopic monitoring of the reactions showed that they proceeded through the formation of the hydroarylation intermediates 8-Ar, followed by hydrogenation of one boron moiety of 8-Ar to yield the intermediates 9-Ar, of which the anthryl derivative was structurally characterised, and finally a second hydrogenation of the B–C bond resulting from hydroarylation to yield the monoboranes (Me_3_P)BArH_2_ (10-Ar). The hydrogenation of the mesityl derivative II, which required 5 bar H_2_, longer reactions times and yielded the analogous monoborane 10-Mes, did not show any stable reaction intermediates. Computational mechanistic analyses using DFT showed that the hydrogenation of II proceeds by two successive H_2_ additions to the B–B bond. In contrast, hydrogenation of the PAH derivatives can proceed either *via* H_2_ addition at the B–B bond first and intramolecular hydroarylation second, or *vice versa*, both pathways converging in the second H_2_ addition, which splits the B–C bond formed in the hydroarylation step. This unprecedented, uncatalysed hydrogenative B–C bond cleavage constitutes the rate-limiting step and effectively reverses the intramolecular hydroarylation process.

Finally, it must be noted that the use of phenanthryl and pyrenyl substituents in both diborane and diborene chemistry affords clear disadvantages over hitherto employed aryl substituents. This is due on the one hand to the lack of steric protection of the B–B bond, which often leads to decomposition, on the other hand to the insoluble nature of both the desired products and side products, which frustrates efforts to purify the compounds.

## Data availability

The datasets supporting this article have been uploaded as part of the ESI.†

## Author contributions

A. O. designed and carried out 90% of the experimental work. L. E., T. S. and J. W. carried out the remaining 10% of the experimental work. A. J. carried out the majority of theoretical calculations. M. A. wrote the manuscript, refined X-ray structures and carried out preliminary computations. A. O., A. J., L. E. and M. A. co-wrote the ESI. C. B., M. H., A. L., C. L. and M. R. carried out X-ray crystallographic experiments. H. B. designed the original project and provided the funding.

## Conflicts of interest

There are no conflicts to declare.

## Supplementary Material

SC-013-D2SC02515A-s001

SC-013-D2SC02515A-s002
