# Supplementary material for: Synthesis and hydrogenation of polycyclic aromatic hydrocarbon-substituted diborenes via uncatalysed hydrogenative B–C bond cleavage
Source: Chem Sci. 2022 Jun 14;13(25):7566–74. doi: 10.1039/d2sc02515a (PMC9242019; doi:10.1039/d2sc02515a)
Supplement: SC-013-D2SC02515A-s001 [file SC-013-D2SC02515A-s001.pdf]

Electronic Supplementary Material (ESI) for Chemical Science.
This journal is © The Royal Society of Chemistry 2022

Electronic Supporting Information for

Synthesis and hydrogenation of polycyclic aromatic hydrocarbon-substituted diborenes via uncatalysed hydrogenative B–C bond cleavage

Alexander Okorn,[a,b] Arumugam Jayaraman,[a,b] Lukas Englert,[a,b] Merle Arrowsmith,[a,b] Theresa Swoboda,[a,b] Jeanette Weigelt,[a,b] Carina Brunecker,[a,b] Merlin Hess,[a,b] Anna Lamprecht,[a,b] Carsten Lenczyk,[a,b] Maximilian Rang,[a,b] and Holger Braunschweig*[a,b]

[a] *Institute for Inorganic Chemistry, Julius-Maximilians-Universität Würzburg, Am Hubland, 97074 Würzburg, Germany.* [b] *Institute for Sustainable Chemistry & Catalysis with Boron, Julius-Maximilians-Universität Würzburg, Am Hubland, 97074 Würzburg, Germany.*

Contents

Methods and materials 2

Synthetic procedures 3

NMR spectra of isolated compounds 18

NMR spectra of products which could not be isolated in > 90% purity. 59

^{11}B NMR spectra of the attempted syntheses of **2-Phen** and **2-Pyr** 75

NMR-monitoring of the addition of H_2 to **7-Ar** 84

UV-vis spectra 87

X-ray crystallographic data 88

Computational details 93

- Computations on the rotational isomers of **2-Phen** and **2-Pyr** 94
- Computed structures and energies of the two rotational isomers of **7-Phen** 99
- Frontier orbitals and associated energies of diborenes **7-Phen**, **7-Pyr** and **III** 101
- Mechanistic pathways for the hydrogenation of **7a-Phen** 104
- Mechanistic pathways for the hydrogenation of **7-Pyr** 118
- Mechanistic pathways computed for the hydrogenation of **III** 133
- Mechanistic pathways for the hydrogenation of diborene **II** 146

References 160

Methods and materials

All manipulations were performed either under an atmosphere of dry argon or *in vacuo* using standard Schlenk line or glovebox techniques. Deuterated solvents were dried over molecular sieves and degassed by three freeze-pump-thaw cycles prior to use. All other solvents were distilled and degassed from appropriate drying agents. Both deuterated and non-deuterated solvents were stored under argon over activated 4 Å molecular sieves. Liquid-phase NMR spectra were acquired on a Bruker Avance 500 spectrometer (^{1}H: 500.1 MHz, ^{11}B: 129.9 or 160.5 MHz, ^{77}Se: 95.4 MHz) at 297 K, unless specified otherwise. Chemical shifts (δ) are reported in ppm and internally referenced to the carbon nuclei ($^{13}C\{^{1}H\}$) or residual protons (^{1}H) of the solvent. Heteronuclei NMR spectra are referenced to external standards (^{11}B: $BF_3{\cdot}OEt_2$, ^{31}P: 85% H_3PO_4 in H_2O). Resonances are given as singlet (s), doublet (d), triplet (t), septet (sept) or multiplet (m). Microanalyses (C, H, N, S) were performed on an Elementar vario MICRO cube elemental analyzer. High-resolution mass spectrometry (HRMS) data were obtained from a Thermo Scientific Exactive Plus spectrometer. UV-vis spectra were acquired on a METTLER TOLEDO UV-vis-Excellence UV5 spectrophotometer inside a glovebox.

Solvents and reagents were purchased from Sigma-Aldrich or Alfa Aesar. $B_2An_2(PMe_3)_2$ (Anth = 9-anthryl, **III**),[1] $B_2Mes_2(PMe_3)_2$ (Mes = 2,4,6-trimethylphenyl, **II**)[2] and $B_2Cl_2(NMe_2)_2$[3] were synthesised using literature procedures.

Synthetic procedures

Note: some of the products could not be isolated in sufficient purity for full NMR characterisation due to their very low solubility (and that of the reaction byproducts) even in highly polar organic solvents. Their identity was, however, ascertained by comparison of the NMR data with that of cleanly isolated analogues and HRMS data, as well as in some cases X-ray crystallographic analysis.

Synthesis of 1-Phen

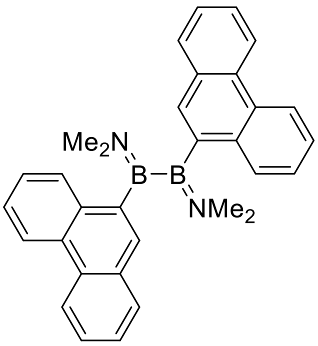

To a stirred suspension of 9-bromophenanthrene (15.3 g, 59.5 mmol) in Et_2O (400 mL) at –78 °C a solution of ^{n}BuLi in hexane (1.60 M, 40.0 mL, 64.0 mmol, 1.10 equiv.) was slowly added. The reaction mixture was then allowed to warm up to rt and stirred for another hour. The resulting colourless suspension was cooled to –78 °C again and a solution of $B_2Cl_2(NMe_2)_2$ (5.37 g, 29.7 mmol, 0.50 equiv.) in Et_2O (30 mL) was added dropwise. The reaction mixture was allowed to warm up to rt and stirred overnight. All volatiles were removed *in vacuo* and the residual solid extracted with warm benzene (3 x 100 mL). The benzene solution was dried *in vacuo* and the residual solid washed with hexane (3 x 25 mL) before drying it again *in vacuo* to yield **1-Phen** as a colourless powder (12.1 g, 26.1 mmol, 88%). Colourless single crystals of **1-Phen** suitable for X-ray crystallographic analysis were obtained from slow evaporation of a saturated benzene solution. ^{1}H NMR (500.1 MHz, d_8-tol): δ = 8.47-8.36 (m, 4H, Ar-*H*), 7.78-7.18 (m, 14H, Ar-*H*), 3.00 (s, 6H, N(C*H*$_3$)$_2$), 2.29 (s, 6H, N(C*H*$_3$)$_2$) ppm. ^{11}B NMR (128.4 MHz, C_6D_6): δ = 51.3 (br) ppm. ^{13}C{^{1}H} NMR (125.8 MHz, d_8-tol): δ = 143.9 (B*C*), 133.9 (Ar-*C*$_q$), 132.3 (Ar-*C*$_q$), 130.5 (Ar-*C*$_q$), 130.2 (Ar-*C*$_q$), 130.1 (Ar-*C*H), 129.7 (Ar-*C*H), 126.6 (Ar-*C*H), 126.3 (Ar-*C*H), 126.2 (Ar-*C*H), 123.4 (Ar-*C*H), 123.2 (Ar-*C*H), 123.0 (Ar-*C*H), 122.8 (Ar-*C*H), 44.7 (N*C*H$_3$), 44.6 (N*C*H$_3$), 40.8 (N*C*H$_3$), 40.7 (N*C*H$_3$) ppm. HRMS ESI pos-MS (*m/z*) calculated for $[C_{32}H_{30}B_2N_2]^+$: 464.2595; found: 464.2590.

Synthesis of 1-Pyr

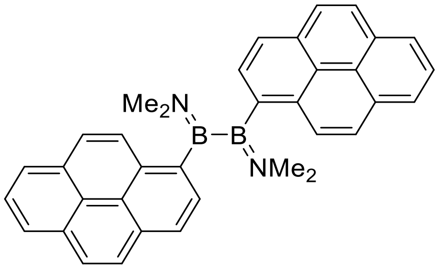

To a stirred suspension of 1-bromopyrene (14.4 g, 51.2 mmol) in Et_2O (400 mL) at –78 °C a solution of ^{n}BuLi in hexane (1.60 M, 33.6 mL, 53.8 mmol, 1.10 equiv.) was slowly added. The reaction mixture was then allowed to warm up to rt and stirred for another hour. The resulting yellow suspension was cooled to –78 °C again and a solution of $B_2Cl_2(NMe_2)_2$ (4.63 g, 25.6 mmol, 0.5 equiv.) in Et_2O (30 mL) was added dropwise. The reaction mixture was allowed to warm up to rt and stirred overnight. All volatiles were removed *in vacuo* and the residual solids extracted with warm benzene (3 x 150 mL). The benzene solution was dried *in vacuo* and the residual solid was washed with hexane (3 x 25 mL) before drying again residual to yield **1-Pyr** as a yellow powder (9.40 g, 18.3 mmol, 71%). Yellow single crystals of **1-Pyr** suitable for X-ray crystallographic analysis were obtained from slow diffusion of hexane into a saturated benzene solution. ^{1}H NMR (500.1 MHz, C_6D_6): δ = 7.92-7.74 (m, 18H, Ar-*H*), 3.21 (s, 6H, N(C*H*$_3$)$_2$), 2.44 (s, 6H, N(C*H*$_3$)$_2$) ppm. ^{11}B NMR (160.5 MHz, C_6D_6): δ = 50.5 (br) ppm. ^{13}C{^{1}H} NMR (125.8 MHz, C_6D_6): δ = 144.1 (B*C*), 132.1 (Ar-*C*$_q$), 131.9 (Ar-*C*$_q$), 131.7 (Ar-*C*$_q$), 130.6 (Ar-*C*$_q$), 129.0 (Ar-*C*H), 128.6 (Ar-*C*H), 127.0 (Ar-*C*H), 126.6 (Ar-*C*H), 126.1 (Ar-*C*H), 125.9 (Ar-*C*$_q$), 125.8 (Ar-*C*H), 125.4 (Ar-*C*$_q$), 125.3 (Ar-*C*H), 124.8 (Ar-*C*H), 124.7 (Ar-*C*H), 44.8 (N(*C*H$_3$)$_2$), 40.8 (N(*C*H$_3$)$_2$) ppm. HRMS ESI pos-MS (*m/z*) calculated for [$C_{36}H_{30}B_2N_2$]: 512.2595; found: 512.2590.

Attempted synthesis of 2-Phen and isolation of 4-Phen

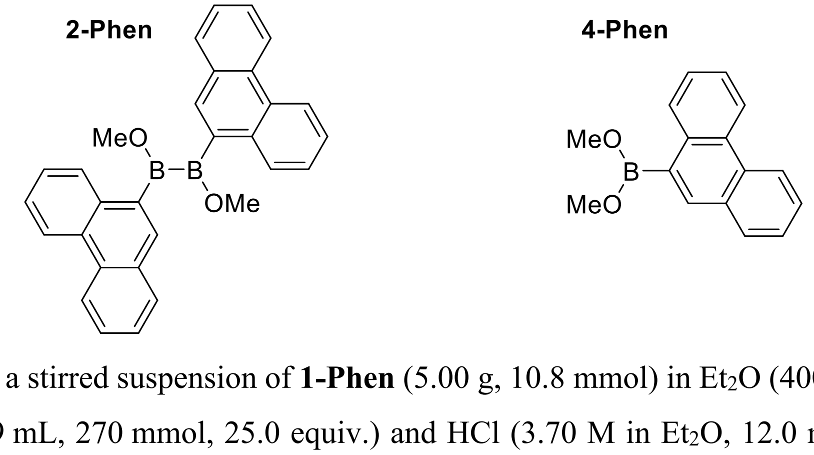

Method A. To a stirred suspension of **1-Phen** (5.00 g, 10.8 mmol) in Et_2O (400 mL) at –78 °C methanol (10.9 mL, 270 mmol, 25.0 equiv.) and HCl (3.70 M in Et_2O, 12.0 mL, 44.4 mmol,

4.10 equiv.) were added. The reaction mixture was allowed to warm up to rt and stirred overnight. ^{11}B NMR-spectroscopic analysis of the reaction mixture showed full conversion of **1-Phen** to ca. 90% **2-Phen** (δ_{11B} = 58.7 ppm) and 10% **4-Phen** (δ_{11B} = 31.2 ppm). All volatiles were removed *in vacuo* and the residual solid extracted with benzene (3 x 100 mL), prior to drying *in vacuo*. The residual solid was washed with hexane (3 x 25 mL) and again dried *in vacuo* to yield **4-Phen** as a pale yellow powder, the sole isolable product (4.21 g, 16.7 mmol, 77%). ^{1}H NMR (400.1 MHz, C_6D_6): δ = 8.54-8.51 (m, 1H, Ar-*H*), 8.47 (d, $^{3}J_{HH}$ = 7.9 Hz, 1H, Ar-*H*), 7.99-7.96 (m, 1H, Ar-*H*), 7.72 (s, 1H, Ar-*H*), 7.67 (d, $^{3}J_{HH}$ = 7.9 Hz, 1H, Ar-*H*), 7.44-7.39 (m, 4H, Ar-*H*), 3.52 (s, 6H, OCH_3) ppm. ^{11}B NMR (128.4 MHz, C_6D_6): δ = 30.9 (s) ppm.
Method B. **1-Phen** (150 mg, 320 μmol), MeOH (330 μL, 8.08 mmol, 25.0 equiv.) and HCl (3.70 M in Et_2O, 360 μL, 1.32 mmol, 4.10 equiv.) in Et_2O (30 mL) at –78 °C, then 4 h at rt. ^{11}B NMR-spectroscopic analysis of the reaction mixture showed full conversion of **1-Phen** to ca. 80% **2-Phen** and 20% **4-Phen**. The volume of the mixture was reduced by 2/3 *in vacuo*, 30 mL benzene were added and the solution decanted to a second flask, prior to volume reduction by half *in vacuo* and precipitation of the product with 20 mL hexane. After decantation the solid was washed with hexane (2 x 10 mL) before drying it *in vacuo* to yield **4-Phen** as the sole isolable product (89.0 mg, 370 μmol, 58%).
Method C. Same reagent quantities as **method B**, in toluene at –78 °C, then 4 h at rt. ^{11}B NMR-spectroscopic analysis of the reaction mixture showed full conversion of **1-Phen** to ca. 50% **2-Phen** and 50% **4-Phen**. Extraction and isolation as in **method A**, yielding **4-Phen** as a pale yellow powder (101 mg, 420 μmol, 66%).
Method D. **1-Phen** (500 mg, 1.08 mmol), MeOH (90.0 μL, 2.26 mmol, 2.10 equiv.) and HCl (3.70 M in Et_2O, 610 μL, 2.26 mmol, 2.10 equiv.) in Et_2O (50 mL) at –78 °C, then 4 h at rt. ^{11}B NMR-spectroscopic analysis of the reaction mixture showed full conversion of **1-Phen** to ca. 90% **2-Phen** and 10% **4-Phen**. Extraction and isolation as in **method A**, yielding **4-Phen** as the sole isolable product (184 mg, 740 μmol, 34%).
Method E. **1-Phen** (500 mg, 1.08 mmol), MeOH (1.09 mL, 27.0 mmol, 25.0 equiv.) and HCl (3.70 M in Et_2O, 1.20 mL, 4.44 mmol, 4.10 equiv) in Et_2O (25 mL) and toluene (25 mL) at –78 °C, then over night at rt. ^{11}B NMR-spectroscopic analysis of the reaction mixture showed full conversion of **1-Phen** to ca. 85% **2-Phen** and 15% **4-Phen**. The solution was decanted and dried *in vacuo* bellow 0 °C. The residual solid was washed with cold hexane (2 x 10 mL) before drying it *in vacuo* to yield **4-Phen** as the sole isolable product (344 mg, 1.37 mmol, 63%).
Method F. Same reagent quantities as **method D**, in toluene at –78 °C, then 18 h at rt. ^{11}B NMR-spectroscopic analysis of the reaction mixture showed ca. 60% conversion of **1-Phen** to

ca. 30% **2-Phen** and 30% **4-Phen**.. After drying *in vacuo* the residual solid was extracted with toluene (2 x 25 mL). The toluene filtrate was dried *in vacuo* and the residual solid washed with hexane (2 x 15 mL) before drying *in vacuo* to yield **4-Phen** as the sole isolable product (168 mg, 670 μmol, 31%).

Method G. 1-Phen (150 mg, 320 μmol), MeOH (30.0 μL, 680 μmol, 2.10 equiv.) and HCl (3.70 M in Et_2O, 180 μL, 680 μmol, 2.10 equiv.) in Et_2O (30 mL) at –78 °C, then 3 h at rt. ^{11}B NMR-spectroscopic analysis of the reaction mixture showed full conversion of **1-Phen** to ca. 80% **2-Phen** and 20% **4-Phen**. The NMe_2H_2Cl by-product was quenched by adding NaH (19.3 mg, 810 μmol, 2.50 equiv.) and stirring for 1 h at rt. Extraction and isolation as in **method A**, yielding **4-Phen** as the sole isolable product (59.0 mg, 250 μmol, 38%).

Attempted synthesis of 2-Pyr and isolation of 4-Pyr.

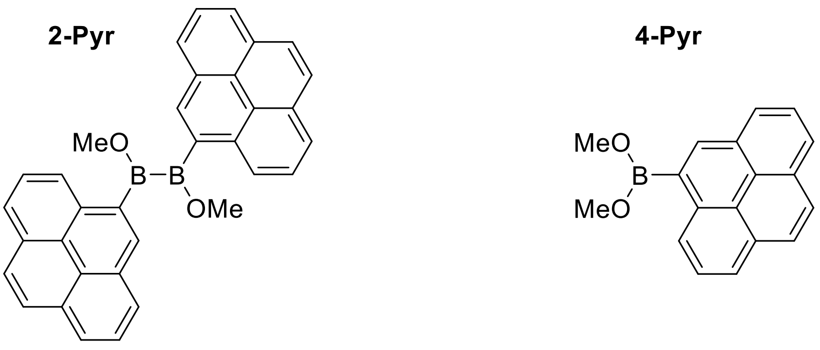

Method A. 1-Pyr (5.00 g, 9.76 mmol), MeOH (9.90 mL, 244 mmol, 25.0 equiv.) and HCl (3.70 M in Et_2O, 10.8 mL, 40.0 mmol, 4.10 equiv.) in Et_2O (400 mL) at –78 °C, then 18 h at rt. All volatiles were removed *in vacuo* and the residual solid extracted with benzene (3 x 100 mL). ^{11}B NMR-spectroscopic analysis of the reaction mixture showed full conversion of **1-Pyr** to ca. 70% **2-Pyr** (δ_{11B} = 58.7 ppm) and 30% **4-Pyr** (δ_{11B} = 31.4 ppm). All volatiles were removed *in vacuo* and the residual solid extracted with benzene (3 x 100 mL), prior to drying *in vacuo*. The residual solid washed with hexane (3 x 25 mL) and again dried *in vacuo* to yield **4-Pyr** as a pale yellow powder, the sole isolable product (4.02 g, 14.7 mmol, 75%). ^{11}B NMR (128.4 MHz, C_6D_6): δ = 30.4 (s) ppm.

Method B. 1-Pyr (500 mg, 980 μmol), MeOH (80.0 μL, 2.05 mmol, 2.10 equiv.) and HCl (1.50 M in toluene, 1.37 mL, 2.05 mmol, 2.10 equiv.) in toluene (50 mL) at –78 °C, then 4 h at rt. ^{11}B NMR-spectroscopic analysis of the reaction mixture showed full conversion of **1-Pyr** to ca. 20% **2-Pyr** and 80% **4-Pyr.** All volatiles were removed *in vacuo* and the residual solid extracted with toluene (3 x 20 mL). The toluene filtrate was dried *in vacuo* and the residual solid washed

with hexane (2 x 15 mL) before drying *in vacuo* to yield **4-Pyr** as the sole isolable product (210 mg, 760 µmol, 39%).

Attempted synthesis of 3-Phen with BBr_3

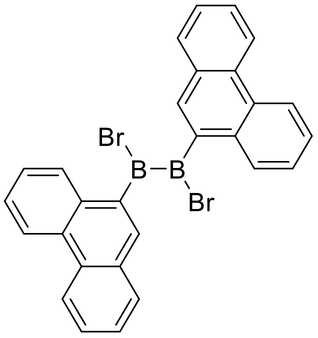

To a stirred suspension of **1-Phen** (100 mg, 0.220 mmol) in toluene (20 mL) at –78 °C BBr_3 (1.00 M, 0.470 mL, 0.470 mmol, 2.20 equiv.) was added dropwise and the reaction mixture was stirred at this temperature before being allowed to warm up to rt and stirred for another hour. All volatiles were removed *in vacuo* and the residual solid was washed with cold hexane (2 x 8 mL). The ^{11}B NMR spectrum of the crude product showed a complex mixture, including the expected $BBr_n(NMe_2)_{3-n}$ by-products, but no shift attributable to the desired diborane(4) **3-Phen**.

Synthesis of 5-Phen

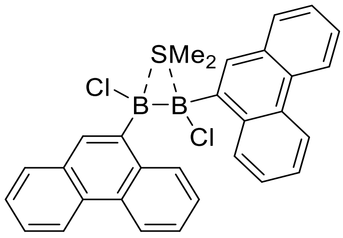

1-Phen (2.05 g, 4.42 mmol) and $Cl_3B{\cdot}SMe_2$ (1.66 g, 9.27 mmol, 2.10 equiv.) were suspended in benzene (150 mL) and the mixture stirred for 5 days at rt. After removal of volatiles *in vacuo* the residual solid was washed with benzene (3 x 10 mL) and hexane (3 x 20 mL). The remaining solid was dried *in vacuo* to yield **5-Phen** as a colourless powder (2.03 g, 3.98 mmol, 90%). ^{1}H NMR (500.1 MHz, C_6D_5Br): δ = 8.52 (d, $^{3}J_{HH}$ = 8.2 Hz, 2H, Ar-*H*), 8.49 (d, $^{3}J_{HH}$ = 8.1 Hz, 2H, Ar-*H*), 8.38 (d, $^{3}J_{HH}$ = 8.3 Hz, 2H, Ar-*H*), 8.26 (br s, 2H, Ar-*H*), 7.52 (ddd, $^{3}J_{HH}$ = 8.1 Hz, $^{3}J_{HH}$ = 6.8 Hz, $^{4}J_{HH}$ = 1.3 Hz, 3H, Ar-*H*), 7.47 (ddd, $^{3}J_{HH}$ = 8.1 Hz, $^{3}J_{HH}$ = 6.8 Hz, $^{4}J_{HH}$ = 1.3 Hz, 3H, Ar-*H*), 7.39 (ddd, $^{3}J_{HH}$ = 8.1 Hz, $^{3}J_{HH}$ = 6.8 Hz, $^{4}J_{HH}$ = 1.1 Hz, 2H, Ar-*H*), 7.25 (br t, $^{3}J_{HH}$ = 8.1 Hz, 2H, Ar-*H*), 1.74 (s, 6H, S(C*H*$_3$)$_2$) ppm. ^{11}B NMR (160.5 MHz, C_6D_5Br): δ = 12.8 (br) ppm. $^{13}C\{^{1}H\}$ NMR (125.8 MHz, C_6D_5Br): δ = 139.4 (B*C*), 133.5 (Ar-*C*H), 133.2 (Ar-*C*$_q$), 130.3(Ar-

*C*q), 129.3 (Ar-*C*H), 129.2 (Ar-*C*H), 128.2 (Ar-*C*q), 127.2 (Ar-*C*H), 126.4 (Ar-*C*H), 126.3 (Ar-*C*H), 126.2 (Ar-*C*H), 123.1 (Ar-*C*H), 122.2 (Ar-*C*H), 19.6 (S(CH_3)2) ppm. *Note: Multiple attempts at obtaining HRMS or elemental analysis data failed due to the high lability of the dimethylsulfide ligand. This lability is also visible in the NMR spectra, which show symmetrical aryl resonances and a single SMe_2 ligand, indicating rapidly fluctuating SMe_2 exchange between the two boron atoms.*

Synthesis 5-Pyr

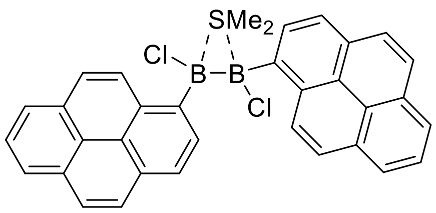

1-Pyr (2.50 g, 4.88 mmol) and $Cl_3B{\cdot}SMe_2$ (1.84 g, 10.3 mmol, 2.10 equiv.) were suspended in benzene (150 mL) and stirred for 5 days at rt. After removal of volatiles *in vacuo* the residual solid was washed with benzene (3 x 10 mL) and hexane (3 x 20 mL). The remaining solid was dried *in vacuo* to yield **5-Pyr** as a pale yellow powder (2.29 g, 4.11 mmol, 84%). ^{1}H NMR (500.1 MHz, C_6D_5Br): δ = 8.76 (d, $^3J_{HH}$ = 9.2 Hz, 2H, Ar-*H*), 8.72 (d, $^3J_{HH}$ = 7.8 Hz, 2H, Ar-*H*), 7.96 (d, $^3J_{HH}$ = 7.0 Hz, 2H, Ar-*H*), 7.92 (d, $^3J_{HH}$ = 7.8 Hz, 4H, Ar-*H*), 7.87 (d, $^3J_{HH}$ = 9.8 Hz, 4H, Ar-*H*), 7.80 (d, $^3J_{HH}$ = 9.5 Hz, 2H, Ar-*H*), 7.78 (t, $^3J_{HH}$ = 7.6 Hz, 2H, Ar-*H*), 1.77 (s, 6H, S(CH_3)2) ppm. *Note: the ^{11}B NMR resonances were not detectable due to line broadening and the baseline of the boron-containing NMR probehead.* ^{13}C{^{1}H} NMR (125.8 MHz, C_6D_5Br): δ = 136.5 (B*C*), 134.3 (Ar-*C*q), 133.3 (Ar-*C*q), 131.0 (Ar-*C*H), 128.7 (Ar-*C*H), 128.2 (Ar-*C*q), 127.9 (Ar-*C*H), 127.6 (Ar-*C*H), 127.4 (Ar-*C*H), 125.8 (Ar-*C*H), 125.7 (Ar-*C*q), 125.6 (Ar-*C*H), 125.6 (Ar-*C*H), 124.7 (Ar-*C*q), 124.4 (Ar-*C*H) 19.3 (S(CH_3)2) ppm. *Note: Multiple attempts at obtaining HRMS or elemental analysis data failed due to the high lability of the dimethylsulfide ligand. This lability is also visible in the NMR spectra, which show symmetrical aryl resonances and a single SMe_2 ligand, indicating rapidly fluctuating SMe_2 exchange between the two boron atoms.*

Synthesis of 6-Phen

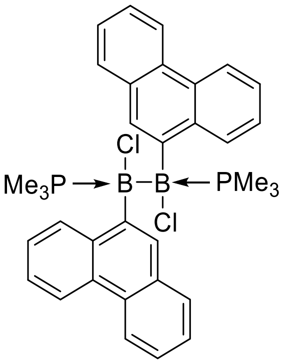

5-Phen (1.33 g, 2.61 mmol) was suspended in benzene (30 mL) and PMe_3 (660 µL, 495 mg, 6.51 mmol, 2.50 equiv.) was added dropwise. The reaction mixture was stirred for 16 h at rt. After removal of volatiles *in vacuo* the residual solid was washed with benzene (2 x 10 ml) and hexane (3 x 10 mL). The residual solid was dried *in vacuo* to yield **6-Phen** as a colourless powder (1.30 g, 2.17 mmol, 83%). Colourless single crystals of **6-Phen** suitable for X-ray crystallographic analysis were obtained from slow evaporation of a saturated benzene solution. ^{1}H NMR (500.1 MHz, $C_2D_2Cl_2$): δ = 8.41 (d, $^3J_{HP}$ = 4.7 Hz, 2H, Ar-*H*), 8.30 (d, $^3J_{HH}$ = 8.2 Hz, 2H, Ar-*H*), 8.27 (d, $^3J_{HH}$ = 8.2 Hz, 2H, Ar-*H*), 8.14 (d, $^3J_{HH}$ = 8.1 Hz, 2H, Ar-*H*), 7.84 (d, $^3J_{HH}$ = 7.9 Hz, 2H, Ar-*H*), 7.45 (ddd, $^3J_{HH}$ = 8.2 Hz, $^3J_{HH}$ = 6.8 Hz, $^4J_{HH}$ = 1.3 Hz, 2H, Ar-*H*), 7.37 (ddd, $^3J_{HH}$ = 8.2 Hz, $^3J_{HH}$ = 6.8 Hz, $^4J_{HH}$ = 1.3 Hz, 2H, Ar-*H*), 6.99 (ddd, $^3J_{HH}$ = 8.2 Hz, $^3J_{HH}$ = 6.8 Hz, $^4J_{HH}$ = 1.3 Hz, 2H, Ar-*H*), 6.89 (ddd, $^3J_{HH}$ = 8.2 Hz, $^3J_{HH}$ = 6.8 Hz, $^4J_{HH}$ = 1.3 Hz, 2H, Ar-*H*), 1.37 (d, $^2J_{PH}$ = 11.0 Hz, 18H, P(C*H*$_3$)$_3$) ppm. ^{11}B NMR (160.5 MHz, $C_2D_2Cl_2$): δ = 1.3 (s, br) ppm. ^{13}C{^{1}H} NMR (125.8 MHz, $C_2D_2Cl_2$): δ = 144.95 (B*C*), 134.64 (Ar-*C*$_q$), 133.38 (Ar-*C*H), 133.27 (Ar-*C*H), 132.73 (Ar-*C*$_q$), 129.60 (Ar-*C*$_q$), 129.43 (Ar-*C*$_q$), 128.59 (Ar-*C*H), 125.97 (Ar-*C*H), 125.29 (Ar-*C*H), 124.83 (Ar-*C*H), 123.61 (Ar-*C*H), 122.06 (Ar-*C*H), 121.40 (Ar-*C*H), 11.90 (d, $^1J_{PC}$ = 38.5 Hz, P(*C*H$_3$)$_3$) ppm. ^{31}P{^{1}H} NMR (202.5 MHz, $C_2D_2Cl_2$): δ = –8.65 (s, *P*Me$_3$) ppm. HRMS ESI pos-MS (*m/z*) calculated for [$C_{34}H_{36}B_2Cl_2P_2$]: 598.1850; found: 598.1837.

Synthesis of 6-Pyr

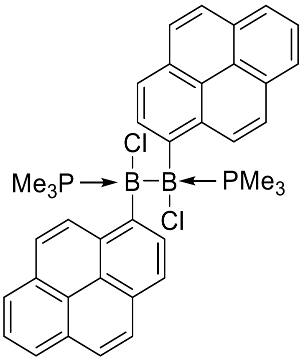

5-Pyr (1.50 g, 2.69 mmol) was suspended in benzene (30 mL) and PMe_3 (0.685 µL, 512 mg, 6.73 mmol, 2.50 equiv.) was added dropwise. The reaction mixture was stirred for 16 h at rt. After removal of volatiles *in vacuo* the residual solid was washed with benzene (2x 10 ml) and hexane (3x 10 mL). The residual solid was dried *in vacuo* to yield **6-Pyr** as a pale yellow powder (1.49 g, 2.30 mmol, 86%). Yellow single crystals of **6-Pyr** suitable for X-ray crystallographic analysis were obtained from slow diffusion of hexane into a saturated THF solution. ^{1}H NMR (500.1 MHz, C_6D_5Br): δ = 9.24 (dd, $^{3}J_{HH}$ = 8.0 Hz, $^{3}J_{HP}$ = 3.0 Hz, 2H, Ar-*H*), 8.70 (d, $^{3}J_{HH}$ = 9.1 Hz, 2H, Ar-*H*), 8.07 (dd, $^{3}J_{HH}$ = 8.0 Hz, $^{4}J_{HH}$ = 1.4 Hz, 2H, Ar-*H*), 7.63 (d, $^{3}J_{HH}$ = 5.9 Hz, 2H, Ar-*H*), 7.62 (d, $^{3}J_{HH}$ = 5.9 Hz, 2H, Ar-*H*), 7.58 (d, $^{3}J_{HH}$ = 8.8 Hz, 2H, Ar-*H*), 7.53 (d, $^{3}J_{HH}$ = 9.1 Hz, 2H, Ar-*H*), 7.46-7.44 (m, 4H, Ar-*H*), 7.33 (dd, $^{3}J_{HH}$ = 8.8 Hz, $^{4}J_{HH}$ = 1.4 Hz, 2H, Ar-*H*), 1.09 (d, $^{2}J_{PH}$ = 10.9 Hz, 18H, P(C*H*$_3$)$_3$) ppm. *Note: the ^{11}B NMR resonances were not detectable due to line broadening and the baseline of the boron-containing NMR probehead.* ^{13}C{^{1}H} NMR (125.8 MHz, C_6D_5Br): δ = 144.4 (B*C*), 132.66 (Ar-*C*H), 132.62 (Ar-*C*$_q$), 132.58 (Ar-*C*$_q$), 131.66 (Ar-*C*H), 128.15 (Ar-*C*$_q$), 127.10 (Ar-*C*H), 125.70 (Ar-*C*H), 124.62 (Ar-*C*$_q$), 124.55 (Ar-*C*H), 124.30 (Ar-*C*$_q$), 124.05(Ar-*C*H), 132.32 (2C, *C*), 123.22 (Ar-*C*H), 10.84 (d, $^{1}J_{PC}$ = 37.6 Hz, P(*C*H$_3$)$_3$) ppm. *Note: the two B*C *^{13}C NMR resonances could not be detected due to strong broadening owing to coupling to the quadrupolar boron nuclei.* ^{31}P{^{1}H} NMR (202.5 MHz, C_6D_5Br): δ = –7.34 (s, *P*Me$_3$) ppm. HRMS ESI pos-MS (*m/z*) calculated for [$C_{38}H_{36}B_2Cl_2P_2$] = [M]: 646.1850; found: 646.1835.

Synthesis of 7-Phen

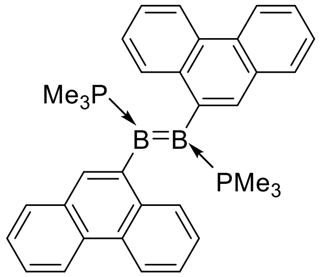

6-Phen (50.0 mg, 83.5 µmol) was suspended in a mixture of benzene (5 mL) and THF (1 mL) and KC_8 (28.2 mg, 209 µmol, 2.50 equiv.) was added. The reaction mixture was stirred for 4h at rt before the suspension was filtrated. The resulting red solution was dried *in vacuo* to yield **7-Phen** as a red powder (32.0 mg, 60.6 µmol, 72%). Red single crystals of **7-Phen** suitable for X-ray crystallographic analysis were obtained from slow evaporation of a saturated benzene solution. ^{1}H NMR data showed the presence of two rotamers in a 55:45 ratio. **Rotamer 1** (ca 55%): ^{1}H NMR (500.1 MHz, C_6D_6): δ = 9.54 (dd, $^3J_{HH}$ = 8.1 Hz, $^4J_{HH}$ = 1.3 Hz, 1H, Ar-*H*), 8.75 (d, $^3J_{HH}$ = 8.2 Hz, 2H, Ar-*H*), 8.33 (t, $^3J_{HH}$ = $^3J_{HP}$ = 2.1 Hz, 1H, Ar-*H*), 7.98 (dm, $^3J_{HH}$ = 7.8 Hz, 1H, Ar-*H*), 7.72 (ddd, $^3J_{HH}$ = 6.8, 8.1 Hz, $^4J_{HH}$ = 1.3 Hz, 1H, Ar-*H*), 7.58 (dt, $^3J_{HH}$ = 6.8 Hz, $^4J_{HH}$ = 1.4 Hz, 1H, Ar-*H*), 7.53 (dt, $^3J_{HH}$ = 7.4 Hz, $^4J_{HH}$ = 1.3 Hz, 1H, Ar-*H*), 7.46 (m, 1H, Ar-*H*), 0.58 (vt, *J* = 5.1 Hz, 9H, P(CH_3)$_3$) ppm. **Rotamer 2** (ca 45%): ^{1}H NMR (500.1 MHz, C_6D_6): δ = 9.58 (dd, $^3J_{HH}$ = 8.1 Hz, $^4J_{HH}$ = 1.3 Hz, 1H, Ar-*H*), 8.70 (d, $^3J_{HH}$ = 8.2 Hz, 2H, Ar-*H*), 8.29 (t, $^3J_{HH}$ = $^3J_{HP}$ = 2.1 Hz, 1H, Ar-*H*), 7.97 (dm, $^3J_{HH}$ = 7.8 Hz, 1H, Ar-*H*), 7.70 (ddd, $^3J_{HH}$ = 6.8, 8.1 Hz, $^4J_{HH}$ = 1.3 Hz, 1H, Ar-*H*), 7.58 (dt, $^3J_{HH}$ = 6.8 Hz, $^4J_{HH}$ = 1.4 Hz, 1H, Ar-*H*), 7.52 (dt, $^3J_{HH}$ = 7.4 Hz, $^4J_{HH}$ = 1.3 Hz, 1H, Ar-*H*), 7.46 (dm, $^3J_{HH}$ = 6.8 Hz, 1H, Ar-*H*), 0.57 (vt, *J* = 5.1 Hz, 9H, P(CH_3)$_3$) ppm. ^{11}B NMR (160.5 MHz, C_6D_6): δ = 21.2 (s, br). ^{13}C{^{1}H} NMR (125.8 MHz, C_6D_6): δ = 141.5 (B*C*), 139.2 (Ar-C_q), 133.9 (Ar-*C*H), 133.4 (Ar-*C*H), 133.2 (Ar-C_q), 130.7 (Ar-C_q), 130.2 (Ar-C_q), 128.1 (Ar-*C*H), 126.6 (Ar-*C*H), 125.6 (Ar-*C*H), 125.3 (Ar-*C*H), 124.9 (Ar-*C*H), 123.2 (2C, Ar-*C*H), 14.0 (vt, *J* = 18.7 Hz, P(*C*H_3)$_3$) ppm. ^{31}P{^{1}H} NMR (202.5 MHz, C_6D_6): δ = –21.6 (s, *P*Me$_3$) ppm. HRMS ESI pos-MS (*m/z*) calculated for [$C_{34}H_{36}B_2P_2$] = [M]: 528.2473; found: 528.2453.

Synthesis of 7-Pyr

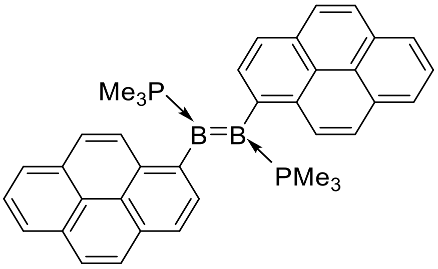

6-Pyr (50.0 mg, 77.3 µmol) was suspended in a mixture of benzene (5 mL) and THF (2.5 ml) and KC_8 (26.1 mg, 19.3 µmol, 2.50 equiv.) was added. The reaction mixture was stirred for 30 min at rt before the suspension was filtrated. The resulting blue solution was dried *in vacuo* to yield crude **7-Pyr** as a blue powder (25.0 mg). Multiple attempts to obtain analytically pure **9-Pyr** by recrystallisation failed due to the systematic co-crystallisation of side-products. ^{11}B NMR (128.4 MHz, C_6H_6 / thf): $\delta = 21.1$ (s, br). $^{31}P\{^1H\}$ NMR (162.1 MHz, C_6H_6 / thf)): $\delta = -21.8$ (s, *P*Me_3) ppm. HRMS ESI pos-MS (*m/z*) calculated for [$C_{35}H_{27}B_2P$] = [M–PMe_3]: 500.2031; found: 500.2013.

Synthesis of μ-(1,10-Phen)B₂PhenH(PMe₃)₂, 8-Phen

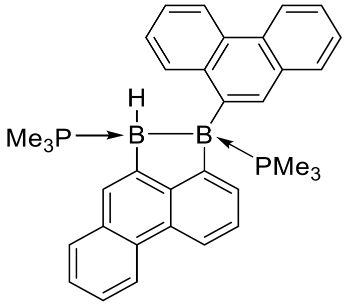

7-Phen (20.0 mg, 37.9 µmol) was suspended in 0.6 mL toluene in a J.-Young NMR-tube and was heated for 2 d at 120 °C. After removal of volatiles *in vacuo* the residual solid was washed with a mixture of benzene and hexane (1:1, 3 x 0.3 mL). The residual solid was dried *in vacuo* to yield **8-Phen** as a colourless powder (10.0 mg, 18.9 µmol, 50%). Colourless single crystals of **8-Phen** suitable for X-ray crystallographic analysis were obtained from slow diffusion of hexane into a saturated benzene solution. $^1H\{^{11}B\}$ NMR (500.1 MHz, C_6D_6): $\delta = 9.16$-9.14 (m, 1H, Ar-*H*), 9.08 (d, $^3J_{HP} = 3.9$ Hz, 1H, Ar-*H*), 8.81 (d, $^3J_{HH} = 7.9$ Hz, 1H, Ar-*H*), 8.75-8.73 (m, 1H, Ar-*H*), 8.65 (d, $^3J_{HH} = 7.8$ Hz, 1H, Ar-*H*), 8.65 (d, $^3J_{HH} = 7.6$ Hz, 1H, Ar-*H*), 8.37 (dd, $^3J_{HH} = 6.8$ Hz, $^3J_{HP} = 4.6$ Hz, 1H, Ar-*H*), 7.99 (d, $^3J_{HH} = 6.1$ Hz, 1H, Ar-*H*), 7.97 (d, $^3J_{HH} = 7.7$ Hz, 1H, Ar-*H*), 7.94 (d, $^3J_{HH} = 7.2$ Hz, 1H, Ar-*H*), 7.85 (t, $^3J_{HH} = 7.5$ Hz, 1H, Ar-*H*), 7.59-7.43 (m, 6H, Ar-*H*), 2.85 (t, 1H, B*H*, $J_{HP} = 17.0$ Hz), 0.34 (d, $^2J_{PH} = 9.9$ Hz, 9H, P(C*H*₃)₃), 0.24 (d, $^2J_{PH} = 10.1$ Hz, 9H, P(C*H*₃)₃) ppm. ^{11}B NMR (160.5 MHz, C_6D_6): $\delta = -13.4$ (br, *B*Phen), −22.2 (br,

*B*H) ppm. $^{13}C\{^1H\}$ NMR (125.8 MHz, C_6D_6): δ = 160.2 (B*C*), 157.6 (B*C*), 151.2 (B*C*), 138.5 (d, J_{PC} = 2.8 Hz, Ar-C_q), 134.7 (d, $^3J_{PC}$ = 5.8 Hz, Ar-C_q), 133.7 (d, J_{PC} = 1.0 Hz, Ar-*C*H), 132.3 (d, $^3J_{PC}$ = 7.2 Hz, Ar-*C*H), 132.0 (d, $^3J_{PC}$ = 10.9 Hz, Ar-*C*H), 131.1 (d, J_{PC} = 2.3 Hz, Ar-C_q), 130.6 (dd, J_{PC} = 3.6 Hz, 1.1 Hz, Ar-C_q), 129.7 (d, J_{PC} = 2.4 Hz, Ar-C_q), 129.3 (Ar-C_q), 128.6 (Ar-C_q), 128.3 (Ar-*C*H), 127.9 (Ar-*C*H), 127.5 (Ar-C_q), 126.8 (d, J_{PC} = 1.0 Hz, Ar-*C*H), 126.5 (d, J_{PC} = 5.3 Hz, Ar-*C*H), 126.1 (d, J_{PC} = 2.0 Hz, Ar-*C*H), 125.8 (Ar-*C*H), 125.2 (d, J_{PC} = 1.4 Hz, Ar-*C*H), 124.4 (Ar-*C*H), 124.3 (d, J_{PC} = 2.6 Hz, Ar-*C*H), 123.3 (Ar-*C*H), 123.2 (Ar-*C*H), 122.7 (2C, Ar-*C*H), 119.6 (d, $^3J_{PC}$ = 5.7 Hz, Ar-*C*H), 11.2 (d, $^1J_{PC}$ = 31.8 Hz, P(CH_3)$_3$), 10.3 (d, $^1J_{PC}$ = 34.2 Hz, P(CH_3)$_3$) ppm. $^{31}P\{^1H\}$ NMR (202.5 MHz, C_6D_6): δ = –4.91 (d, $^3J_{PP}$ = 84.5 Hz), –9.09 (d, $^3J_{PP}$ = 84.5 Hz) ppm. HRMS ESI pos-MS (*m/z*) calculated for [$C_{31}H_{27}B_2P_1$] = [M–PMe_3]: 452.2031; found: 452.2017.

Synthesis of *μ*-(1,10-Pyr)B_2PyrH(PMe_3)$_2$, 8-Pyr

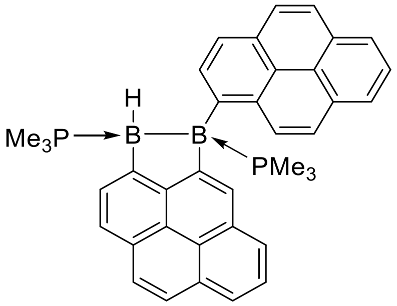

7-Pyr (20.0 mg, 37.9 μmol) was suspended in 0.6 mL toluene in a J.-Young NMR-tube and was heated for 1d at 120 °C. After removal of volatiles *in vacuo* the residual solid was washed with a mixture of benzene and hexane (1:1, 3 x 0.3 mL). The residual solid was dried *in vacuo* to yield **8-Pyr** as a yellow powder (11.0 mg, 20.8 μmol, 55%). ^1H NMR (500.1 MHz, C_6D_6): δ = 9.71 (dd, $^3J_{HH}$ = 7.7 Hz, $^4J_{HH}$ = 2.5 Hz, 1H, Ar-*H*), 9.35 (d, $^3J_{HH}$ = 9.3 Hz, 1H, Ar-*H*), 8.86 (d, $^3J_{HH}$ = 8.9 Hz, 1H, Ar-*H*), 8.32-8.28 (m, 3H, Ar-*H*), 8.23 (d, $^3J_{HH}$ = 9.0 Hz, 2H, Ar-*H*), 8.13 (d, $^3J_{HH}$ = 8.9 Hz, 1H, Ar-*H*), 8.09 (d, $^3J_{HH}$ = 7.6 Hz, 2H, Ar-*H*), 8.05-7.98 (m, 4H, Ar-*H*), 7.88 (d, $^3J_{HH}$ = 8.7 Hz, 1H, Ar-*H*), 7.84 (d, $^3J_{HH}$ = 6.2 Hz, 1H, Ar-*H*), 3.60-2.70 (br s, 1H, B*H*), 0.35 (d, $^2J_{PH}$ = 9.8 Hz, 9H, P(CH_3)$_3$), 0.17 (d, $^2J_{PH}$ = 10.1 Hz, 9H, P(CH_3)$_3$) ppm. ^{11}B NMR (160.5 MHz, C_6D_6): δ = −12.9 (br, *B*Pyr), −20.5 (br, *B*H) ppm. $^{13}C\{^1H\}$ NMR (125.8 MHz, C_6D_6): δ = 156.6 (B*C*), 151.9 (B*C*), 149.5 (B*C*), 135.4 (Ar-C_q), 134.2 (Ar-C_q), 133.6 (Ar-*C*H), 133.3 (Ar-*C*H), 132.6 (Ar-C_q), 132.4 (Ar-C_q), 132.2 (Ar-C_q), 131.1 (Ar-*C*H), 130.5 (Ar-*C*H), 129.4-129.3 (Ar-C_q), 129.0 (Ar-C_q), 128.6 (Ar-*C*H), 127.9 (Ar-*C*H), 126.7 (Ar-C_q), 126.4 (Ar-C_q), 126.0 (Ar-*C*H), 125.9 (Ar-*C*H), 125.7 (Ar-*C*H), 125.2 (Ar-*C*H), 124.3 (2C, Ar-*C*H), 124.1 (Ar-*C*H), 124.0 (2C, Ar-*C*H), 123.2 (Ar-*C*H), 122.3 (Ar-*C*H), 10.8 (d, $^1J_{PC}$ = 32.5 Hz, P(CH_3)$_3$), 10.3 (d, $^1J_{PC}$ =

33.9 Hz, P(CH_3)$_3$) ppm. *Note: the probe was too dilute to accurately measure J_{CP} coupling constants.* $^{31}P\{^1H\}$ NMR (202.5 MHz, C_6D_6): δ = –5.05 (d, $^3J_{PP}$ = 84.0 Hz), –8.43 (d, $^3J_{PP}$ = 84.0 Hz) ppm. HRMS ESI pos-MS (*m/z*) calculated for [$C_{35}H_{27}B_2P_1$] = [M–PMe_3]: 500.2031; found: 500.2014.

Synthesis of 8-Anth

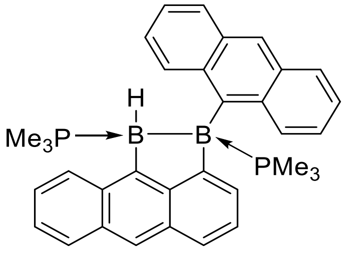

Diborene **III** (20 mg, 38 μmol) was suspended in 0.6 mL toluene in a J.-Young NMR tube and the mixture heated for 10 d at 125 °C. After removal of volatiles *in vacuo* the residual solid was washed with a mixture of benzene and hexane (1:1, 3 x 0.3 mL) and dried *in vacuo* to yield **8-Anth** as a yellow powder (8.0 mg). Multiple attempts to obtain analytically pure **8-Anth** by recrystallisation failed due to the systematic co-crystallisation of side-products. ^{11}B NMR (160.5 MHz, C_6D_6): δ = −13.4 (*B*Anth), −22.2 (*B*H) ppm. $^{31}P\{^1H\}$ NMR (162.1 MHz, C_6D_6): –6.5 (br), –13.4 (br) ppm. HRMS ESI pos-MS (*m/z*) calculated for [$C_{31}H_{27}B_2P_1$] = [M–PMe_3]: 452.2031; found: 452.2017.

Synthesis of 10-Mes

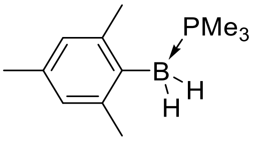

50 mg of diborene **7-Mes** (0.12 mmol) were dissolved in 0.6 mL benzene in a high pressure-Young NMR-tube and frozen using liquid nitrogen. The inert gas atmosphere was exchanged with 5 bar H_2 or D_2 and the yellow solution heated for 9 days at 80 °C. After removal of all volatiles under reduced pressure, the crude products of four identical batches were sublimed at 60 °C and at 10^{-3} bar to yield colourless crystals of **10-Mes** (86 mg, 41 μmol, 85% yield) or **10-Mes-D$_2$** (82 mg, 39 μmol, 80% yield). $^1H\{^{11}B\}$ NMR (500.1 MHz, C_6D_6): δ= 7.04 (s, 2H, *m*-Mes-*CH*), 2.59 (d, $^5J_{PH}$ = 1.7 Hz, 6H, *o*-Mes-CH_3), 2.46 (d, $^1J_{BH}$ = 18.5 Hz, 2H, BH_2, **10-Mes**), 2.33 (d, $^7J_{PH}$ = 3.2 Hz, 3H, *p*-Mes-CH_3), 0.54 (d, $^2J_{PH}$= 10.1 Hz, 9H, PCH_3) ppm. $^2H\{^{11}B\}$ NMR (76.8 MHz, C_6D_6): δ = 2.44 (d, $^1J_{BD}$ = 2.7 Hz, BD_2, **10-Mes-D$_2$**) ppm. $^{13}C\{^1H\}$ NMR (125.8 MHz, C_6D_6): δ = 142.8 (d, $^3J_{PC}$ = 6.8 Hz, *o*-Mes-*C*), 140.0 (br, *i*-Mes-*C*), 133.8 (d, $^5J_{PC}$

= 5.2 Hz, *p*-Mes-*C*), 128.3–128.4 (m, *m*-Mes-*C*H), 25.0–25.1 (m, *o*-Mes-*C*H_3), 21.3 (d, $^{6}J_{PC}$ = 2.0 Hz, *p*-Mes-*C*H_3), 11.2 (d, $^{1}J_{PC}$ = 34.3 Hz, P*C*H_3) ppm. $^{11}B\{^{1}H\}$ NMR (160.5 MHz, C_6D_6): δ = −28.3 (d, $^{1}J_{PB}$ = 50 Hz) ppm. $^{31}P\{^{1}H\}$ NMR (202.5 MHz, C_6D_6): δ = −7.1 (br m, fwmh ≈ 158 Hz) ppm. Solid-state FT-IR: ν = 2309 (B–H), 2353 (B–H) cm^{-1}. Elemental analysis calculated for [$C_{12}H_{22}BP$] (M_W = 208.09): C 69.26, H 10.66%; found: C 69.27, H 10.65%.

Synthesis of 10-Anth

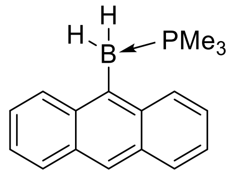

7-Anth (25.0 mg, 47.3 µmol) was suspended in 0.6 mL toluene and frozen using liquid nitrogen. The argon atmosphere was exchanged with 1 bar H_2 and the reaction mixture was heated for 5 days at 135 °C. After removal of all volatiles under reduced pressure the residual solid was washed with a mixture of benzene and hexane (1:3, 3 x 0.3 mL). The residual solid was dried *in vacuo* to yield **10-Anth** as a pale yellow powder (16.0 mg) in ca. 70% purity as determined by NMR spectroscopy. Multiple attempts to obtain analytically pure **10-Anth** by recrystallisation failed due to the systematic co-crystallisation of side-products. Yellow single crystals of **10-Anth** suitable for X-ray crystallographic analysis were obtained from slow evaporation of a saturated benzene solution. ^{1}H NMR (400.1 MHz, C_6D_6): δ = 9.10 (d, $^{3}J_{HH}$ = 8.7 Hz, 2H, Ar-*H*), 8.29 (d, $^{4}J_{HH}$ = 4.1 Hz, 1H, Ar-*H*), 8.00 (d, $^{3}J_{HH}$ = 7.9 Hz, 2H, Ar-*H*), 7.46-7.39 (m, 4H, Ar-*H*), 3.59-2.90 (br m, 2H, Ar-B*H*$_2$), 0.41 (d, $^{2}J_{PH}$ = 10.4 Hz, 9H, P(C*H*$_3$)$_3$) ppm. ^{11}B NMR (128.4) MHz, C_6D_6): δ = −28.8 (d, $^{1}J_{PB}$ = 50.1 Hz) ppm. $^{13}C\{^{1}H\}$ NMR (100.8 MHz, C_6D_6): δ = 137.2 (d, $^{3}J_{PC}$ = 7.3 Hz, Ar-C_q), 132.6 (d, $^{4}J_{PC}$ = 4.8 Hz, Ar-C_q), 131.3 (d, $^{5}J_{PC}$ = 3.1 Hz, Ar-*C*H), 129.6 (d, $^{6}J_{PC}$ = 1.8 Hz, Ar-*C*H), 125.5 (d, $^{4}J_{PC}$ = 6.5 Hz, Ar-*C*H), 124.5 (d, $^{5}J_{PC}$ = 1.9 Hz, Ar-*C*H), 123.5 (d, $^{5}J_{PC}$ = 2.6 Hz, Ar-*C*H), 10.9 (d, $^{1}J_{PC}$ = 35.3 Hz, P*C*H_3) ppm. *Note: The B*C *^{13}C NMR resonance could not be detected due to strong broadening owing to coupling to the quadrupolar boron nuclei.* $^{31}P\{^{1}H\}$ NMR (162.1 MHz, C_6D_6): δ = –6.1 (br, m) ppm. HRMS ESI pos-MS (*m/z*) calculated for [$C_{17}H_{20}B_1P_1$] = [M]: 266.1390; found: 266.1382.

Synthesis of 10-Phen

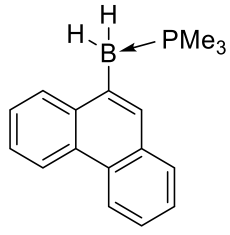

7-Phen (20.0 mg, 37.8 µmol) was suspended in 0.6 mL toluene and frozen using liquid nitrogen. The inert gas atmosphere was exchanged with 1 bar H_2 and the reaction mixture was heated for 5 days at 125 °C. After removal of all volatiles under reduced pressure, the residual solid was washed with a mixture of benzene and hexane (1:3, 3 x 0.3 mL). The residual solid was dried *in vacuo* to yield **10-Phen** as a colourless powder (14.0 mg) in ca. 80% purity as determined by NMR spectroscopy. Multiple attempts to obtain analytically pure **10-Phen** by recrystallisation failed due to the systematic co-crystallisation of side-products. ^{1}H NMR (500.1 MHz, C_6D_6): δ = 8.76 (d, $^3J_{HH}$ = 7.9 Hz, 1H, Ar-*H*), 8.72 (d, $^3J_{HH}$ = 8.1 Hz, 1H, Ar-*H*), 8.64 (d, $^3J_{HH}$ = 7.9 Hz, 1H, Ar-*H*), 8.17 (d, $^4J_{HH}$ = 4.8 Hz, 1H, Ar-*H*), 7.88 (d, $^3J_{HH}$ = 7.5 Hz, 1H, Ar-*H*), 7.61-7.53 (m, 2H, Ar-*H*), 7.49-7.44 (m, 2H, Ar-*H*), 2.85 d, $^2J_{PH}$ = 17.6 Hz, 2H, Ar-B*H*$_2$), 0.47 (d, $^2J_{PH}$ = 10.4 Hz, 9H, P(C*H*$_3$)$_3$) ppm. ^{11}B{^{1}H} NMR (160.5 MHz, C_6D_6): δ = −24.8 (d, $^1J_{PB}$ = 50 Hz) ppm. ^{13}C{^{1}H} NMR (125.8 MHz, C_6D_6): δ = 144.0 (B*C*), 138.1 (d, $^4J_{PC}$ = 3.9 Hz, Ar-*C*$_q$), 135.5 (d, $^3J_{PC}$ = 10.5 Hz, Ar-*C*H), 133.4 (d, $^3J_{PC}$ = 4.3 Hz, Ar-*C*$_q$), 130.9 (d, $^5J_{PC}$ = 2.4 Hz, Ar-*C*$_q$), 130.6 (d, $^4J_{PC}$ = 3.1 Hz, Ar-*C*$_q$), 130.5 (d, $^6J_{PC}$ = 1.3 Hz, Ar-*C*H), 128.1 (d, $^4J_{PC}$ = 2.3 Hz, Ar-*C*H), 126.4 (d, $^5J_{PC}$ = 1.8 Hz, Ar-*C*H), 125.7 (d, $^7J_{PC}$ = 0.6 Hz Ar-*C*H), 125.6 (d, $^6J_{PC}$ = 0.7 Hz ,Ar-*C*H), 125.3 (d, $^5J_{PC}$ = 2.2 Hz Ar-*C*H), 123.3 (d, $^6J_{PC}$ = 0.7 Hz, Ar-*C*H), 122.9 (d, $^5J_{PC}$ = 2.1 Hz, Ar-*C*H), 10.4 (d, $^1J_{PC}$ = 36.0 Hz, P(*C*H$_3$)$_3$). ^{31}P{^{1}H} NMR (202.5 MHz, C_6D_6): δ = –8.1 (br, m) ppm. HRMS ESI pos-MS (*m/z*) calculated for [$C_{17}H_{20}B_1P_1$] = [M]: 266.1390; found: 266.1380.

Synthesis of 10-Pyr

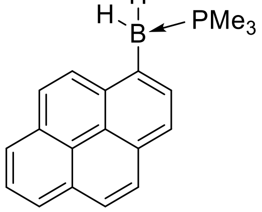

7-Pyr (20.0 mg, 34.7 µmol) was suspended in 0.6 mL toluene and frozen using liquid nitrogen. The inert gas atmosphere was exchanged with 1 bar H_2 and the reaction mixture was heated for 5 days at 125 °C. After removal of all volatiles under reduced pressure, the residual solid was washed with a mixture of benzene and hexane (1:3, 3 x 0.3 mL). The residual solid was dried

in vacuo to yield crude **10-Pyr** as a yellow powder (11.0 mg). Multiple attempts to obtain analytically pure **10-Pyr** by recrystallisation failed due to its low solubility and the systematic co-crystallisation of side-products. $^{11}B\{^1H\}$ NMR (160.5 MHz, C_6D_6): δ = −24.9 (d, $^1J_{PB}$ = 52 Hz) ppm. $^{31}P\{^1H\}$ NMR (202.5 MHz, C_6D_6): δ = – 7.4 (br, m) ppm. HRMS ESI pos-MS (*m/z*) calculated for $[C_{17}H_{20}B_1P_1]$ = [M]: 290.1390; found: 290.1376.

NMR spectra of isolated compounds

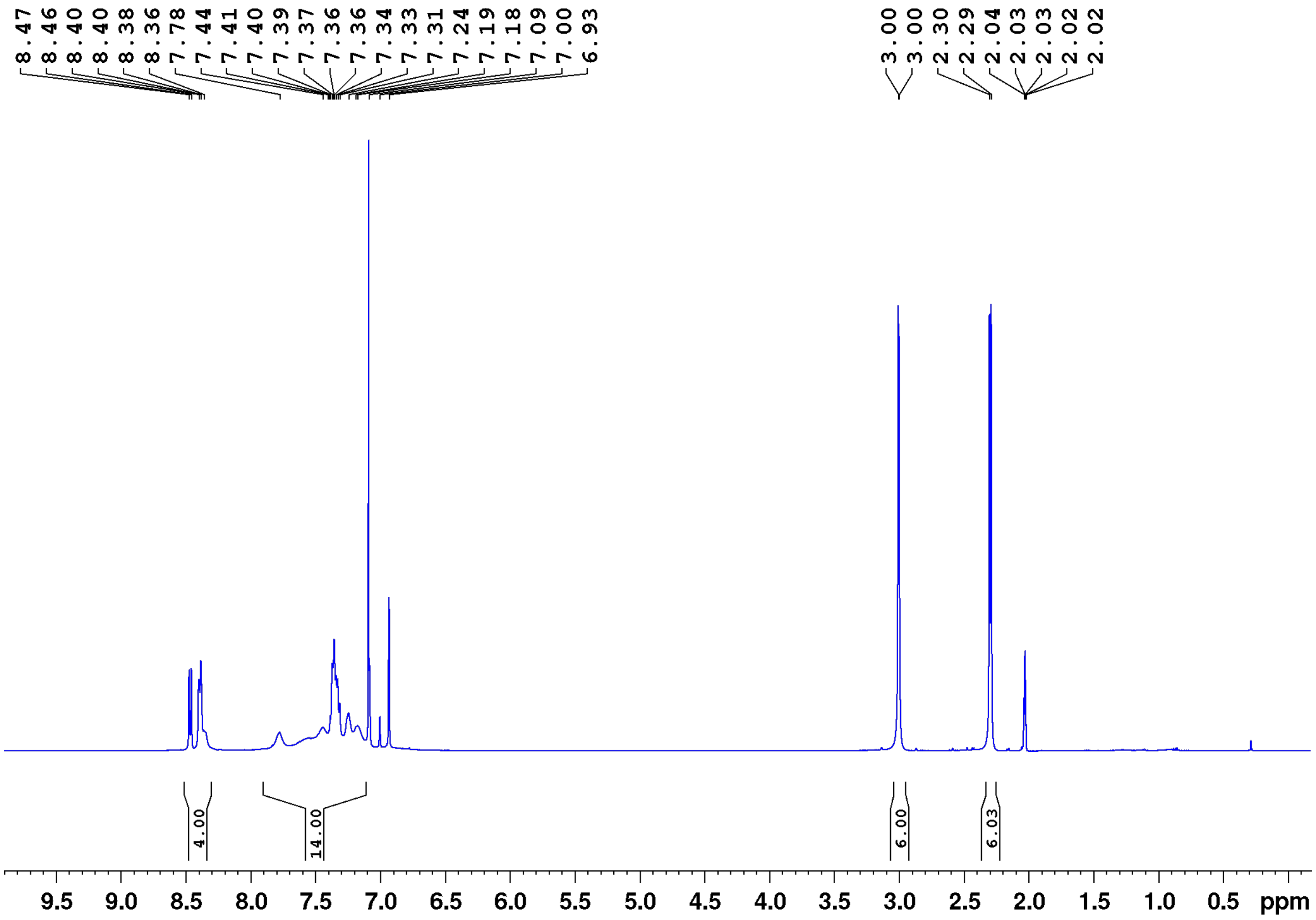

Figure S1. ^{1}H NMR spectrum of **1-Phen** in d_8-toluene at –30 °C.

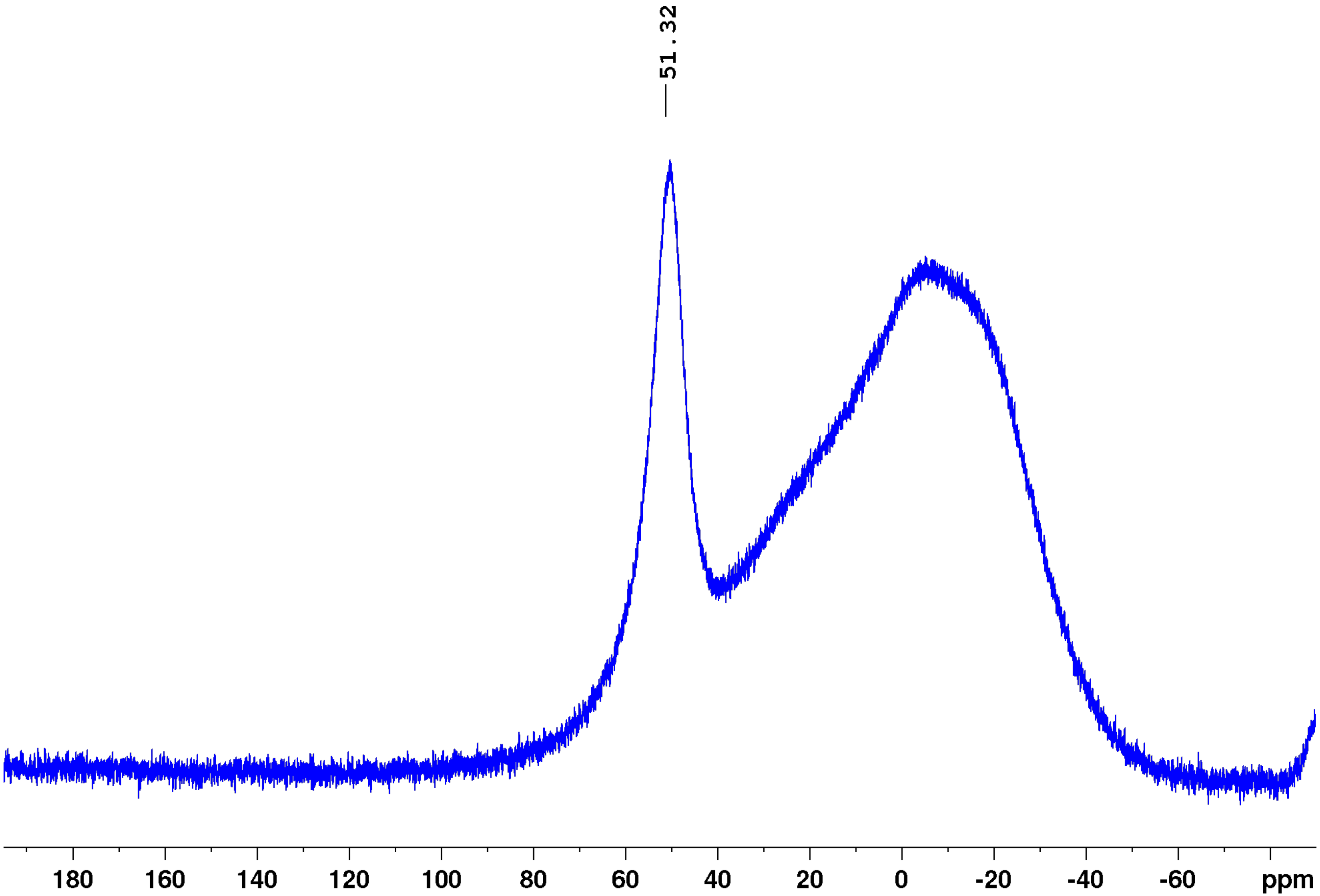

Figure S2. ^{11}B NMR spectrum of **1-Phen** in C_6D_6.

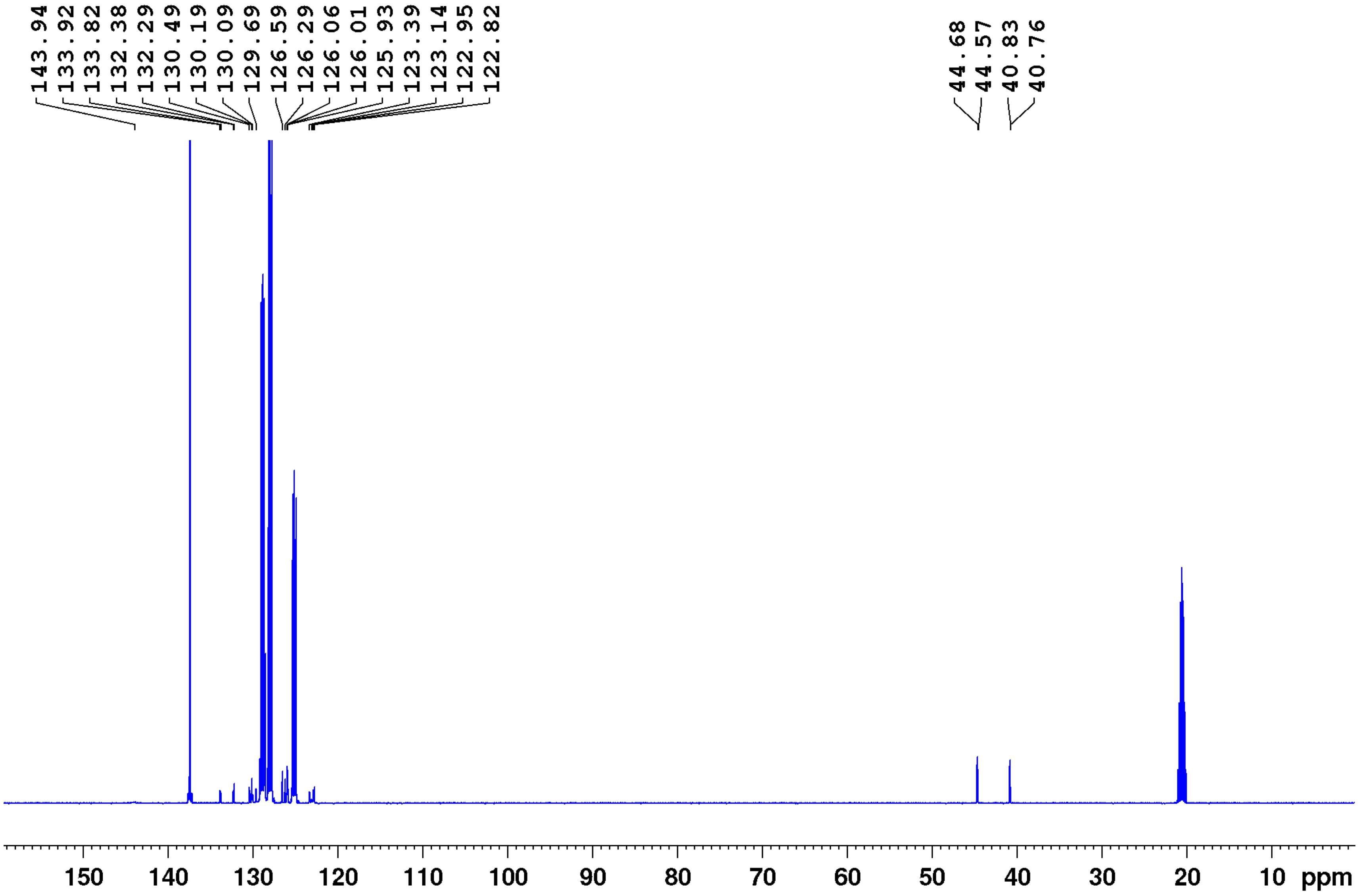

Figure S3. $^{13}C\{^1H\}$ NMR spectrum of **1-Phen** in d_8-toluene at –30 °C.

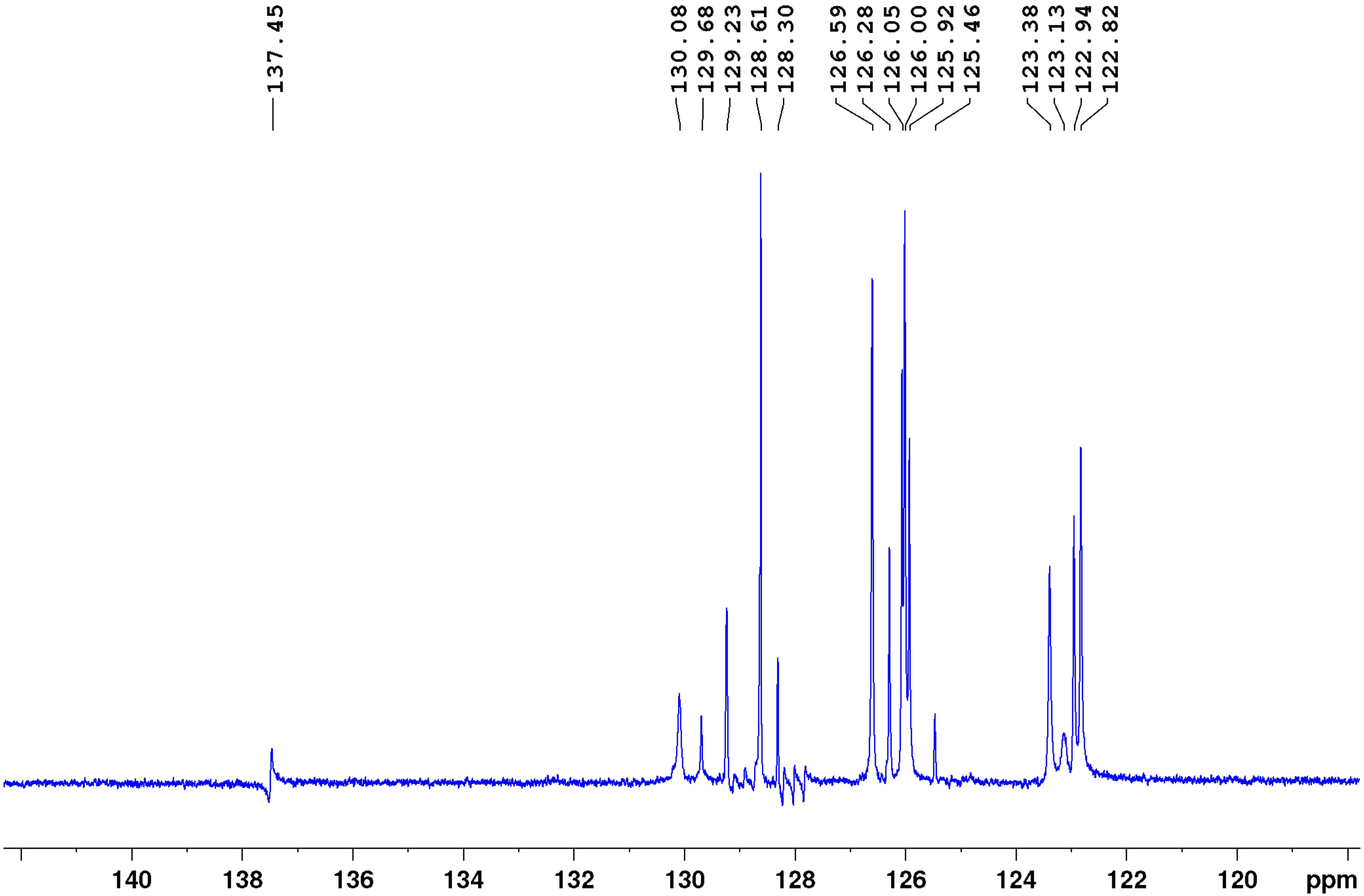

Figure S4. Zoom-in on the aromatic region of the $^{13}C\{^{1}H\}$-dept135 NMR spectrum of **1-Phen** in d_8-toluene at –30 °C.

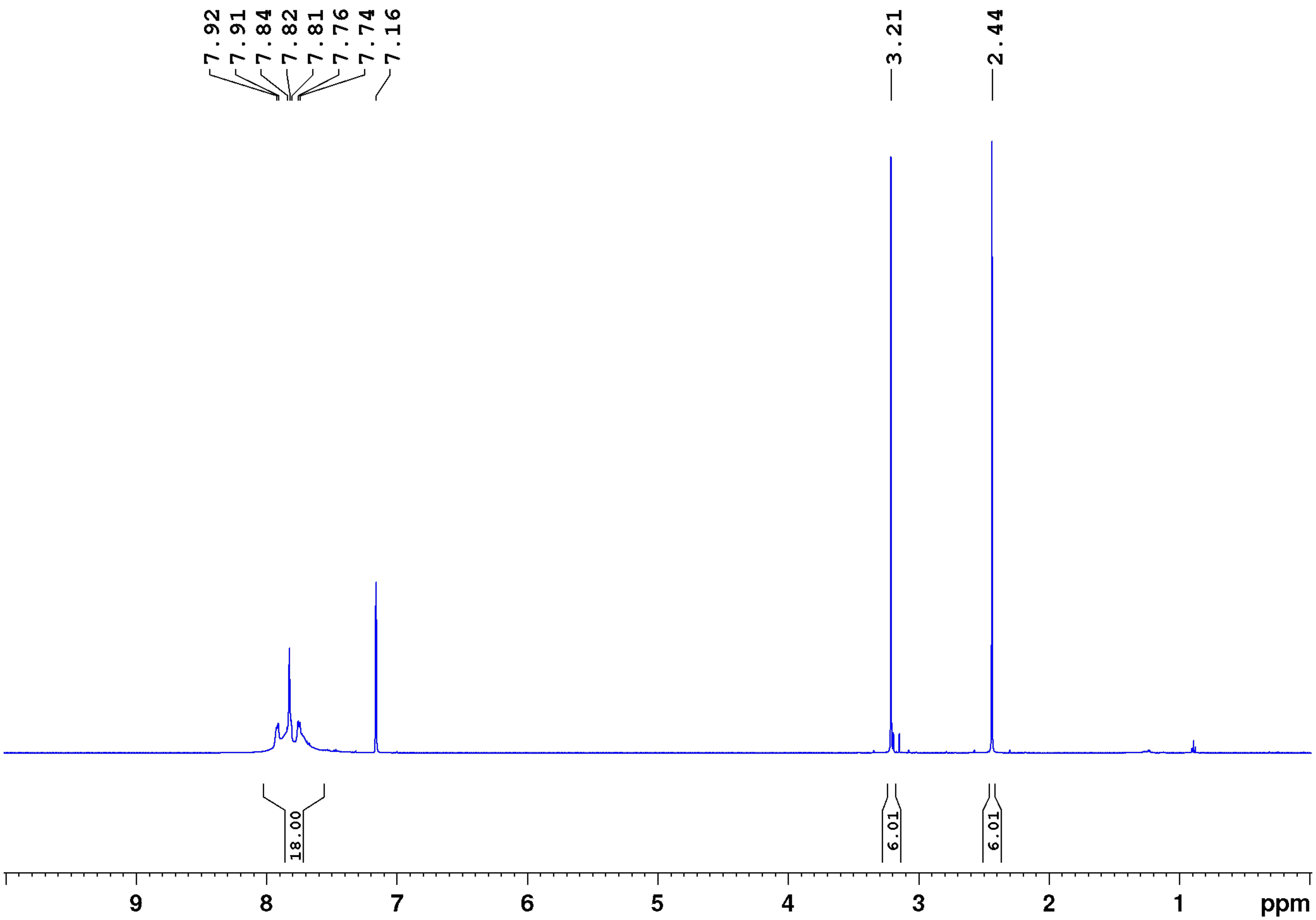

Figure S5. ^{1}H NMR spectrum of **1-Pyr** in C_6D_6.

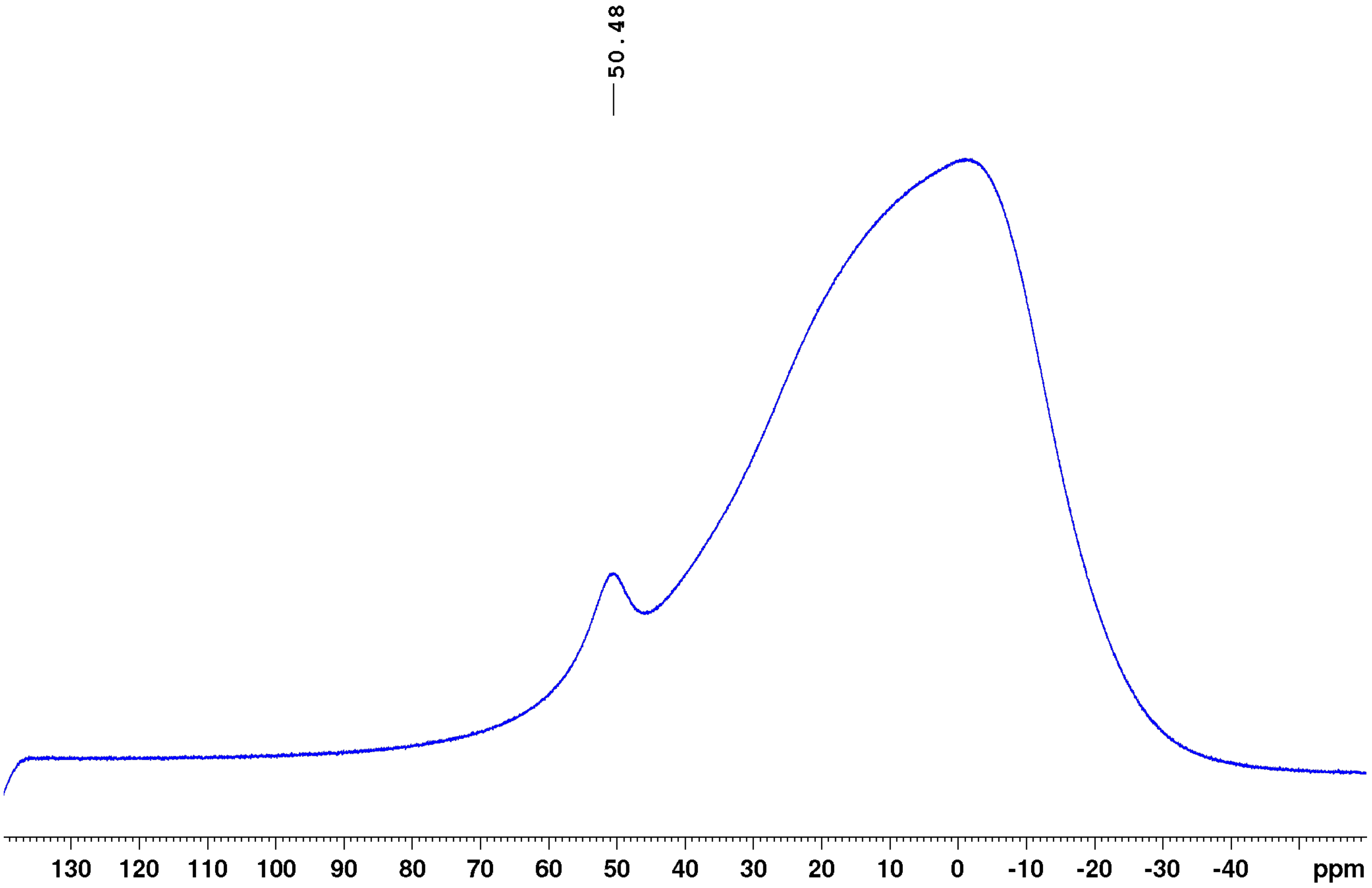

Figure S6. ^{11}B NMR spectrum of **1-Pyr** in C_6D_6.

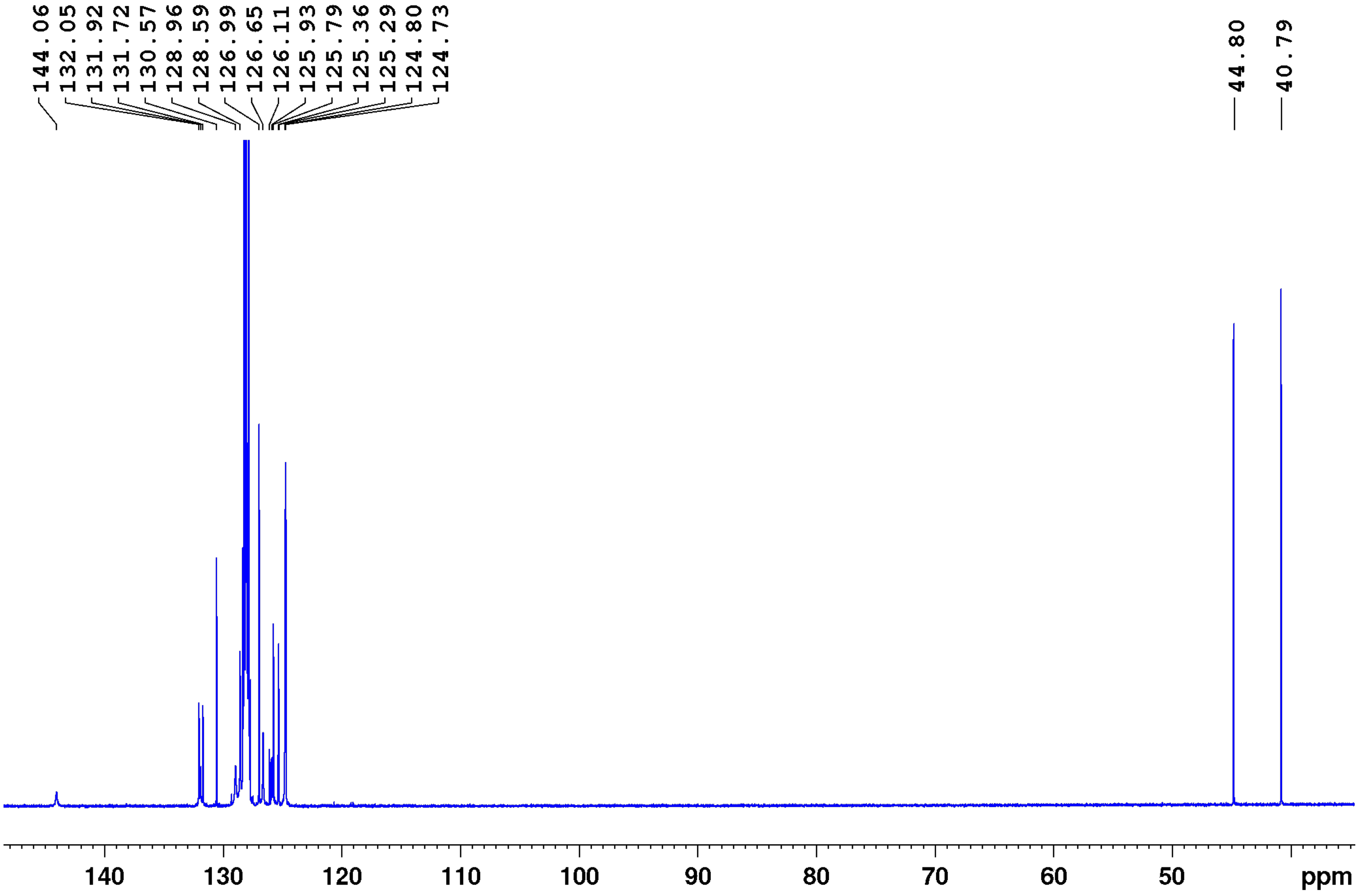

Figure S7. $^{13}C\{^1H\}$ NMR spectrum of **1-Pyr** in C_6D_6.

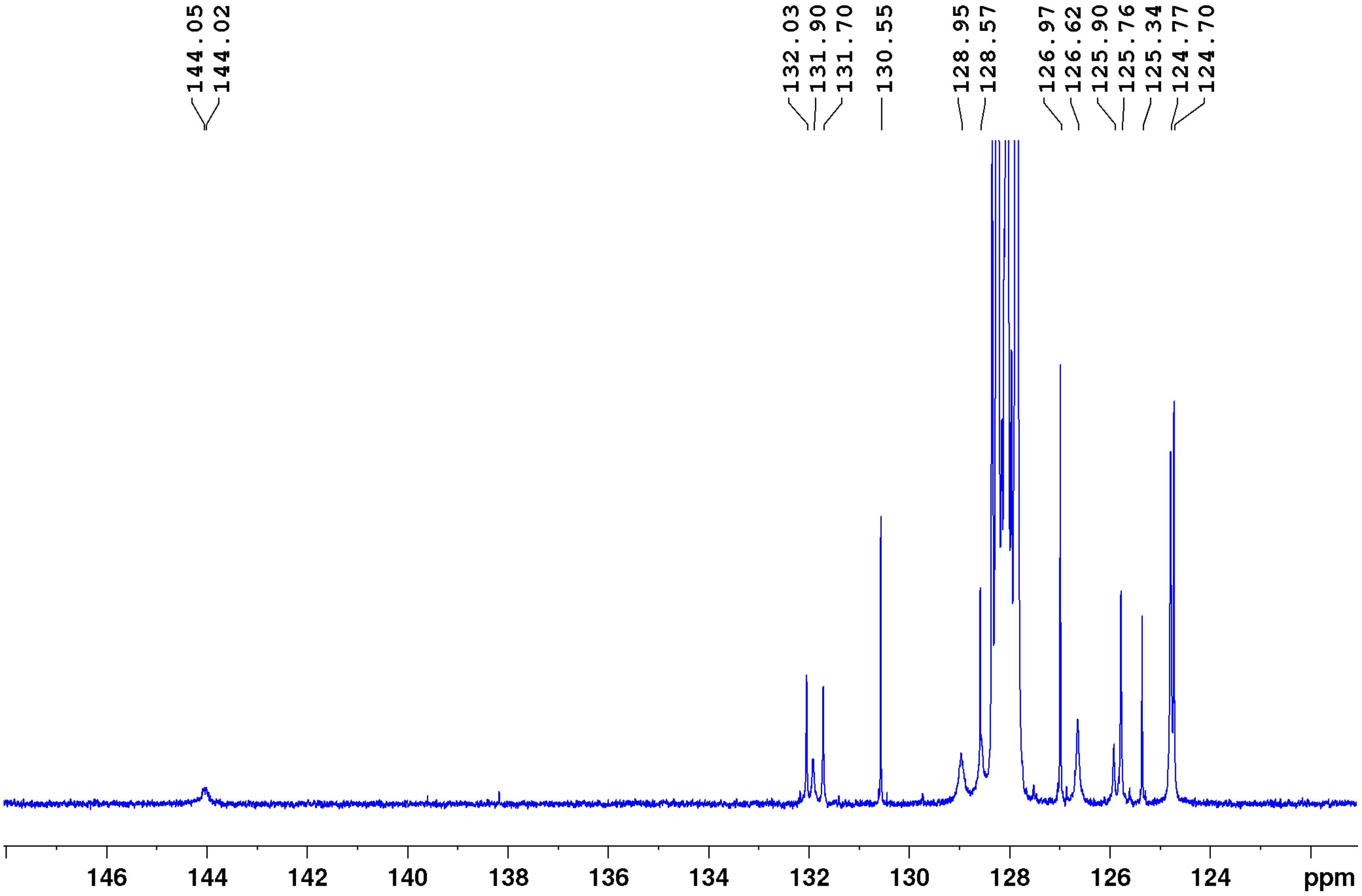

Figure S8. Zoom-in on the aromatic region of the $^{13}C\{^1H\}$ NMR spectrum of **1-Pyr** in d_8-toluene at –30 °C.

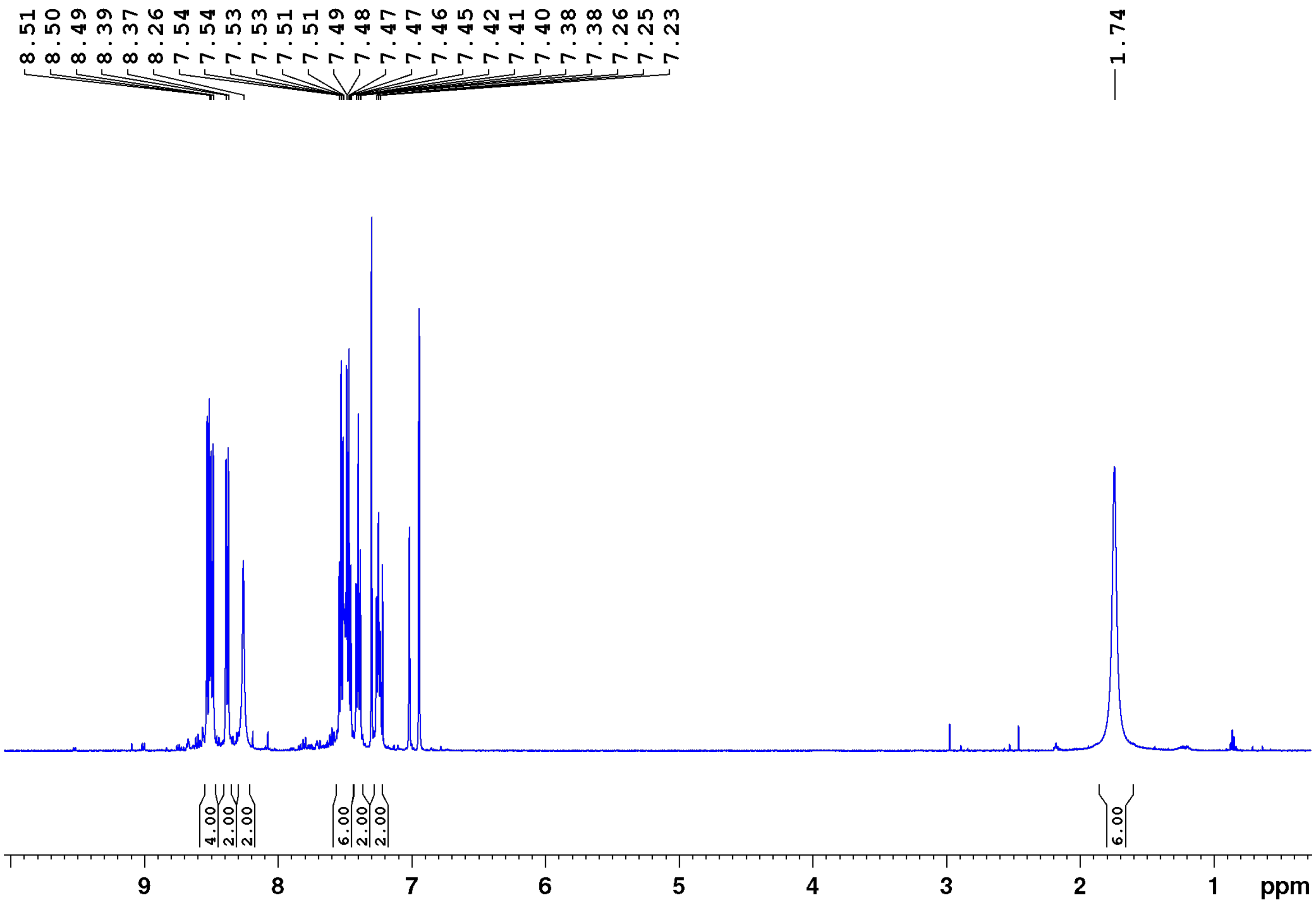

Figure S9. ^{1}H NMR spectrum of **5-Phen** in C_6D_5Br. The impurities visible mostly in the aromatic region account for < 5% of the sample and could not be removed by fractional crystallisation.

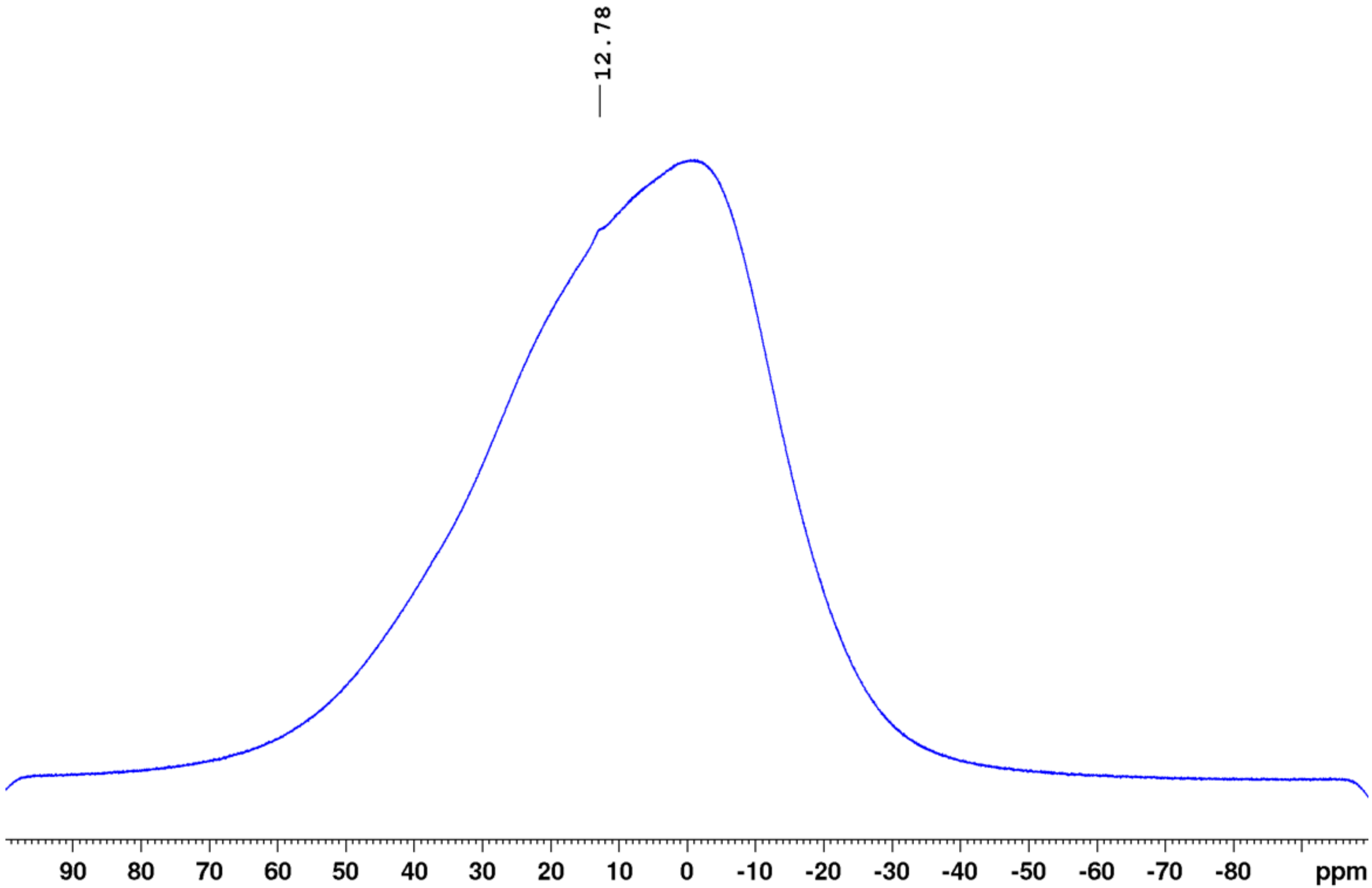

Figure S10. ^{11}B NMR spectrum of **5-Phen** in C_6D_5Br.

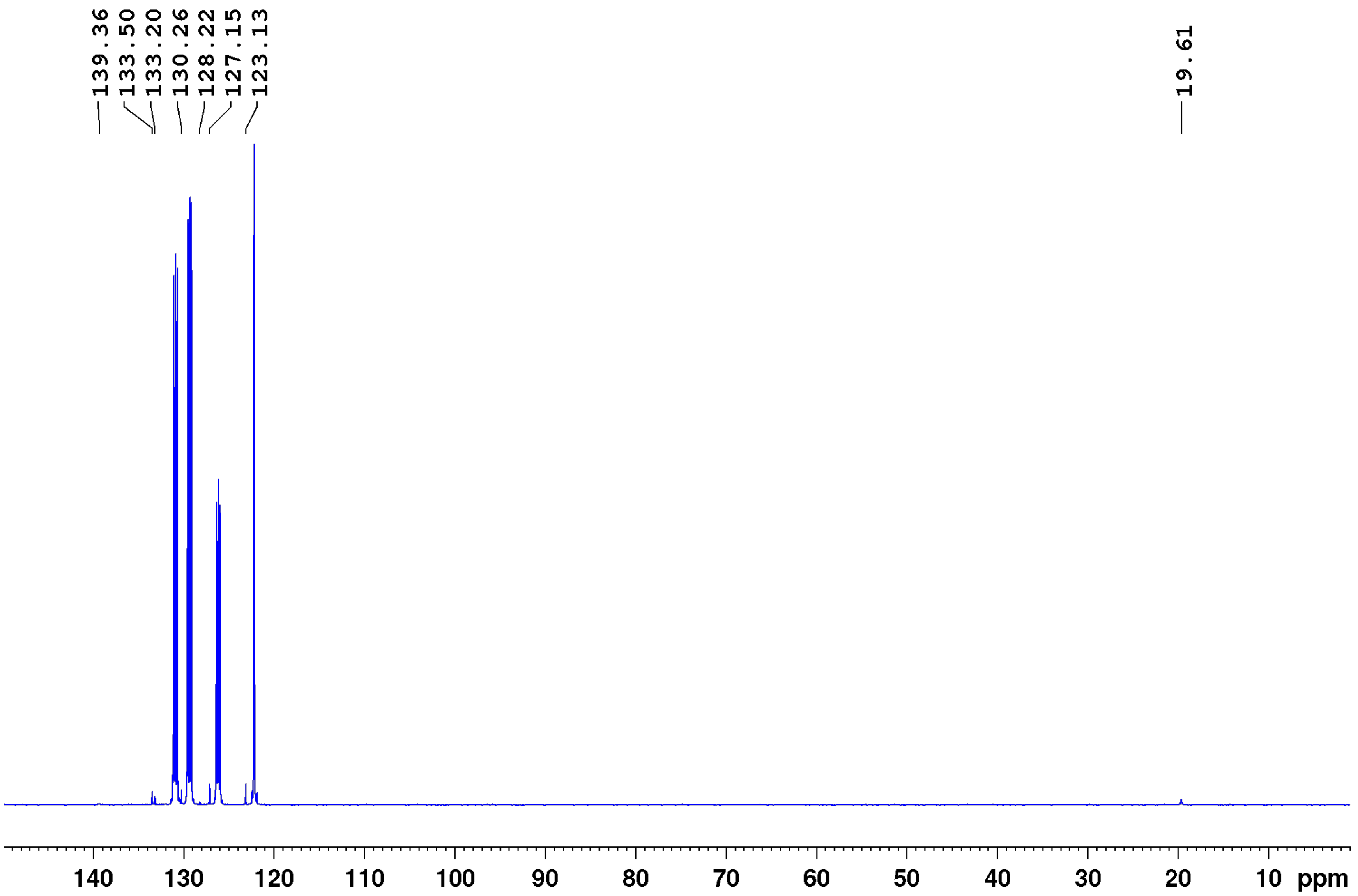

Figure S11. $^{13}C\{^1H\}$ NMR spectrum of **5-Phen** in C_6D_5Br.

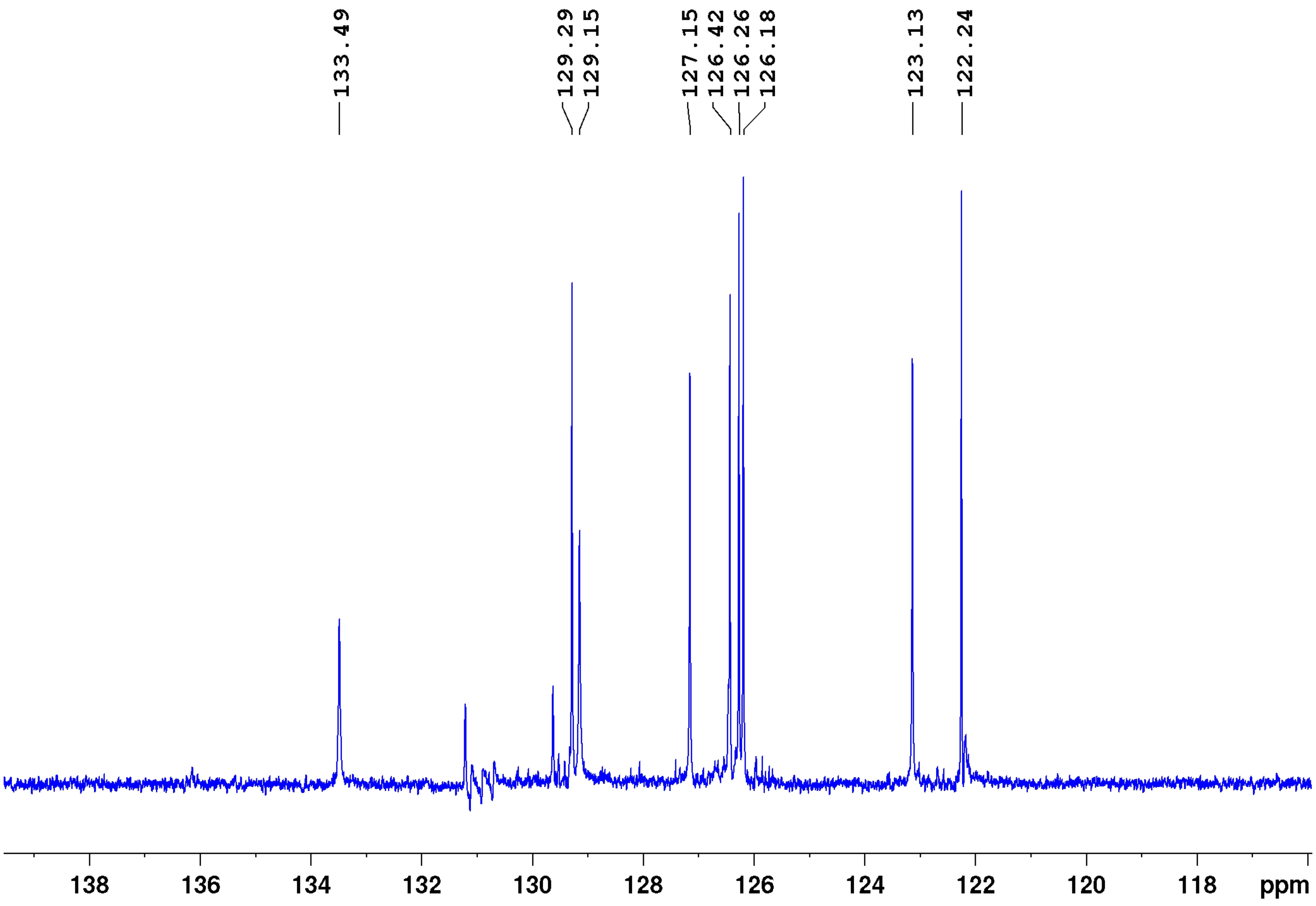

Figure S12. Zoom-in on the aromatic region of the $^{13}C\{^{1}H\}$-dept135 NMR spectrum of **5-Phen** in C_6D_5Br.

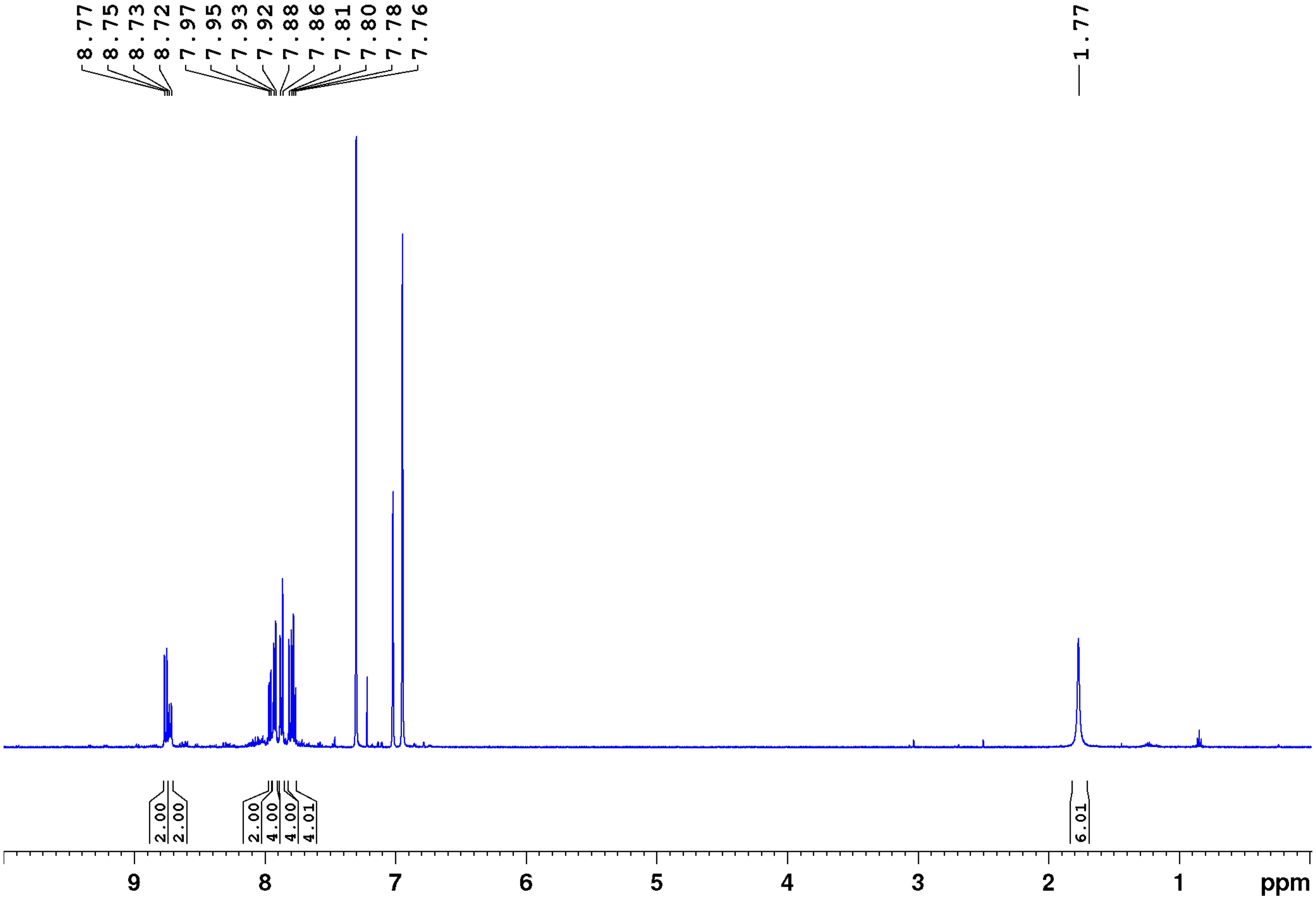

Figure S13. ^{1}H NMR spectrum of **5-Pyr** in C_6D_5Br.

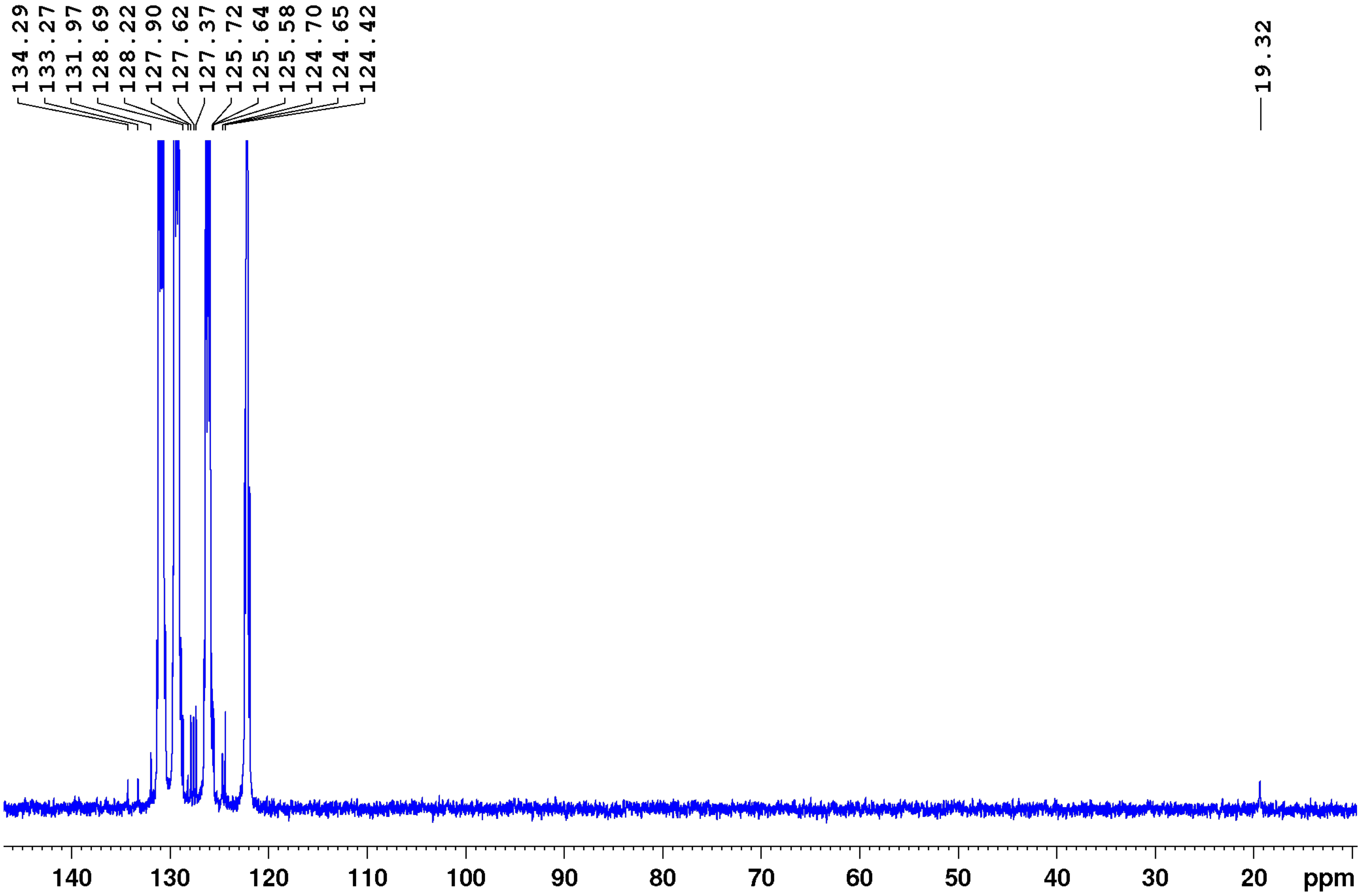

Figure S14. $^{13}C\{^{1}H\}$ NMR spectrum of **5-Pyr** in C_6D_5Br.

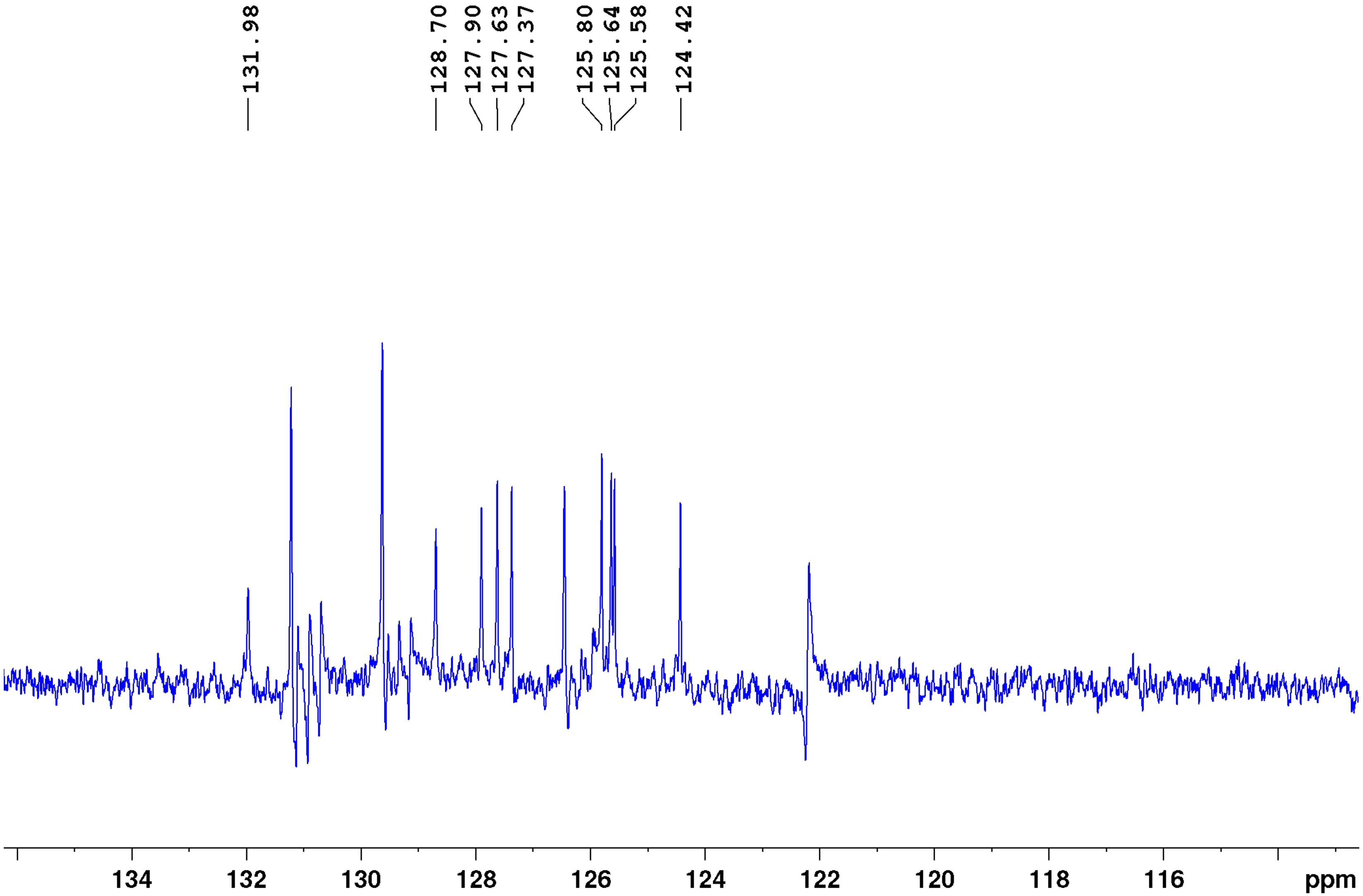

Figure S15. Zoom-in on the aromatic region of the $^{13}C\{^1H\}$-dept135 NMR spectrum of **5-Pyr** in C_6D_5Br.

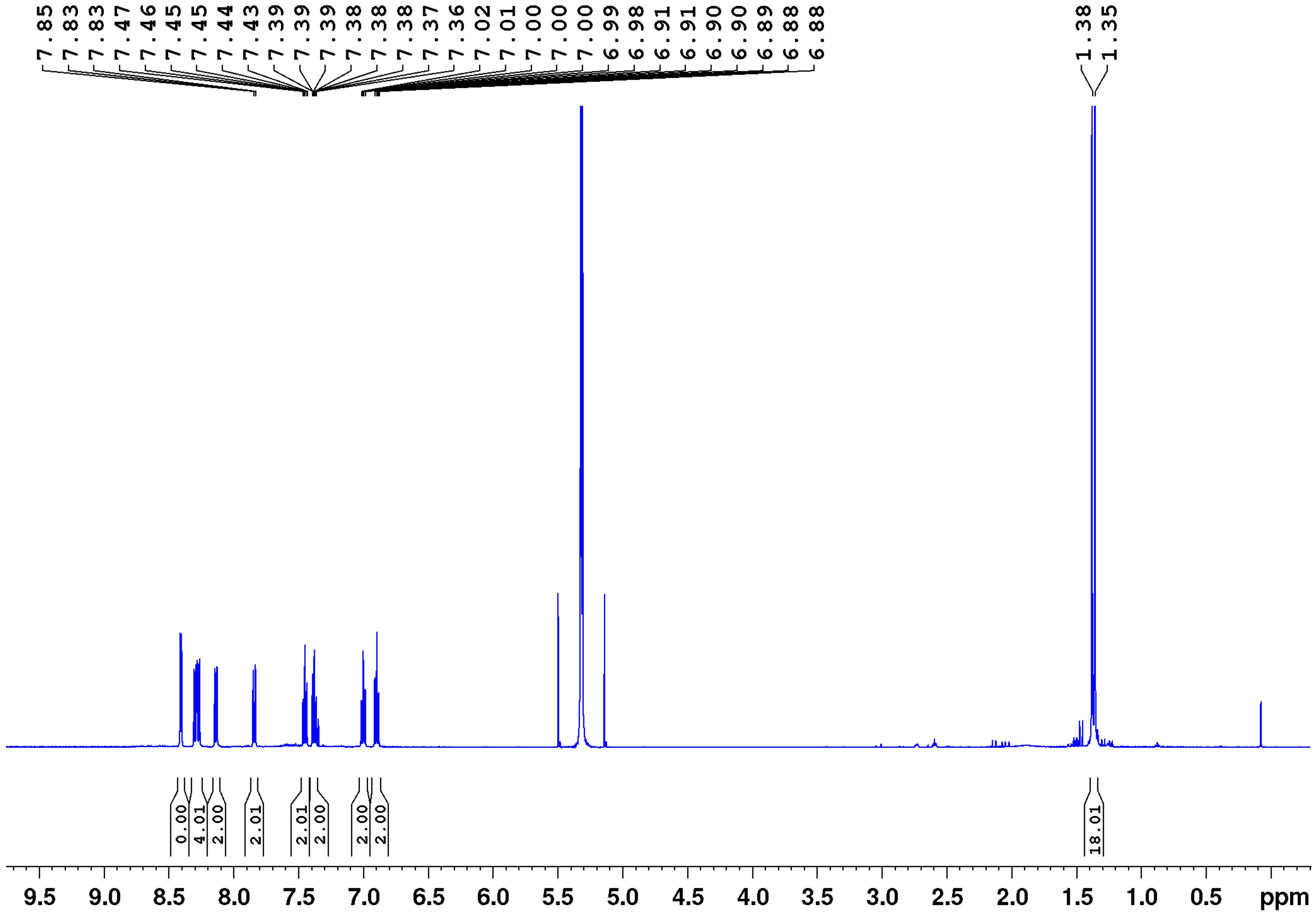

Figure S16. ^{1}H NMR spectrum of **6-Phen** in CD_2Cl_2.

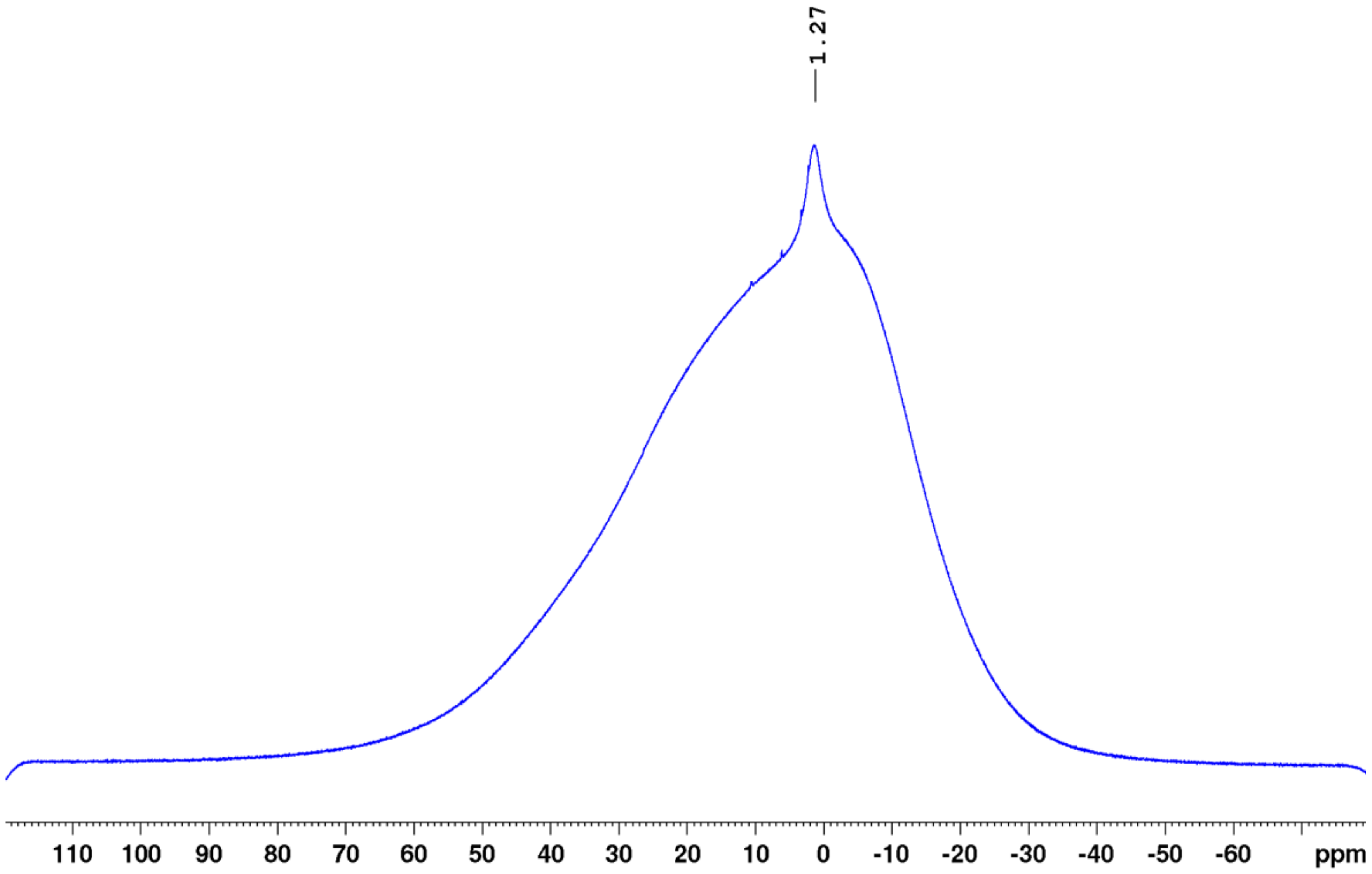

Figure S17. ^{11}B NMR spectrum of **6-Phen** in $C_2D_2Cl_2$.

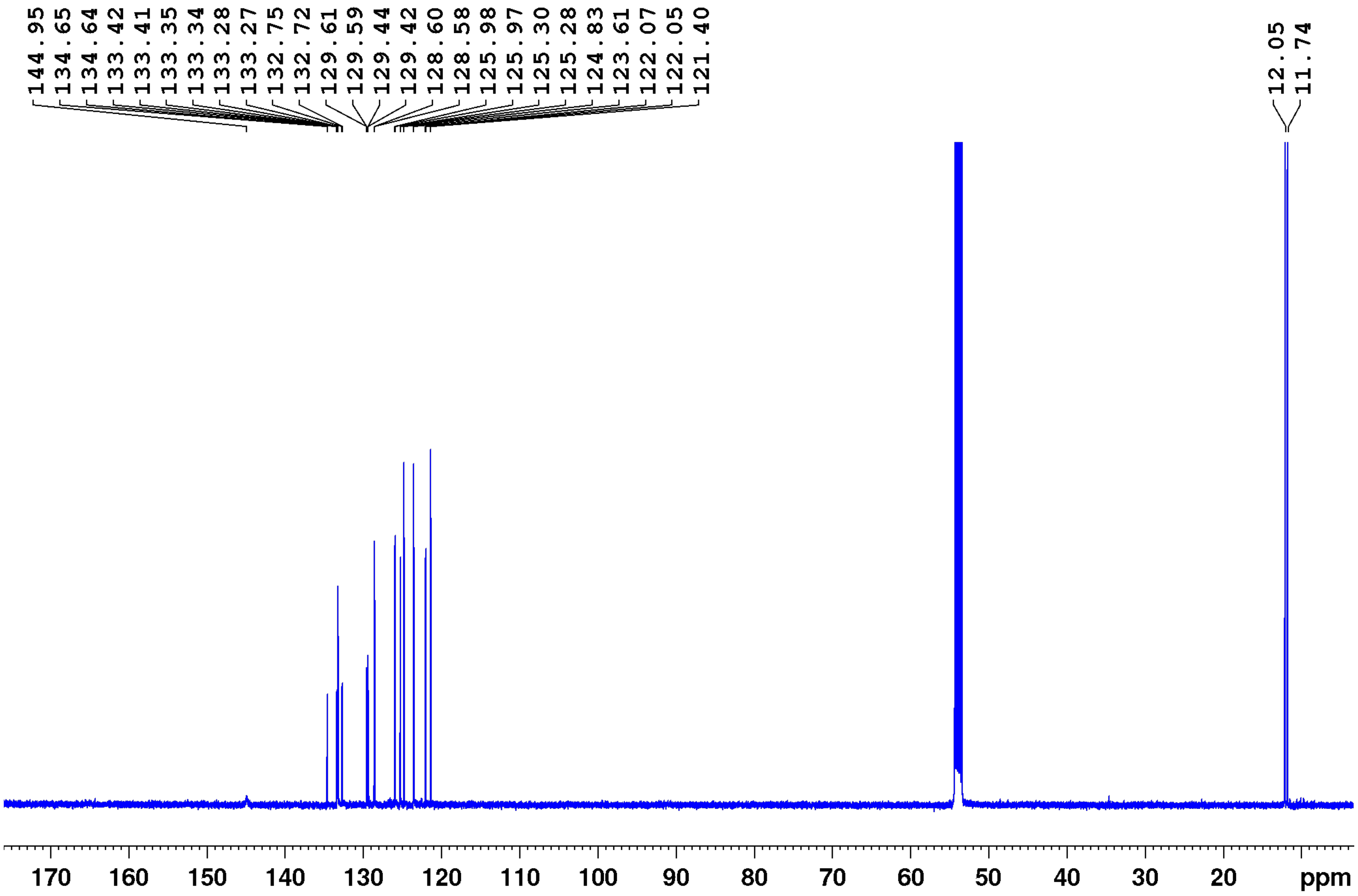

Figure S18. $^{13}C\{^1H\}$ NMR spectrum of **6-Phen** in $C_2D_2Cl_2$.

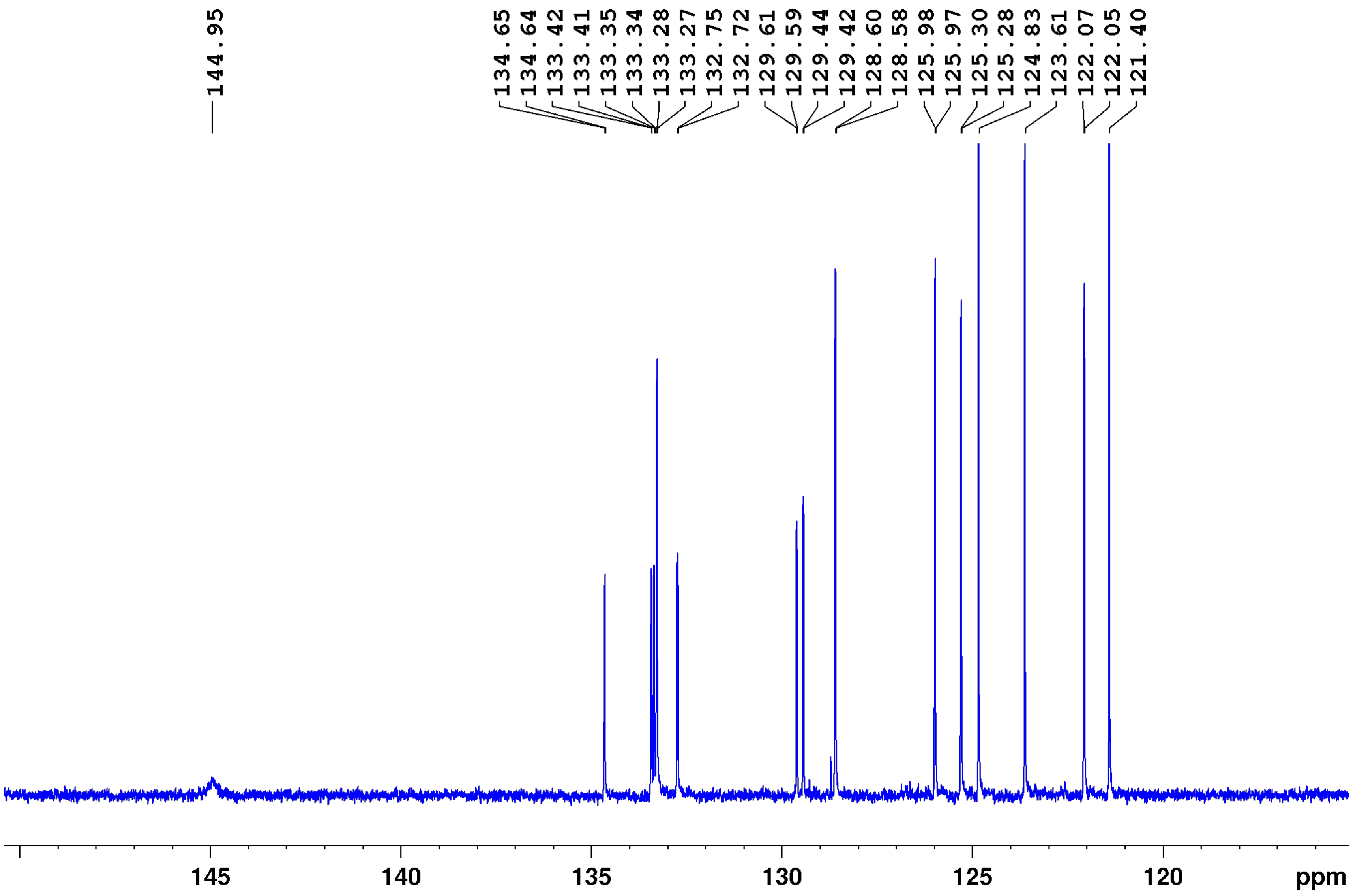

Figure S19. Zoom-in on the aromatic region of the $^{13}C\{^{1}H\}$ NMR spectrum of **6-Phen** in C_6D_5Br.

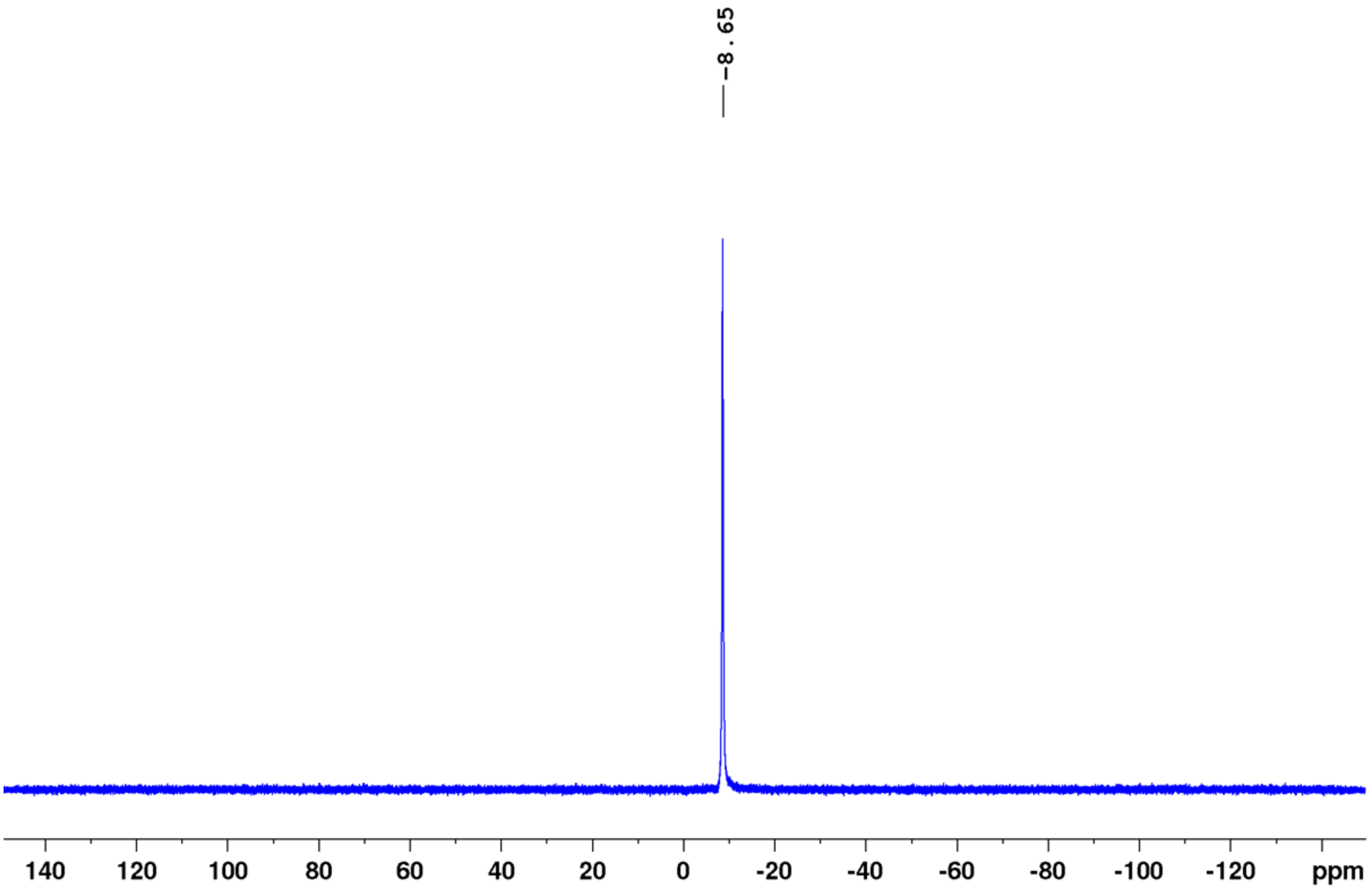

Figure S20. $^{31}P\{^{1}H\}$ NMR spectrum of **6-Phen** in $C_2D_2Cl_2$.

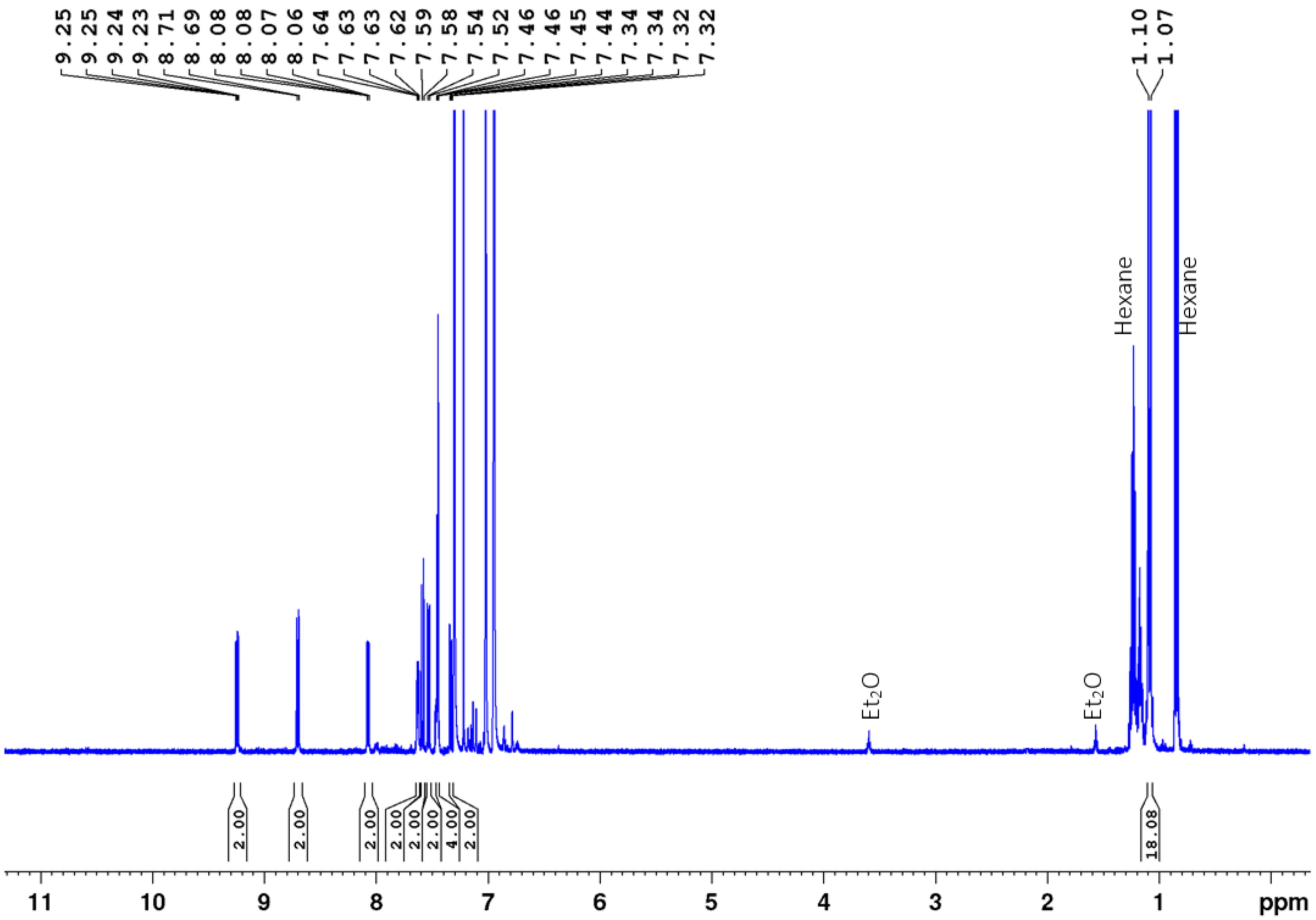

Figure S21. ^{1}H NMR spectrum of **6-Pyr** in C_6D_5Br (residual Et_2O and hexane from washing).

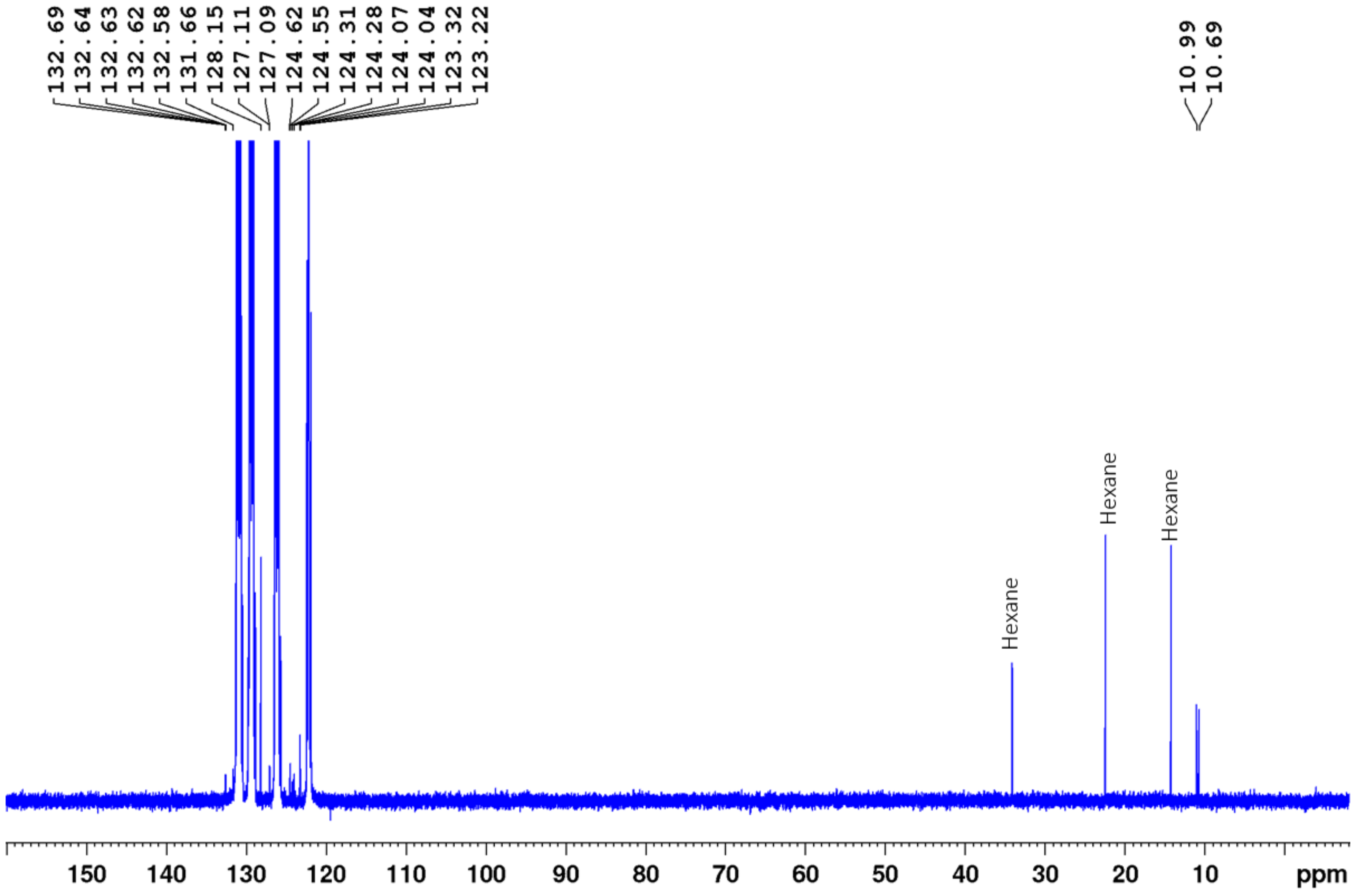

Figure S22. $^{13}C\{^{1}H\}$ NMR spectrum of **6-Pyr** in C_6D_5Br (residual Et_2O and hexane from washing).

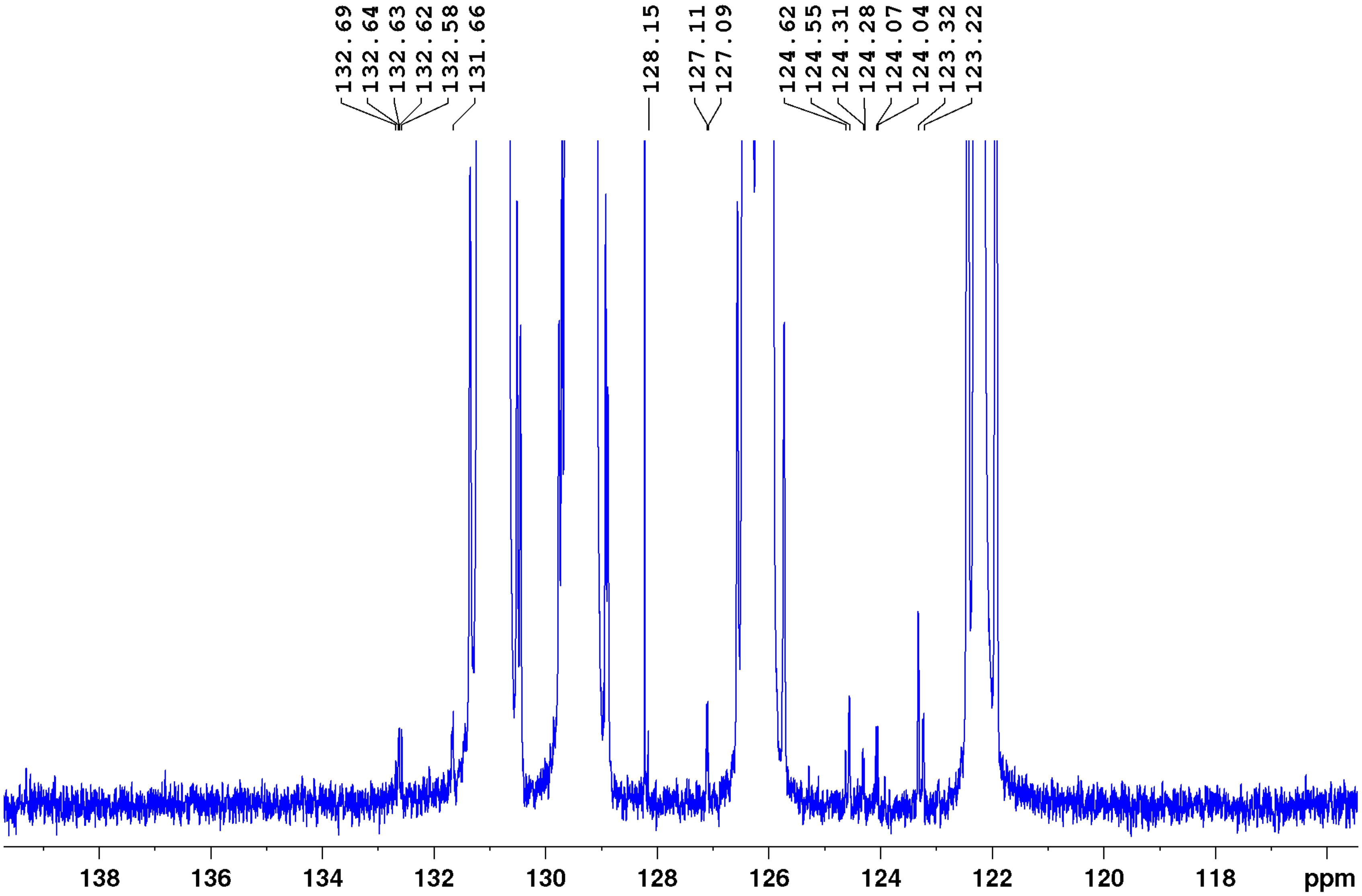

Figure S23. Zoom-in on the aromatic region of the $^{13}C\{^{1}H\}$ spectrum of **6-Pyr** in C_6D_5Br.

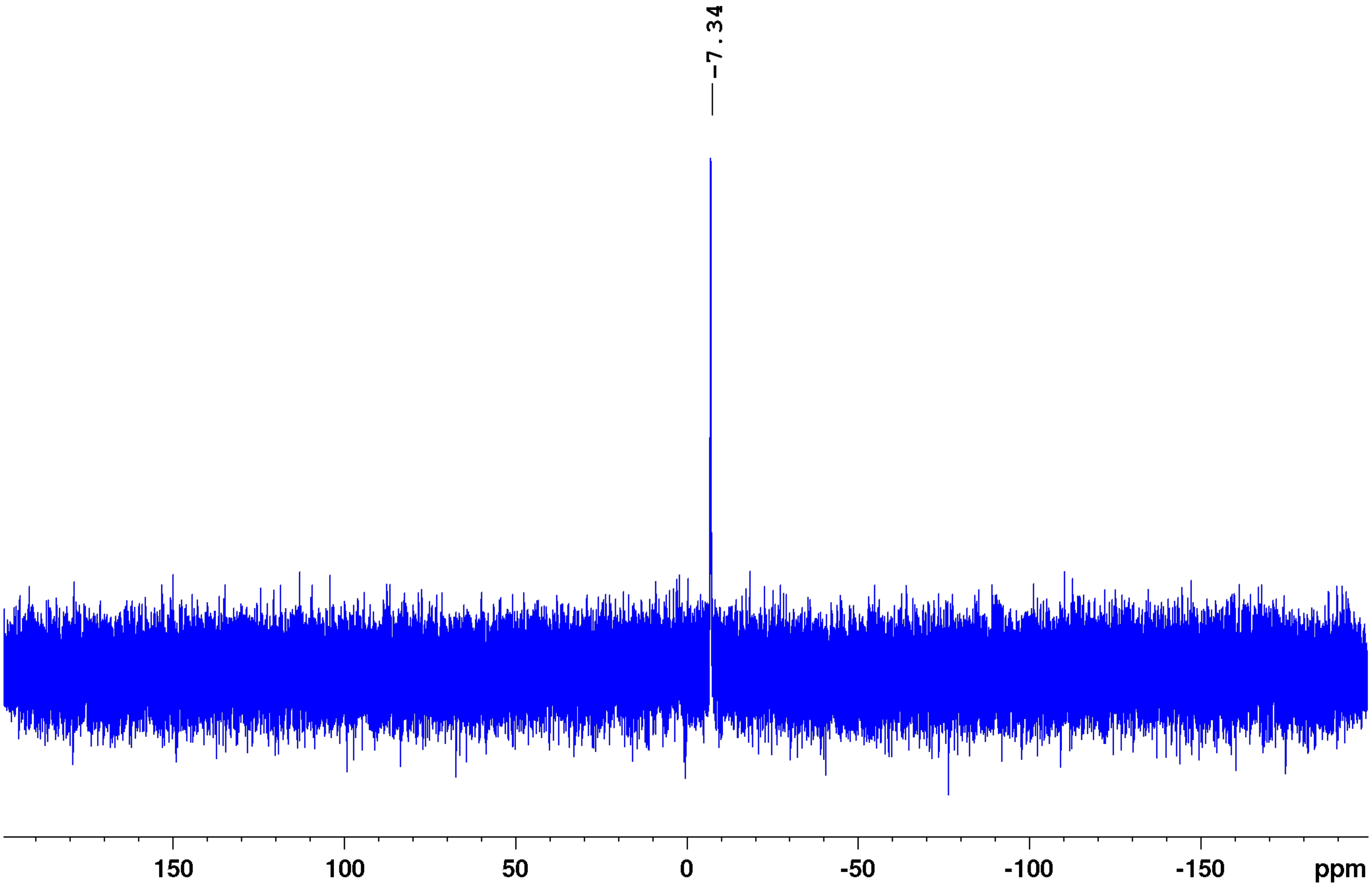

Figure S24. $^{31}P\{^{1}H\}$ NMR spectrum of **6-Pyr** in C_6D_5Br.

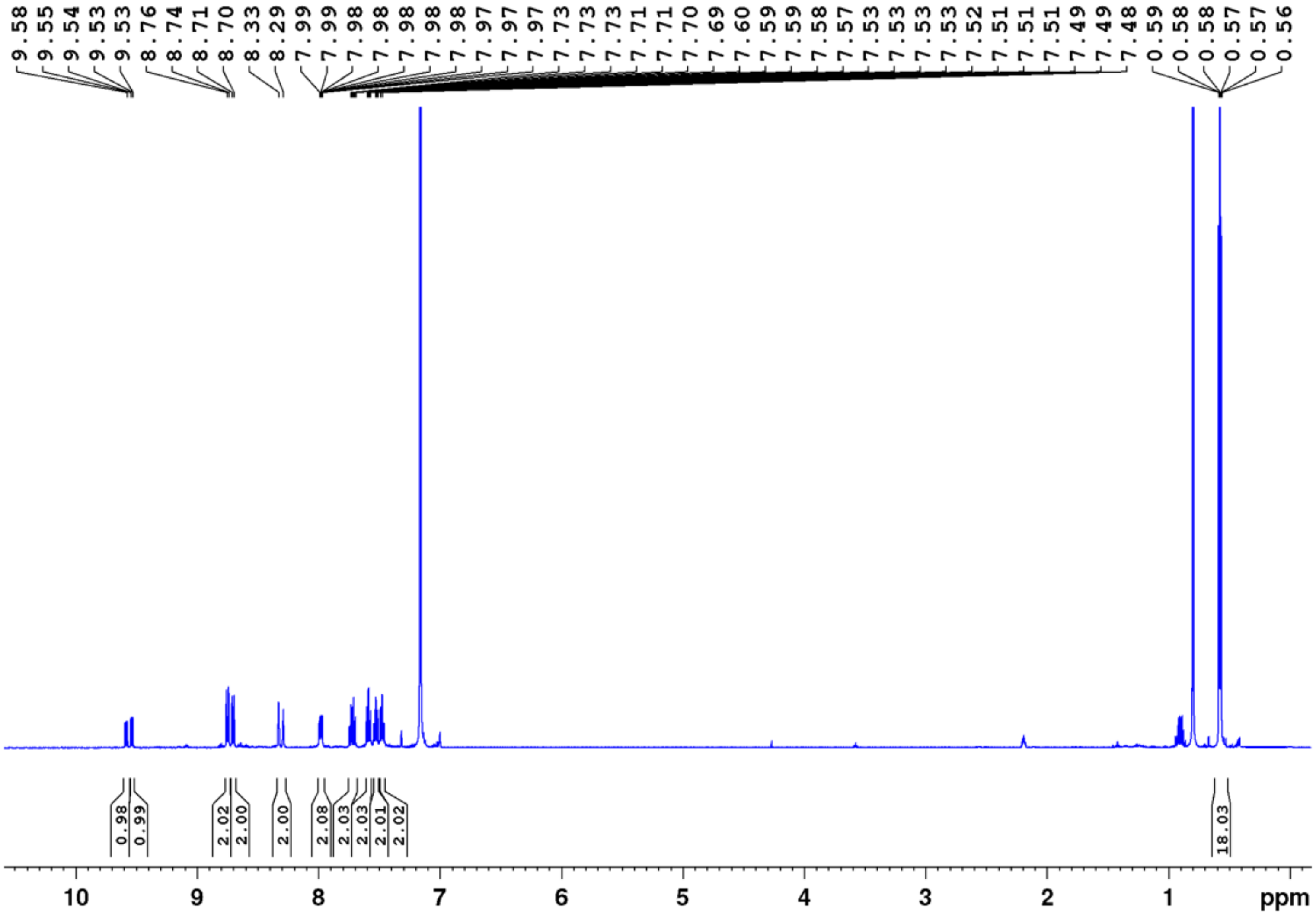

Figure S25. ^{1}H NMR spectrum of **7-Phen** in C_6D_6. The additional doublet at 0.8 ppm corresponds to residual PMe_3, without which **7-Phen** is not stable.

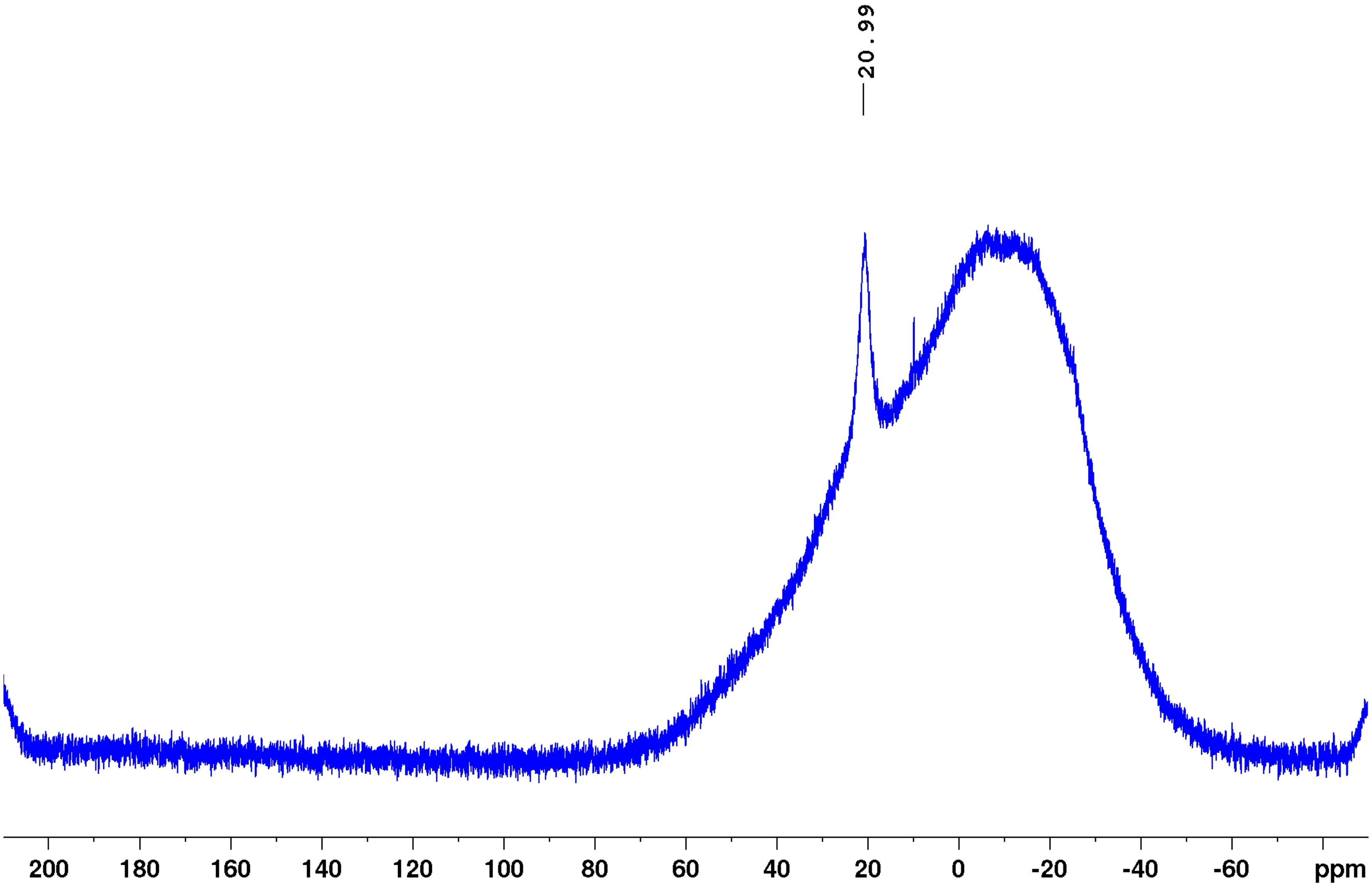

Figure S26. ^{11}B NMR spectrum of **7-Phen** in C_6D_6/thf (3:1).

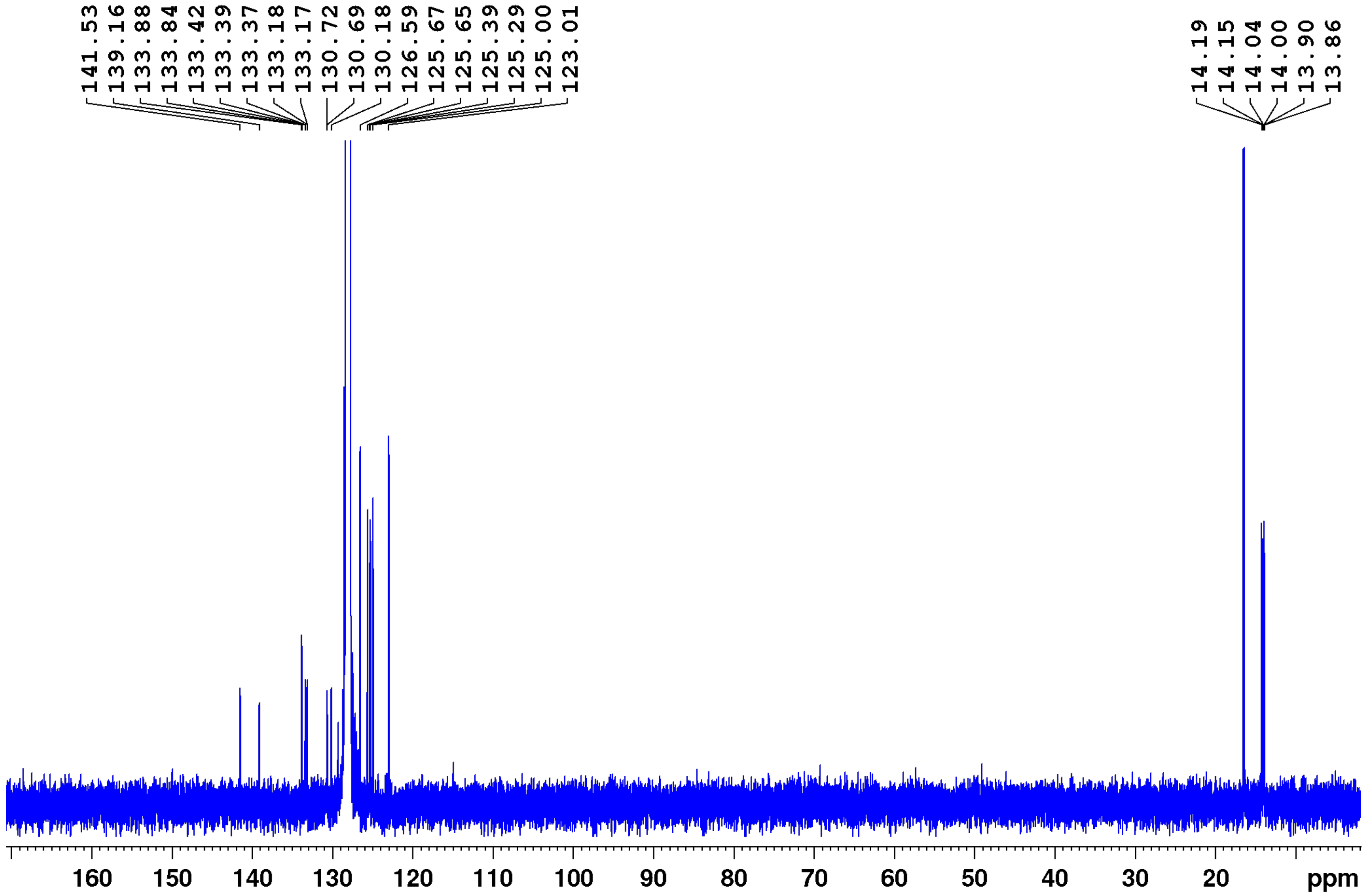

Figure S27. $^{13}C\{^1H\}$ NMR spectrum of **7-Phen** in C_6D_6. The additional doublet at 16.4 ppm corresponds to residual PMe_3, without which **7-Phen** is not stable.

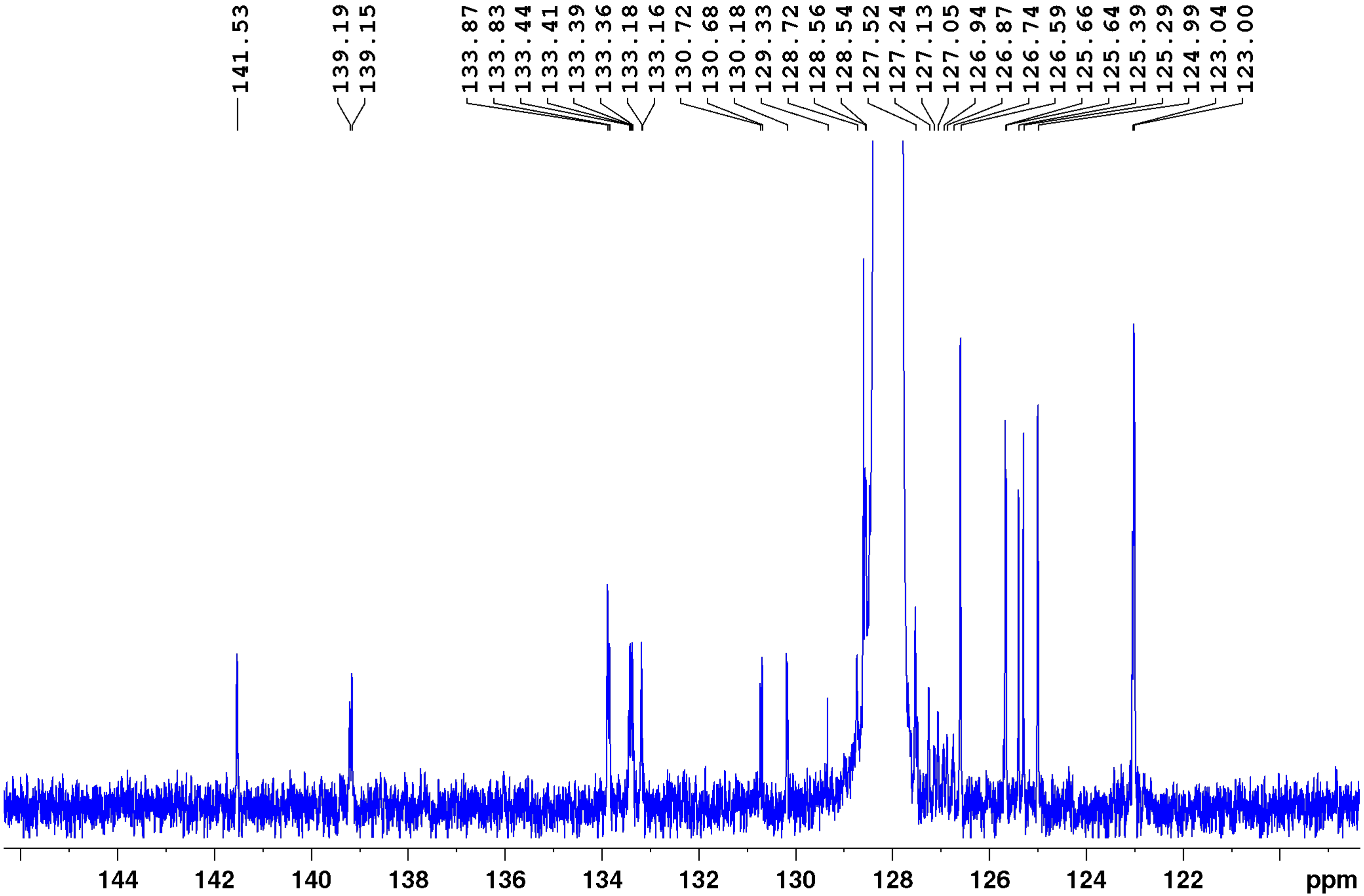

Figure S28. Zoom-in on the aromatic region of the $^{13}C\{^1H\}$ spectrum of **7-Phen** in C_6D_6.

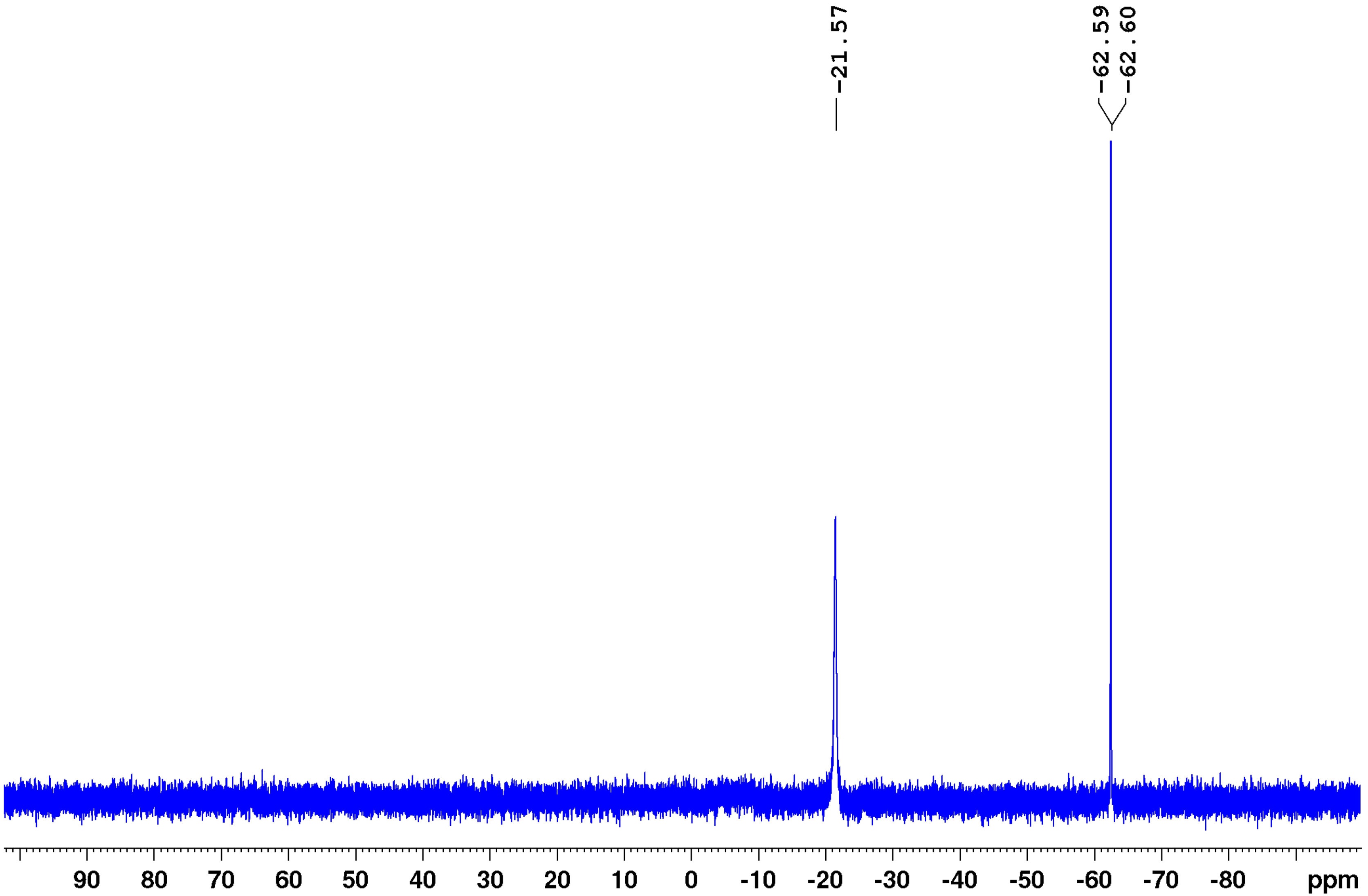

Figure S29. $^{31}P\{^{1}H\}$ NMR spectrum of **7-Phen** in C_6D_6. The additional singlet at –62.6 ppm corresponds to residual PMe_3, without which **7-Phen** is not stable.

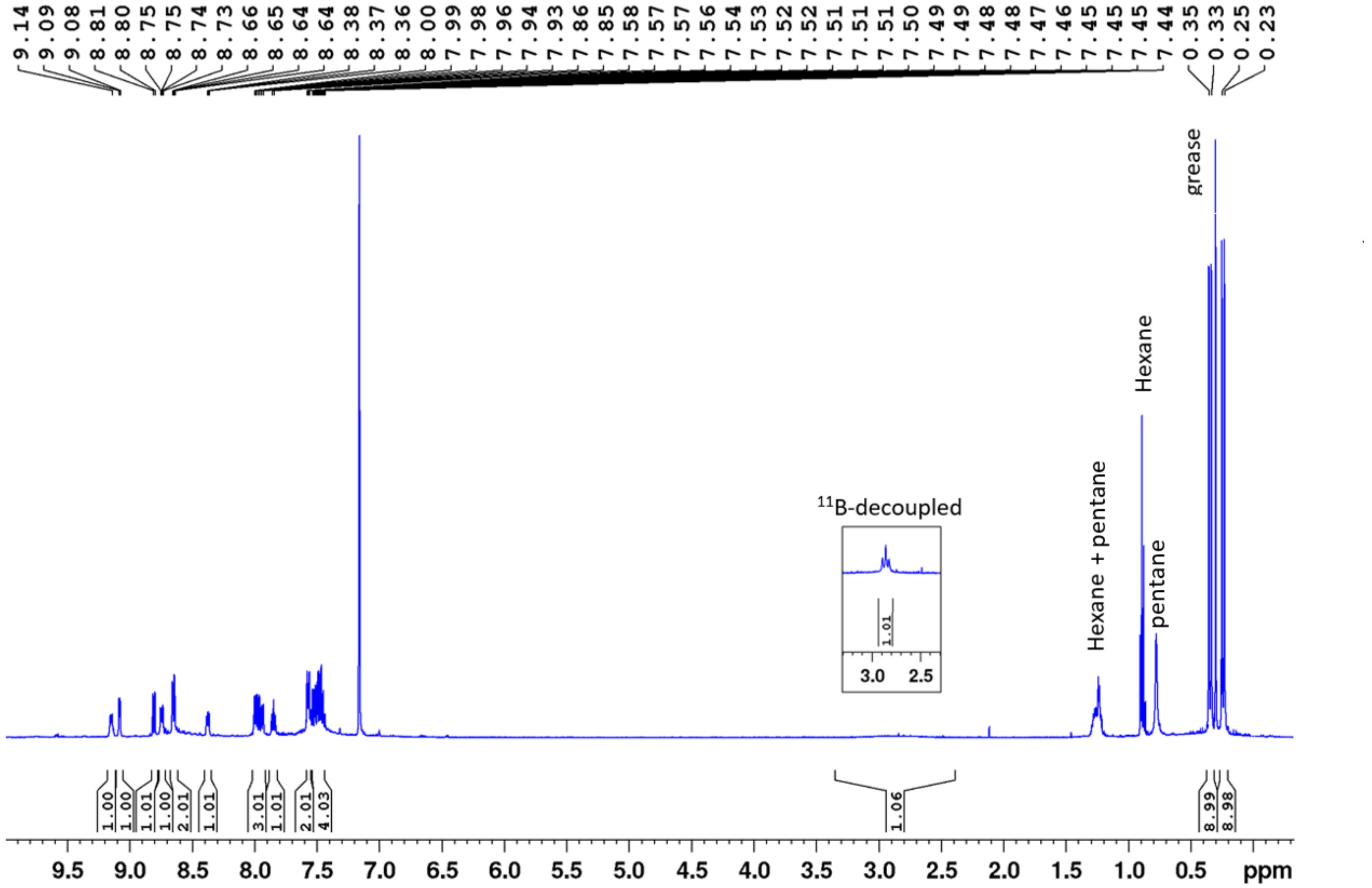

Figure S30. ^{1}H NMR spectrum of **8-Phen** in C_6D_6 (residual solvent from washing). Insert = selective ^{11}B-decoupling for δ_{11B} = –22 pm.

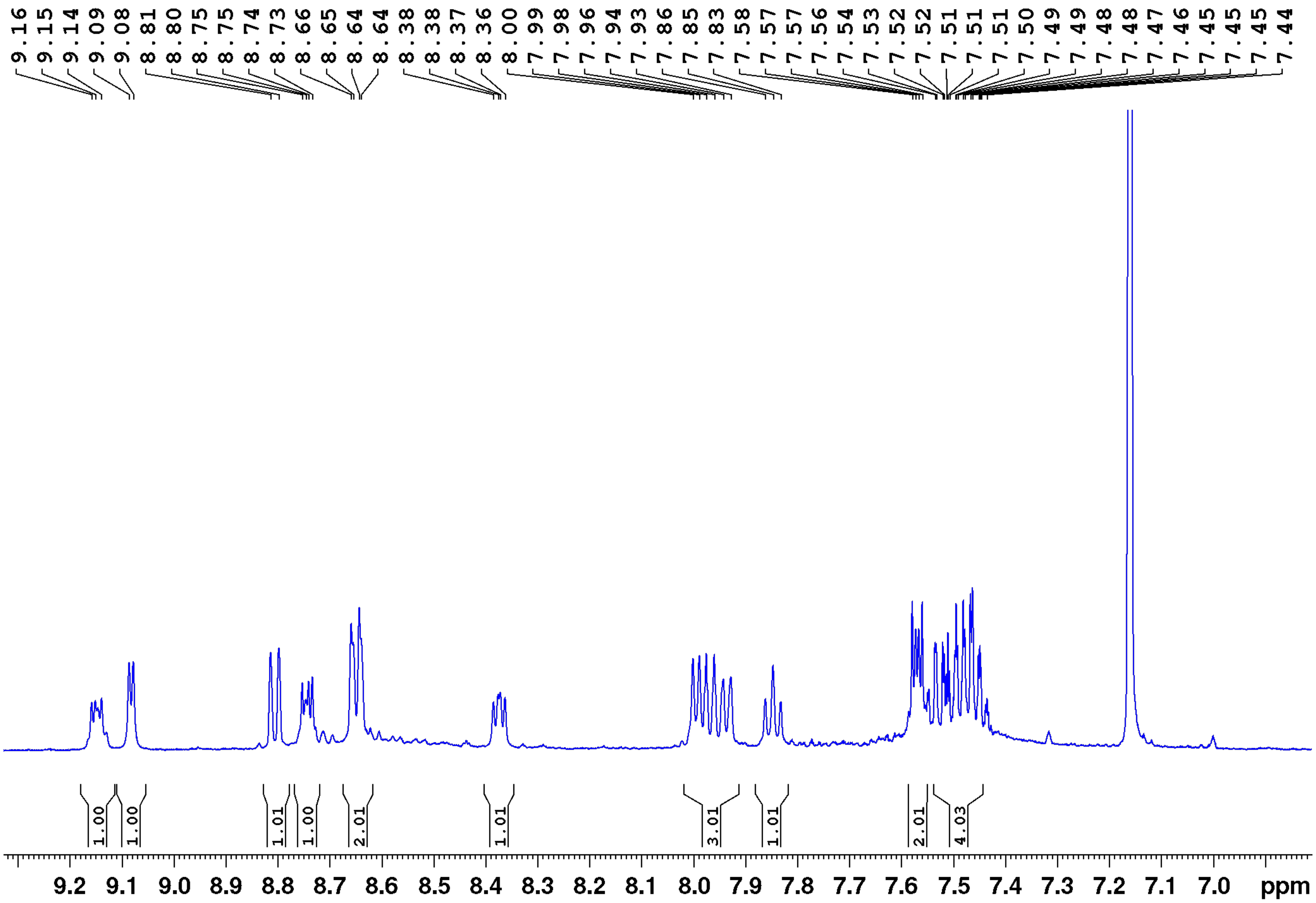

Figure S31. Zoom-in on the aromatic region of the $^{1}H\{^{11}B\}$ NMR spectrum of **8-Phen** in C_6D_6.

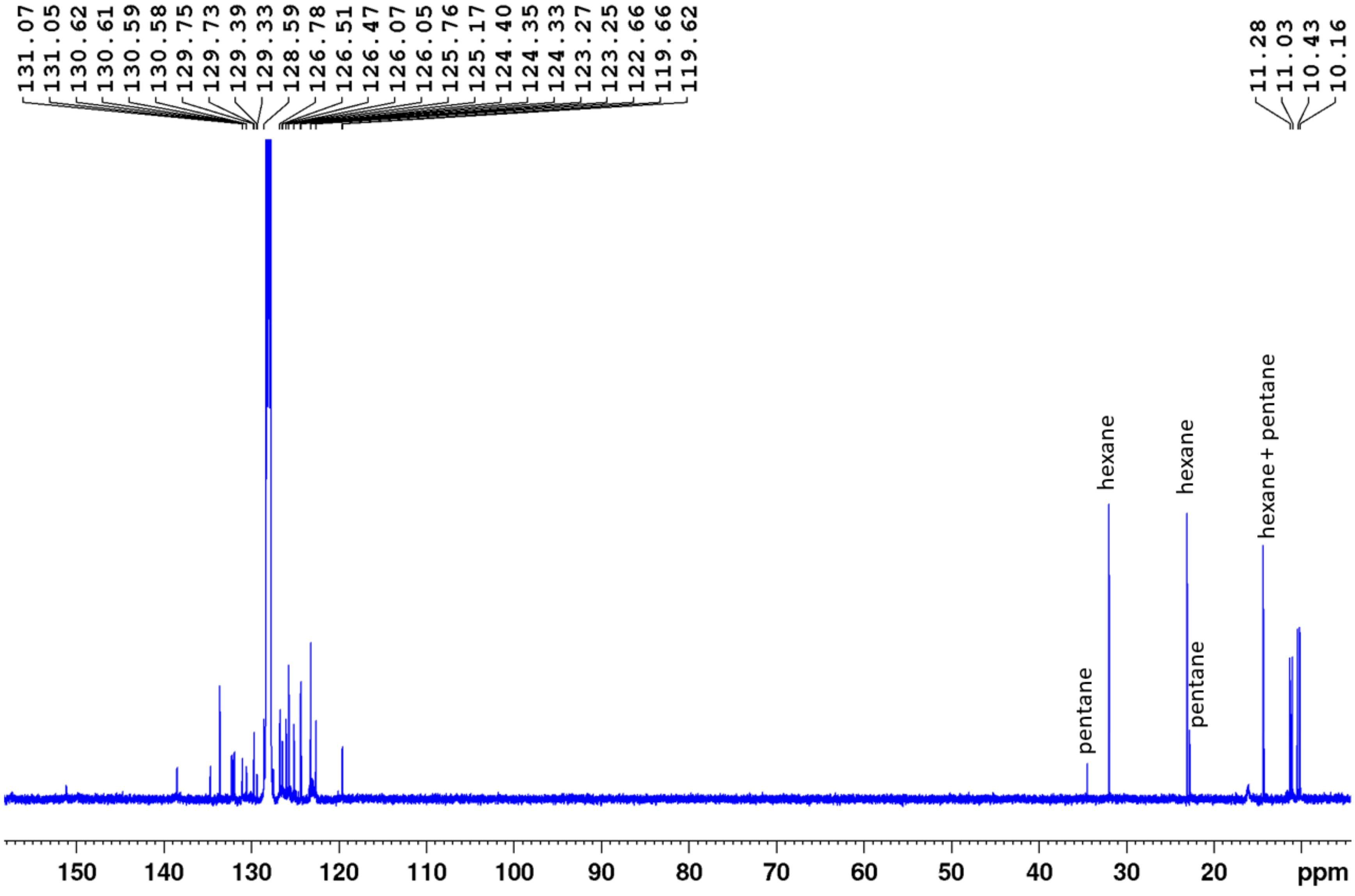

Figure S32. $^{13}C\{^{1}H\}$ NMR spectrum of **8-Phen** in C_6D_6 (residual solvent from washing).

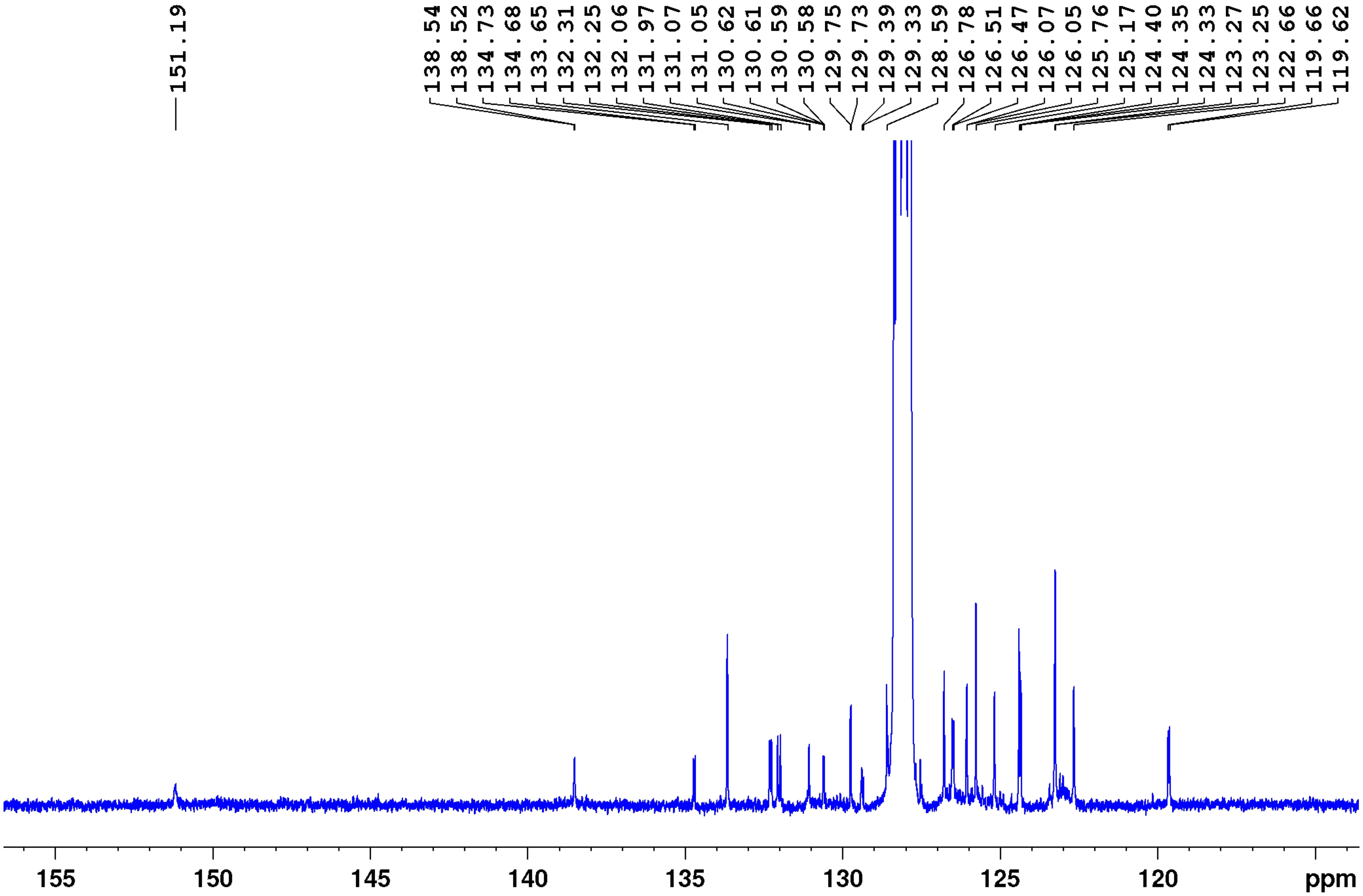

Figure S33. Zoom-in on the aromatic region of the $^{13}C\{^{1}H\}$ NMR spectrum of **8-Phen** in C_6D_6.

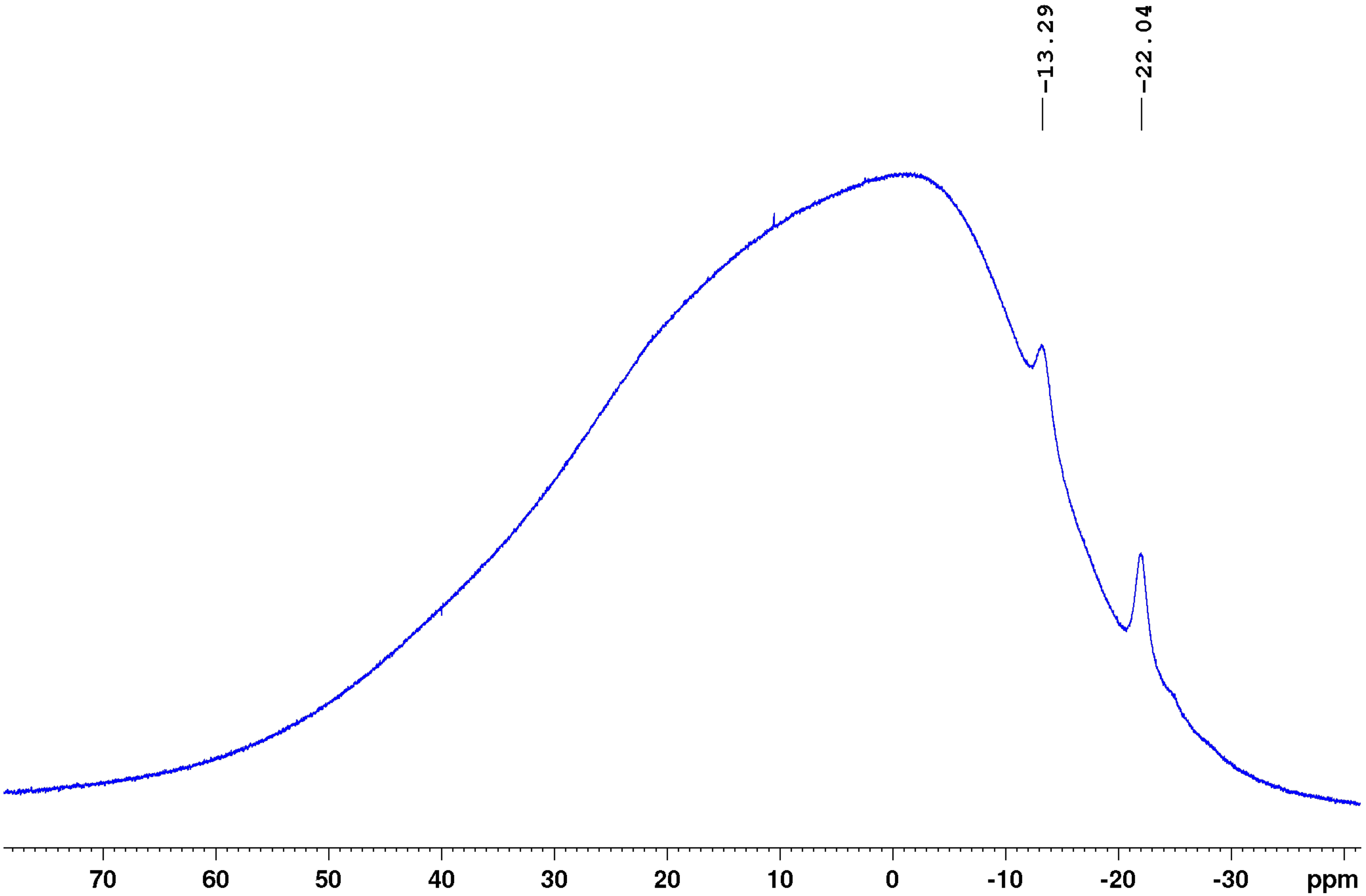

Figure S34. $^{11}B\{^{1}H\}$ NMR spectrum of **8-Phen** in C_6D_6. Small impurities at 11 and –24 ppm are from co-crystallisation of side-products.

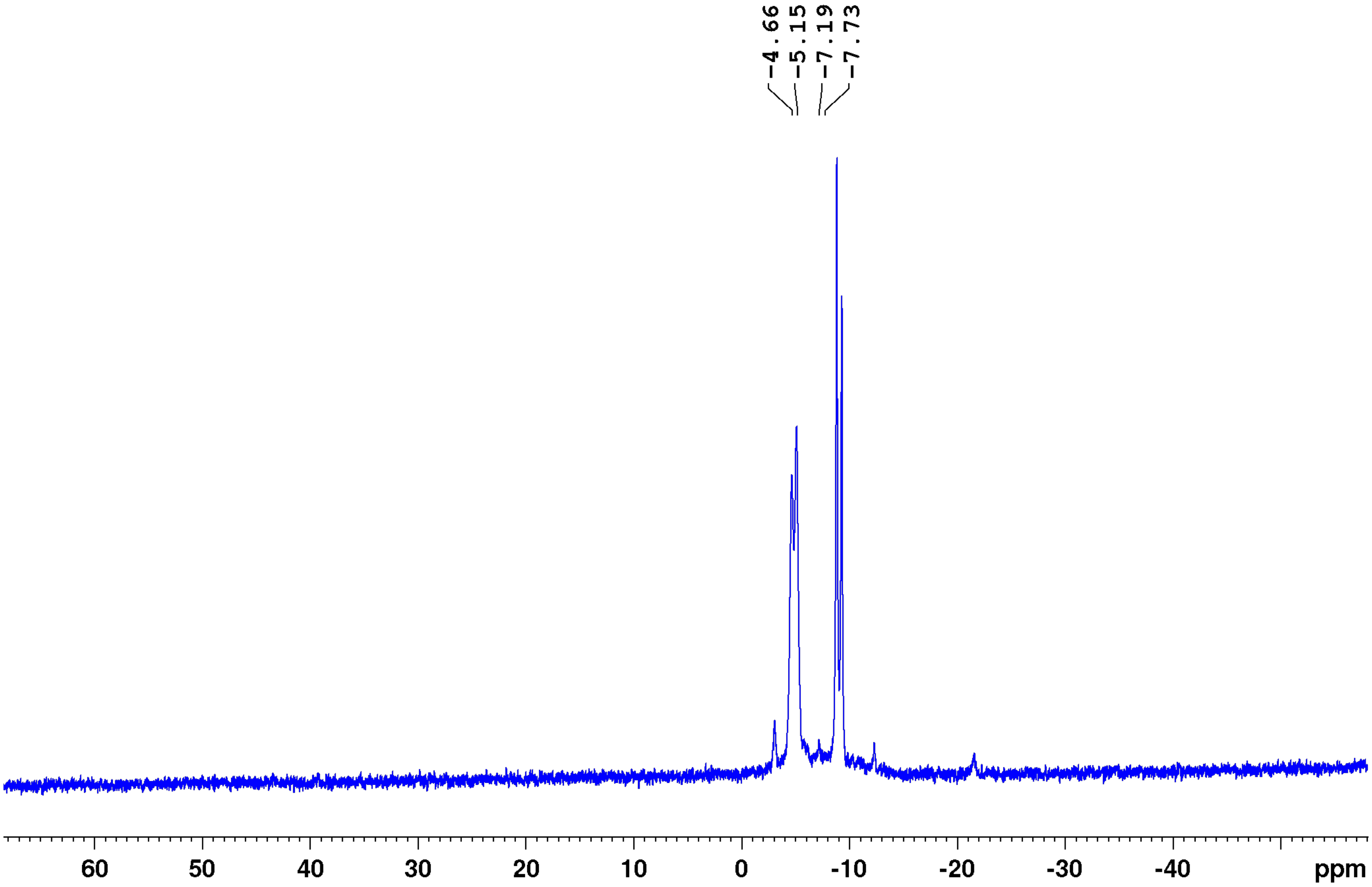

Figure S35. $^{31}P\{^{1}H\}$ NMR spectrum of **8-Phen** in C_6D_6. Small impurities are from co-crystallisation of side-products.

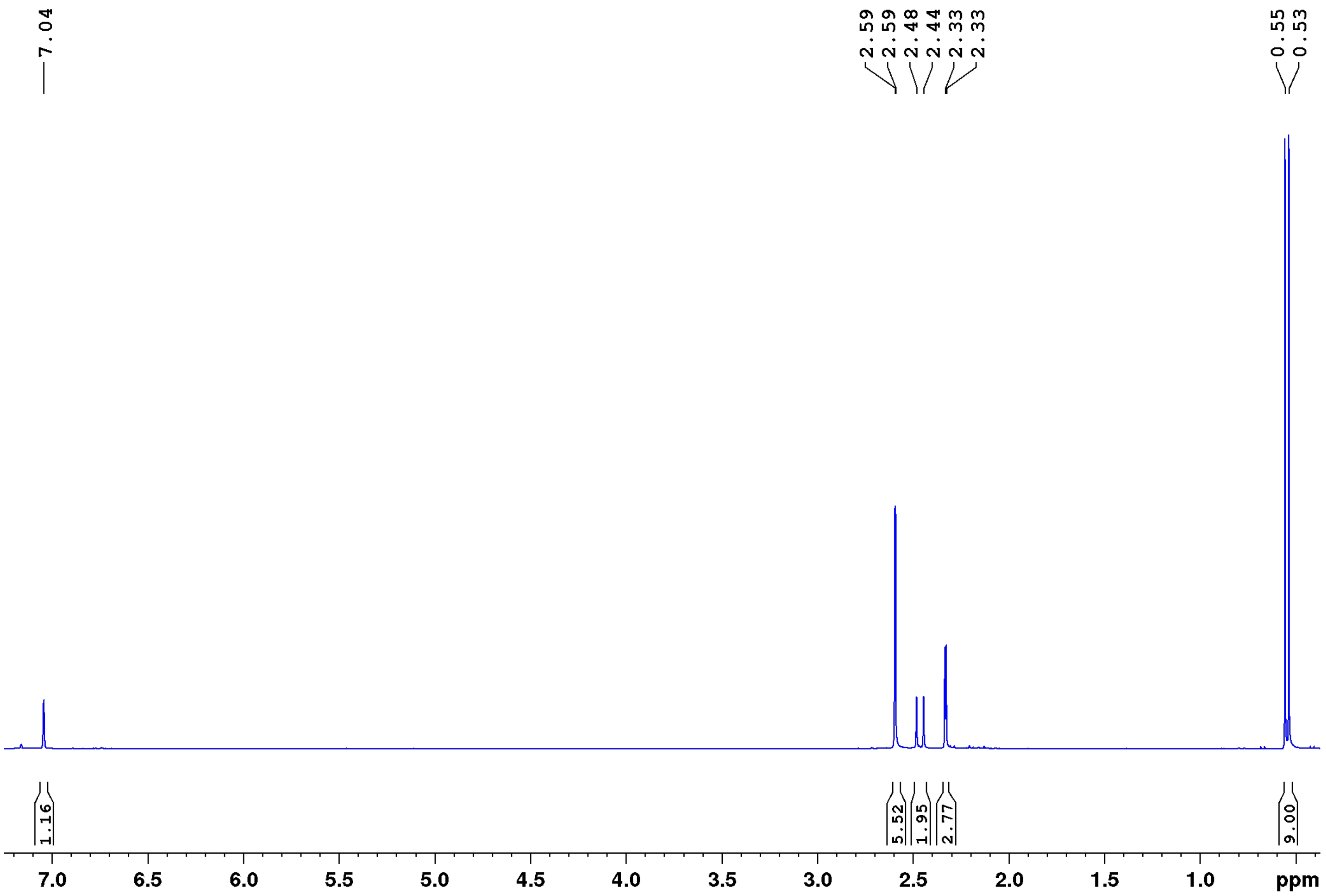

Figure S36. $^{1}H\{^{11}B\}$ NMR spectrum of **10-Mes** in C_6D_6.

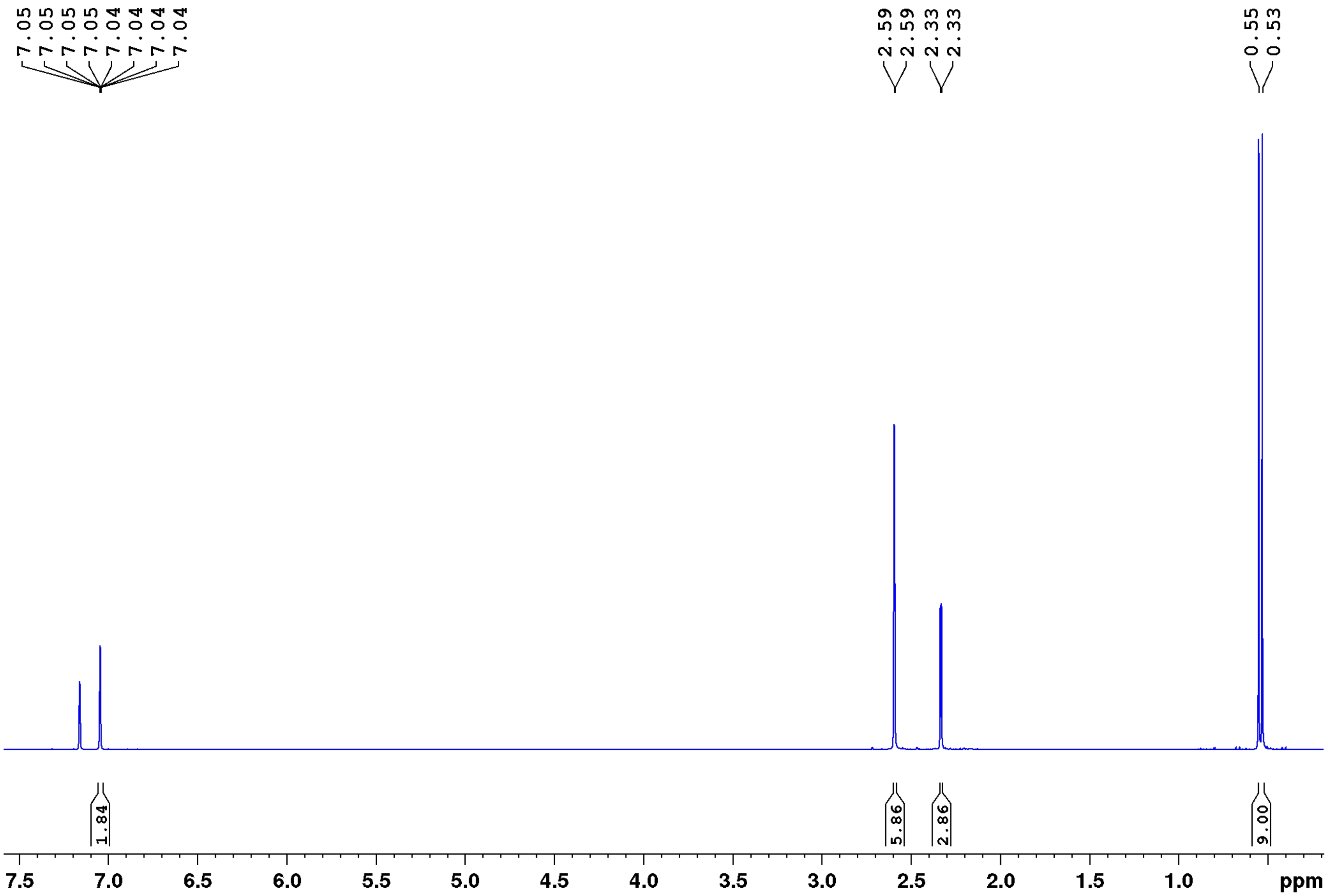

Figure S37. $^{1}H\{^{11}B\}$ NMR spectrum of **10-Mes-D$_2$** in C_6D_6.

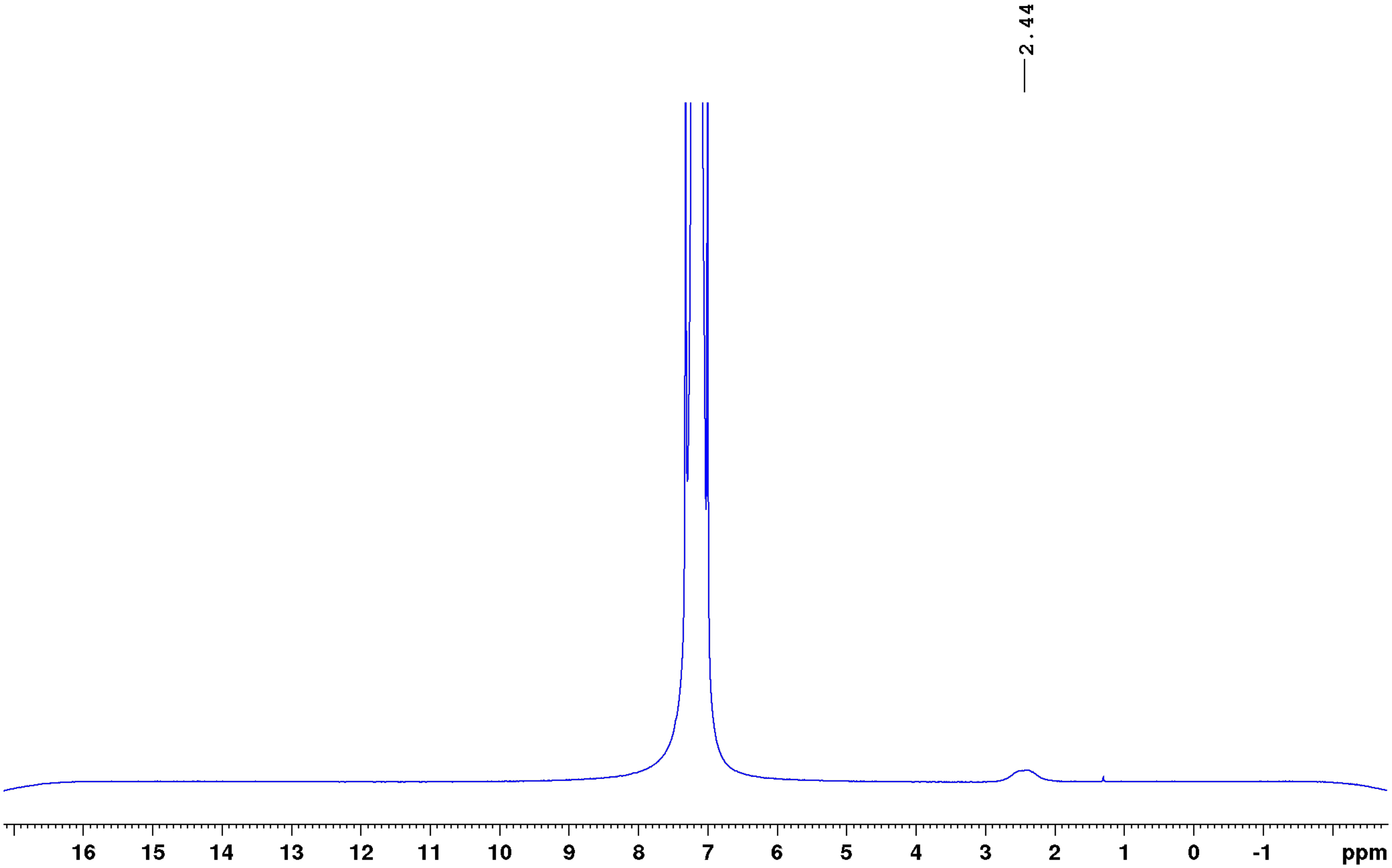

Figure S38. $^{2}H\{^{11}B\}$ NMR spectrum of **10-Mes-D$_2$** in C_6D_6.

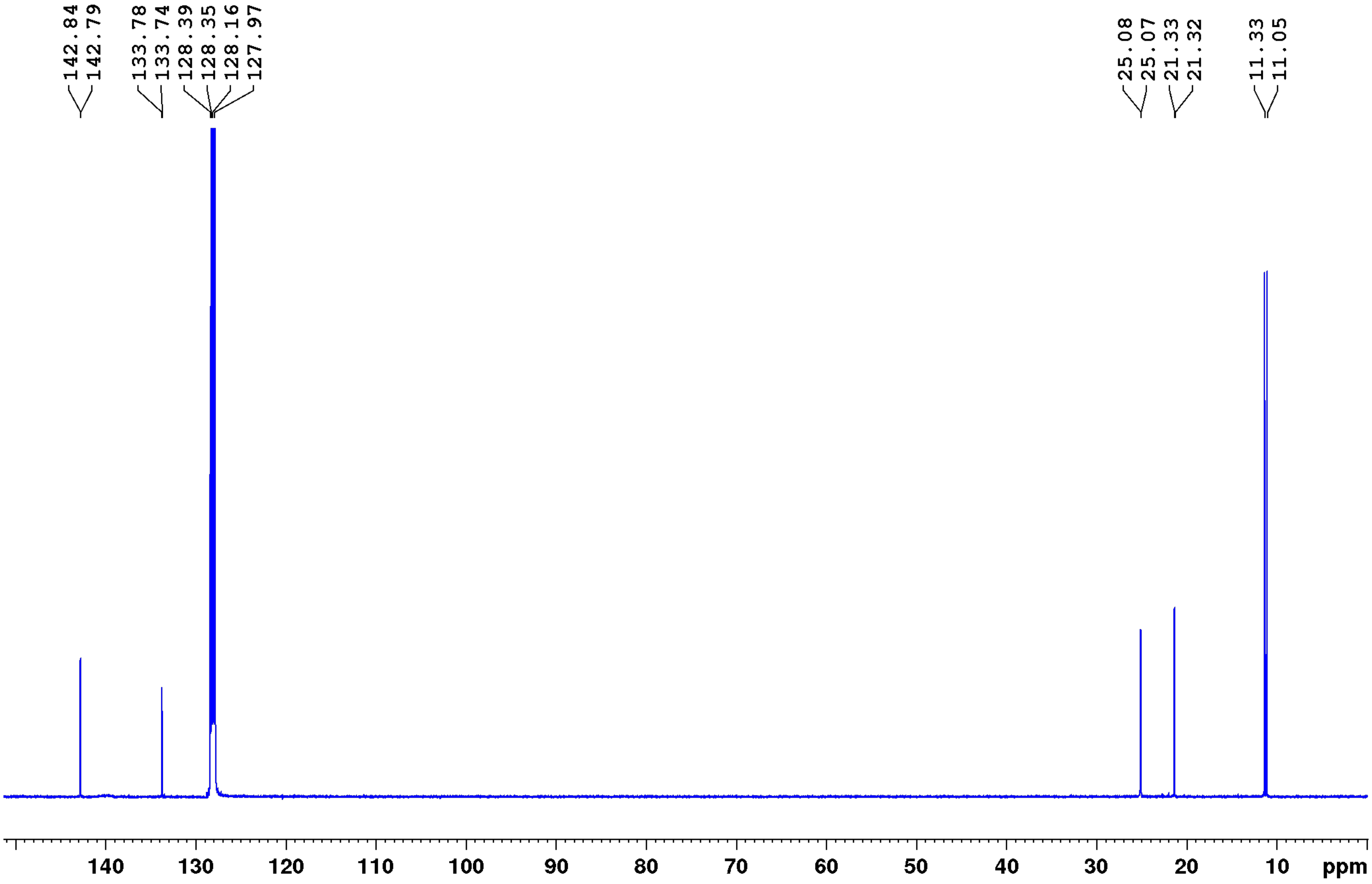

Figure S39. $^{13}C\{^{1}H\}$ NMR spectrum of **10-Mes** in C_6D_6.

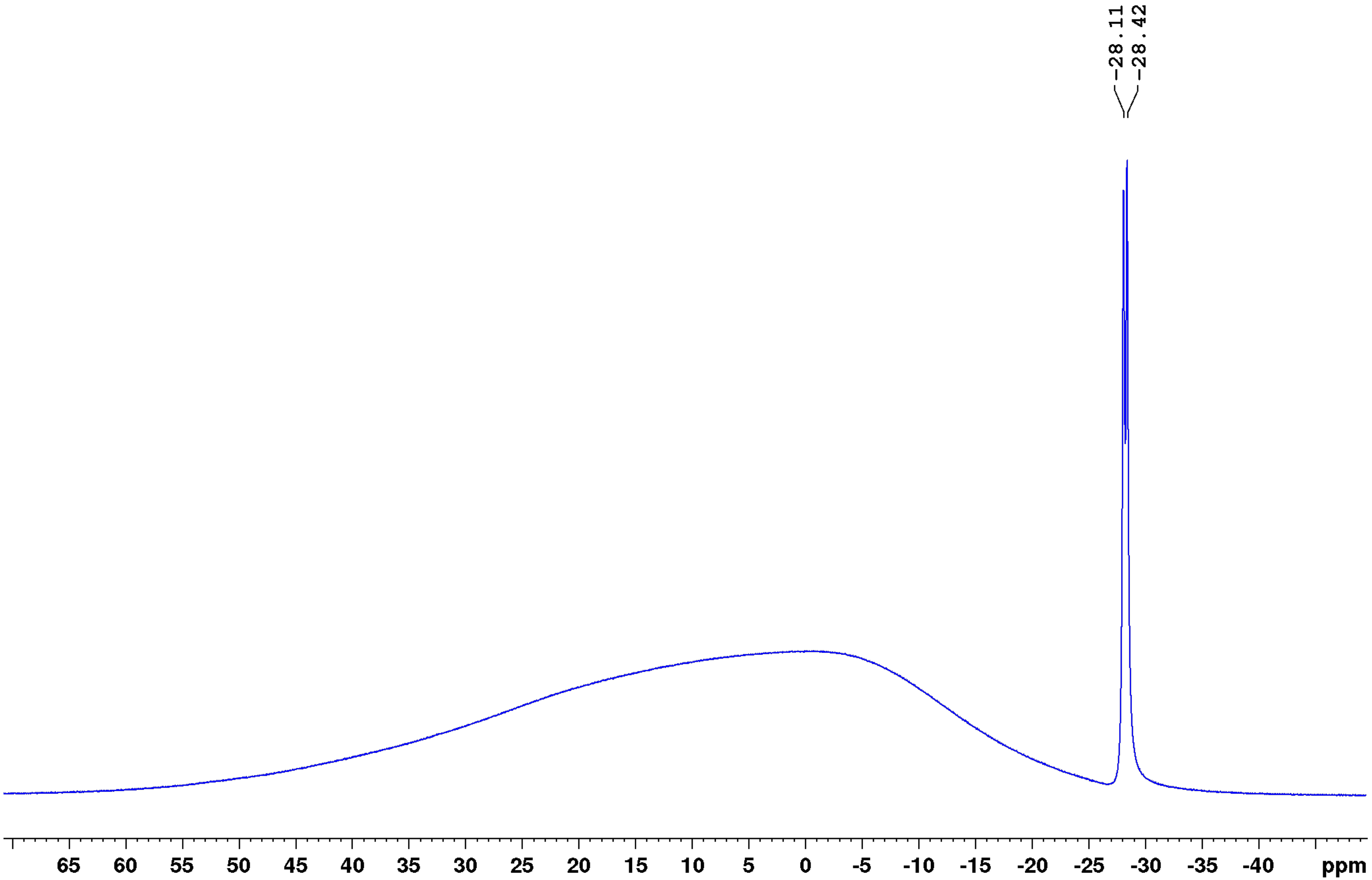

Figure S40. $^{11}B\{^1H\}$ NMR spectrum of **10-Mes** in C_6D_6.

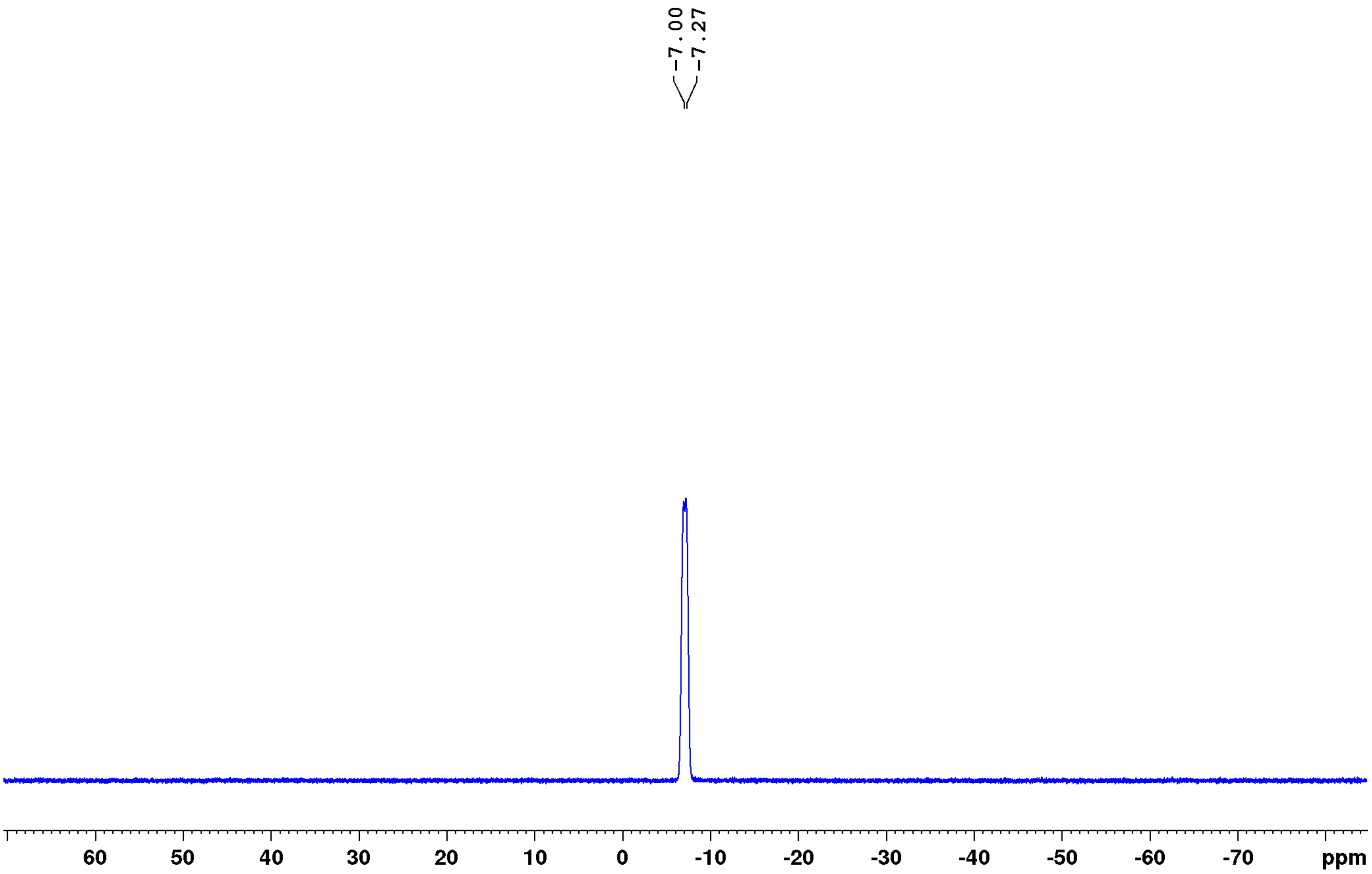

Figure S41. $^{31}P\{^1H\}$ NMR spectrum of **10-Mes** in C_6D_6.

NMR spectra of products which could not be isolated in > 90% purity.

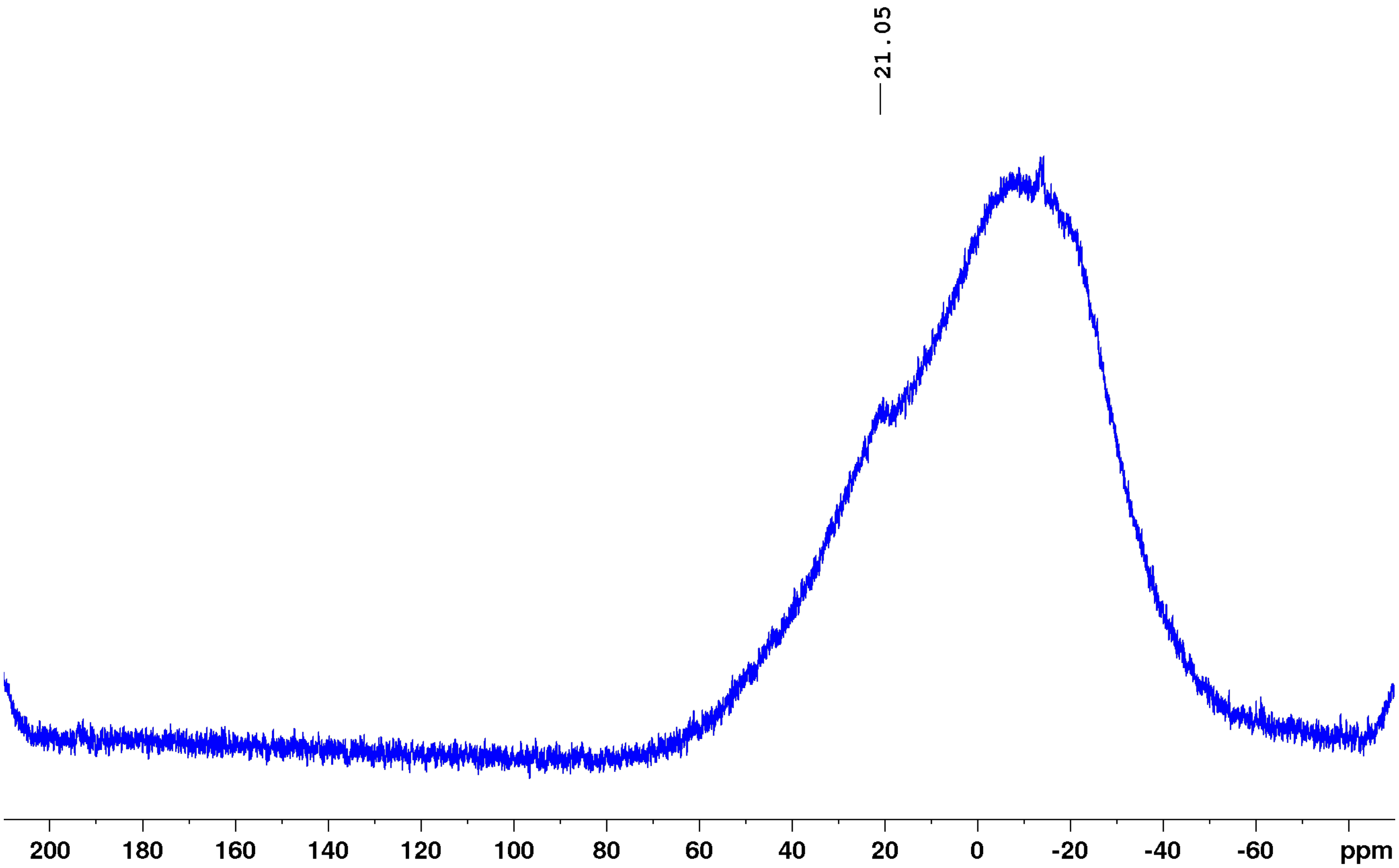

Figure S42. ^{11}B NMR spectrum of crude **7-Pyr** in C_6D_6 (ca. 80% purity). The impurity at –13 ppm could neither be removed nor identified.

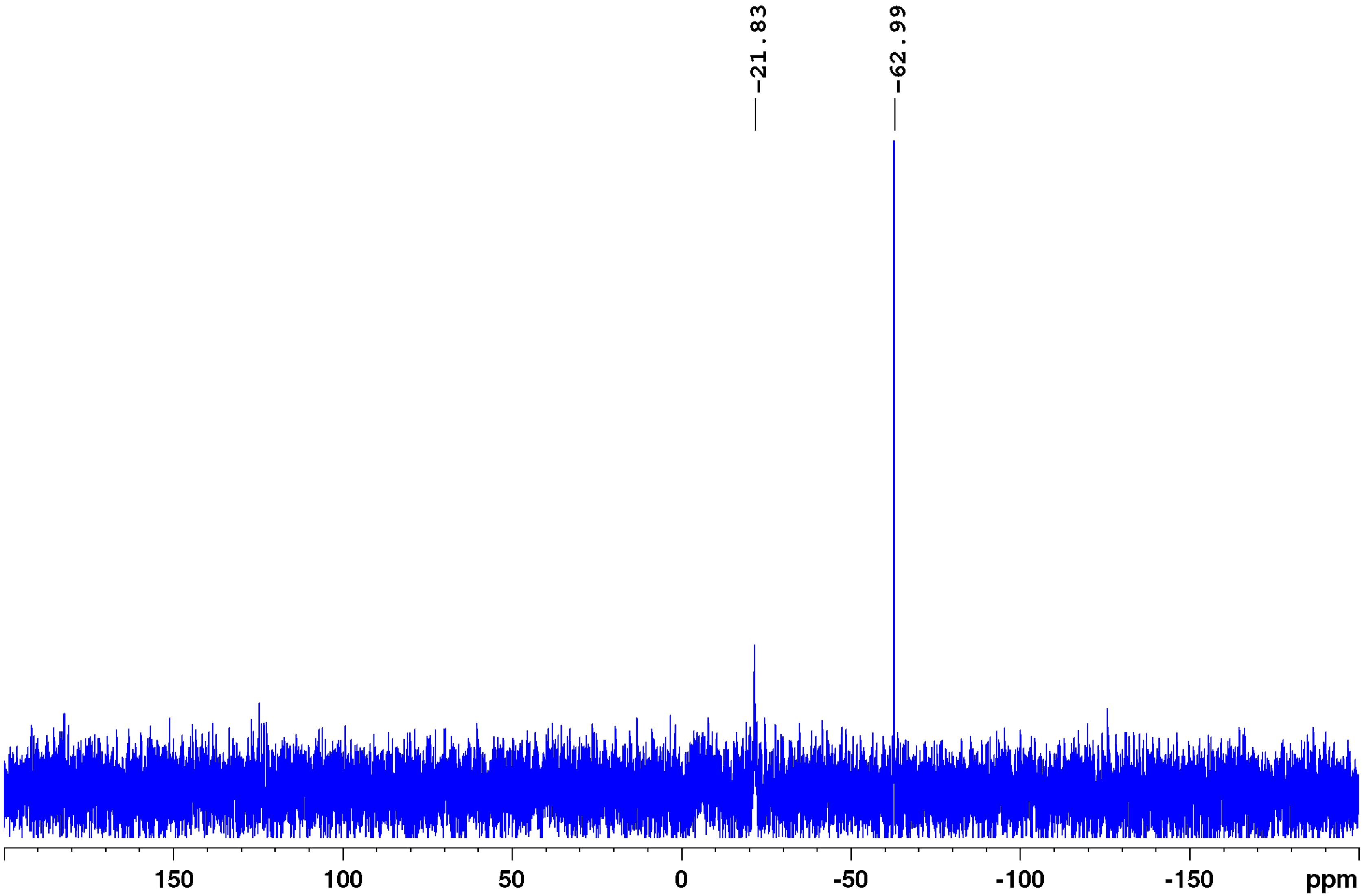

Figure S43. $^{31}P\{^1H\}$ NMR spectrum of **7-Pyr** in C_6D_6. The additional singlet at –62.9 ppm corresponds to residual PMe_3, without which **9-Pyr** is not stable.

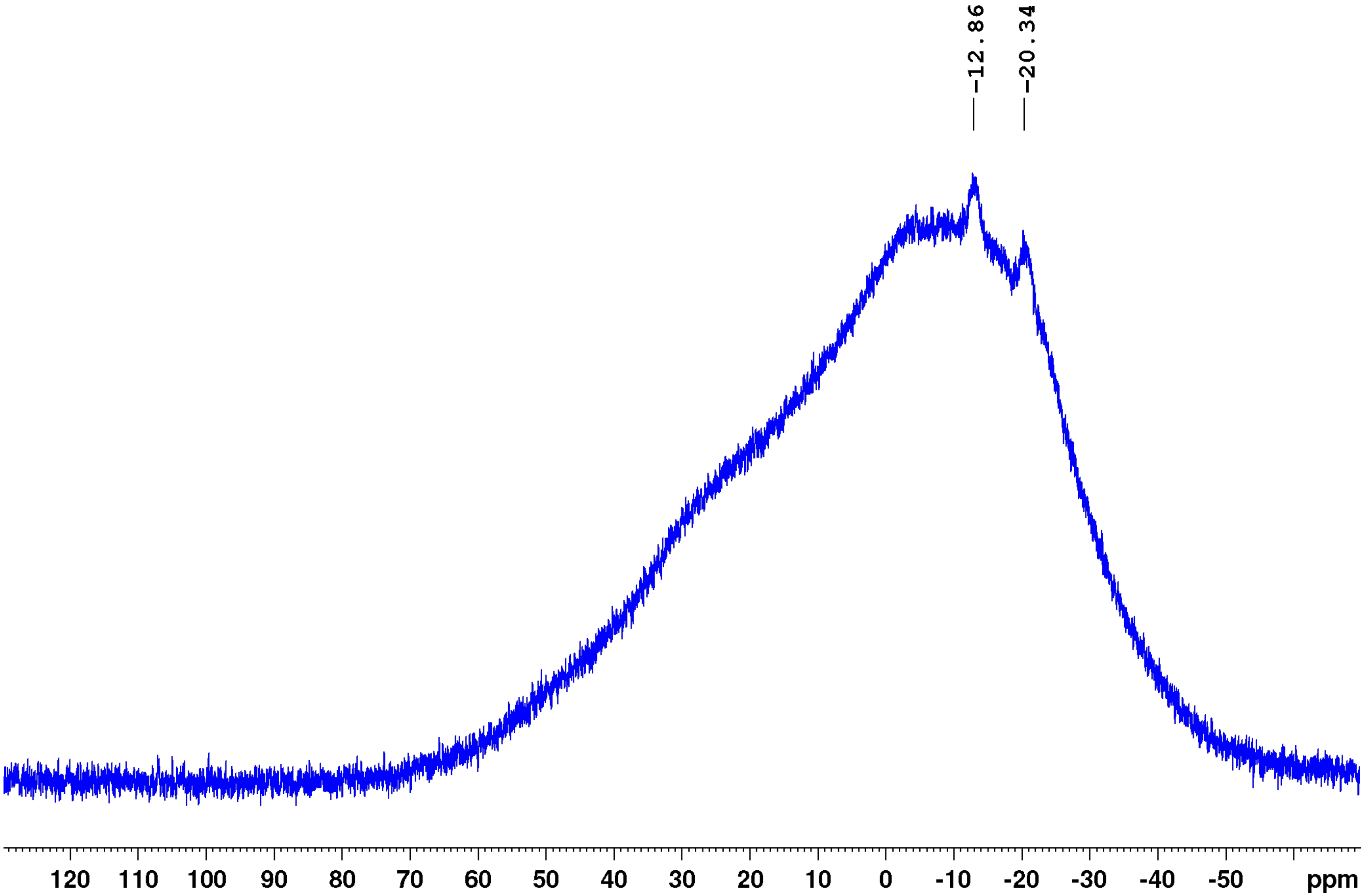

Figure S44. $^{11}B\{^1H\}$ NMR spectrum of **8-Pyr** in C_6D_6 (ca. 80% purity).

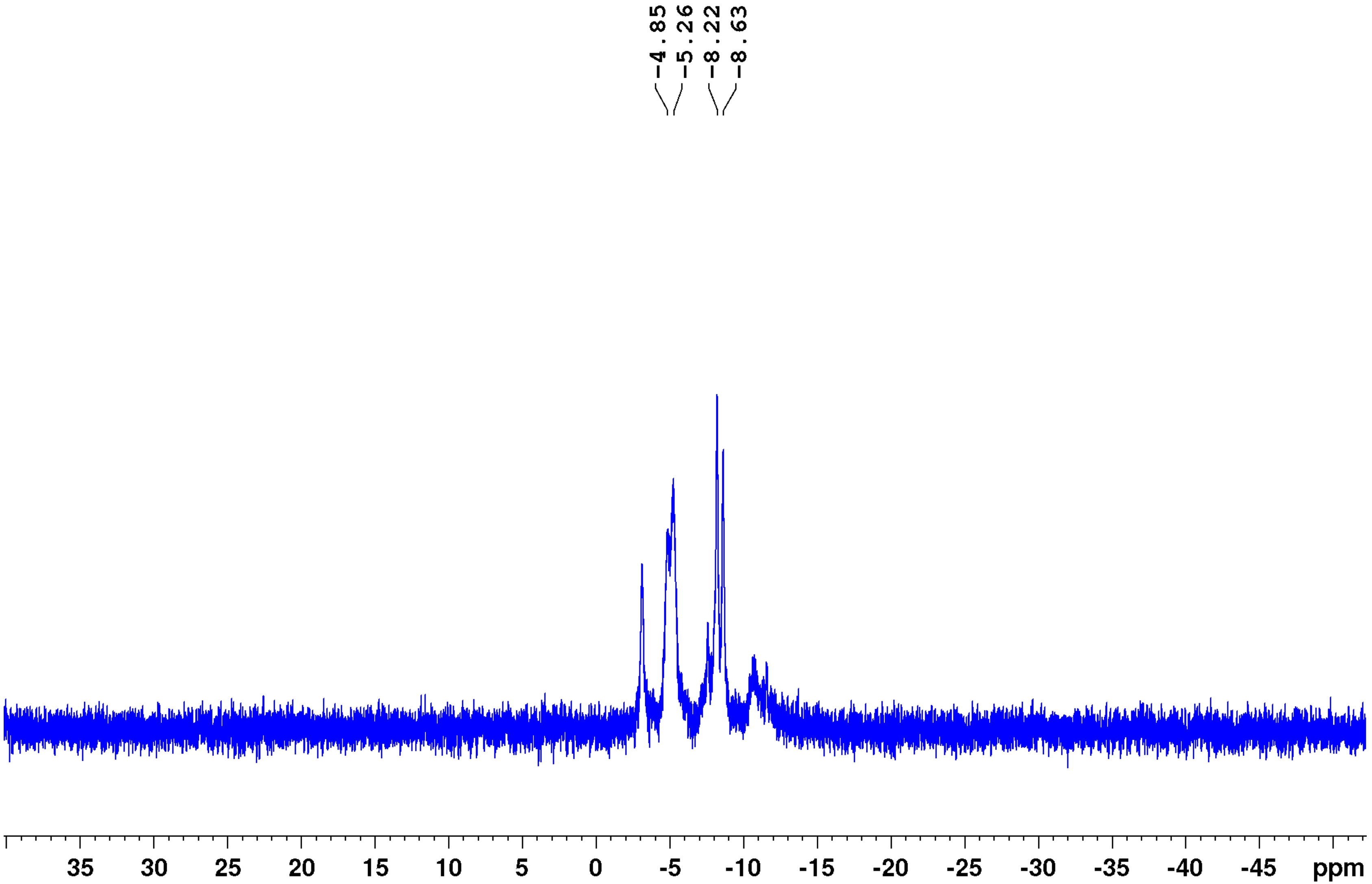

Figure S45. $^{31}P\{^1H\}$ NMR spectrum of **8-Pyr** in C_6D_6 (ca. 80% purity). The impurities at –3, –7.5 and –10.5 ppm could neither be removed nor identified.

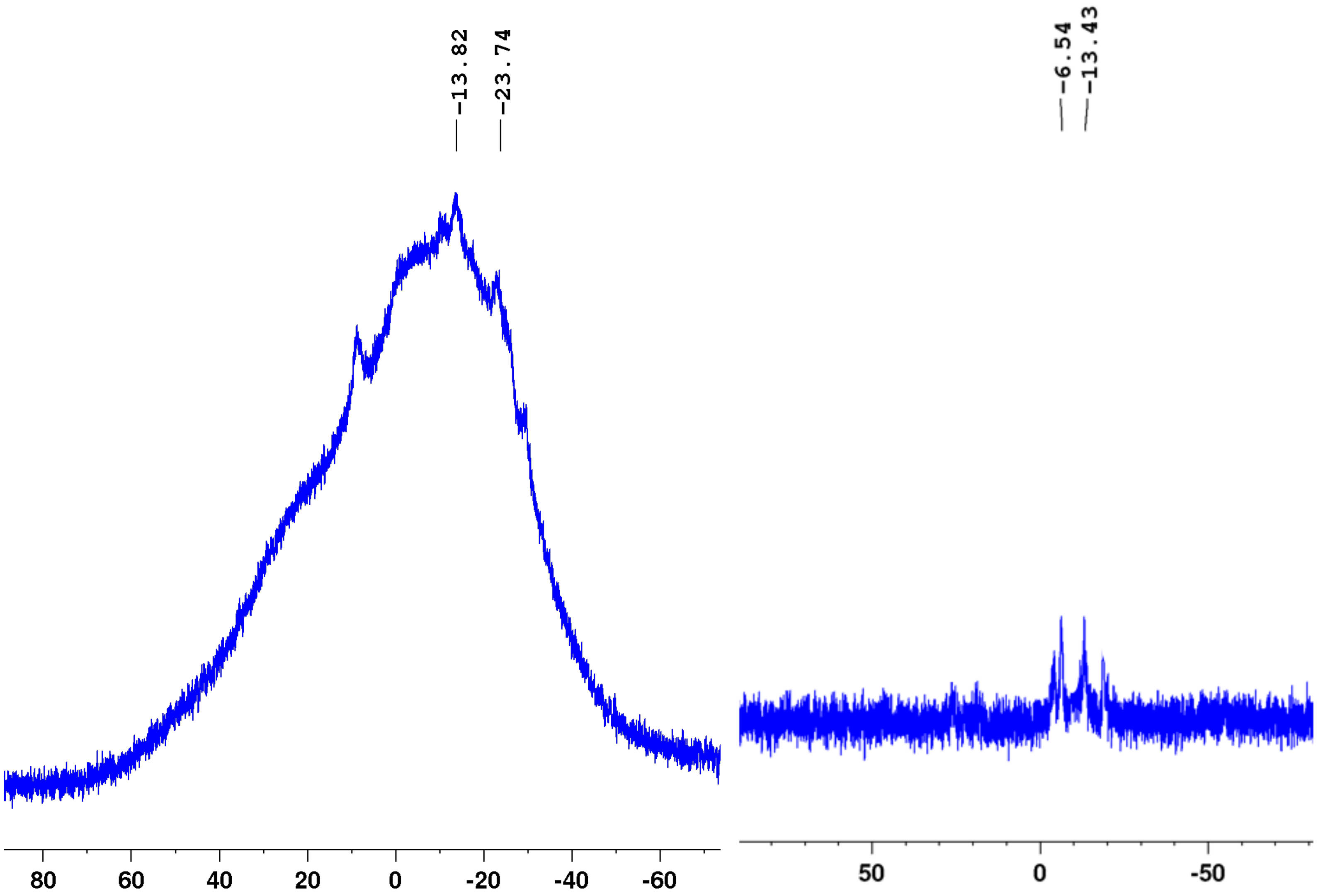

Figure S46. $^{11}B\{^1H\}$ (left) and $^{31}P\{^1H\}$ (right) NMR spectra of crude **8-Anth** in C_6D_6. The impurities at δ_{11B} = 18 and –30 ppm and δ_{31P} = –4 and –20 ppm could neither be removed nor identified.

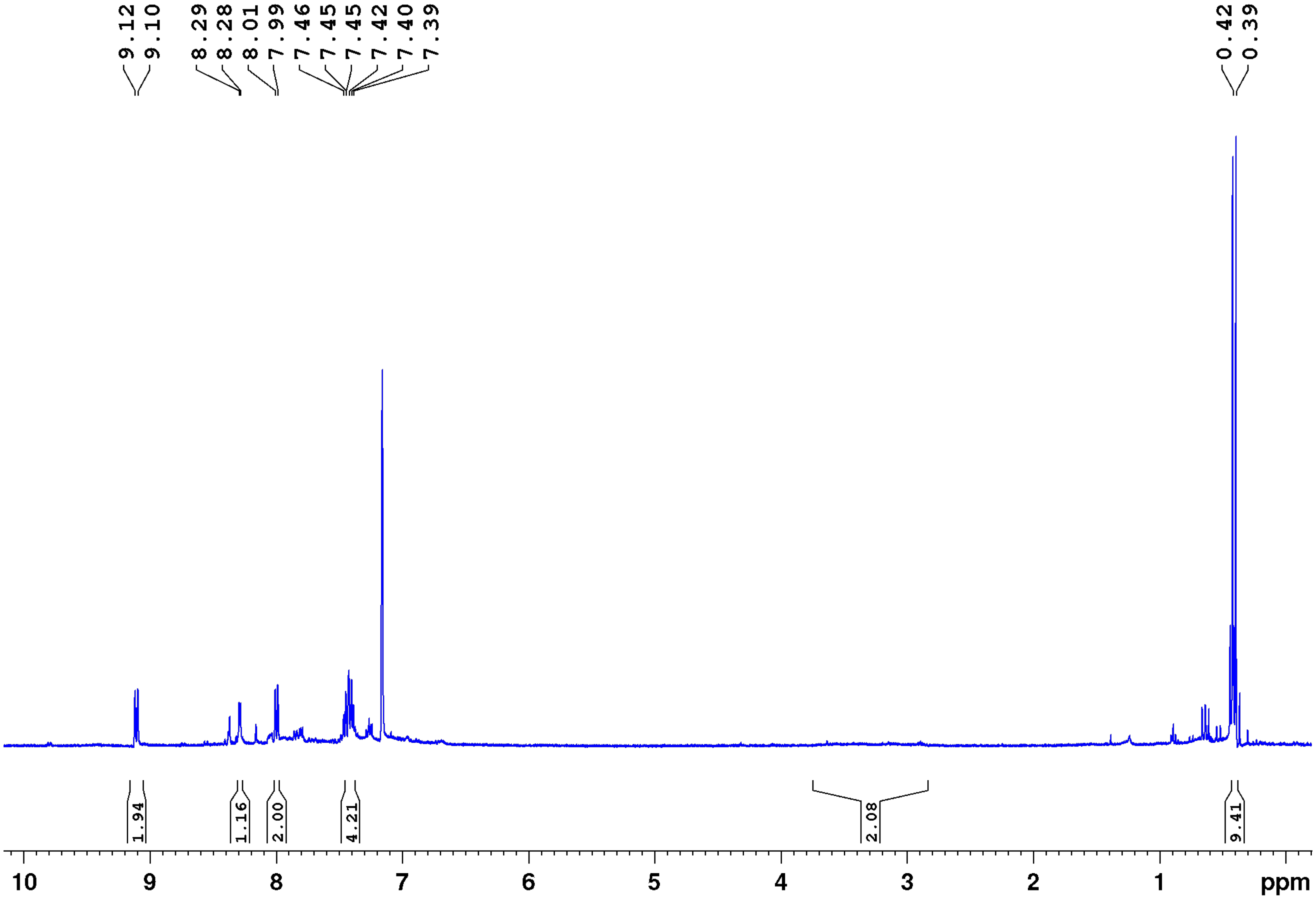

Figure S47. $^{1}H\{^{11}B\}$ NMR spectrum of crude **10-Anth** in C_6D_6 (ca. 70% purity). The impurities could neither be removed nor identified.

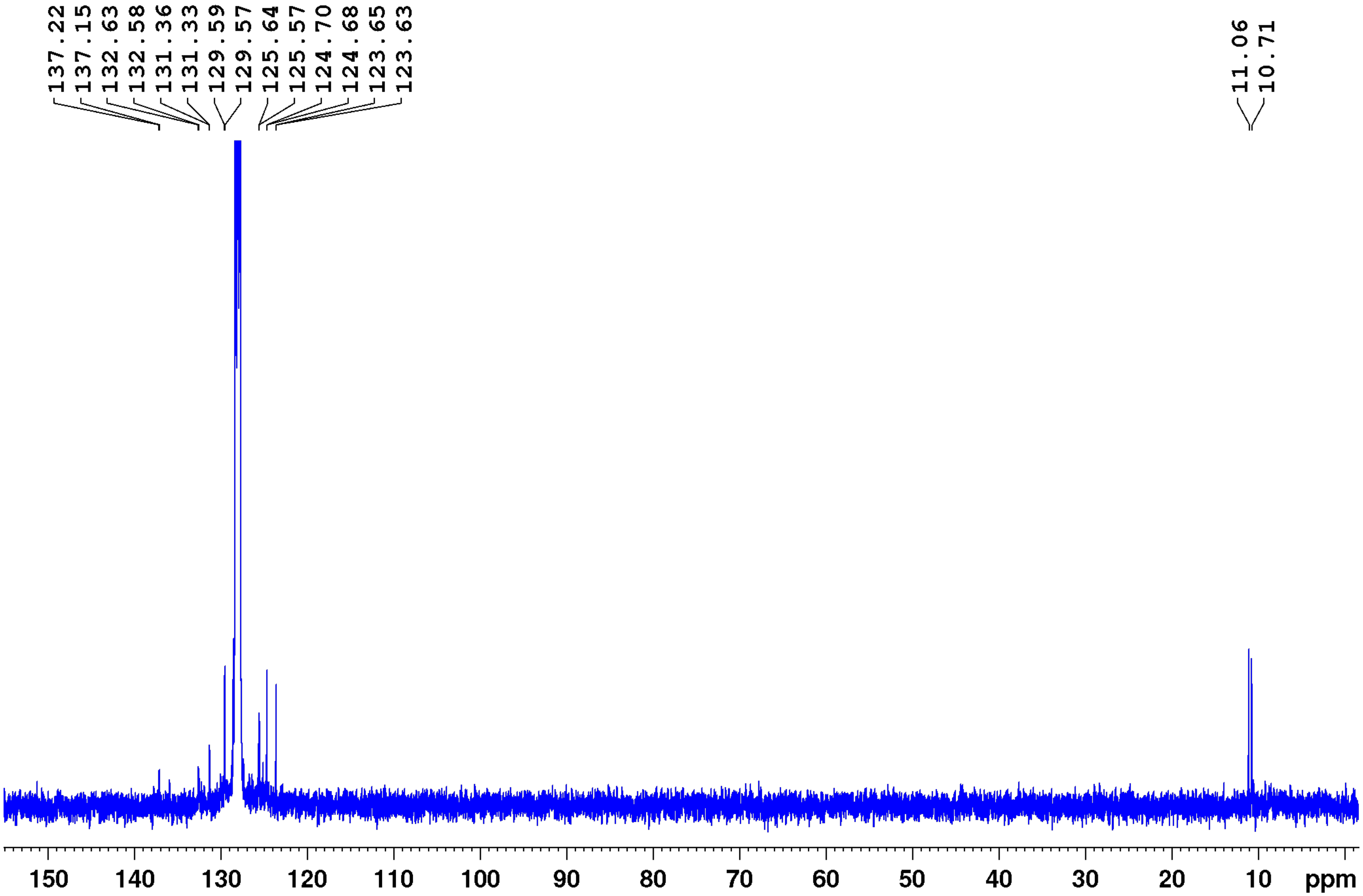

Figure S48. $^{13}C\{^{1}H\}$ NMR spectrum of crude **10-Anth** in C_6D_6 (ca. 70% purity).

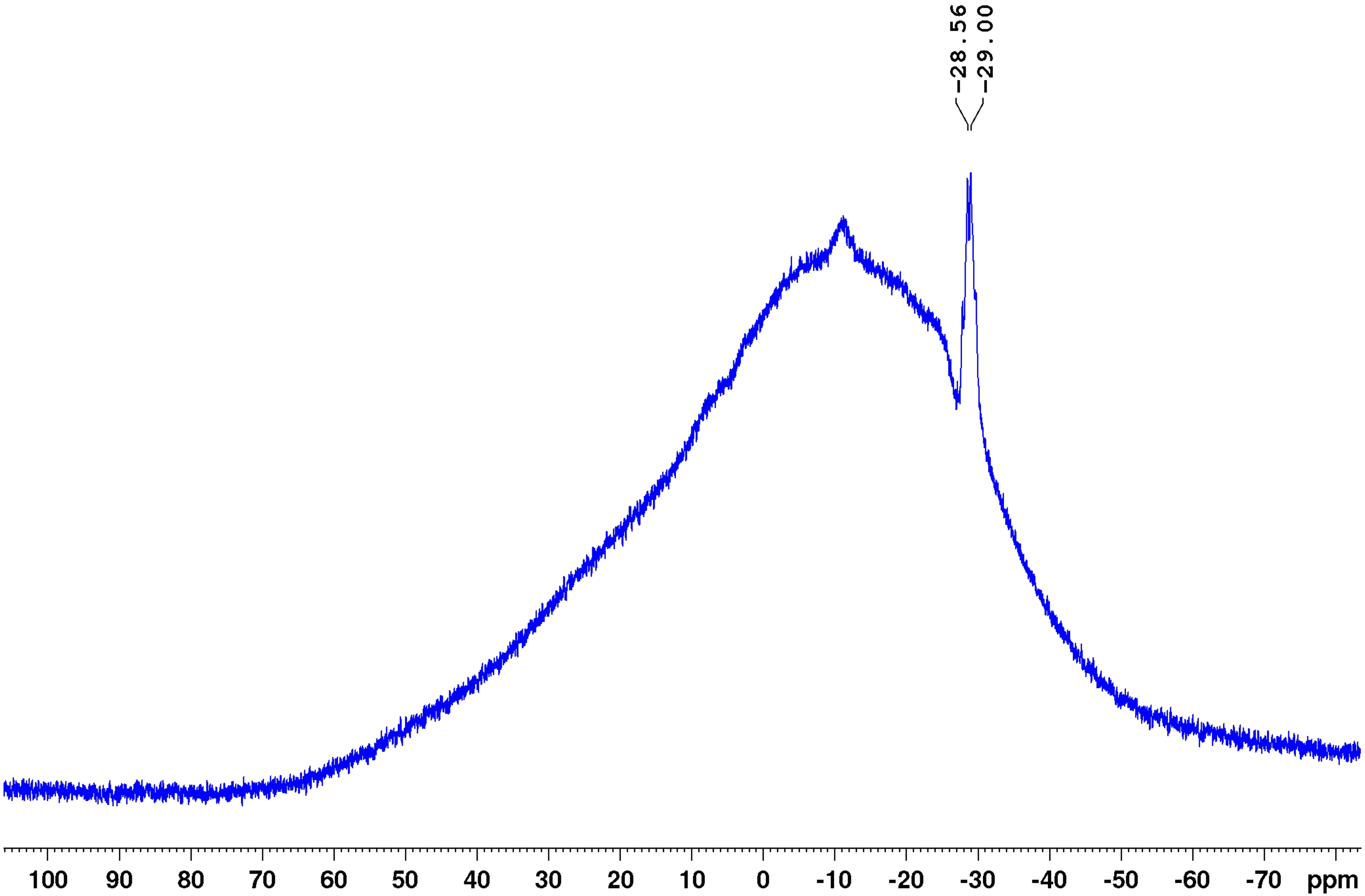

Figure S49. $^{11}B\{^{1}H\}$ NMR spectrum of **10-Anth** in C_6D_6 (ca. 70% purity). The impurity at –11 ppm could neither be removed nor identified.

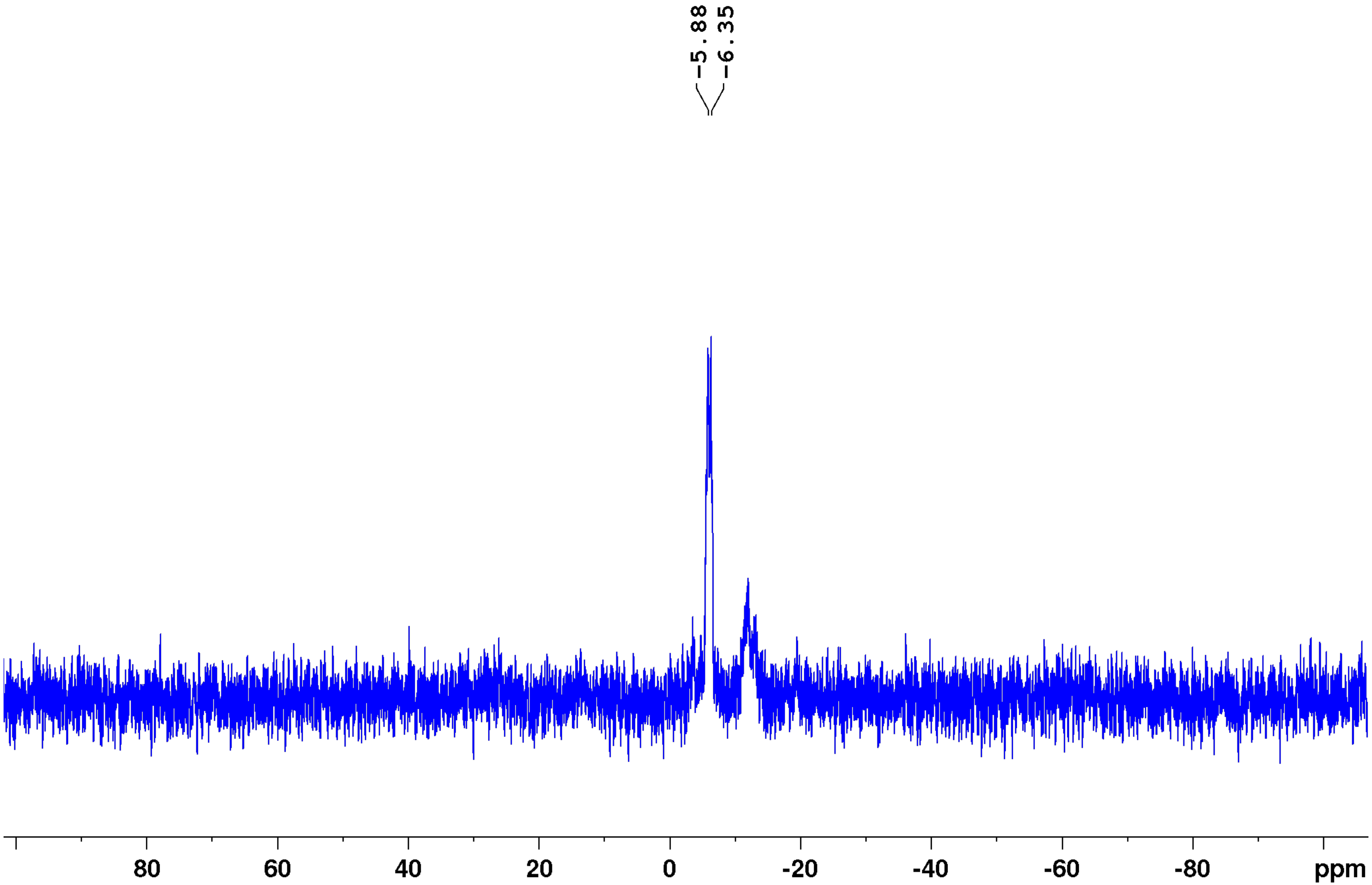

Figure S50. $^{31}P\{^1H\}$ NMR spectrum of **10-Anth** in C_6D_6 (ca. 70% purity). The impurity at –12 ppm could neither be removed nor identified.

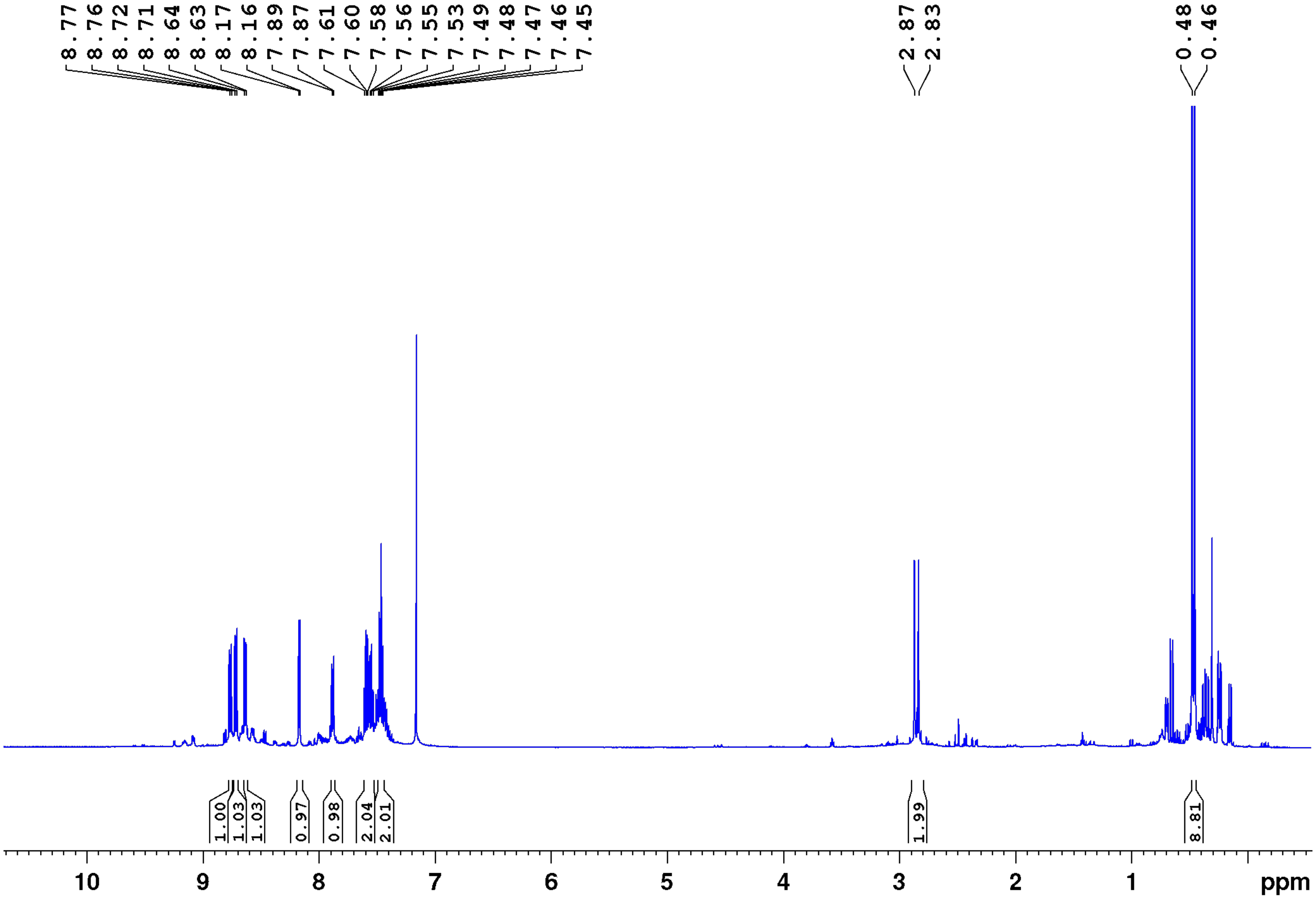

Figure S51. $^{1}H\{^{11}B\}$ NMR spectrum of **10-Phen** (ca. 80% purity) in C_6D_6. The impurities could neither be removed nor identified.

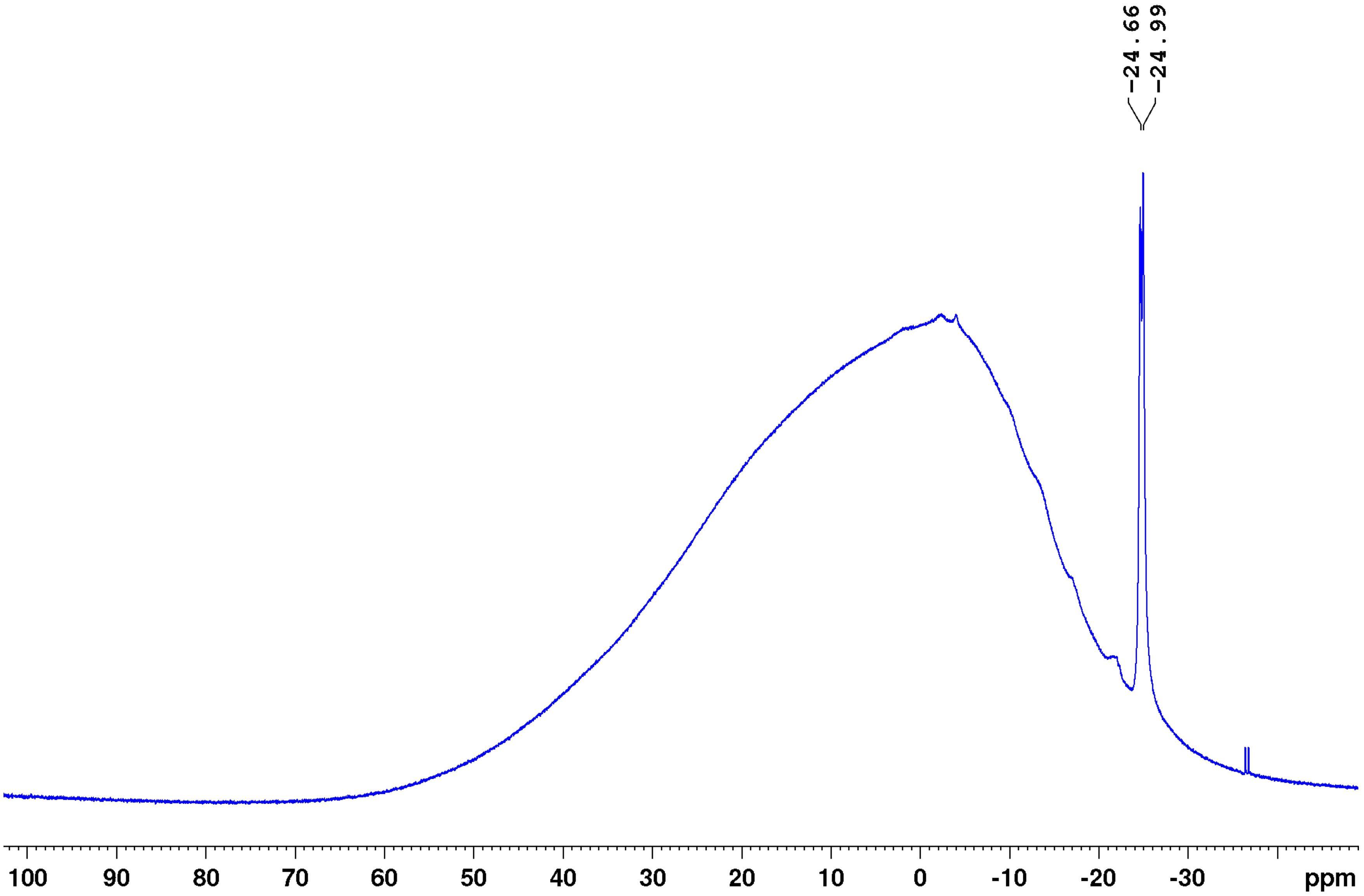

Figure S52. $^{11}B\{^1H\}$ NMR spectrum of **10-Phen** (ca. 80% purity) in C_6D_6. The impurities could neither be removed nor identified.

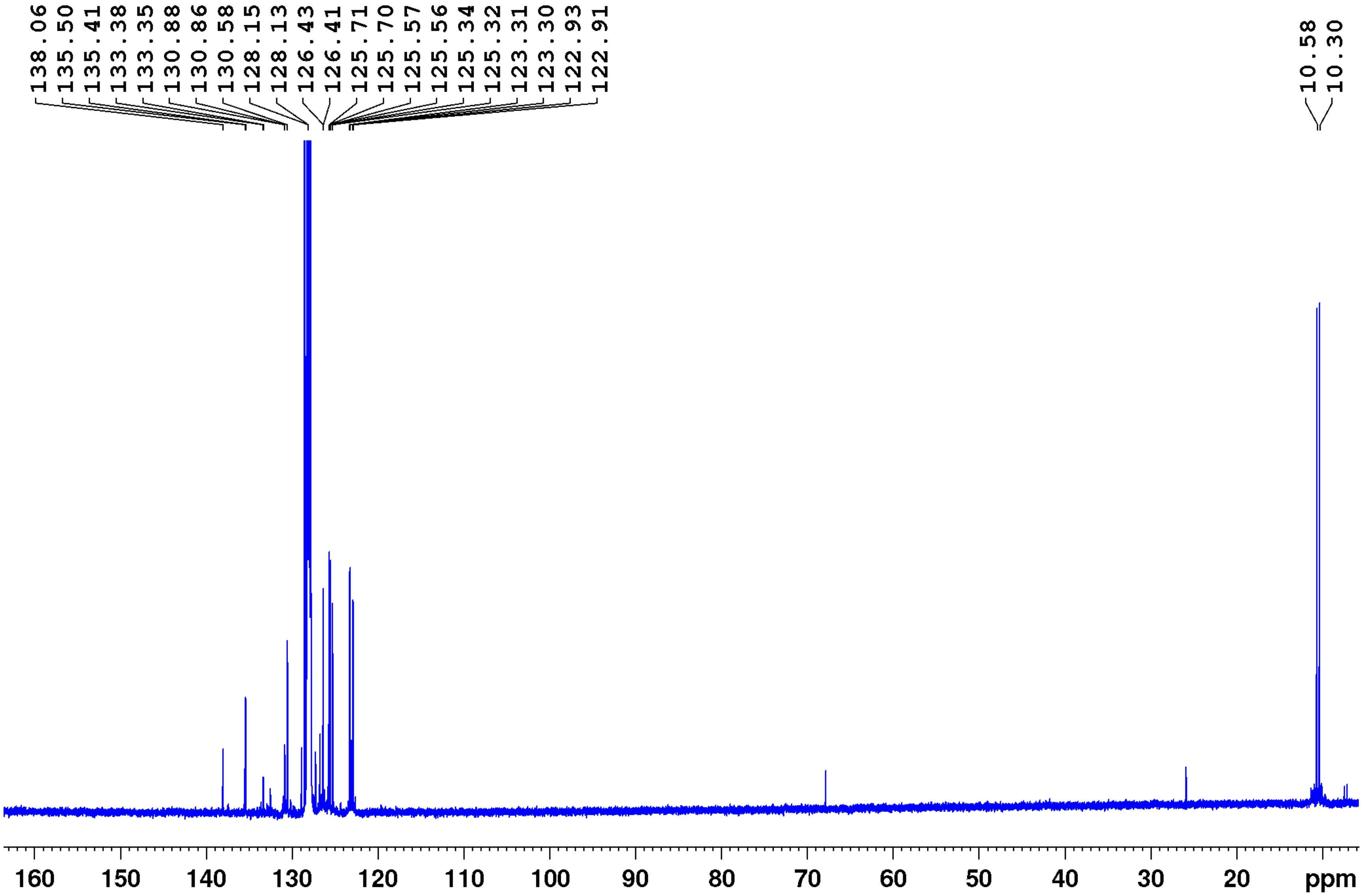

Figure S53. $^{13}C\{^{1}H\}$ NMR spectrum of **10-Phen** (ca. 80% purity) in C_6D_6.

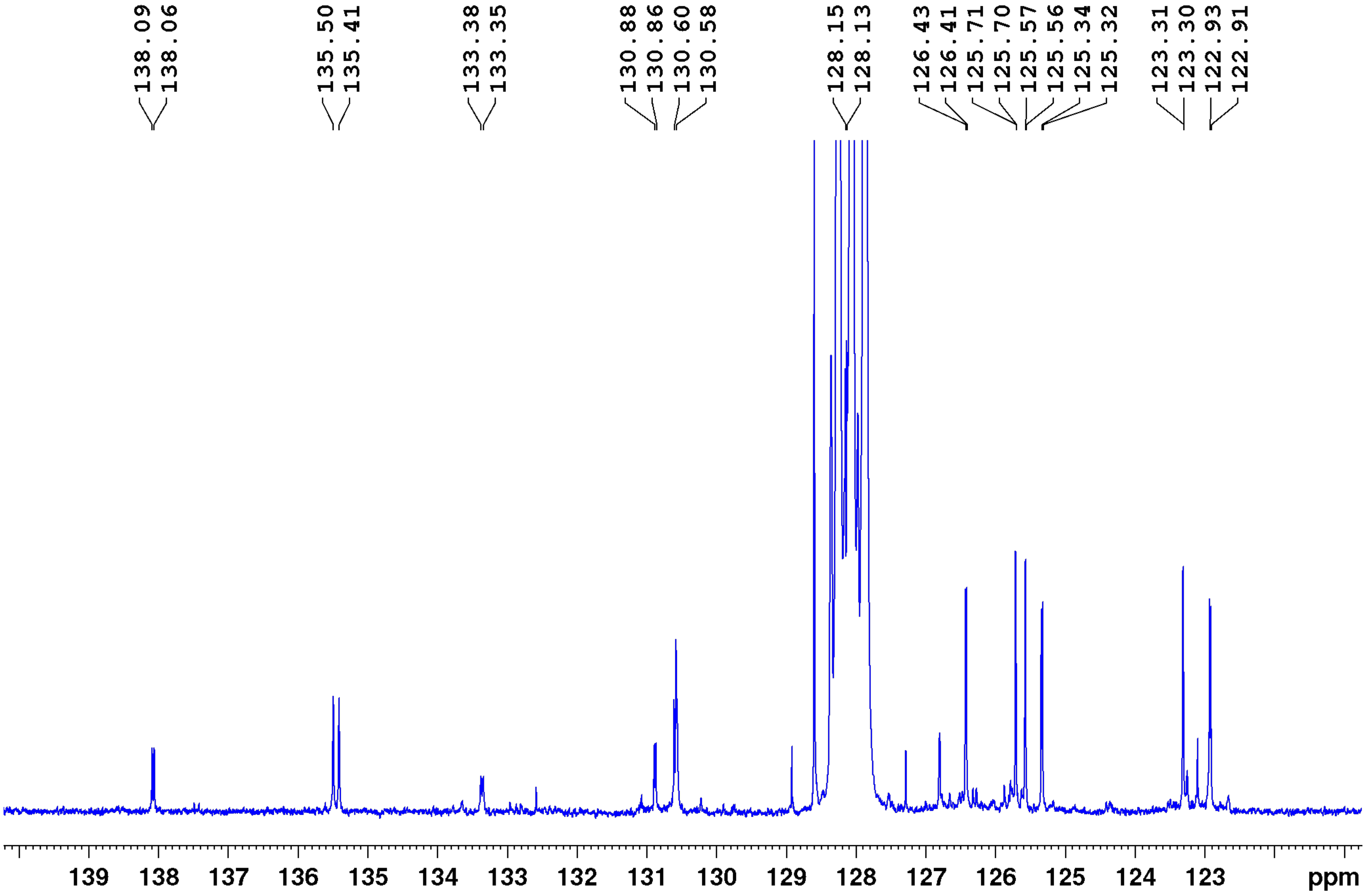

Figure S54. Zoom-in on the aromatic region of the $^{13}C\{^{1}H\}$ spectrum of **10-Phen** in C_6D_6.

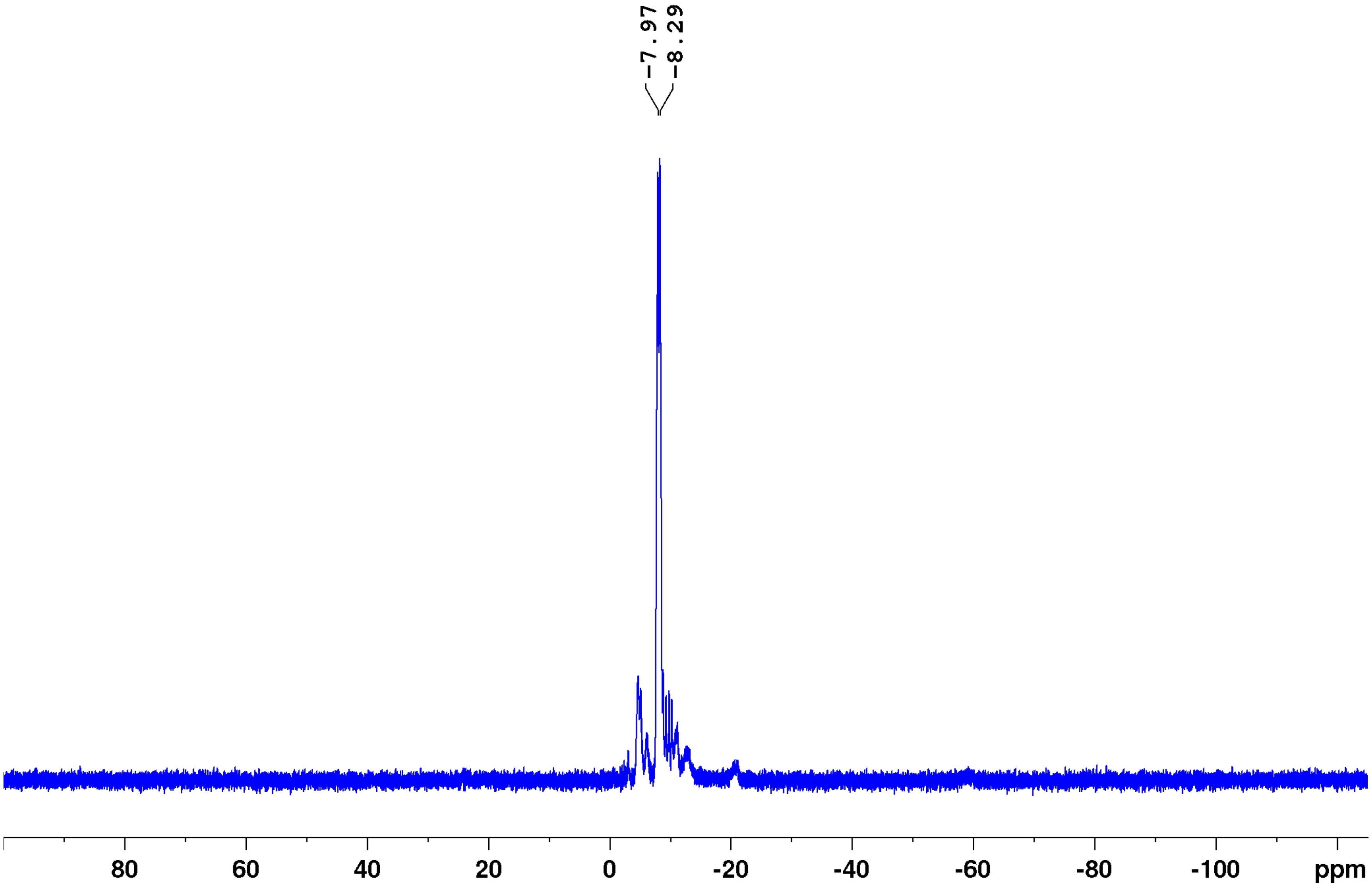

Figure S55. $^{31}P\{^{1}H\}$ NMR spectrum of **10-Phen** (ca. 80% purity) in C_6D_6. The impurities could neither be removed nor identified.

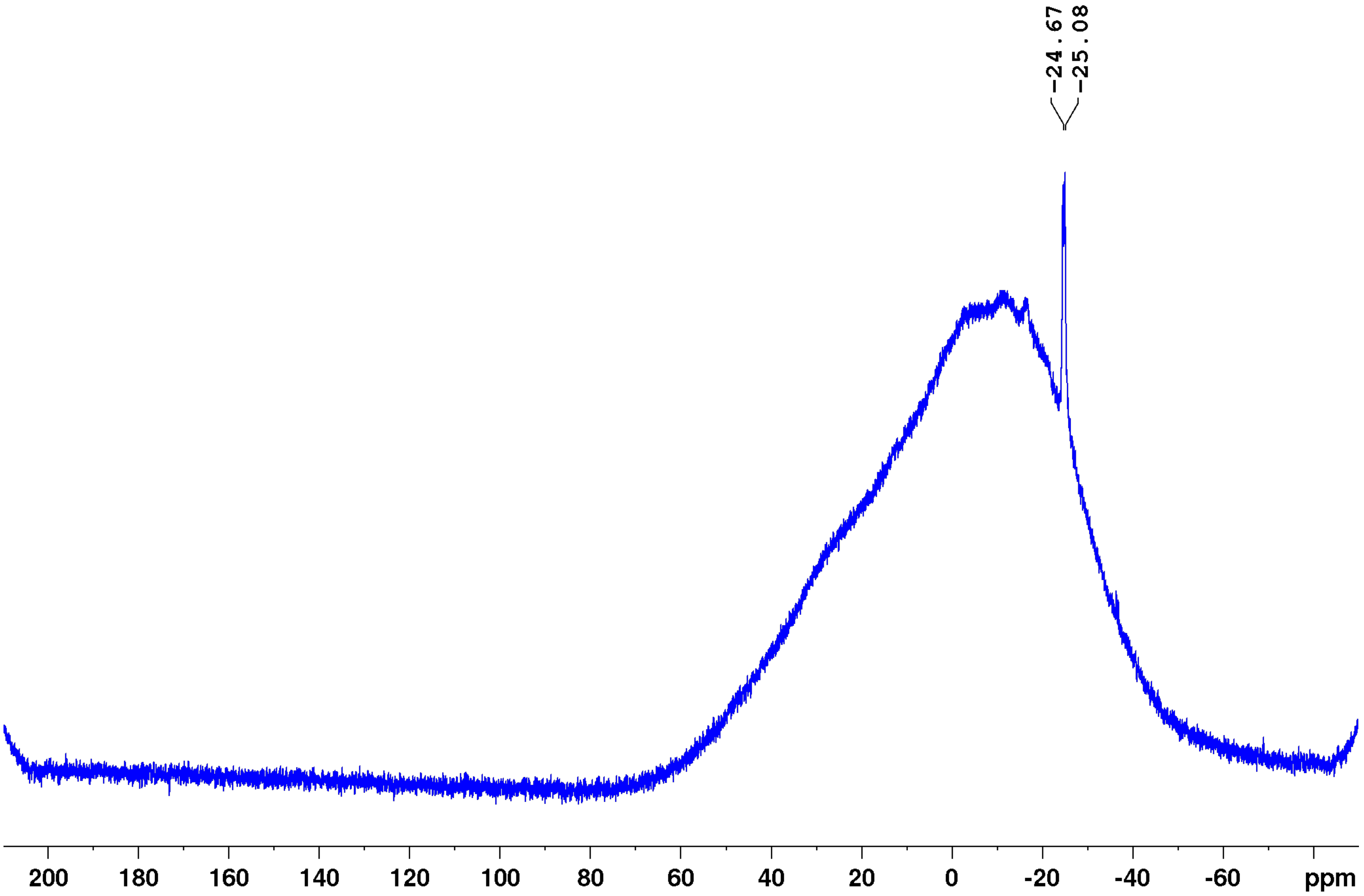

Figure S56. $^{11}B\{^1H\}$ NMR spectrum of **10-Pyr** in C_6D_6 (ca. 70% purity). The impurities could neither be removed nor identified.

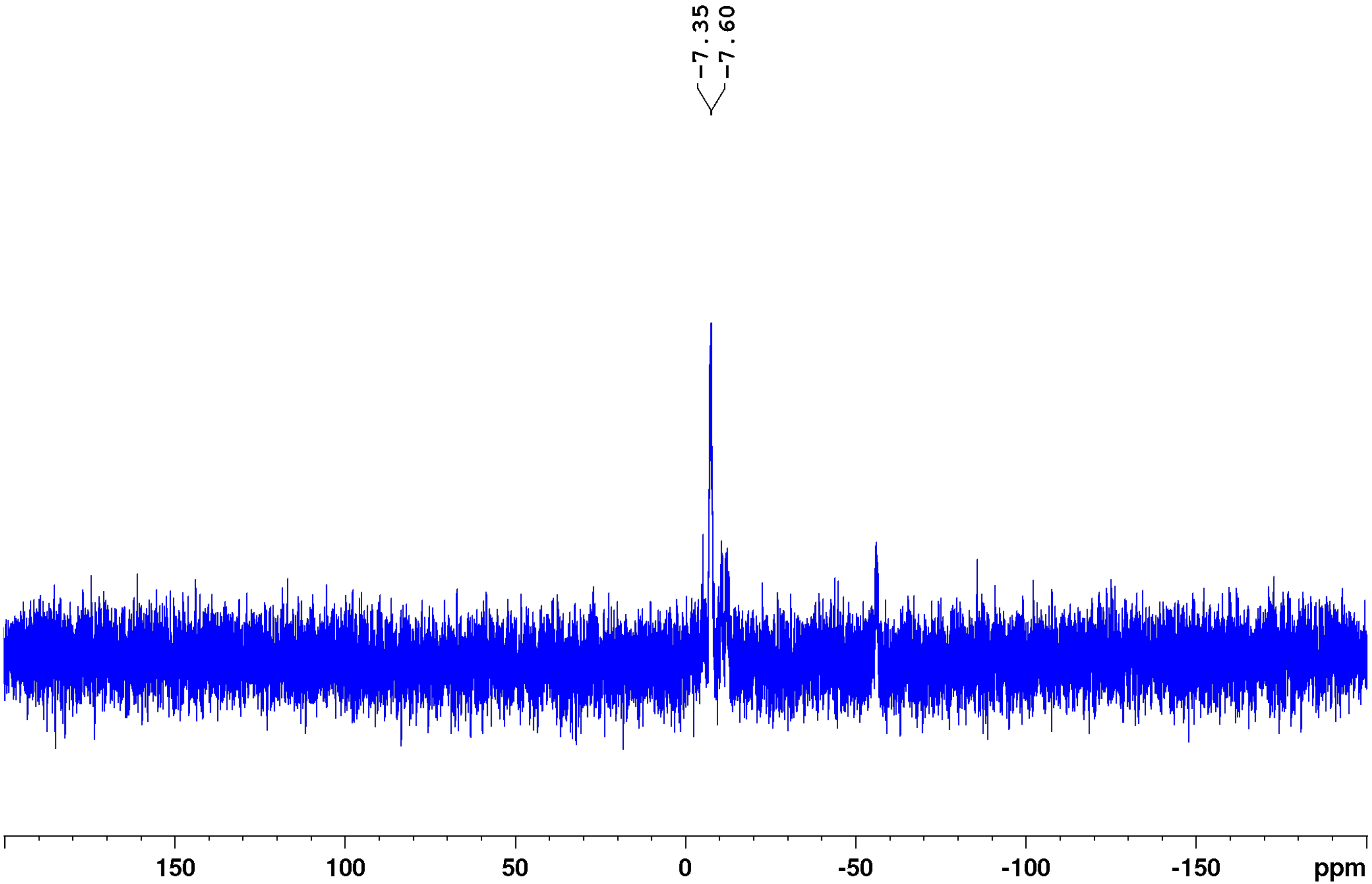

Figure S57. $^{31}P\{^1H\}$ NMR spectrum of **10-Pyr** in C_6D_6 (ca. 70% purity). The impurities could neither be removed nor identified.

^{11}B NMR spectra of the attempted syntheses of 2-Phen and 2-Pyr

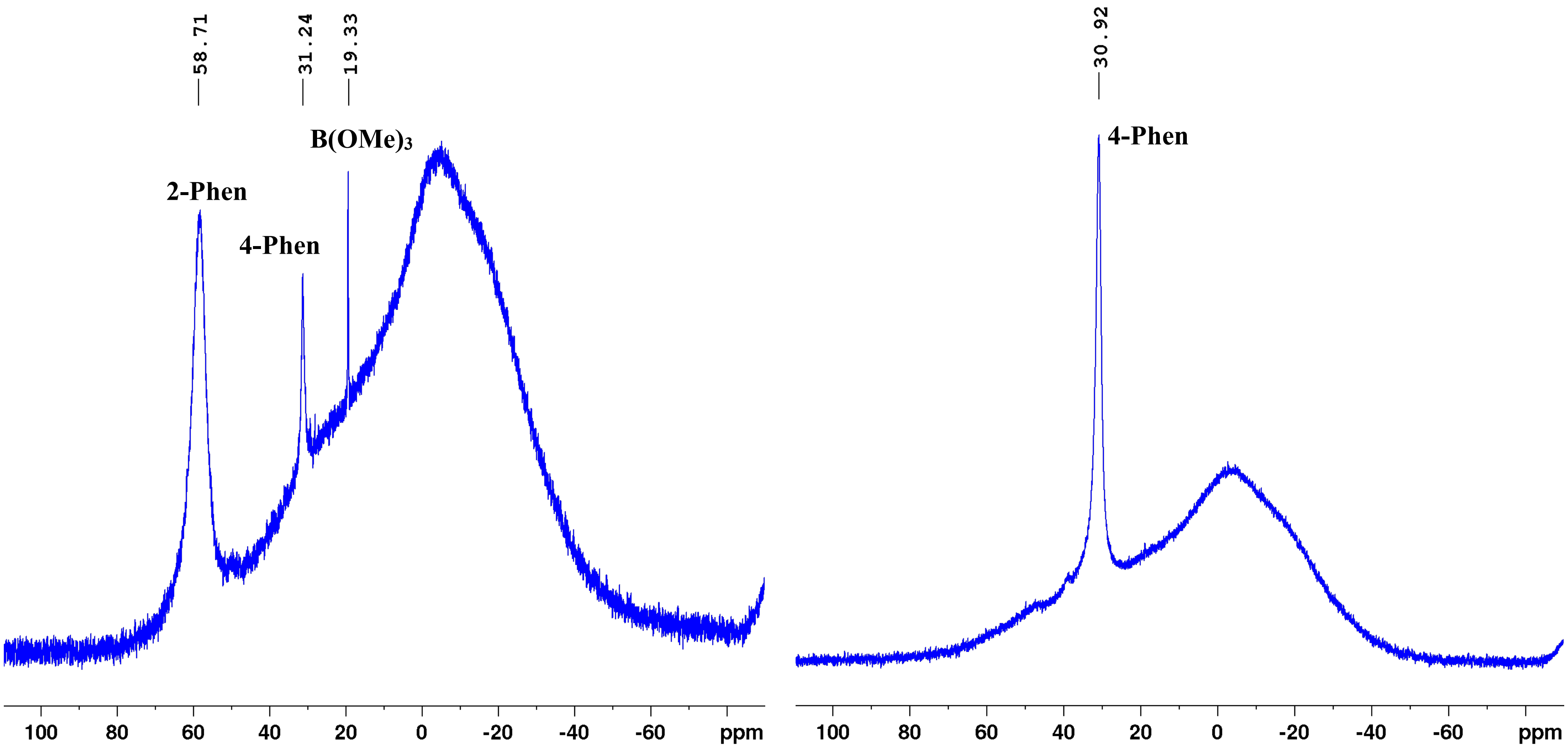

Figure S58. ^{11}B NMR spectra of the synthesis of **2-Phen**, **method A**, directly prior to workup (left) and after workup (right).

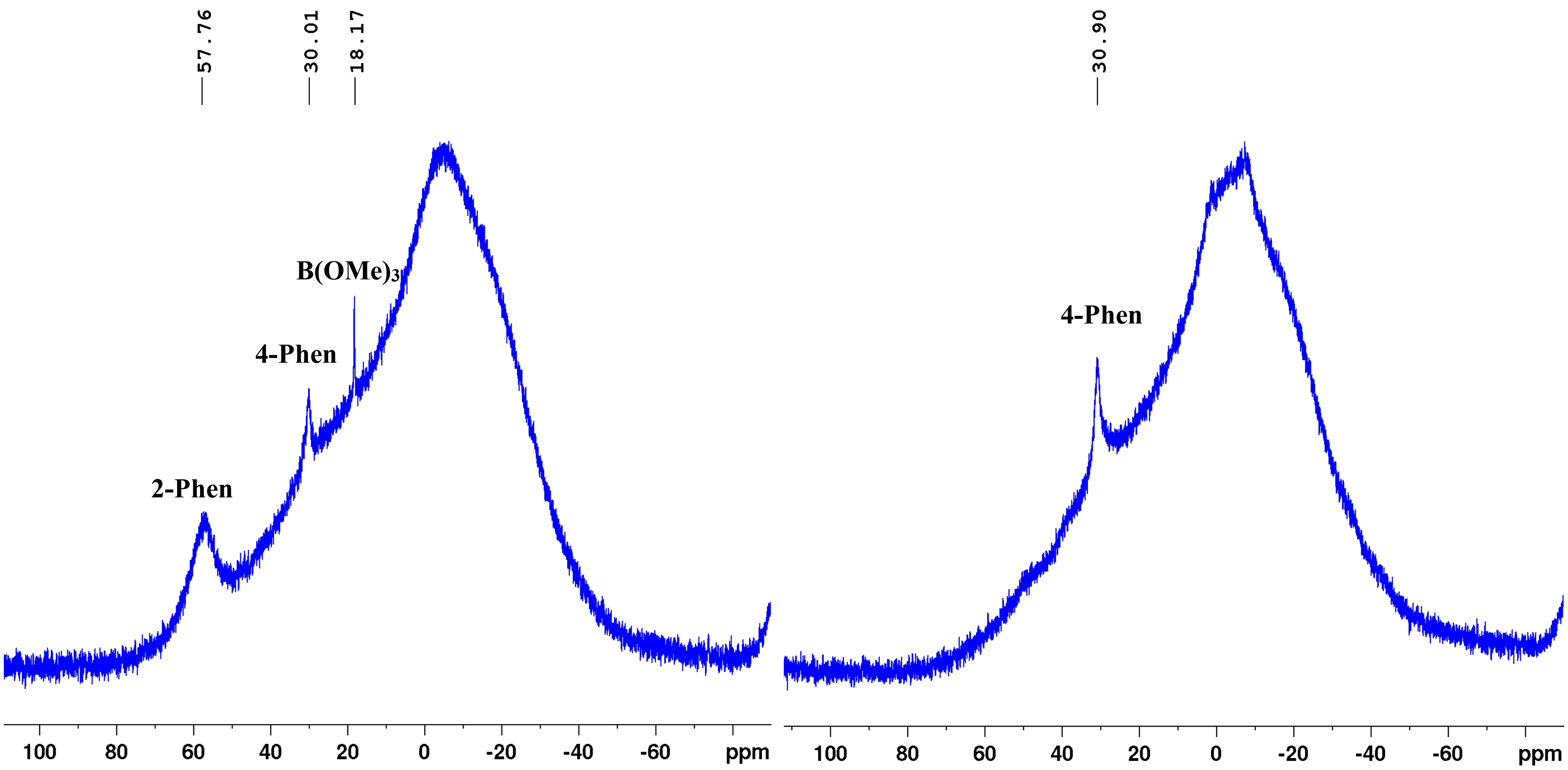

Figure S59. ^{11}B NMR spectra of the synthesis of **2-Phen**, **method B**, directly prior to workup (left) and after workup (right).

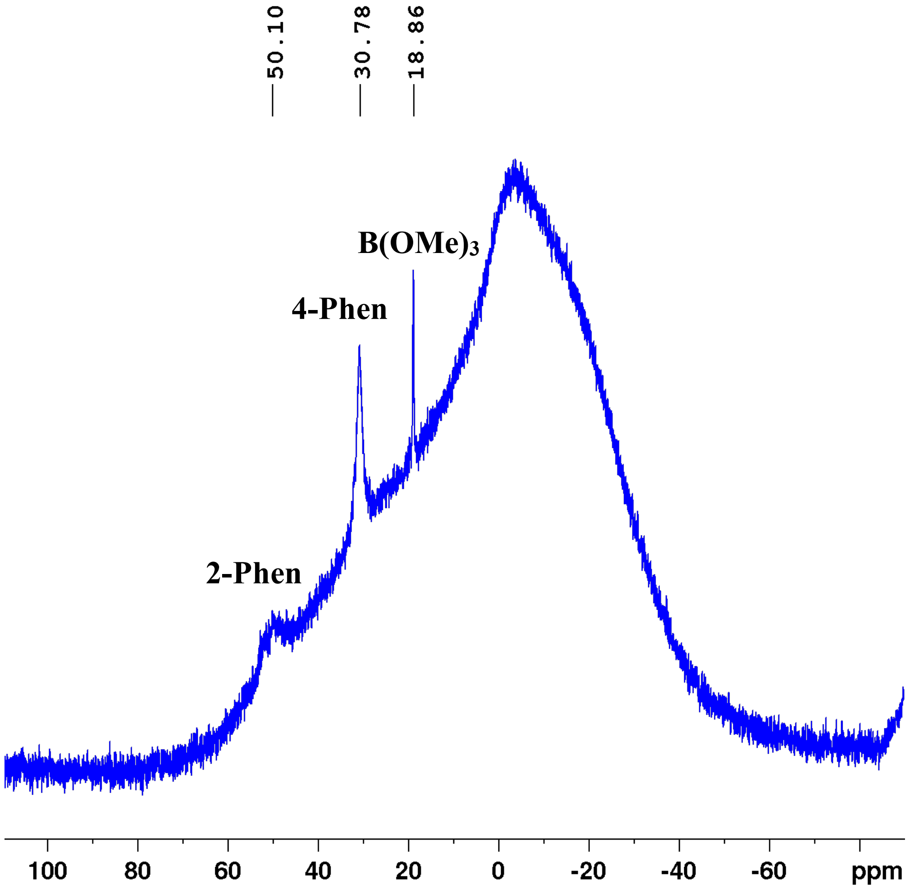

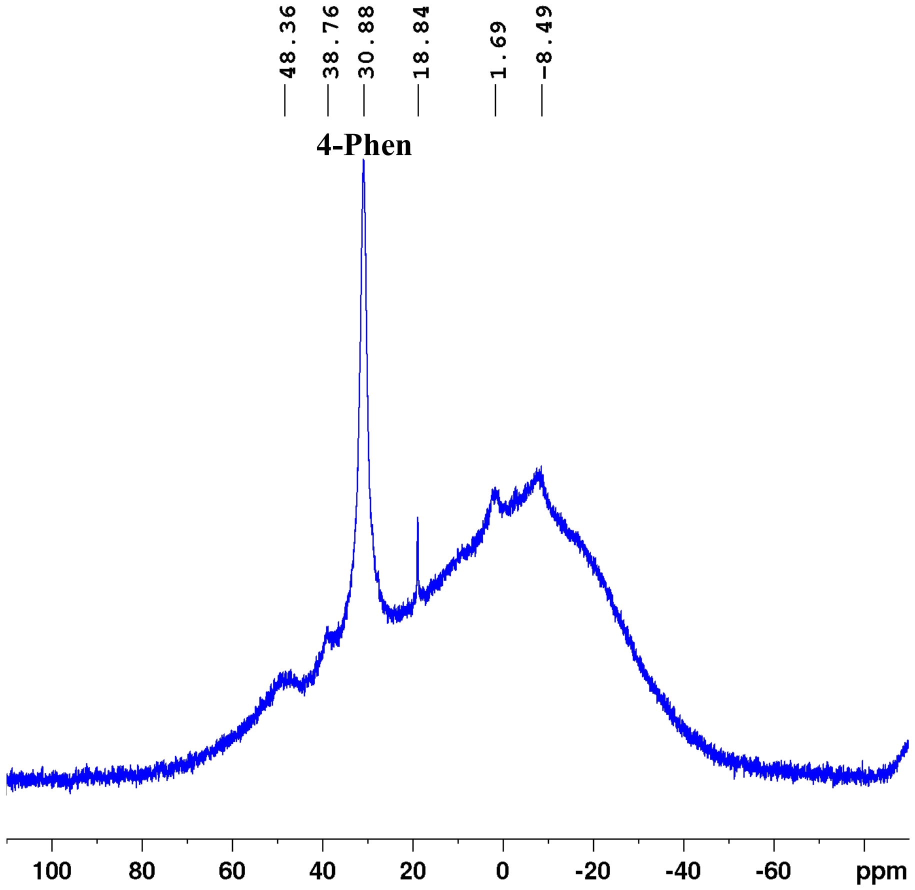

Figure S60. ^{11}B NMR spectra of the synthesis of **2-Phen**, **method C**, directly prior to workup (left) and after workup (right).

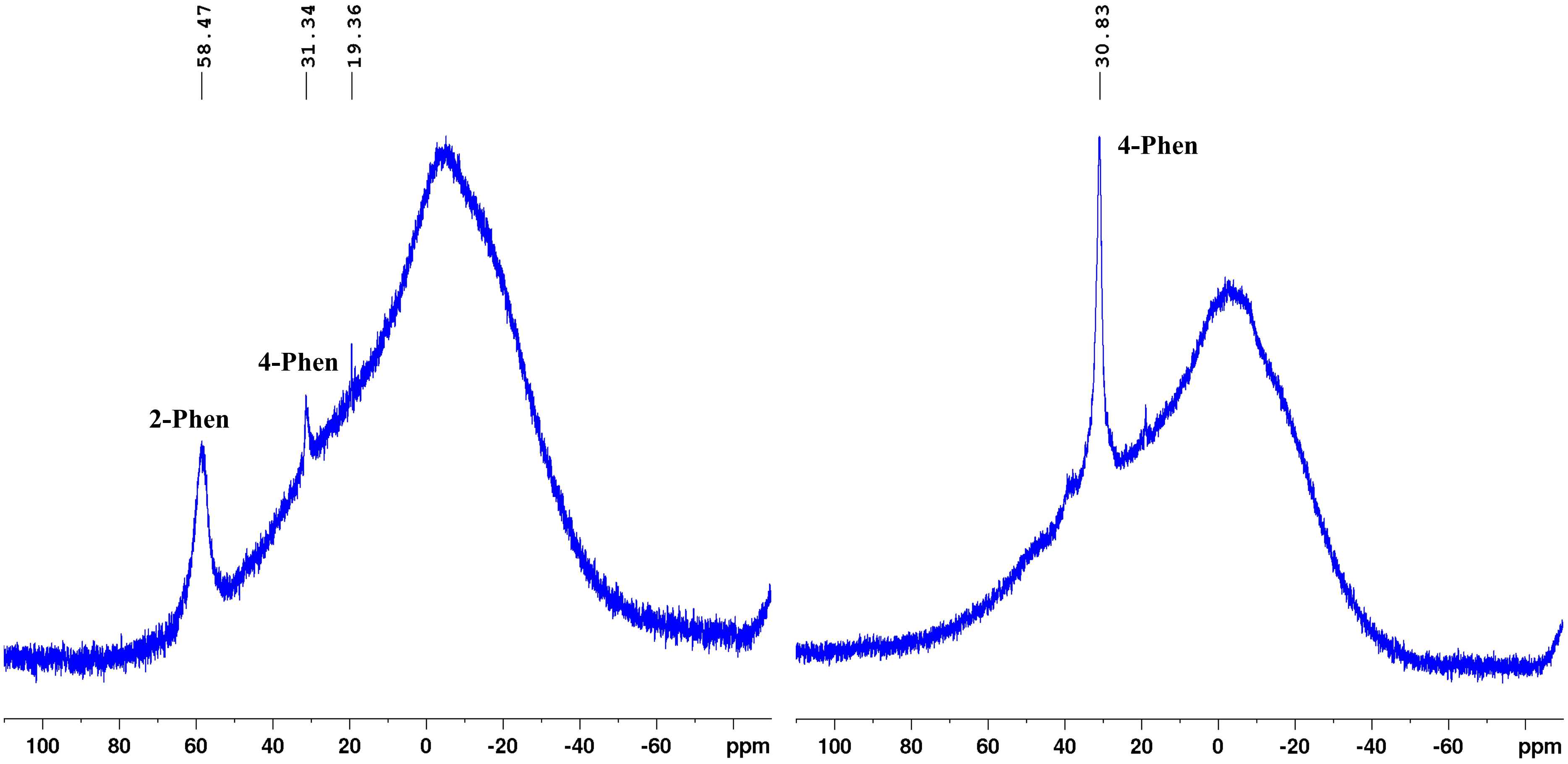

Figure S61. ^{11}B NMR spectra of the synthesis of **2-Phen**, **method D**, directly prior to workup (left) and after workup (right).

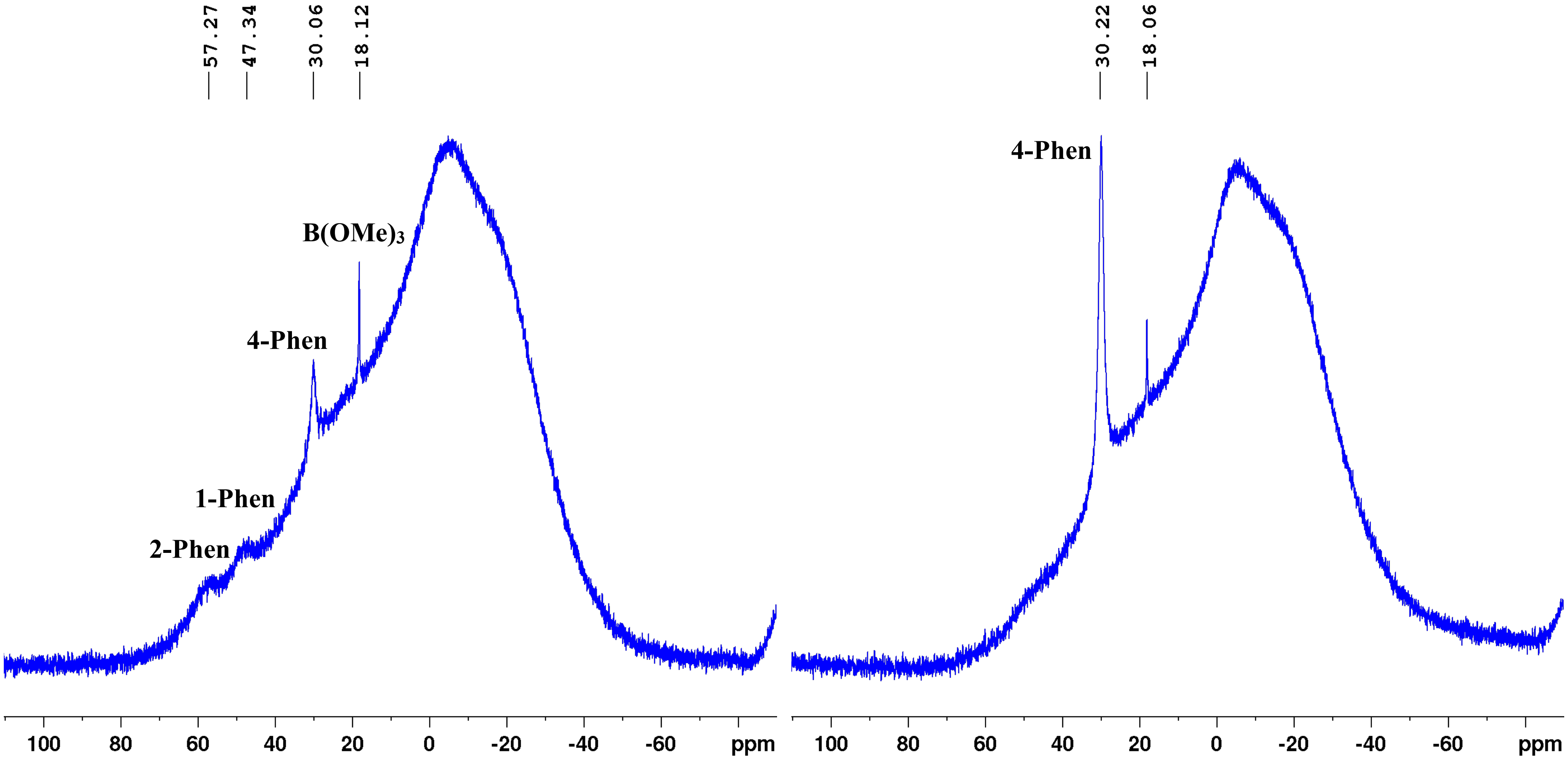

Figure S62. ^{11}B NMR spectra of the synthesis of **2-Phen**, **method E**, directly prior to workup (left) and after workup (right).

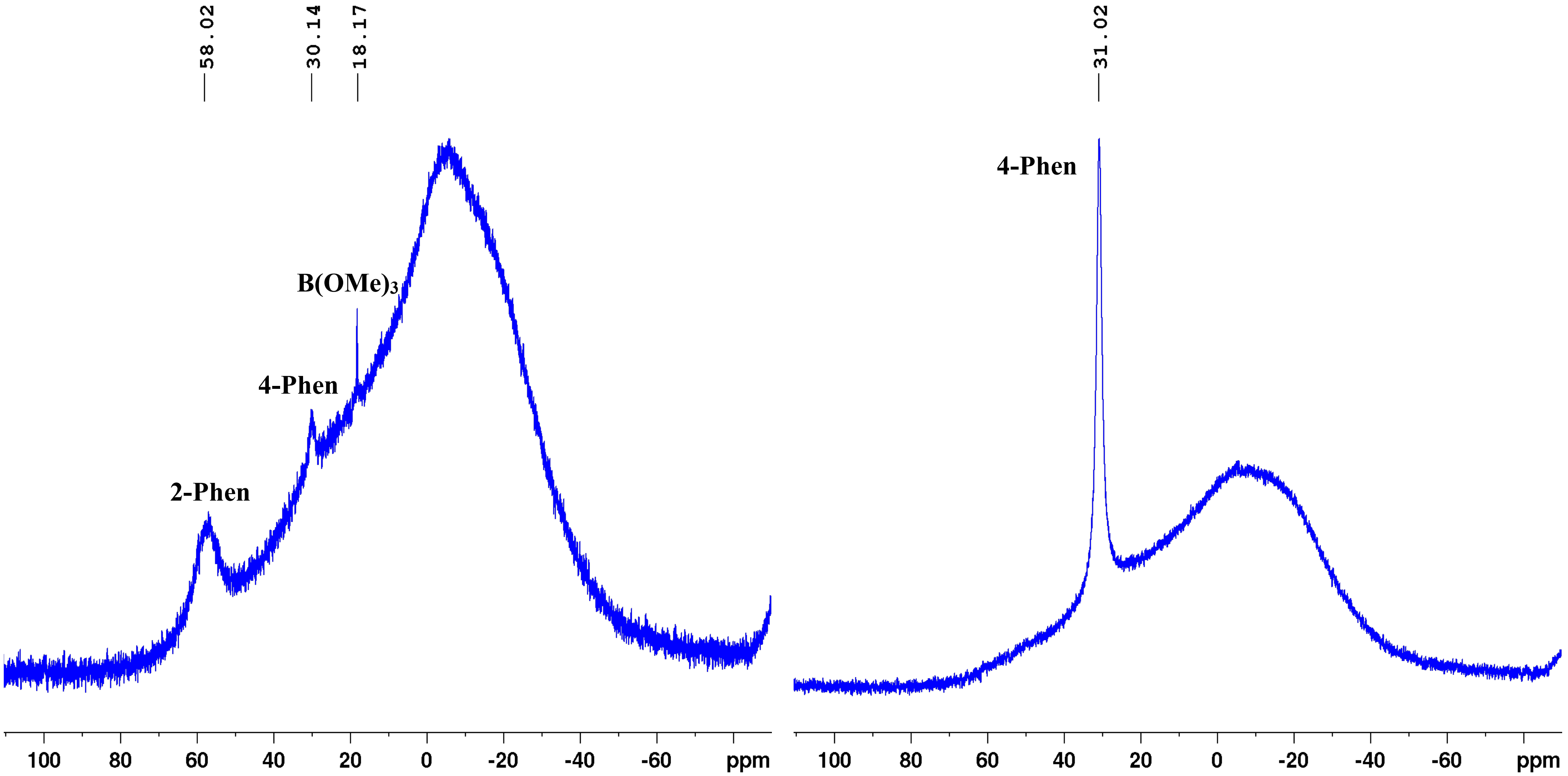

Figure S63. ^{11}B NMR spectra of the synthesis of **2-Phen**, **method F**, directly prior to workup (left) and after workup (right).

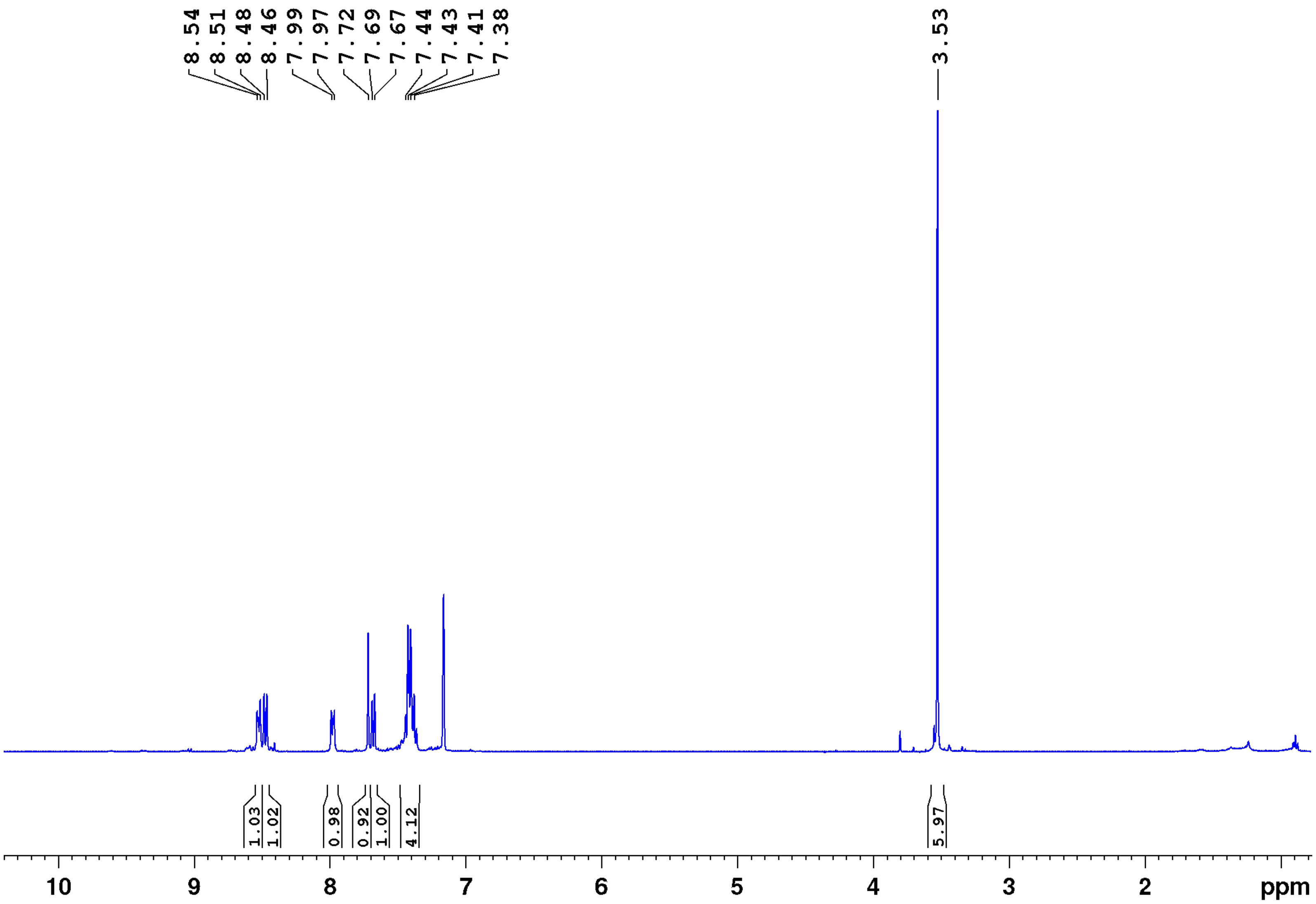

Figure S64. ^{1}H NMR spectrum of isolated **4-Phen** in C_6D_6.

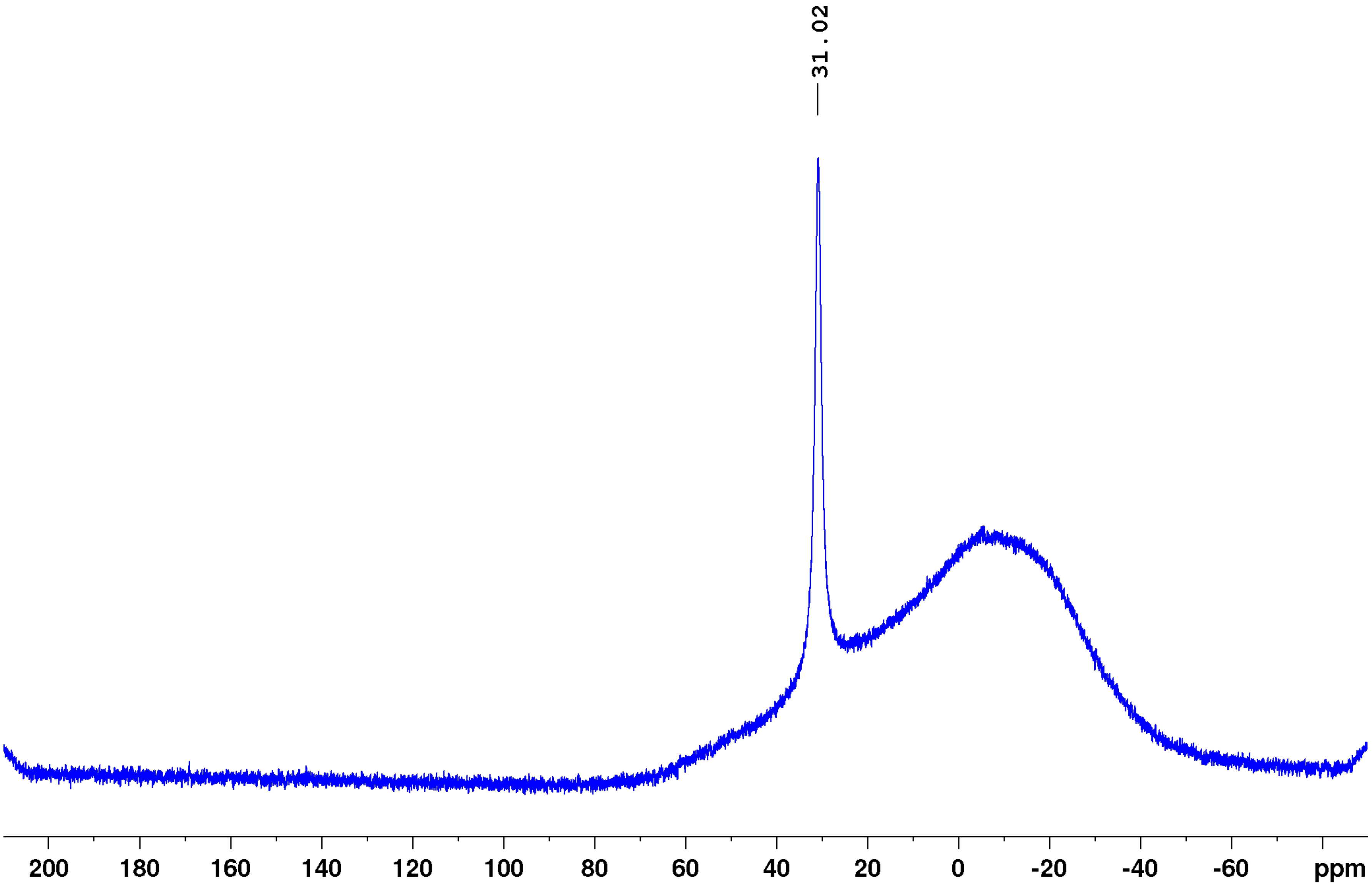

Figure S65. ^{11}B NMR spectrum of isolated **4-Phen** in C_6D_6.

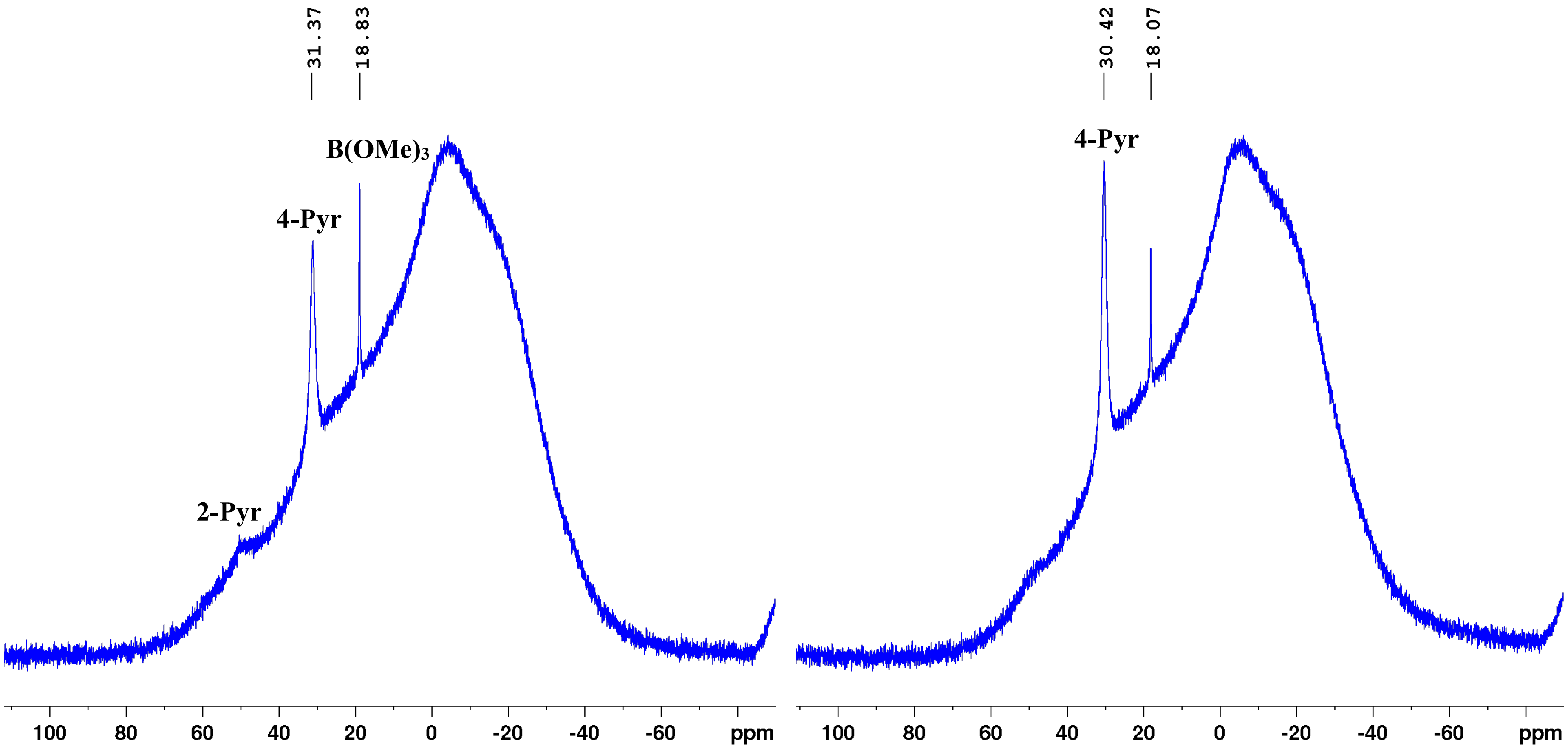

Figure S66. ^{11}B NMR spectra of the synthesis of **2-Pyr**, **method B**, directly prior to workup (left) and after workup (right).

NMR-monitoring of the addition of H_2 to 7-Ar

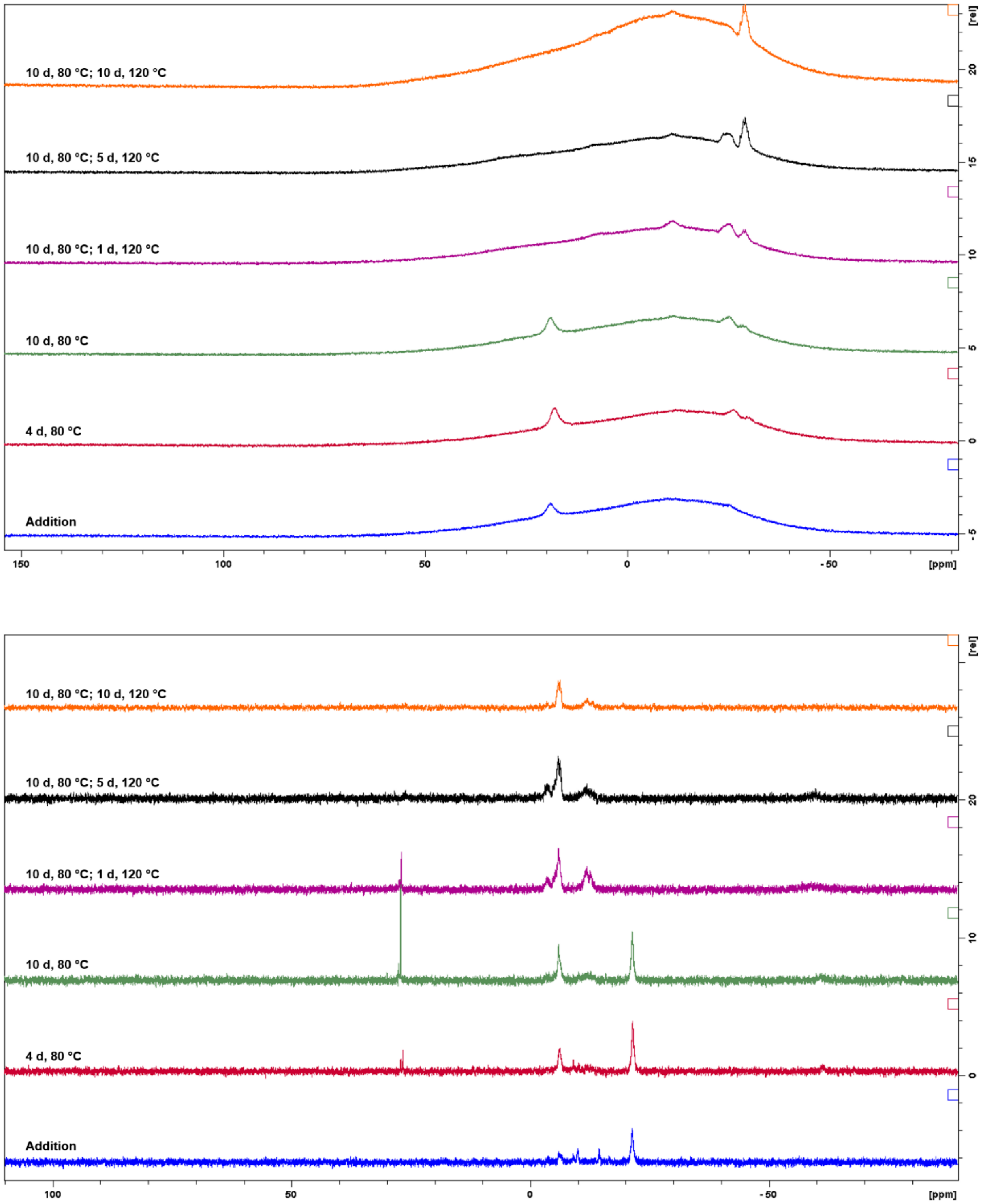

Figure S67. Stack-plots of annotated ^{11}B (top) and $^{31}P\{^{1}H\}$ NMR spectra monitoring the hydrogenation of **7-Anth** in benzene.

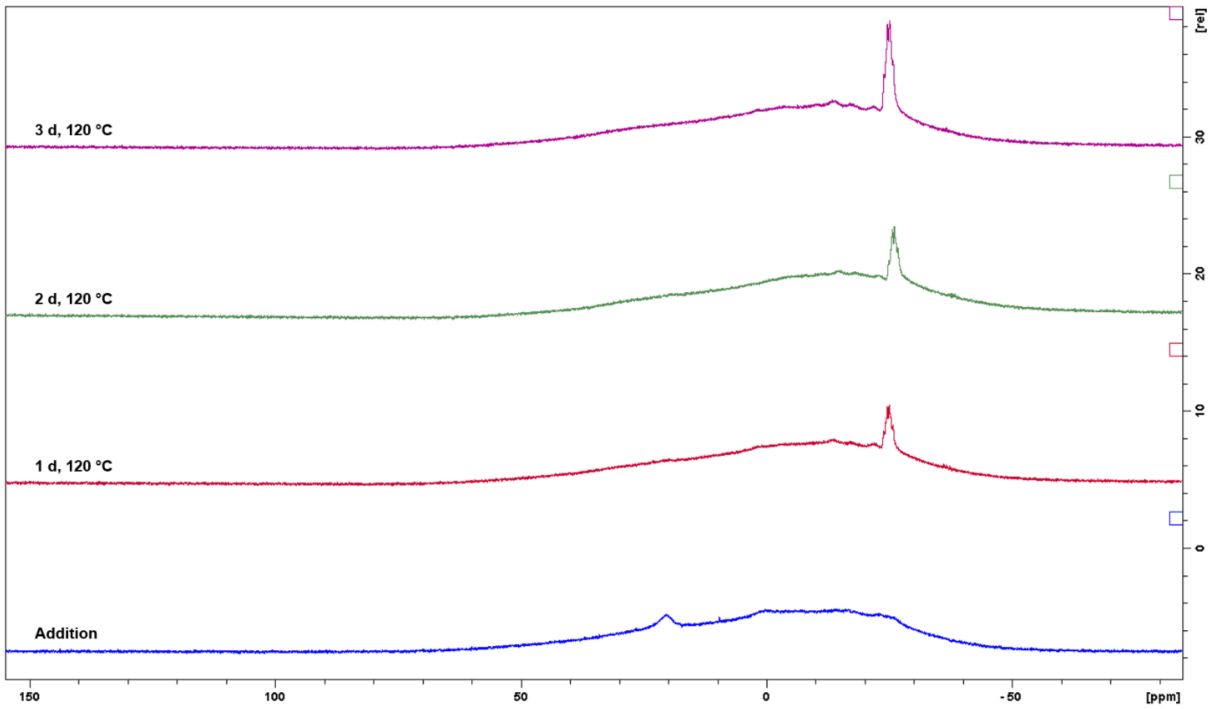

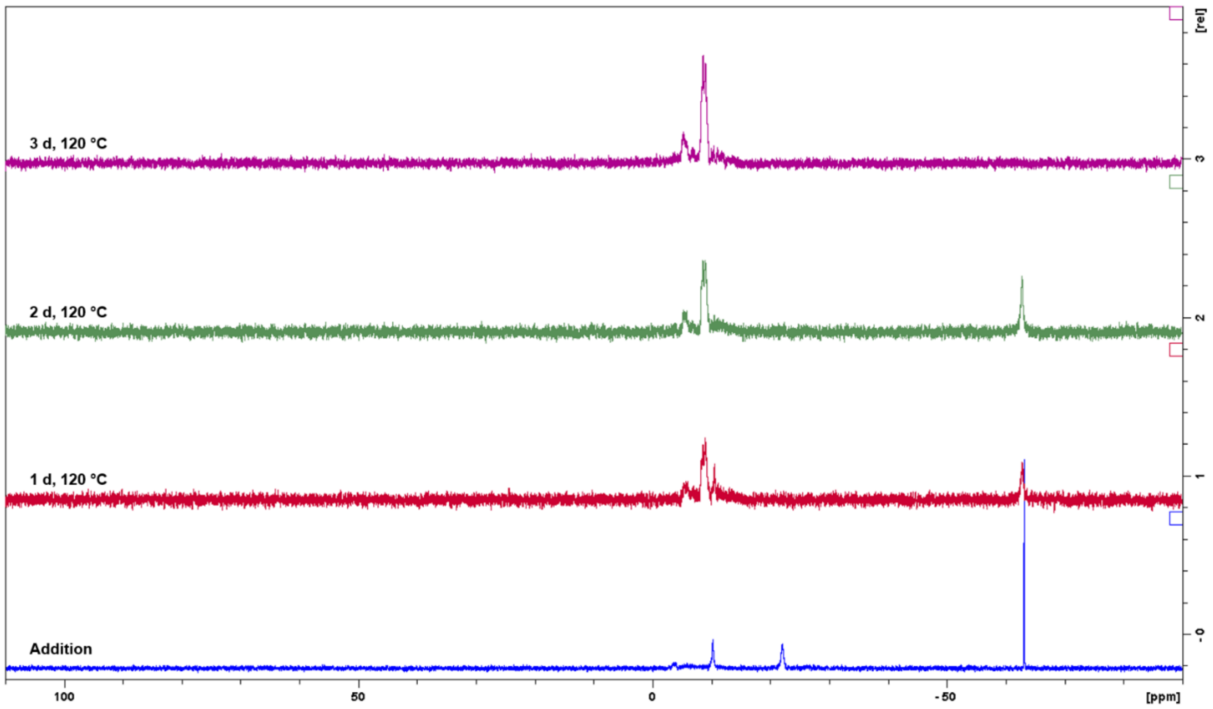

Figure S68. Stack-plots of annotated ^{11}B (top) and $^{31}P\{^1H\}$ NMR spectra monitoring the hydrogenation of **7-Phen** in THF:benzene 1:3.

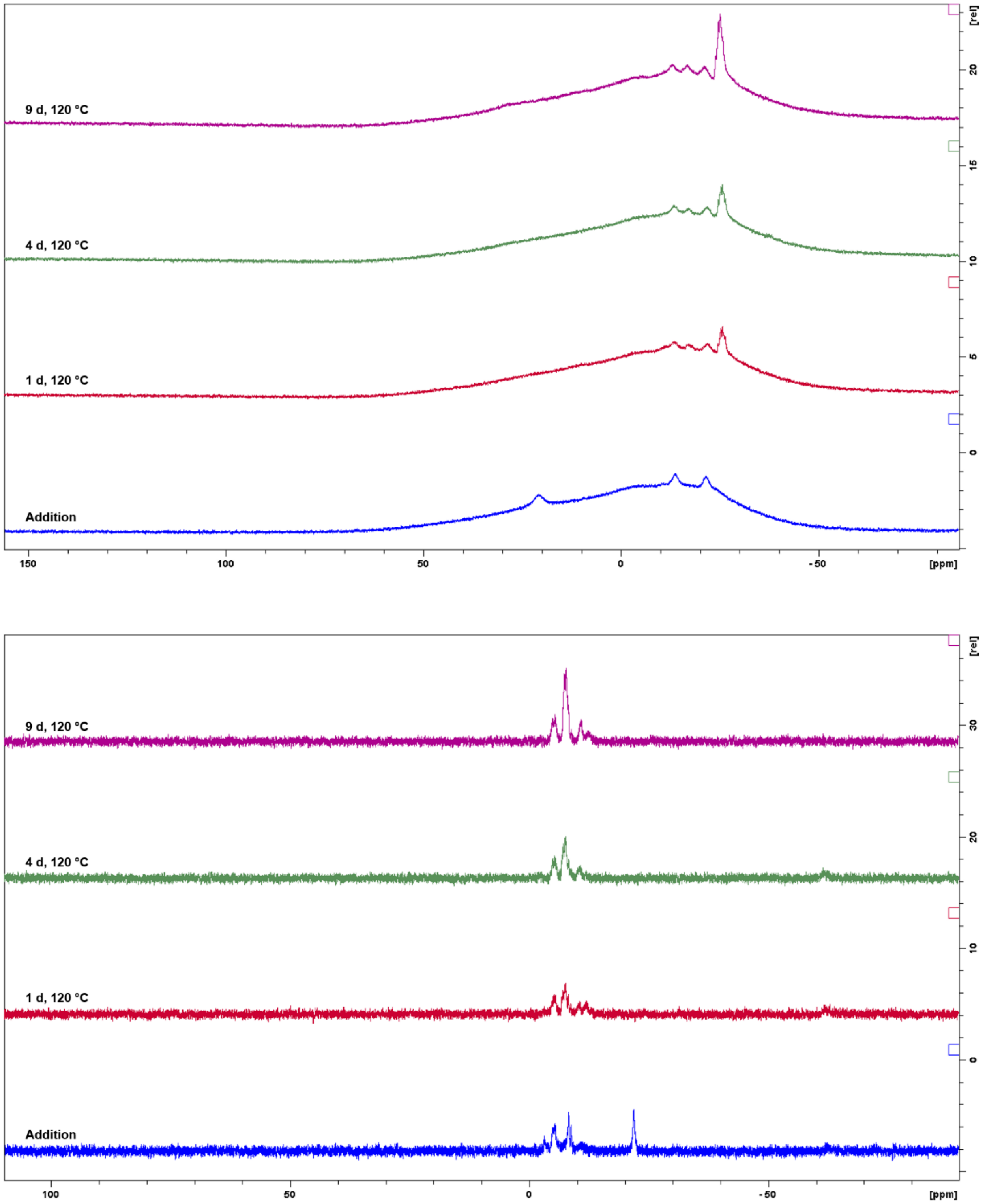

Figure S69. Stack-plots of annotated ^{11}B (top) and $^{31}P\{^1H\}$ NMR spectra monitoring the hydrogenation of **7-Pyr** in THF:benzene 1:3.

UV-vis spectra

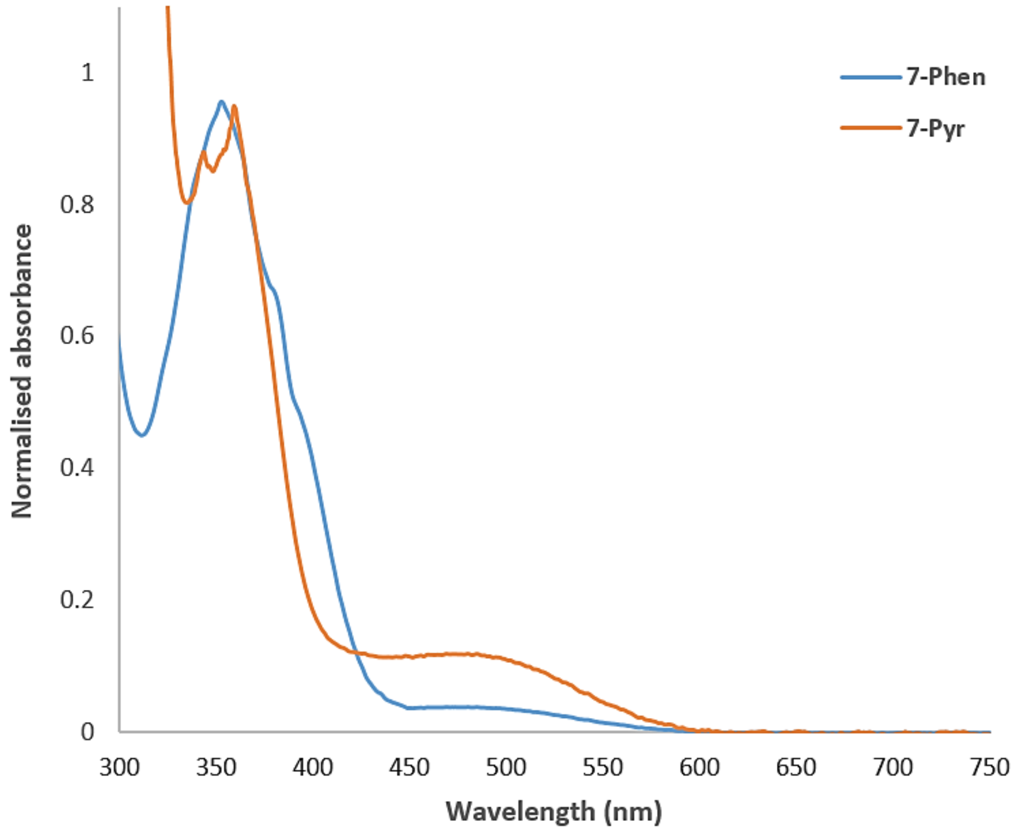

Figure S70. Overlay of UV-vis spectra of diborenes **7-Phen** (blue) and **7-Pyr** (red) in a 3:1 benzene/THF solution.

X-ray crystallographic data

The crystal data of **7-Phen** were collected on a *XtaLAB Synergy Dualflex HyPix* diffractometer with a Hybrid Pixel array detector and multi-layer mirror monochromated $Cu_{K\alpha}$ radiation. The crystal data of **1-Pyr**, **6-Phen**, **6-Pyr, 9-Anth, 10-Mes** and **10-Anth** were collected on a *Bruker D8 Quest* diffractometer with a CPA area detector and multi-layer mirror monochromated $Mo_{K\alpha}$ radiation. The crystal data of **1-Phen** and **8-Phen** were collected on a Bruker X8-APEX II diffractometer with a CCD area detector and multi-layer mirror monochromated $Mo_{K\alpha}$ radiation. The structures were solved using the intrinsic phasing method,[4] refined with the ShelXL program[5] and expanded using Fourier techniques. All non-hydrogen atoms were refined anisotropically. Hydrogen atoms were included in structure factor calculations. All hydrogen atoms were assigned to idealised geometric positions.

Crystallographic data have been deposited with the Cambridge Crystallographic Data Center as supplementary publication nos. CCDC 2169207 (**1-Phen**), 2169208 (**6-Pyr**), 2169209 (**8-Phen**), 2169210 (**7-Phen**), 2169211 (**10-Anth**), 2169212 (**1-Pyr**), 2169213 (**9-Anth**), 2169214 (**6-Phen**) and 2169215 (**10-Mes**). These data can be obtained free of charge from The Cambridge Crystallographic Data Center *via* www.ccdc.cam.ac.uk/data_request/cif.

Crystal data for 1-Phen: $C_{35}H_{33}B_2N_2$, $M_r = 503.25$, colourless block, $0.814\times0.357\times0.312$ mm^3, triclinic space group $P\bar{1}$, $a = 9.694(4)$ Å, $b = 12.008(5)$ Å, $c = 12.976(5)$ Å, $\alpha = 74.380(18)°$, $\beta = 80.89(2)°$, $\gamma = 71.04(2)°$, $V = 1371.6(10)$ Å^3, $Z = 2$, $\rho_{calcd} = 1.219$ g·cm^{-3}, $\mu = 0.069$ mm^{-1}, $F(000) = 534$, $T = 170(2)$ K, $R_1 = 0.0883$, $wR_2 = 0.1308$, 5402 independent reflections [$2\theta \leq 52.04°$] and 356 parameters.

Crystal data for 1-Pyr: $C_{36}H_{30}B_2N_2$, $M_r = 512.24$, colourless block, $0.331\times0.269\times0.244$ mm^3, monoclinic space group $P2_1/c$, $a = 13.398(4)$ Å, $b = 18.112(6)$ Å, $c = 23.412(10)$ Å, $\beta = 104.273(13)°$, $V = 5506(3)$ Å^3, $Z = 8$, $\rho_{calcd} = 1.236$ g·cm^{-3}, $\mu = 0.070$ mm^{-1}, $F(000) = 2160$, $T = 101(2)$ K, $R_1 = 0.0550$, $wR_2 = 0.1286$, 11259 independent reflections [$2\theta \leq 52.746°$] and 729 parameters.

Refinement details for 6-Phen: The asymmetric unit contains half a twofold disordered THF molecule placed on a fourfold rotoinversion axis, modelled with PARTs -1 and -2 and refined to a 64:36 ratio. The displacement parameters of all atoms of residues RESI 3 THF and RESI 4 THF were restrained to the same value with similarity restraint SIMU. The 1-2 and 1-3 distances in RESI 3 THF and RESI 4 THF were restrained to the same values with SAME.

Crystal data for 6-Phen: $C_{34}H_{36}B_2Cl_2P_2{\cdot}C_4H_8O$, $M_r = 671.24$, colourless block, $0.273\times0.266\times0.222$ mm^3, tetragonal space group $P4_2/n$, $a = b = 14.3363(13)$ Å, $c = 17.508(2)$ Å, $V = 3598.4(8)$ Å^3, $Z = 4$, $\rho_{calcd} = 1.239$ g·cm^{-3}, $\mu = 0.299$ mm^{-1}, $F(000) = 1416$, $T = 100(2)$ K, $R_1 = 0.0461$, $wR_2 = 0.0838$, 3540 independent reflections [$2\theta \leq$ 52.04°] and 275 parameters.

Refinement details for 6-Pyr: The asymmetric unit contains one molecule of THF, which was modelled as twofold disordered and refined to a 58:42 ratio. The displacement parameters of all atoms in the residues RESI 6 and 16 THF were restrained to the same value with similarity restraint SIMU 0.01.

Crystal data for 6-Pyr: $C_{38}H_{36}B_2Cl_2P_2{\cdot}C_4H_8O$, $M_r = 719.23$, colourless block, $0.248\times0.234\times0.121$ mm^3, triclinic space group $P\bar{1}$, $a = 11.987(4)$ Å, $b = 12.467(4)$ Å, $c = 12.683(4)$ Å, $\alpha = 83.308(9)$°, $\beta = 88.82(2)$°, $\gamma = 76.780(13)$°, $V = 1832.5(10)$ Å^3, $Z = 2$, $\rho_{calcd} = 1.303$ g·cm^{-3}, $\mu = 0.298$ mm^{-1}, $F(000) = 756$, $T = 100(2)$ K, $R_1 = 0.0852$, $wR_2 = 0.1604$, 7665 independent reflections [$2\theta \leq 53.464$°] and 444 parameters.

Refinement details for 7-Phen: Refined as a 2-component twin. Component 2 rotated by 2.6239° around [–0.47 0.62 0.63] (reciprocal) or [–0.20 0.84 0.50] (direct). The BASF parameter was refined to 53.7%. The asymmetric unit contains 1.5 benzene molecules, the half one being twofold disordered over an inversion centre and modelled using PART -1 10.5 and AFIX 66 and ISOR 0.01 restraints. The entire diborene is centrosymmetric and twofold disordered in a 1:1 ratio (PART 2 and 3, respectively, for the phenanthryl ligands, PART 1 and 4 for the $BPMe_3$ fragments) via a mirror perpendicular to the boron planes and bisecting the B-B bond in its centre. The external aromatic rings of the phenanthryl ligands (C2 > C7 and C8 > C13) were modelled with AFIX 66. The PMe_3 ligand of PART 1 was modelled as threefold

disordered by rotation about the B–P axis in a 1:1:3 ratio (PARTS 11, 12 and 13, respectively). All B–P bond lengths were restrained to similarity using SADI 0.002. All 1,2- and 1,3-distances in all four PMe_3 fragments were restrained to similarity with SAME. The ADPs of the phenanthryl ligands (RESI 2 and 3 Phen) were restrained with SIMU 0.01, those of the $BPMe_3$ fragments with SIMU 0.005 and those of the boron and phosphorus atoms with SIMU 0.002. Due to the disorders and the large number of restraints the data can only be used as proof of connectivity.

Crystal data for 7-Phen: $C_{52}H_{54}B_2P_2$, M_r = 254.17, yellow plate, 0.266×0.126×0.083 mm^3, triclinic space group $P\bar{1}$, a = 9.1596(3) Å, b = 9.7622(4) Å, c = 13.7547(5) Å, α = 106.196(3)°, β = 97.907(3)°, γ = 109.115(3)°, V = 1079.89(7) $Å^3$, Z = 3, ρ_{calcd} = 1.173 g·cm^{-3}, μ = 1.162 mm^{-1}, $F(000)$ = 406, T = 99.99(10) K, R_1 = 0.0977, wR_2 = 0.2577, 7990 independent reflections [$2\theta \leq 136.466°$] and 465 parameters.

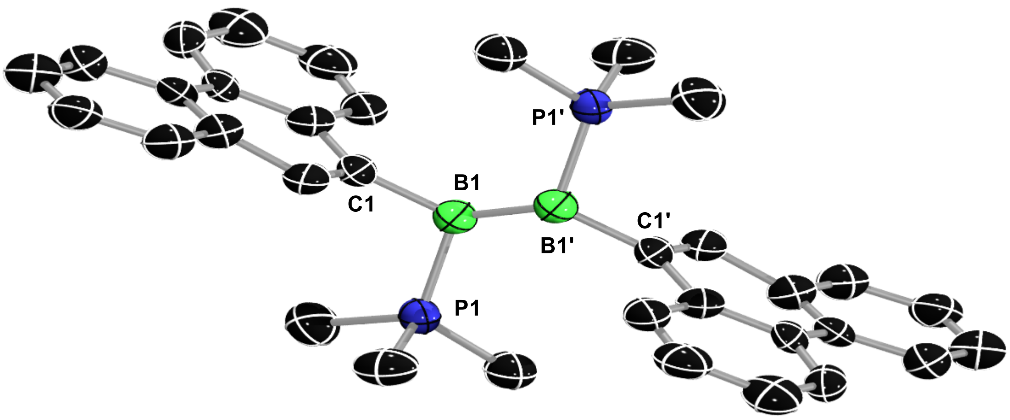

Figure S71. Crystallographically-derived solid-state structures of **7-Phen** (one of the two mirror-disordered molecules present in the unit cell). Atomic displacement ellipsoids at 50%. Ellipsoids of ligand periphery and hydrogen atoms omitted for clarity.

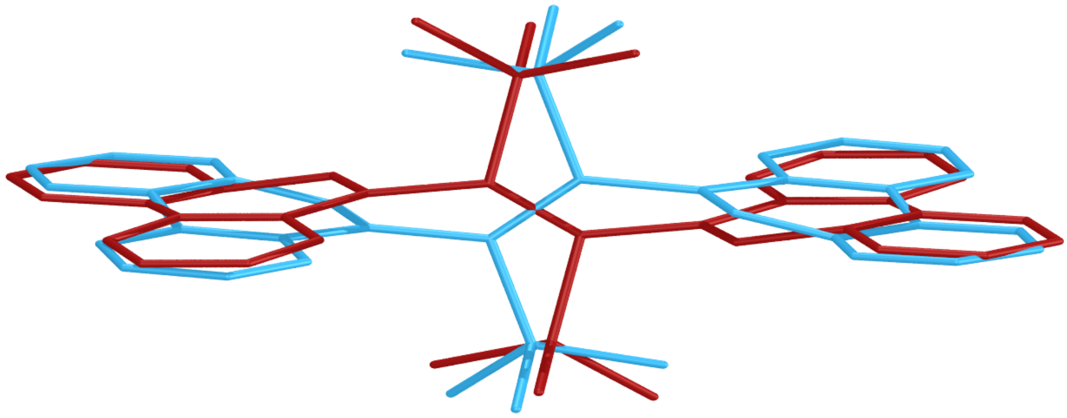

Figure S72. Overlay of the two disordered parts of diborene **7-Phen** in the asymmetric unit.

Crystal data for 8-Phen: $C_{46}H_{48}B_2P_2$, $M_r = 684.40$, colourless block, $0.559\times0.436\times0.362$ mm^3, triclinic space group $P\bar{1}$, $a = 9.478(4)$ Å, $b = 13.2148(14)$ Å, $c = 16.397(7)$ Å, $\alpha = 83.571(10)°$, $\beta = 79.41(4)°$, $\gamma = 71.546(5)°$, $V = 1911.8(12)$ Å^3, $Z = 2$, $\rho_{calcd} = 1.189$ g·cm^{-3}, $\mu = 0.146$ mm^{-1}, $F(000) = 728$, $T = 101(2)$ K, $R_1 = 0.0443$, $wR_2 = 0.1022$, 8132 independent reflections [$2\theta \leq 53.538°$] and 461 parameters.

Crystal data for 9-Anth: $C_{31}H_{29}B_2P\cdot C_6H_6$, $M_r = 532.24$, yellow block, $0.21\times0.19\times0.15$ mm^3, monoclinic space group $P2_1/c$, $a = 19.472(2)$ Å, $b = 16.526(2)$ Å, $c = 9.3359(16)$ Å, $\beta = 102.454(6)°$, $V = 2933.6(7)$ Å^3, $Z = 4$, $\rho_{calcd} = 1.205$ g·cm^{-3}, $\mu = 0.119$ mm^{-1}, $F(000) = 1128$, $T = 100(2)$ K, $R_1 = 0.0690$, $wR_2 = 0.1633$, 5779 independent reflections [$2\theta \leq$ 52.026°] and 433 parameters.

Crystal data for 10-Mes: $C_{12}H_{22}BP$, $M_r = 208.07$, colourless block, $0.354\times0.239\times0.207$ mm^3, tetragonal space group $I4_1/a$, $a = b = 13.697(2)$ Å, $c = 28.327(9)$ Å, $V = 5315(3)$ Å^3, $Z = 16$, $\rho_{calcd} = 1.040$ g·cm^{-3}, $\mu = 0.171$ mm^{-1}, $F(000) = 1824$, $T = 100(2)$ K, $R_1 = 0.0425$, $wR_2 = 0.0895$, 2614 independent reflections [$2\theta \leq 52.036°$] and 141 parameters.

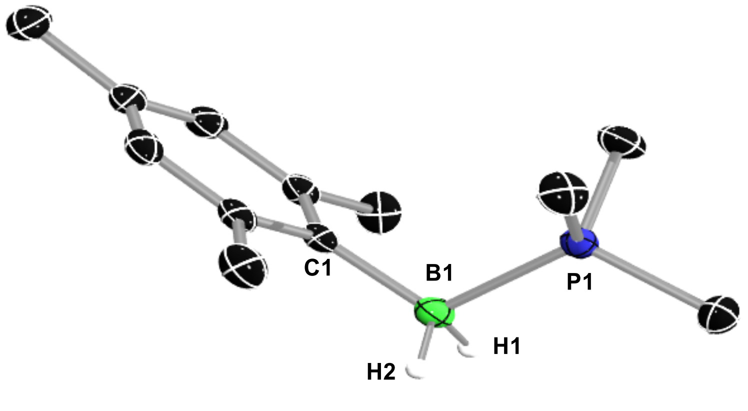

Figure S73. Crystallographically-derived solid-state structure of **10-Mes** (one of the two mirror-disordered molecules present in the unit cell). Atomic displacement ellipsoids at 50%. Ellipsoids of ligand periphery and hydrogen atoms omitted for clarity.

Crystal data for 10-Anth: $C_{17}H_{20}BP$, M_r = 266.11, colourless block, 0.369×0.346×0.238 mm^3, monoclinic space group *C*2/*c*, *a* = 25.718(9) Å, *b* = 7.093(2) Å, *c* = 18.318(3) Å, β = 116.062(10)°, *V* = 3001.7(15) Å^3, *Z* = 8, ρ_{calcd} = 1.178 g·cm^{-3}, μ = 0.166 mm^{-1}, *F*(000) = 1136, *T* = 100(2) K, R_1 = 0.0439, wR_2 = 0.1141, 3061 independent reflections [2θ≤52.742°] and 175 parameters.

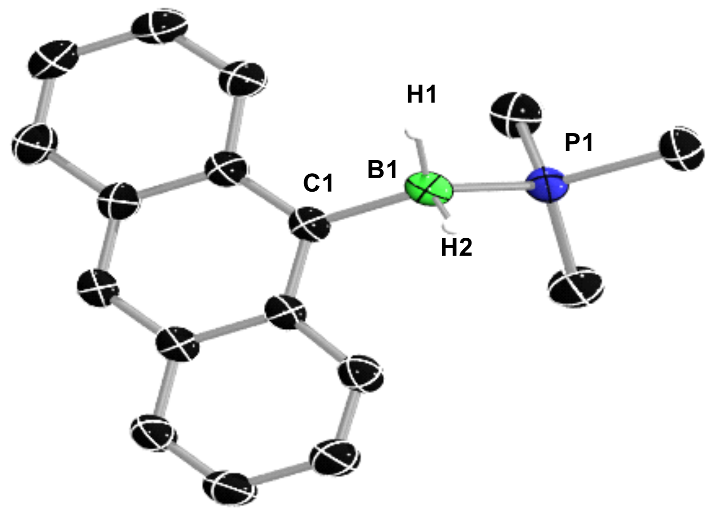

Figure S74. Crystallographically-derived solid-state structure of **10-Anth**. Atomic displacement ellipsoids at 50%. Ellipsoids of ligand periphery and hydrogen atoms omitted for clarity.

Computational details

All computations were performed using the Gaussian 16 (Revision C.01) software.[6] Two level of theories were employed for geometry optimisations and vibrational frequency calculations: (i) the hybrid DFT functional M06[7] and the double split valence basis set 6-31G(d,p), combined with the SMD solvation model (SCRF = SMD) for the inclusion of the benzene, toluene or THF solvent effect;[8] and (ii) the hybrid DFT functional B3LYP9 and the double split valence basis set 6-31G(d,p), augmented with Grimme's dispersion correction[10] and the PCM solvation model (SCRF = IEFPCM) for the inclusion of the benzene, toluene or THF solvent effect.[11] While both level of theories provided similar results, only the results obtained from the former level of theory are reported in this work, as a similar level of theory has been previously employed to study the hydrogenation chemistry of a diborane(4) compound.[12] Geometry optimisation of all stationary points was performed without any symmetry constraints. Transition state geometries were obtained using opt = (ts, noeigentest, calcfc) algorithms.[13] All optimised transition state structures were confirmed as maxima with only one imaginary frequency, and the magnitudes of all frequencies were greater than the residual frequencies due to rotations and translations.

Additionally, each transition state located was ensured to be on the preferred reaction path by performing "plus-and-minus-displacement" minimisation calculations,[14] which involves the displacement of transition state structure by ca. 0.05 Å or 5°–10° along the imaginary frequency normal mode in both directions, and the displaced geometries were subsequently optimised to the nearest minimum. Zero-point vibrational energies and thermal corrections were computed from frequency calculations with a standard state of 298 K and 1 atm. The energies (ΔG) given are corrected for zero-point vibrational energies (ZPVEs).

Computations on the rotational isomers of 2-Phen and 2-Pyr

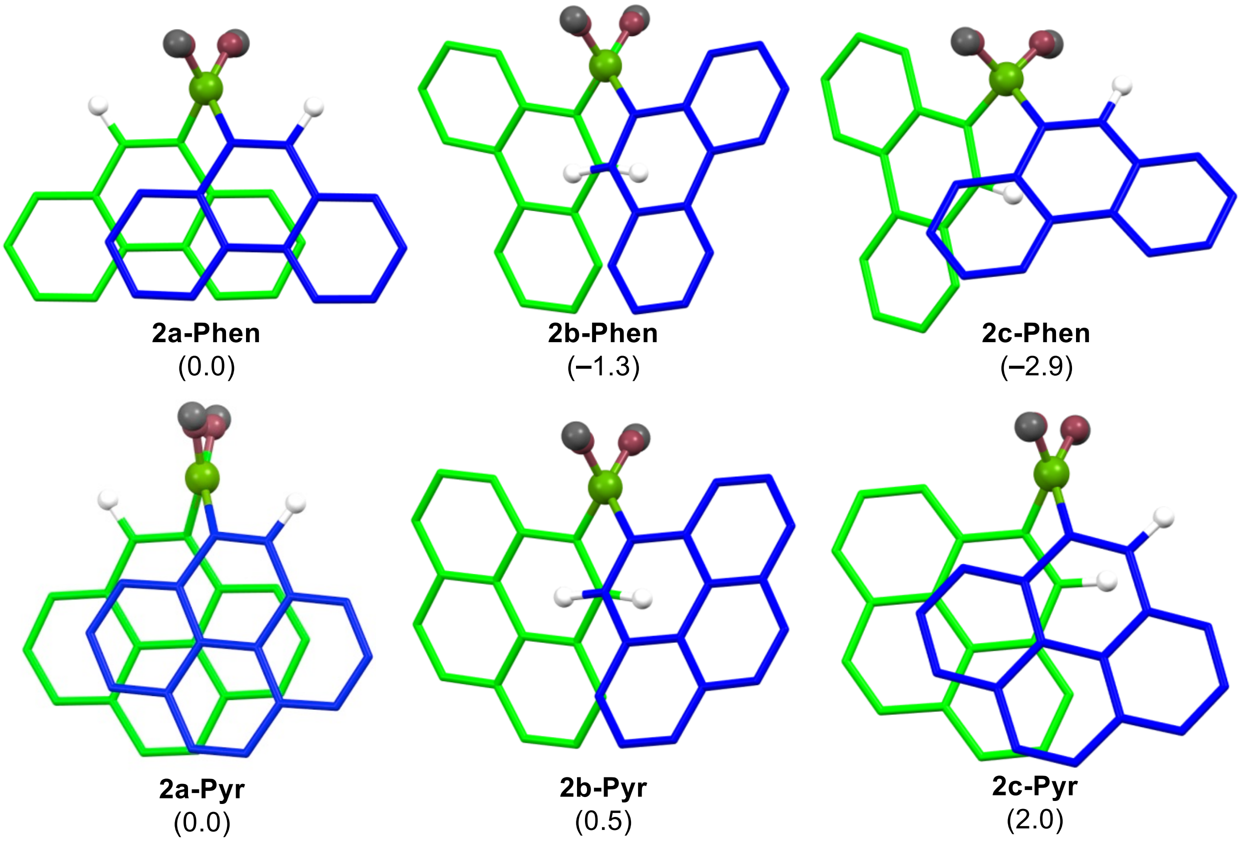

Figure S75. Structures of the computed rotational isomers of **2-Phen** and **2-Pyr,** viewed along the B–B axis, and their free energy difference (ΔG in kcal mol^{-1}). Hydrogen atoms omitted for clarity, except for *ortho* protons.

Energies and cartesian coordinates of 2-Ar:

1) 2a-Phen

Number of imaginary frequencies = 0

E_{total} = -1356.965782 a.u

$G_{correction}$ = 0.403906 a.u

Cartesian coordinates:

B	-0.36926900	2.59840500	-0.77285100
B	0.36924000	2.59837900	0.77290000
C	1.32842900	1.35023700	0.94074400
C	2.56693300	1.34903300	0.36846400
C	3.34564900	0.16091200	0.21675200
C	2.81540800	-1.08442800	0.64057200
C	1.50985300	-1.11212600	1.27159100
C	0.79344200	0.10220300	1.43784500
C	-0.48335800	0.06668400	2.03762800
C	-1.06046600	-1.11886800	2.43317500
C	-0.35630900	-2.31817500	2.26155500
C	0.90355800	-2.30869600	1.70282800
C	3.57749800	-2.24417800	0.39897200
C	4.80473500	-2.18032000	-0.22558900
C	5.32732500	-0.94533900	-0.63774900
C	4.60358400	0.20404200	-0.41683200
O	0.03964500	3.45930900	1.76198900
C	0.56188600	3.36195900	3.07289500
C	-1.32844600	1.35025700	-0.94072500
C	-2.56695200	1.34902700	-0.36844700
C	-3.34565000	0.16089200	-0.21675300
C	-2.81539100	-1.08443400	-0.64059200
C	-1.50983900	-1.11210200	-1.27161700
C	-0.79344400	0.10223900	-1.43785000
C	0.48335500	0.06675000	-2.03763900
C	1.06047700	-1.11878700	-2.43321100
C	0.35633600	-2.31810600	-2.26161300
C	-0.90353000	-2.30865600	-1.70288100
C	-3.57745900	-2.24420000	-0.39900000
C	-4.80469300	-2.18037000	0.22556800
C	-5.32730300	-0.94540300	0.63774400
C	-4.60358300	0.20399300	0.41683600
O	-0.03969800	3.45937400	-1.76191300
C	-0.56194700	3.36205500	-3.07281900
H	2.97313500	2.26953100	-0.05816700
H	-1.03257700	0.99973500	2.16330300
H	-2.06188900	-1.12567400	2.85866700
H	-0.80479800	-3.26044800	2.56788100
H	1.42856700	-3.25296700	1.58391300
H	3.19565300	-3.21586600	0.70119900
H	5.36888700	-3.09348700	-0.40032200
H	6.29537800	-0.89918300	-1.13077700
H	4.98911800	1.17138600	-0.73677900
H	1.33096000	2.58333300	3.15053800
H	-0.25137900	3.13137700	3.77034600
H	0.99548200	4.32710800	3.35359200
H	-2.97316600	2.26951200	0.05820000
H	1.03255800	0.99981200	-2.16330000
H	2.06189600	-1.12557100	-2.85871200
H	0.80483600	-3.26036800	-2.56796000
H	-1.42852700	-3.25293600	-1.58398800
H	-3.19559600	-3.21587800	-0.70123700
H	-5.36882700	-3.09355000	0.40029600
H	-6.29535400	-0.89926900	1.13077600
H	-4.98913200	1.17132700	0.73679800
H	0.25131500	3.13149100	-3.77028000
H	-1.33101900	2.58342800	-3.15047600
H	-0.99554600	4.32721000	-3.35348900

2) 2b-Phen

Number of imaginary frequencies = 0

E_{total} = -1356.964910 a.u

$G_{correction}$ = 0.400897 a.u

Cartesian coordinates:

B	-0.69056200	-1.88214600	0.50686500
B	0.69055200	-1.88214300	-0.50692600
C	1.76705400	-0.73500800	-0.41463100
C	1.44166200	0.52938900	-0.81522500
C	2.35612000	1.62445900	-0.73870400
C	3.66427800	1.41377500	-0.23490400
C	4.03737700	0.08281900	0.20646700
C	3.08851300	-0.97067700	0.12026300
C	3.44694900	-2.25520100	0.58210400
C	4.69814700	-2.51037900	1.09606200
C	5.63998800	-1.47489100	1.16640800
C	5.31131300	-0.20890600	0.73310000
C	4.53514200	2.51943200	-0.17571400
C	4.13256000	3.77217000	-0.58688400
C	2.83336200	3.97648100	-1.07509400
C	1.96205000	2.91391400	-1.14825600
O	0.79217000	-2.91163600	-1.38608900
C	1.83466300	-3.03664800	-2.33315500
C	-1.76705900	-0.73500500	0.41459400
C	-1.44164000	0.52939000	0.81517400
C	-2.35608800	1.62447000	0.73867900
C	-3.66426400	1.41380000	0.23492000
C	-4.03739200	0.08284600	-0.20643500
C	-3.08853700	-0.97066000	-0.12025800
C	-3.44700300	-2.25518200	-0.58208400
C	-4.69822000	-2.51034700	-1.09600000
C	-5.64005100	-1.47484900	-1.16632000
C	-5.31134800	-0.20886600	-0.73302800
C	-4.53511800	2.51946600	0.17575400
C	-4.13251000	3.77220100	0.58690800
C	-2.83329500	3.97649900	1.07507700
C	-1.96199200	2.91392200	1.14821600
O	-0.79217400	-2.91164800	1.38601900
C	-1.83465300	-3.03666200	2.33310000
H	0.44533600	0.73951000	-1.20831800
H	2.70657000	-3.05449400	0.53383500
H	4.95324700	-3.50694700	1.44796200
H	6.63163600	-1.66738900	1.56893800
H	6.05804100	0.57739800	0.80666100

H	5.54631700	2.39492900	0.20278900
H	4.82718100	4.60681300	-0.52861200
H	2.51934600	4.96772500	-1.39284900
H	0.94716100	3.05051900	-1.52049900
H	2.34792900	-2.08341900	-2.50876500
H	1.40595900	-3.39414800	-3.27437500
H	2.56914600	-3.77198700	-1.98350500
H	-0.44530000	0.73950100	1.20823500
H	-2.70663100	-3.05448300	-0.53383500
H	-4.95334400	-3.50691400	-1.44788800
H	-6.63171500	-1.66733700	-1.56881700
H	-6.05806900	0.57744700	-0.80656800
H	-5.54630700	2.39497300	-0.20271700
H	-4.82712300	4.60685100	0.52865600
H	-2.51925800	4.96773900	1.39282100
H	-0.94709100	3.05051600	1.52042900
H	-1.40593700	-3.39418700	3.27430600
H	-2.34790000	-2.08342900	2.50873900
H	-2.56915300	-3.77198400	1.98345000

3) 2c-Phen

Number of imaginary frequencies = 0

E_{total} = -1356.966274 a.u

$G_{correction}$ = 0.399792 a.u

Cartesian coordinates:

B	0.73814000	-1.95163700	-0.05342900
B	-0.50929500	-1.72321200	1.08794600
C	-1.78865300	-0.94249700	0.58963500
C	-2.56731100	-1.53335700	-0.36393500
C	-3.76683500	-0.93628500	-0.86055200
C	-4.15588900	0.34551400	-0.39563700
C	-3.30873200	1.03003600	0.56311100
C	-2.14577700	0.37909100	1.05399800
C	-1.31497400	1.06025000	1.96997300
C	-1.61015600	2.33495500	2.39756200
C	-2.76176600	2.97441200	1.91856700
C	-3.58810700	2.33294400	1.02183300
C	-5.34998800	0.89751100	-0.89916100
C	-6.12002800	0.22118100	-1.82091200
C	-5.72639300	-1.04309400	-2.28361400
C	-4.56608000	-1.60818300	-1.80662600
O	-0.35780300	-2.15094500	2.36609000
C	-1.34427900	-2.00836600	3.36791700
C	1.76763100	-0.77058400	-0.22736400
C	1.25943300	0.46651600	-0.50626200
C	2.08081600	1.61881700	-0.69432100
C	3.48896900	1.50214600	-0.58173900
C	4.05841200	0.20866100	-0.25137700
C	3.19743900	-0.90746100	-0.07115100
C	3.75750800	-2.15090100	0.29051200
C	5.11529600	-2.30929000	0.45155700
C	5.96612600	-1.21323700	0.25483500
C	5.44417700	0.01577500	-0.08507400
C	4.26488200	2.66058800	-0.78211800
C	3.67839200	3.87458300	-1.07025300
C	2.28317500	3.98580800	-1.16738900
C	1.50011400	2.87001600	-0.98119500
O	0.71958200	-3.05621500	-0.84533800
C	1.57495300	-3.24464000	-1.95526100
H	-2.28366100	-2.50843500	-0.76661400
H	-0.41220300	0.56407700	2.32661200
H	-0.95171300	2.84400900	3.09691400
H	-3.00216400	3.98214600	2.24890500
H	-4.46756200	2.85670100	0.65651300
H	-5.68363500	1.87359900	-0.55704300
H	-7.03829800	0.67184600	-2.18985700
H	-6.33627900	-1.57194100	-3.01190100
H	-4.24529400	-2.59054700	-2.15054900
H	-2.33471900	-1.79864600	2.94592600
H	-1.07235700	-1.19188600	4.04752600
H	-1.38863800	-2.93816700	3.94341700
H	0.18009900	0.60398400	-0.60053800
H	3.08882300	-2.99564100	0.45516000
H	5.52470100	-3.27616300	0.73384400
H	7.04035800	-1.32851100	0.37817000
H	6.12512800	0.85224000	-0.21896000
H	5.34808300	2.60894000	-0.70975700
H	4.30346400	4.75179000	-1.22006400
H	1.82660800	4.94743600	-1.38857600
H	0.41438900	2.93297000	-1.04802700
H	0.99120100	-3.68785200	-2.76803600
H	2.01476200	-2.30134200	-2.30088400
H	2.38631400	-3.93454700	-1.69414100

4) 2a-Pyr

Number of imaginary frequencies = 0

E_{total} = -1509.329383 a.u

$G_{correction}$ = 0.429119 a.u

Cartesian coordinates:

B	-0.71953300	2.99576400	-0.49282700
B	0.71949400	2.99576900	0.49281200
C	1.54680800	1.67867700	0.74577600
C	2.77180100	1.59370100	0.14144700
C	3.45356100	0.35234900	-0.06626000
C	2.83327100	-0.84374400	0.38247100
C	1.59105200	-0.78071700	1.07038500
C	0.95750200	0.47322900	1.28636300
C	-0.25069200	0.49816000	1.99121500
C	-0.85703800	-0.67598100	2.42354900
C	-0.26069300	-1.90454700	2.17694900
C	0.96501100	-1.97945500	1.50745600
C	1.60411100	-3.23204000	1.23200100
C	2.78890100	-3.29060000	0.57376500
C	3.44748700	-2.09899000	0.12646300
C	4.66524800	-2.13148000	-0.56151100
C	5.27045600	-0.95701600	-0.99142800
C	4.67240500	0.27331800	-0.74824400
O	1.13060600	4.21705600	0.90906100
C	2.23293700	4.44577400	1.75967500
O	-1.13067200	4.21704900	-0.90905400
C	-2.23301800	4.44575700	-1.75965200
C	-1.54683000	1.67866200	-0.74579300
C	-2.77181600	1.59367100	-0.14145000
C	-3.45355400	0.35231000	0.06627200
C	-2.83325400	-0.84377500	-0.38246800
C	-1.59104700	-0.78073200	-1.07039900

C	-0.95751500	0.47322200	-1.28638500
C	0.25067200	0.49816900	-1.99124800
C	0.85702700	-0.67596500	-2.42359100
C	0.26070100	-1.90453900	-2.17698300
C	-0.96499500	-1.97946300	-1.50747600
C	-1.60407400	-3.23205500	-1.23201200
C	-2.78885500	-3.29063000	-0.57375900
C	-3.44744900	-2.09902900	-0.12644800
C	-4.66519600	-2.13153500	0.56155100
C	-5.27041100	-0.95707900	0.99147800
C	-4.67238300	0.27326300	0.74827900
H	3.23323400	2.48945800	-0.28329900
H	-0.73498000	1.45540800	2.18121200
H	-1.81532400	-0.63208200	2.93760000
H	-0.74752500	-2.82606600	2.49323700
H	1.10799500	-4.14304000	1.56445200
H	3.26503200	-4.24882900	0.37051500
H	5.13415000	-3.09468200	-0.75765800
H	6.21670200	-1.00152600	-1.52560800
H	5.14308800	1.19217500	-1.09524800
H	3.05004300	4.90315000	1.18917000
H	1.92914100	5.14775300	2.54307900
H	2.59946500	3.52049800	2.22048200
H	-1.92924800	5.14776500	-2.54304100
H	-2.59952300	3.52048300	-2.22047800
H	-3.05013100	4.90309600	-1.18912900
H	-3.23325400	2.48942300	0.28330000
H	0.73494600	1.45542300	-2.18124900
H	1.81530400	-0.63205400	-2.93765800
H	0.74754300	-2.82605200	-2.49327500
H	-1.10795100	-4.14304900	-1.56447000
H	-3.26497200	-4.24886500	-0.37050400
H	-5.13408100	-3.09474300	0.75770700
H	-6.21664600	-1.00160100	1.52567700
H	-5.14307200	1.19211400	1.09529000

5) 2b- Pyr

Number of imaginary frequencies = 0

E_{total} = -1509.326131 a.u

$G_{correction}$ = 0.426648 a.u

Cartesian coordinates:

B	-0.68445600	-2.03123400	0.51495200
B	0.68445600	-2.03123600	-0.51500600
C	1.76260300	-0.88406300	-0.45491600
C	1.43795900	0.37667900	-0.87246700
C	2.36516500	1.46706200	-0.84358000
C	3.67626000	1.23201700	-0.35222500
C	4.03865500	-0.06541100	0.10183400
C	3.09351400	-1.12743700	0.05927000
C	3.47889700	-2.38619900	0.53797100
C	4.75988800	-2.60513800	1.03034700
C	5.68830100	-1.57367100	1.06068200
C	5.34829900	-0.29604000	0.60499900
C	6.27868400	0.79367900	0.63292200
C	5.93058900	2.03033400	0.19782000
C	4.61641900	2.29629400	-0.30681500
C	4.22846300	3.56484100	-0.75071500
C	2.94253600	3.78821100	-1.22691000
C	2.01816000	2.75210700	-1.27360600
O	0.77973600	-3.07358900	-1.37952800
C	1.80822300	-3.20833000	-2.34064000
O	-0.77972600	-3.07359000	1.37947300
C	-1.80819700	-3.20832800	2.34060300
C	-1.76260100	-0.88405900	0.45487700
C	-1.43794100	0.37668500	0.87241100
C	-2.36514600	1.46707100	0.84354100
C	-3.67625500	1.23202600	0.35222500
C	-4.03866600	-0.06540400	-0.10181600
C	-3.09352700	-1.12743200	-0.05927000
C	-3.47892700	-2.38619600	-0.53795400
C	-4.75993300	-2.60513400	-1.03029400
C	-5.68834300	-1.57366500	-1.06060900
C	-5.34832500	-0.29603200	-0.60494200
C	-6.27870800	0.79369000	-0.63284700
C	-5.93059700	2.03034600	-0.19776200
C	-4.61641200	2.29630500	0.30683400
C	-4.22843900	3.56485400	0.75071500
C	-2.94249900	3.78822300	1.22687300
C	-2.01812500	2.75211700	1.27355100
H	0.43412900	0.58523800	-1.24741100
H	2.75600700	-3.20241600	0.53000200
H	5.03485700	-3.59137300	1.39668400
H	6.69211800	-1.74674300	1.44570300
H	7.27961800	0.60304200	1.01736600
H	6.64656500	2.85037200	0.22641100
H	4.95071400	4.37905500	-0.71530500
H	2.65745000	4.78159700	-1.56525300
H	1.00941200	2.92848800	-1.64434400
H	2.55762200	-3.92621500	-1.98657900
H	1.36813300	-3.59273200	-3.26582500
H	2.30671700	-2.25346000	-2.54704900
H	-1.36809100	-3.59273500	3.26577900
H	-2.30668200	-2.25345600	2.54702300
H	-2.55760500	-3.92621000	1.98655400
H	-0.43410100	0.58524300	1.24732700
H	-2.75604000	-3.20241600	-0.53000100
H	-5.03491400	-3.59137000	-1.39661700
H	-6.69217200	-1.74673600	-1.44560100
H	-7.27965200	0.60305300	-1.01726100
H	-6.64657100	2.85038600	-0.22633800
H	-4.95068900	4.37907000	0.71532100
H	-2.65740100	4.78161100	1.56520300
H	-1.00936700	2.92849700	1.64426100

6) 2c- Pyr

Number of imaginary frequencies = 0

E_{total} = -1509.327186 a.u

$G_{correction}$ = 0.430057 a.u

Cartesian coordinates:

B	-0.93018100	2.77680600	-0.75729700
B	0.45844700	3.12058100	0.23564100
C	1.44954400	1.94158300	0.56931300
C	2.66070400	1.94196300	-0.06778900
C	3.47265500	0.76828900	-0.19800200
C	2.99044600	-0.45227500	0.34583900
C	1.75666600	-0.47354200	1.05234000

C	1.00385800	0.72129200	1.20655600
C	-0.18643100	0.66965600	1.93801500
C	-0.66689700	-0.53027000	2.45019200
C	0.04539600	-1.70666600	2.26491800
C	1.26726300	-1.69824300	1.58262000
C	2.03543200	-2.89071500	1.38358600
C	3.21796800	-2.86712900	0.71872900
C	3.74079300	-1.64706000	0.17994200
C	4.95895300	-1.59358400	-0.50700600
C	5.43064000	-0.39454800	-1.02565000
C	4.69510000	0.77517600	-0.87750400
O	0.69514900	4.41685700	0.54080600
C	1.76903900	4.86223500	1.34132100
O	-1.56209400	3.83507700	-1.31577800
C	-2.63158400	3.71575400	-2.23120000
C	-1.35797700	1.27373400	-0.98193900
C	-0.48202900	0.41488100	-1.58152400
C	-0.64465000	-1.00577400	-1.56255500
C	-1.76763000	-1.55085400	-0.88678900
C	-2.71464000	-0.68345700	-0.27867300
C	-2.52645600	0.72596800	-0.32694600
C	-3.44712700	1.54430100	0.34121500
C	-4.52766700	1.00266200	1.02775600
C	-4.71487800	-0.37187600	1.07241500
C	-3.81603900	-1.23246300	0.43348100
C	-3.95340600	-2.65729900	0.50217500
C	-3.04455100	-3.48221800	-0.07538500
C	-1.91329400	-2.95999300	-0.78184400
C	-0.94018400	-3.78901300	-1.34983200
C	0.15933600	-3.24778300	-2.00441800
C	0.30791800	-1.87081700	-2.11119000
H	3.01418700	2.84812300	-0.56712000
H	-0.76456000	1.58309900	2.08024000
H	-1.61922300	-0.54635400	2.97660800
H	-0.34139500	-2.65110100	2.64550700
H	1.64202900	-3.82489300	1.78203100
H	3.79467700	-3.78024300	0.57852100
H	5.53449100	-2.50981000	-0.63080500
H	6.37946600	-0.37212700	-1.55656800
H	5.06097000	1.71198300	-1.29552200
H	2.50005900	5.38818000	0.71597500
H	1.38299100	5.56888400	2.08312600
H	2.27304500	4.03364400	1.85366200
H	-2.41500200	4.33746000	-3.10585600
H	-2.78401300	2.67791900	-2.55269700
H	-3.55400800	4.08420400	-1.76724100
H	0.43982900	0.79811300	-2.02609400
H	-3.30283000	2.62477600	0.33114300
H	-5.22700800	1.66106600	1.53787600
H	-5.55715600	-0.79685500	1.61643100
H	-4.80638700	-3.06441700	1.04340300
H	-3.15462700	-4.56364600	-0.00698200
H	-1.05295100	-4.86891300	-1.26455800
H	0.91457600	-3.90594000	-2.42754200
H	1.17945300	-1.44704200	-2.60847700

Computed structures and energies of the two rotational isomers of 7-Phen

*Note: the computed structures and energies of both isomers of **7-Phen** include the implicit THF solvation.*

7) 7a-Phen

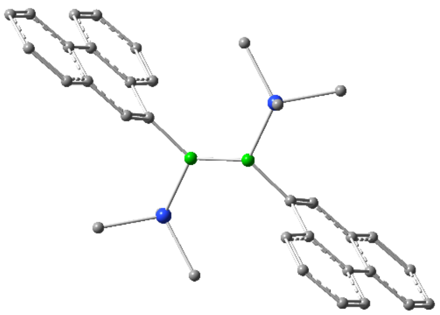

Note: Hydrogen atoms are omitted for clarity.

Number of imaginary frequencies = 0

E_{total} = -2048.730622 a.u

$G_{correction}$ = 0.534094 a.u

Cartesian coordinates:

B	-0.60000000	0.28484400	-0.42318300
P	-0.16854000	1.25884100	-2.01864200
C	-0.06414300	3.08253300	-1.90412700
H	0.19321900	3.51669400	-2.87808200
H	0.70374200	3.35554200	-1.17136000
H	-1.02626000	3.48853400	-1.57283800
C	1.40404300	0.81410300	-2.82221800
H	1.49395400	1.35626000	-3.77091400
H	1.42861700	-0.26313900	-3.01634300
H	2.24873900	1.07588600	-2.17717500
C	-1.40365100	1.01843100	-3.33987800
H	-1.11673000	1.57223500	-4.24134800
H	-2.38368500	1.37142800	-2.99959200
H	-1.48285500	-0.04758900	-3.58036600
C	-2.17366600	0.12625500	-0.24703000
C	-3.02135000	1.18294400	0.28186200
C	-2.44249200	2.38669100	0.74169700
H	-1.35489000	2.46193000	0.71857800
C	-3.20788500	3.42114500	1.23461300
H	-2.73399300	4.33597300	1.58407500
C	-4.60170700	3.28218600	1.29260100
H	-5.21693500	4.09295500	1.67692100
C	-5.19525700	2.10954300	0.87598400
H	-6.27665100	2.02011200	0.94499600
C	-4.43464800	1.03427600	0.37102400
C	-5.04846900	-0.21285100	-0.04599100
C	-6.43616800	-0.45440500	0.01721200
H	-7.10676600	0.31637100	0.38948400
C	-6.97320700	-1.66008300	-0.38383800
H	-8.04743600	-1.81981600	-0.32301200
C	-6.13888000	-2.67965200	-0.86915700
H	-6.56559800	-3.62909300	-1.18511200
C	-4.78022600	-2.46951600	-0.94311300
H	-4.11880700	-3.24955300	-1.31925900
C	-4.20874300	-1.24486700	-0.53724800
C	-2.79744800	-1.03146800	-0.62996100
H	-2.19829700	-1.84761900	-1.04126000
B	0.60001200	-0.28485000	0.42322400
P	0.16854300	-1.25888000	2.01866300
C	0.06415000	-3.08256900	1.90412900
H	-0.19322600	-3.51673500	2.87807800
H	-0.70371600	-3.35558500	1.17134600
H	1.02627900	-3.48855700	1.57286000
C	-1.40406000	-0.81413800	2.82220100
H	-1.49406400	-1.35638400	3.77083800
H	-1.42857300	0.26308700	3.01643300
H	-2.24873100	-1.07580600	2.17707900
C	1.40362000	-1.01848300	3.33993300
H	1.11669200	-1.57232700	4.24137600
H	2.38367100	-1.37144100	2.99965600
H	1.48279300	0.04753000	3.58046200
C	2.17367300	-0.12625100	0.24705600
C	3.02135700	-1.18294100	-0.28183400
C	2.44250400	-2.38670200	-0.74163600
H	1.35490300	-2.46195400	-0.71849400
C	3.20789800	-3.42115600	-1.23455100
H	2.73401000	-4.33599500	-1.58399000
C	4.60171700	-3.28218200	-1.29257200
H	5.21694600	-4.09295000	-1.67689400
C	5.19526300	-2.10952600	-0.87598600
H	6.27665400	-2.02008300	-0.94502100
C	4.43465200	-1.03426000	-0.37102600
C	5.04846900	0.21287800	0.04596100
C	6.43616400	0.45444500	-0.01727300
H	7.10676200	-0.31632900	-0.38955100
C	6.97319900	1.66013200	0.38375200
H	8.04742600	1.81987500	0.32290200
C	6.13887300	2.67969800	0.86907800
H	6.56558900	3.62914600	1.18501400
C	4.78022200	2.46954900	0.94306600
H	4.11880400	3.24958400	1.31921600
C	4.20874300	1.24489000	0.53722500
C	2.79745100	1.03147900	0.62996700
H	2.19829800	1.84763300	1.04125800

8) 7b-Phen

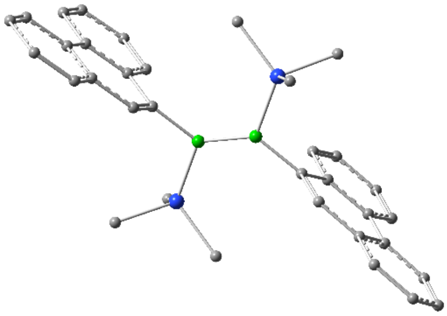

Note: Hydrogen atoms are omitted for clarity.

Number of imaginary frequencies = 0

E_{total} = -2048.730373 a.u

$G_{correction}$ = 0.534494 a.u

Cartesian coordinates:

B	-0.59161000	0.16715400	-0.46248700
P	-0.14488000	1.07703200	-2.09073900
C	-0.14952900	2.90769300	-2.06988200
H	0.11989400	3.30266500	-3.05716400
H	0.57324600	3.26755700	-1.32846800
H	-1.14575400	3.27332200	-1.79779600
C	1.48481200	0.68876000	-2.80431100
H	1.58905500	1.20416400	-3.76641900
H	1.57417100	-0.39113200	-2.95975000
H	2.28706800	1.01606000	-2.13491200
C	-1.30576300	0.70143900	-3.44738500
H	-1.01113800	1.22493100	-4.36437100
H	-2.31660300	1.01597300	-3.16419600
H	-1.31777500	-0.37808100	-3.63420600
C	-2.16523600	-0.03438400	-0.32044200
C	-3.06777600	1.00774300	0.14151900
C	-2.55251900	2.25790100	0.55034100
H	-1.47010100	2.38640600	0.53304300
C	-3.37123300	3.27842200	0.98286000
H	-2.94466000	4.23007600	1.29267400
C	-4.75794900	3.07804200	1.03049900
H	-5.41508900	3.87659400	1.36808100
C	-5.29080800	1.86142000	0.66116000
H	-6.36805800	1.72610200	0.71843800
C	-4.47465600	0.79994000	0.21727100
C	-5.02659700	-0.48995400	-0.15262200
C	-6.40298000	-0.79102300	-0.09614400
H	-7.11250000	-0.03754900	0.23719700
C	-6.88024200	-2.03483400	-0.45379400
H	-7.94707900	-2.23997100	-0.39933800
C	-5.99487300	-3.03483300	-0.88677200
H	-6.37467800	-4.01439400	-1.16886000
C	-4.64587800	-2.76745800	-0.95171500
H	-3.94513100	-3.53193900	-1.28628700
C	-4.13507600	-1.50280600	-0.58939900
C	-2.73291300	-1.23240400	-0.66619200
H	-2.09089100	-2.04052900	-1.02518300
B	0.59429300	-0.35275300	0.43386700
P	0.15470300	-1.23706800	2.07191200
C	0.27651600	-3.06146400	2.04771600
H	0.03291900	-3.48353700	3.03049800
H	-0.41652000	-3.45953100	1.29796700
H	1.29598200	-3.35633200	1.77508900
C	-1.50042800	-0.93415800	2.76605700
H	-1.57691300	-1.42214400	3.74481300
H	-1.65811200	0.14285500	2.88631400
H	-2.27509300	-1.33228900	2.10360200
C	1.27154400	-0.78848000	3.44483100
H	1.01572400	-1.35300600	4.34922600
H	2.30735700	-1.01171900	3.16609800
H	1.18827300	0.28346200	3.65487900
C	2.17604100	-0.33357900	0.23422400
C	2.99983400	0.83282100	0.51659200
C	2.40833800	2.01931300	1.00528200
H	1.32943400	2.01301100	1.16090300
C	3.15151400	3.14846300	1.27317900
H	2.66684700	4.04607500	1.65159500
C	4.53660600	3.13227200	1.05798200
H	5.13362800	4.01776300	1.26545200
C	5.14530300	1.99031500	0.58317400
H	6.22072800	2.00104200	0.42392400
C	4.40848100	0.82055200	0.30177100
C	5.04588500	-0.38398600	-0.19639000
C	6.43070700	-0.48510000	-0.44263900
H	7.08000400	0.36753100	-0.25895800
C	6.99148000	-1.65285400	-0.91629400
H	8.06285000	-1.70264000	-1.09759800
C	6.18488800	-2.77486500	-1.16532800
H	6.63023000	-3.69402700	-1.53970100
C	4.82978500	-2.70341600	-0.93345800
H	4.18925800	-3.56443200	-1.12260000
C	4.23471200	-1.51944100	-0.44786000
C	2.82544000	-1.45135800	-0.21904200
H	2.24409100	-2.34599400	-0.45724300

Frontier orbitals and associated energies of diborenes 7-Phen, 7-Pyr and III

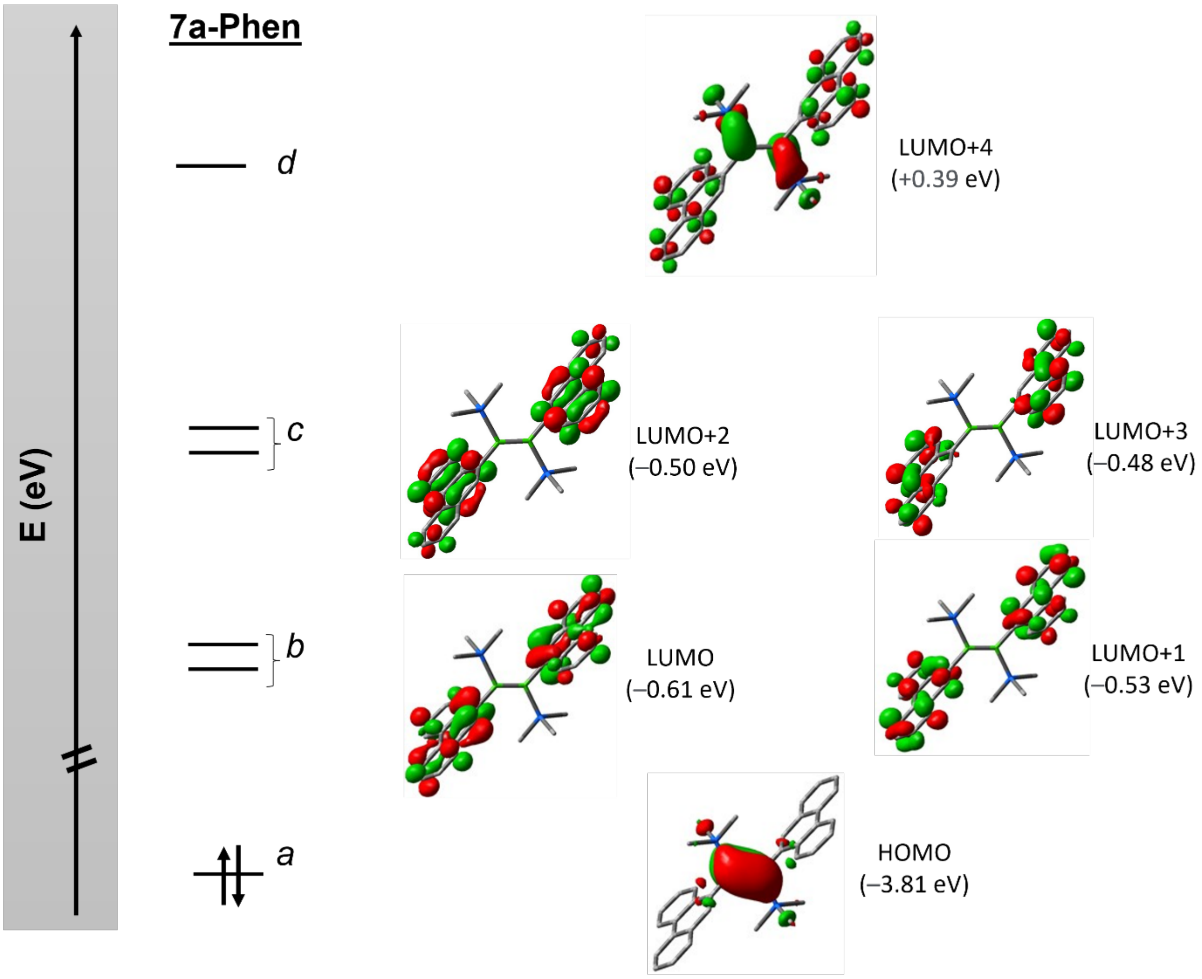

Figure S76. Frontier orbitals (isovalue: ±0.04) of diborene **7a-Phen** showing the orbital intercalation between PAH and diborene units. ^{a} π orbital of the B=B bond; $^{b\ and\ c}$ π* orbitals of PAH units; and ^{d} π* orbital of the B=B bond.

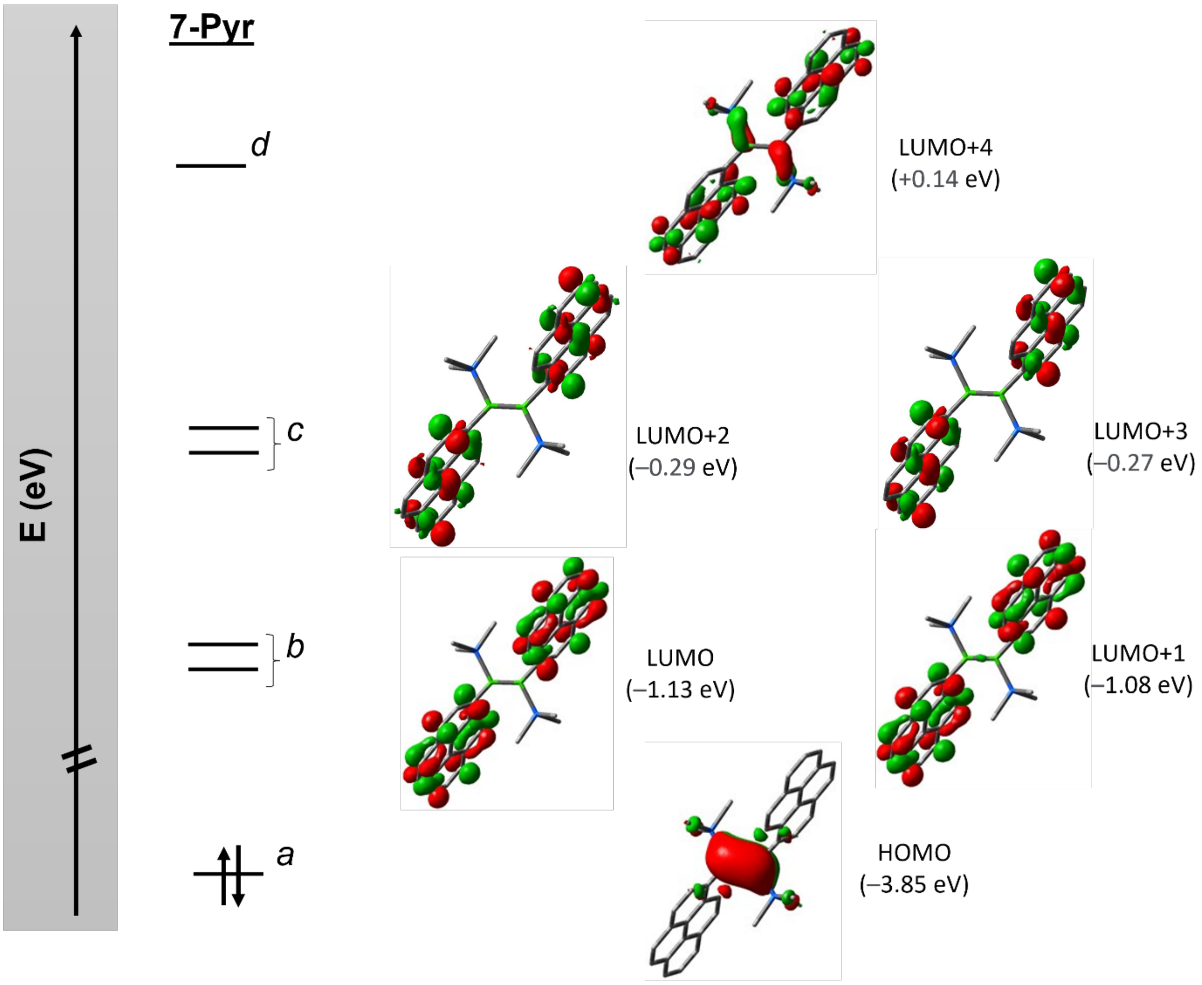

Figure S77. Frontier orbitals (isovalue: ±0.04) of diborene **7a-Pyr** showing the orbital intercalation between PAH and diborene units. [a] π orbital of the B=B bond; [b and c] π* orbitals of PAH units; and [d] π* orbital of the B=B bond.

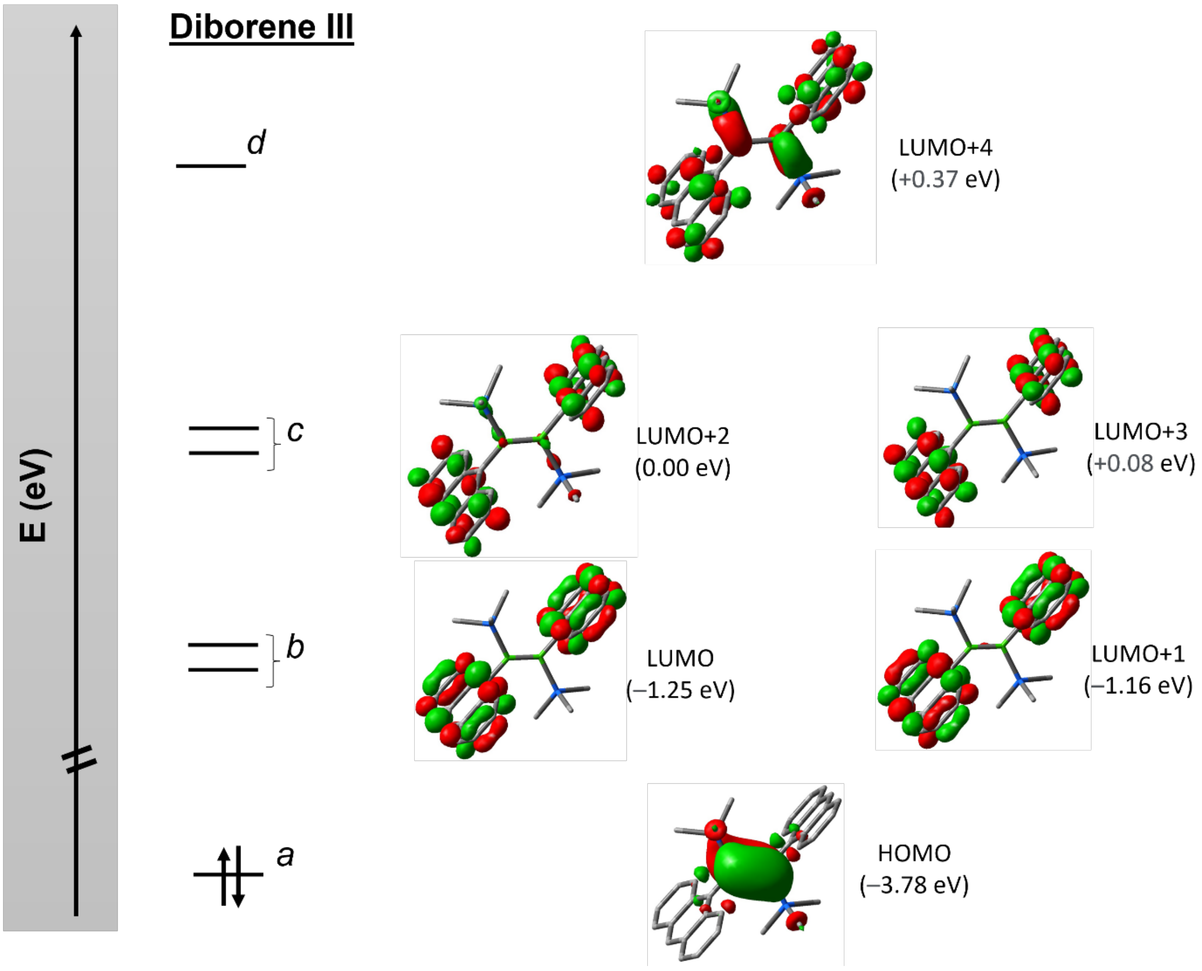

Figure S78. Frontier orbitals (isovalue: ±0.04) of diborene **III** showing the orbital intercalation between PAH and diborene units. [a] π orbital of the B=B bond; [b and c] π* orbitals of PAH units; and [d] π* orbital of the B=B bond.

Mechanistic pathways for the hydrogenation of 7a-Phen

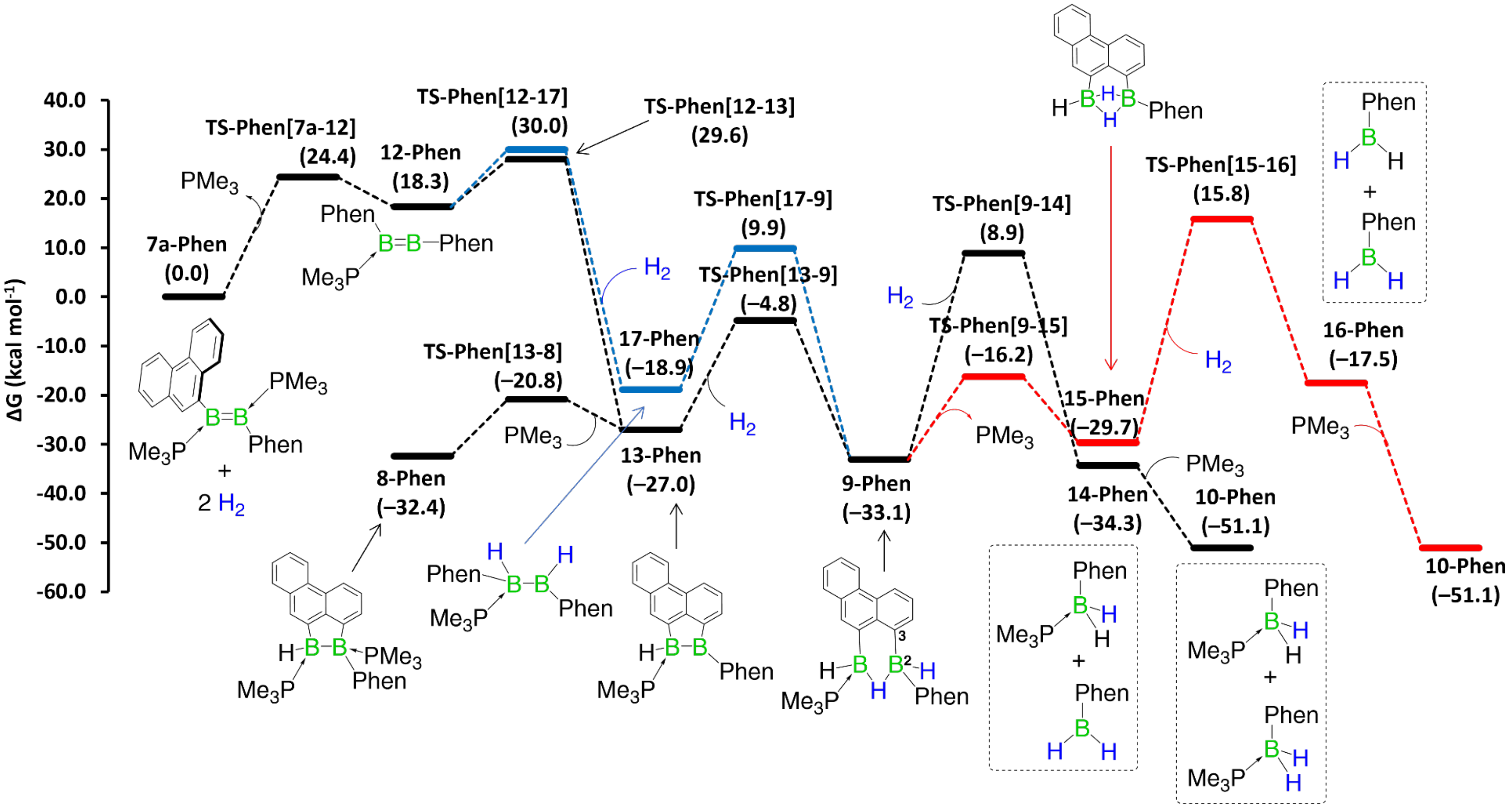

Figure S79. Computed mechanism for the formation of the borane **10-Phen** from the hydrogenation of diborene **7a-Phen**.

Structures, energies and cartesian coordinates involving 7a-Phen in the hydrogenation:

9) 7a-Phen

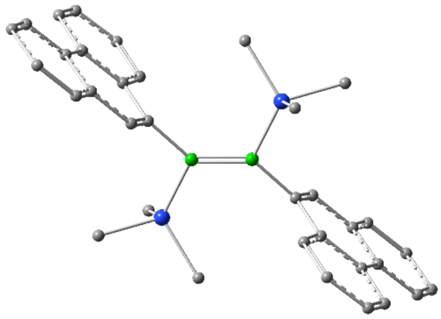

Note: Hydrogen atoms are omitted for clarity.

Number of imaginary frequencies = 0

E_{total} = -2048.728537 a.u

$G_{correction}$ = 0.534399 a.u

Cartesian coordinates:

B	0.60497800	0.26762700	0.42477600
B	-0.60497500	-0.26787900	-0.42468600
P	0.18051900	1.20572000	2.03781500
P	-0.18048700	-1.20600300	-2.03770400
C	-1.40598000	0.77887000	2.82466400
H	-2.24103100	1.06993000	2.17987700
H	-1.45649900	-0.30158600	2.99284200
H	-1.49356900	1.30060100	3.78509900
C	1.40262800	0.91357900	3.36356600
H	1.46002400	-0.15875600	3.58020100
H	2.39236400	1.24981800	3.03501800
H	1.12644200	1.45262700	4.27744800
C	0.11478800	3.03640100	1.97206800
H	1.08411900	3.43032900	1.64711500
H	-0.64721000	3.34285200	1.24644300
H	-0.13244700	3.45721500	2.95471100
C	-1.40264500	-0.91397100	-3.36342600
H	-2.39236300	-1.25018100	-3.03479400
H	-1.46005500	0.15834500	-3.58015000
H	-1.12650400	-1.45309600	-4.27727400
C	-0.11465900	-3.03667800	-1.97190900
H	0.64733200	-3.34306700	-1.24625700
H	-1.08398400	-3.43062800	-1.64696700
H	0.13262100	-3.45752000	-2.95453000
C	1.40596400	-0.77908900	-2.82462100
H	1.45649700	0.30138700	-2.99266900
H	2.24104700	-1.07024300	-2.17992100
H	1.49348900	-1.30071300	-3.78512200
C	-2.17725800	-0.11391700	-0.24141000
C	-2.80457900	1.04602600	-0.61012300
C	-3.02000100	-1.18012400	0.27458700
C	-4.21655100	1.25236500	-0.51610700
H	-2.20492400	1.86742700	-1.01004200
C	-4.43379200	-1.03941100	0.36225400
C	-2.43418000	-2.38438700	0.72360200
C	-4.79318600	2.47930200	-0.90575400
C	-5.05211700	0.21019500	-0.03962700
C	-5.18839200	-2.12555500	0.85154900
H	-1.34545100	-2.44741600	0.70443800
C	-3.19473300	-3.42851000	1.20239800
C	-6.15218400	2.68307100	-0.83054600
H	-4.13464600	3.26693200	-1.27088400
C	-6.44017900	0.44611100	0.02540300
C	-4.58925200	-3.29898100	1.25622100
H	-6.27052500	-2.04397000	0.91774400
H	-2.71659200	-4.34386400	1.54469100
C	-6.98221300	1.65409000	-0.35970800
H	-6.58242400	3.63486600	-1.13419900
H	-7.10753400	-0.33212000	0.38763200
H	-5.20091800	-4.11790400	1.62871100
H	-8.05708800	1.80834300	-0.29728400
C	2.17726600	0.11381000	0.24140700
C	2.80475600	-1.04596700	0.61035500
C	3.01986600	1.18006400	-0.27473600
C	4.21674800	-1.25214700	0.51630700
H	2.20523500	-1.86734400	1.01052400
C	4.43366900	1.03950200	-0.36245900
C	2.43388200	2.38419100	-0.72390400
C	4.79355800	-2.47893100	0.90617800
C	5.05216200	-0.20996700	0.03958300
C	5.18811800	2.12566200	-0.85195200
H	1.34514800	2.44709600	-0.70467300
C	3.19428700	3.42832700	-1.20290400
C	6.15257700	-2.68254600	0.83094400
H	4.13513500	-3.26657000	1.27150100
C	6.44025200	-0.44571900	-0.02545800
C	4.58882000	3.29895600	-1.25677100
H	6.27025900	2.04420300	-0.91817300
H	2.71602100	4.34357200	-1.54531100
C	6.98245500	-1.65355500	0.35986200
H	6.58295100	-3.63422500	1.13477000
H	7.10749400	0.33253100	-0.38785400
H	5.20037300	4.11789800	-1.62940500
H	8.05734700	-1.80768200	0.29742300

10) TS-Phen[7a-12]

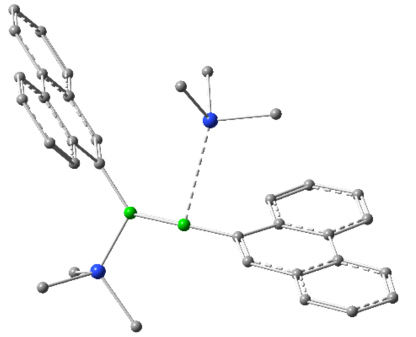

Note: Hydrogen atoms are omitted for clarity.

Number of imaginary frequencies = 1 (29.5 cm^{-1})

E_{total} = -2048.688266 a.u

$G_{correction}$ = 0.532994 a.u

Cartesian coordinates:

B	0.79140900	-0.82807800	-0.48053000
B	-0.67739200	-1.21685400	-0.68713600
P	0.15290300	1.58411900	1.14146000
P	-0.76396600	-2.76562900	-1.75336500
C	-0.68982700	1.45088500	2.78277200
H	-1.73045500	1.13757400	2.63860000
H	-0.19375700	0.69219000	3.39889800
H	-0.68018500	2.40820500	3.32153400
C	1.67989800	2.48840200	1.66618700
H	2.34261700	1.81131300	2.21897000
H	2.22400600	2.84848400	0.78442400
H	1.44176800	3.34721600	2.30814700
C	-0.81143500	2.99467200	0.43477800
H	-0.39093100	3.28626300	-0.53510700
H	-1.85018700	2.68214200	0.27268500
H	-0.80625900	3.86956800	1.09930400
C	-1.63131600	-2.57906100	-3.35246900
H	-2.65618900	-2.23712300	-3.16973800
H	-1.11696900	-1.82290100	-3.95548500
H	-1.66426200	-3.52655800	-3.90411700
C	-1.63046600	-4.19704700	-1.01488000
H	-1.11751200	-4.49759800	-0.09489600
H	-2.65496600	-3.90417200	-0.75780800
H	-1.66528400	-5.04761400	-1.70673200
C	0.86439100	-3.43421000	-2.21985800
H	1.42360300	-2.69110000	-2.79807100
H	1.43785600	-3.68703900	-1.32186300
H	0.73948900	-4.33773600	-2.82720200
C	-2.06689900	-0.55414700	-0.31672900
C	-2.67821300	0.28907000	-1.20367500
C	-2.75410500	-0.81576900	0.93512200
C	-3.94429300	0.90840000	-0.96453700
H	-2.17825300	0.52228100	-2.14698500
C	-4.01179900	-0.21545500	1.22785900
C	-2.16670800	-1.66128900	1.90129400
C	-4.51657400	1.77035700	-1.92316500
C	-4.62981300	0.66328600	0.25250500
C	-4.61301500	-0.49162900	2.47366600
H	-1.18979400	-2.08337500	1.66552500
C	-2.77861900	-1.91662700	3.10814900
C	-5.73471600	2.37123000	-1.70165100
H	-3.96997800	1.95012400	-2.84837600
C	-5.87705900	1.28960400	0.45067500
C	-4.01575700	-1.32364200	3.39590800
H	-5.56970800	-0.03957400	2.72340100
H	-2.30177800	-2.56763200	3.83780000
C	-6.42072600	2.12455400	-0.50246300
H	-6.16495500	3.03313000	-2.44989800
H	-6.43214600	1.11769600	1.36944500
H	-4.50595800	-1.51487200	4.34809500
H	-7.38501400	2.59417600	-0.32173800
C	2.26285200	-0.43874300	-0.27336200
C	3.05237300	-1.19156100	0.55614800
C	2.86888700	0.69594500	-0.94965400
C	4.42867500	-0.90070400	0.79874300
H	2.61711700	-2.04482000	1.07863100
C	4.22307800	1.04783600	-0.70755200
C	2.10569800	1.48463700	-1.83466100
C	5.19139400	-1.71338000	1.66218400
C	5.02698200	0.22884500	0.18299400
C	4.74197900	2.19351100	-1.34479400
H	1.07401000	1.18283500	-2.01995400
C	2.63801800	2.59949900	-2.44437200
C	6.51298200	-1.42900000	1.91929300
H	4.70854100	-2.57342100	2.12448300
C	6.37982300	0.49705400	0.46794200
C	3.96851900	2.95996600	-2.19003600
H	5.77351000	2.48876900	-1.16995300
H	2.03004200	3.19569800	-3.12131600
C	7.10851800	-0.31182800	1.31488400
H	7.09264300	-2.06257700	2.58635100
H	6.87068200	1.35467700	0.01560200
H	4.39596800	3.84092700	-2.66359200
H	8.15219000	-0.07981000	1.51437600

11) PMe_3

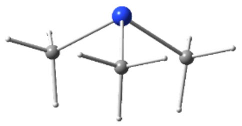

Number of imaginary frequencies = 0

E_{total} = -460.969828 a.u

$G_{correction}$ = 0.082968 a.u

Cartesian coordinates:

P	0.41161400	0.45489400	0.00000000
C	-1.39234200	0.87853800	0.00000000
H	-1.63263900	1.47872400	0.88507600
H	-1.63263900	1.47872400	-0.88507600
H	-2.02602800	-0.01863300	0.00000000
C	0.41161400	-0.75372800	-1.40474600
H	0.24970500	-0.22381800	-2.35043000
H	1.38554500	-1.25288000	-1.46494700
H	-0.36935700	-1.51765900	-1.29091900
C	0.41161400	-0.75372800	1.40474600
H	1.38554500	-1.25288000	1.46494700
H	0.24970500	-0.22381800	2.35043000
H	-0.36935700	-1.51765900	1.29091900

12) H_2

Number of imaginary frequencies = 0

E_{total} = -1.169087 a.u

$G_{correction}$ = -0.001363 a.u

Cartesian coordinates:

H	0.00000000	0.00000000	0.37121100
H	0.00000000	0.00000000	-0.37121100

13) 12-Phen

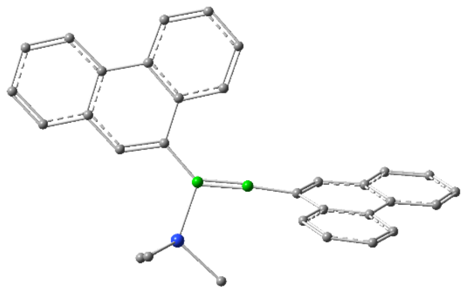

Note: Hydrogen atoms are omitted for clarity.

Number of imaginary frequencies = 0

E_{total} = -1587.706122 a.u

$G_{correction}$ = 0.428063 a.u

Cartesian coordinates:

B	0.82794800	0.86721700	-0.34172100
B	-0.63829000	0.44450500	-0.20766500
P	0.74891100	2.66263900	-0.93094400
C	1.40418200	3.94102600	0.20310400
H	0.82333600	3.91439500	1.13194200
H	2.45025200	3.73823900	0.45260300
H	1.32752500	4.93971000	-0.24363400
C	1.59766100	3.00882500	-2.51286900
H	2.65502200	2.73402900	-2.44952100
H	1.13463200	2.40088100	-3.29737400
H	1.51524000	4.06983500	-2.77753800
C	-0.94443700	3.25698300	-1.24965100
H	-1.43015800	2.63770200	-2.01029700
H	-1.54700600	3.23645500	-0.33492900
H	-0.89639400	4.29060200	-1.61124000
C	2.20661900	0.16209800	-0.12320600
C	3.39711400	0.84041600	-0.21577500
C	2.29611400	-1.25787200	0.20149300
C	4.67922600	0.25110800	-0.01801900
H	3.40184200	1.90753700	-0.44751800
C	3.55234700	-1.89202600	0.41801100
C	1.13423000	-2.04700800	0.31535100
C	5.85498700	1.02295100	-0.13422200
C	4.77969600	-1.12561600	0.30279500
C	3.57234700	-3.26362600	0.74364800
H	0.16501300	-1.58411200	0.13455000
C	1.17737400	-3.38558000	0.63408300
C	7.09698300	0.46174900	0.05656400
H	5.75860900	2.08054500	-0.37952200
C	6.06318100	-1.67351400	0.49115300
C	2.41496600	-4.00176300	0.85375900
H	4.52334100	-3.76031100	0.91690000
H	0.25579100	-3.95768500	0.71339800
C	7.20040100	-0.90153600	0.37157700
H	7.99411000	1.06996200	-0.03640300
H	6.17581000	-2.72695300	0.73472100
H	2.46552400	-5.05814400	1.10800400
H	8.17833300	-1.35304300	0.52218400
C	-2.11891900	0.10185200	-0.14561800
C	-2.59548000	-0.83928400	-1.02592900
C	-3.03890900	0.66509100	0.82801400
C	-3.94338000	-1.30085800	-1.01129600
H	-1.92220300	-1.26057300	-1.77382000
C	-4.40357700	0.27331600	0.84165100
C	-2.59123600	1.61341900	1.76948800
C	-4.36963000	-2.28832400	-1.92348100
C	-4.86695800	-0.73866700	-0.09179800
C	-5.26412300	0.87697100	1.78067600
H	-1.52966800	1.86495200	1.77313000
C	-3.45291100	2.18446600	2.67954200
C	-5.67512100	-2.72122000	-1.93598200
H	-3.63977300	-2.70086100	-2.61851100
C	-6.19690800	-1.20008400	-0.13095500
C	-4.80513400	1.81726800	2.67745600
H	-6.31464000	0.60022000	1.80841500
H	-3.08432500	2.90932300	3.40158400
C	-6.59268500	-2.16866700	-1.02954900
H	-5.99597000	-3.48429000	-2.64067200
H	-6.93450300	-0.79623200	0.55718000
H	-5.49339900	2.26464000	3.39075800
H	-7.62642400	-2.50638300	-1.03340200

14) TS-Phen[12-13]

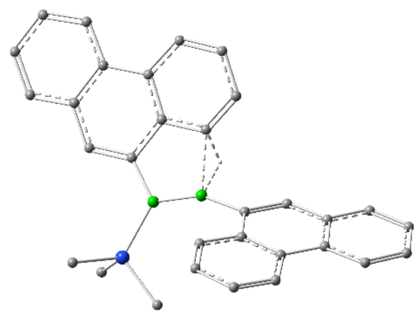

Note: Selected hydrogen atoms are omitted for clarity.

Number of imaginary frequencies = 1 (283.0 cm^{-1})

E_{total} = -1587.688834 a.u

$G_{correction}$ = 0.428695 a.u

Cartesian coordinates:

B	0.89890600	1.03908900	-0.53896000
B	-0.33210300	0.09241400	-0.30040600
P	0.74761800	2.88331300	-0.88923300
C	-1.84049000	-0.22415300	-0.15244600
C	-2.44850500	-1.23201200	-0.86033400
C	-2.68117600	0.56269500	0.73743500
C	-3.84261300	-1.51481600	-0.79080700
H	-1.85996200	-1.84419200	-1.54808000
C	-4.08496700	0.34660300	0.80731600
C	-2.11026500	1.55117600	1.56761900
C	-4.40268700	-2.56386200	-1.55160800
C	-4.68638800	-0.71579800	0.02374300
C	-4.84937600	1.17057900	1.65949600
H	-1.02507600	1.65488500	1.55219800
C	-2.87945000	2.33865700	2.39573200
C	-5.75269200	-2.82477500	-1.51632400
H	-3.73878700	-3.16410400	-2.17280200

C	-6.06510100	-1.00442400	0.03665700
C	-4.26808100	2.15336100	2.43035000
H	-5.92543800	1.03007000	1.72130300
H	-2.41028600	3.08747700	3.03053600
C	-6.59082400	-2.03516800	-0.71357100
H	-6.17037600	-3.63676200	-2.10721500
H	-6.73899500	-0.41121900	0.64944100
H	-4.88702400	2.76992300	3.07837000
H	-7.65927300	-2.23562400	-0.68168100
C	2.28144500	0.33211900	-0.41874700
C	3.56303700	0.81820400	-0.39313200
C	2.13152400	-1.07692600	-0.12453100
C	4.69766200	0.01072400	-0.05988200
H	3.76083100	1.87643200	-0.58599400
C	3.18510700	-1.90256500	0.29761600
C	0.81639400	-1.58968100	-0.18389900
C	5.99462700	0.56379400	-0.05639000
C	4.52891200	-1.35140200	0.31453400
C	2.86623600	-3.21409000	0.71576300
H	0.18653900	-1.13391700	-1.02502300
C	0.52240400	-2.87666800	0.22360200
C	7.09249300	-0.18968400	0.29785400
H	6.11302000	1.60890800	-0.34116400
C	5.66405800	-2.09413500	0.67996100
C	1.56522900	-3.68097000	0.70557400
H	3.65354500	-3.87604000	1.06889700
H	-0.49246100	-3.26355000	0.15241200
C	6.92519000	-1.53056000	0.66940800
H	8.08557700	0.25399100	0.29113100
H	5.55766900	-3.13785400	0.96735200
H	1.35394400	-4.69215300	1.04508900
H	7.78838000	-2.13047300	0.94803600
C	1.33321000	4.02794600	0.40856100
H	2.37574100	3.80319500	0.65769200
H	1.25739600	5.07050200	0.07708900
H	0.72941900	3.89270500	1.31255500
C	1.67542800	3.40671300	-2.36949100
H	2.74220200	3.19723000	-2.23881500
H	1.31849100	2.83813600	-3.23451800
H	1.53977500	4.47839600	-2.55710600
C	-0.95453000	3.41936100	-1.22201600
H	-1.37368400	2.81893500	-2.03587600
H	-1.57711900	3.27829100	-0.33179600
H	-0.96720200	4.47815400	-1.50497000

15) 13-Phen

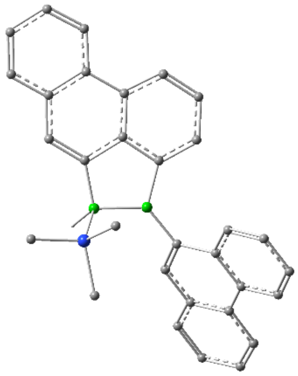

Note: Selected hydrogen atoms are omitted for clarity.

Number of imaginary frequencies = 0

E_{total} = -1587.778692 a.u

$G_{correction}$ = 0.428430 a.u

Cartesian coordinates:

B	0.76342400	1.45445000	-0.45608400
B	-0.20753700	0.03350300	-0.39021600
P	0.48692500	2.38546700	1.20030100
C	-1.76540900	-0.08263900	-0.33800600
C	-2.45714500	0.65405000	-1.26309200
C	-2.53005000	-0.90433700	0.58029400
C	-3.87280800	0.57422600	-1.42808800
H	-1.90974700	1.32348600	-1.92951600
C	-3.94809600	-0.97723300	0.48950900
C	-1.88322600	-1.60757500	1.62006900
C	-4.51971200	1.31199000	-2.44076600
C	-4.63599200	-0.24637200	-0.55932100
C	-4.64257000	-1.75605100	1.43742000
H	-0.79759900	-1.55086700	1.68664600
C	-2.58699900	-2.36180400	2.53280600
C	-5.88391500	1.24315500	-2.60590900
H	-3.91171500	1.93593000	-3.09457700
C	-6.03052000	-0.30033500	-0.75461700
C	-3.98274600	-2.43596800	2.43797500
H	-5.72654400	-1.82075000	1.39201600
H	-2.06227100	-2.89426200	3.32266500
C	-6.64270900	0.42591900	-1.75430000
H	-6.37218200	1.81383000	-3.39231800
H	-6.64674400	-0.92760400	-0.11559700
H	-4.54867900	-3.02584900	3.15534800
H	-7.72077100	0.36190400	-1.88299000
C	2.23830500	0.80745700	-0.48771400
C	3.48409700	1.35887300	-0.53826800
C	2.14631100	-0.63163800	-0.46959700
C	4.67684500	0.55938500	-0.57023700
H	3.61181100	2.44413300	-0.57458300
C	3.27872500	-1.47381400	-0.52066000
C	0.82005200	-1.14655200	-0.42445800
C	5.94748700	1.16821000	-0.61290800
C	4.59235900	-0.86021100	-0.55836000
C	3.04455000	-2.86138200	-0.54410700
H	0.49789100	2.29855600	-1.29819600

C	0.64672900	-2.52597500	-0.46202700
C	7.10118200	0.41610800	-0.64029400
H	5.99873000	2.25664800	-0.62519500
C	5.78704500	-1.60420800	-0.58712400
C	1.75811000	-3.37523500	-0.52218900
H	3.88231500	-3.55458500	-0.59166100
H	-0.35540300	-2.95408800	-0.44510800
C	7.01838200	-0.98393700	-0.62613800
H	8.07260500	0.90433300	-0.67341000
H	5.74460400	-2.69123500	-0.57618500
H	1.61542100	-4.45352400	-0.55347000
H	7.92609800	-1.58276600	-0.64686900
C	0.64501700	1.33295000	2.67551100
H	1.61457100	0.82220600	2.65811000
H	0.56848300	1.92543200	3.59424800
H	-0.14973500	0.57714000	2.67546200
C	1.62551000	3.77133300	1.51677500
H	2.64711200	3.38738100	1.61119000
H	1.59118100	4.46823000	0.67204100
H	1.35685500	4.30306200	2.43705900
C	-1.17229000	3.11565700	1.32059500
H	-1.33574600	3.78531900	0.46895300
H	-1.92677500	2.32230000	1.28293200
H	-1.28618200	3.67957800	2.25333200

16) TS-Phen[13-8]

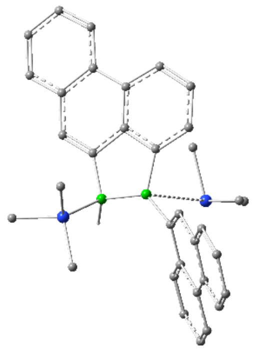

Note: Selected hydrogen atoms are omitted for clarity.

Number of imaginary frequencies = 1 (32.9 cm^{-1})

E_{total} = -2048.758041 a.u

$G_{correction}$ = 0.530739 a.u

Cartesian coordinates:

B	-0.23781500	0.13224600	0.02330800
P	0.15481200	2.30708900	-1.89535100
C	1.14639300	3.04345500	-3.23383000
H	2.18152200	3.16267700	-2.89582800
H	0.75157500	4.02317400	-3.52722300
H	1.13698700	2.37379800	-4.10084600
B	0.68936500	0.53367100	-1.37720500
H	0.55257700	-0.10893700	-2.40469100
C	0.24658800	3.53152700	-0.55287000
H	1.26798500	3.56057700	-0.15693200
H	-0.43365600	3.24558800	0.25733600
H	-0.02780900	4.52978700	-0.91229600
P	0.46102300	-3.81992800	-0.67397200
C	-1.55865800	2.38869100	-2.49500500
H	-1.79845400	3.39540400	-2.85568400
H	-2.24590900	2.12452600	-1.68317600
H	-1.69412200	1.66887000	-3.30967700
C	0.54579700	-5.67029400	-0.57319000
H	1.32300100	-6.04575100	-1.24868400
H	-0.40952500	-6.10489300	-0.88923500
H	0.76895200	-6.01672800	0.44526900
C	-0.72026800	-3.53506500	0.72442900
H	-0.39261900	-4.03779900	1.64509000
H	-1.71456400	-3.90785400	0.45066600
H	-0.81439700	-2.46177700	0.92959800
C	2.04964600	-3.42703300	0.19163300
H	2.09826400	-3.87792300	1.19254800
H	2.16556500	-2.34137600	0.28841000
H	2.89210800	-3.79716100	-0.40419700
C	3.39597800	0.83687900	-1.28955400
H	3.49634600	1.05014100	-2.35706500
C	4.60369100	0.80664300	-0.51255900
C	5.84901100	1.08267900	-1.11272100
H	5.86914700	1.31569400	-2.17698900
C	7.01664100	1.05971800	-0.38235300
H	7.96787400	1.27562000	-0.86371000
C	6.97457800	0.75603700	0.98628600
H	7.89335100	0.73807900	1.56806300
C	5.76936400	0.47875800	1.59695900
H	5.75820100	0.24676000	2.65975500
C	4.56122300	0.49465000	0.87453800
C	3.27516200	0.19946500	1.47582100
C	3.08840600	-0.19526000	2.81447800
H	3.94455300	-0.28693900	3.48018400
C	1.82931900	-0.49441400	3.30949400
H	1.72716100	-0.81037400	4.34576100
C	0.69589900	-0.40262100	2.49168200
H	-0.28450600	-0.65378800	2.89779200
C	0.82266300	-0.00879700	1.16441200
C	2.12296700	0.27677100	0.66354900
C	2.17172300	0.58765200	-0.74355800
C	-2.25162500	0.88574300	1.31871400
H	-1.54873100	1.35867200	2.00791100
C	-3.64164200	1.14706900	1.52588300
C	-4.06539400	1.98541700	2.57704400
H	-3.30908100	2.42452900	3.22664200
C	-5.40121400	2.24592000	2.78184700
H	-5.71658900	2.89398500	3.59612700
C	-6.35543400	1.67022900	1.92980900
H	-7.41259600	1.87499500	2.08269600
C	-5.96234800	0.84477200	0.89719700
H	-6.72592700	0.41660200	0.25292400
C	-4.60273600	0.55619500	0.66663000
C	-4.14259200	-0.31664400	-0.39929200
C	-5.03699300	-0.98454800	-1.26025000
H	-6.10872900	-0.85723500	-1.13100800
C	-4.58576200	-1.81476400	-2.26378700
H	-5.30121900	-2.32111300	-2.90800400
C	-3.21055600	-2.01051300	-2.44861900
H	-2.84884200	-2.67039100	-3.23404400
C	-2.31409800	-1.36795200	-1.62339600
H	-1.24496500	-1.52809500	-1.75569200
C	-2.74778300	-0.51538700	-0.58672000
C	-1.77764500	0.11696200	0.28688900

17) 8-Phen

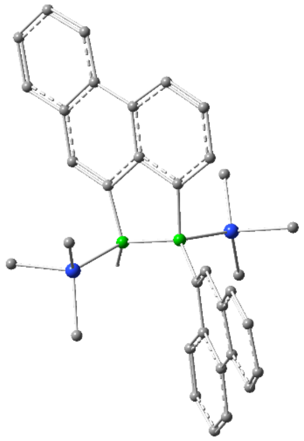

Note: Selected hydrogen atoms are omitted for clarity.

Number of imaginary frequencies = 0

E_{total} = -2048.784190 a.u

$G_{correction}$ = 0.538373 a.u

Cartesian coordinates:

B	-0.17045000	-0.07176600	0.62040800
P	0.31816200	-1.78611000	-1.85413200
C	1.45895000	-2.95468600	-2.66818100
H	2.47420500	-2.54463200	-2.65566100
H	1.15952800	-3.13857800	-3.70660600
H	1.45925300	-3.90326900	-2.11918800
B	0.78444300	-1.44863700	0.00511400
H	0.73887700	-2.56663100	0.51061300
C	0.37697400	-0.31442800	-2.92316900
H	1.37326700	0.13805100	-2.87575500
H	-0.35980000	0.42006000	-2.57953000
H	0.15641000	-0.58715100	-3.96169300
P	-0.07781600	-0.17073000	2.61467200
C	-1.31640300	-2.51619200	-2.19652600
H	-1.40084100	-2.75700600	-3.26284600
H	-2.11178600	-1.81502600	-1.92387300
H	-1.44448000	-3.43337600	-1.61147500
C	-0.63850800	-1.65349300	3.52753400
H	-0.13635800	-2.54483500	3.13554100
H	-1.72046500	-1.78487600	3.43628500
H	-0.38243800	-1.54141600	4.58785500
C	-0.99371000	1.17532500	3.44018300
H	-0.85957200	1.13416800	4.52736200
H	-2.06051200	1.08870000	3.20507400
H	-0.63431600	2.14253300	3.07268600
C	1.62571400	0.01066300	3.24124800
H	1.62401500	-0.05490400	4.33543600
H	2.04588000	0.97560600	2.94085100
H	2.25783200	-0.78703900	2.83539900
C	3.47992000	-1.37254300	-0.28761800
H	3.57558800	-2.45707700	-0.38981500
C	4.68045400	-0.59388500	-0.37869700
C	5.92737700	-1.21477800	-0.60252000
H	5.95475600	-2.30100500	-0.68764900
C	7.08509800	-0.47796000	-0.71584700
H	8.03653700	-0.97593700	-0.89010300
C	7.03250100	0.92000500	-0.60757400
H	7.94306400	1.50793400	-0.69953000
C	5.82669000	1.55072400	-0.38393200
H	5.80767800	2.63546800	-0.30437300
C	4.62661400	0.82312100	-0.26193800
C	3.34481200	1.45248200	-0.00626700
C	3.17123500	2.83655800	0.19917000
H	4.02066200	3.51411300	0.14228100
C	1.92304900	3.34783800	0.49254400
H	1.81064600	4.41844900	0.65763600
C	0.80068000	2.50810400	0.60267800
H	-0.15687300	2.95417800	0.87548900
C	0.90639000	1.13789200	0.39663600
C	2.19719500	0.63109100	0.07717700
C	2.25629400	-0.79999000	-0.08620000
C	-2.06112300	1.34937300	-0.46948000
H	-1.29640200	2.08850000	-0.70786000
C	-3.39632500	1.67950200	-0.85993500
C	-3.67147300	2.89986100	-1.51148800
H	-2.83908900	3.56724000	-1.73260600
C	-4.95781300	3.24759100	-1.85608900
H	-5.15568700	4.19264200	-2.35700900
C	-6.01587000	2.37704200	-1.55305900
H	-7.03551300	2.64780000	-1.81771700
C	-5.76845100	1.17673500	-0.92064600
H	-6.60829500	0.52295800	-0.69868900
C	-4.46014500	0.79363100	-0.56339800
C	-4.13511100	-0.46987400	0.07021100
C	-5.12264300	-1.44346000	0.32754000
H	-6.16059900	-1.23325500	0.08221700
C	-4.80953600	-2.67097500	0.86908300
H	-5.59560600	-3.40066000	1.05031100
C	-3.47479900	-2.98168300	1.15756600
H	-3.21111900	-3.96169800	1.55026900
C	-2.49304200	-2.04253800	0.92690400
H	-1.45049000	-2.29650900	1.10041100
C	-2.78104400	-0.76050500	0.40903100
C	-1.70613700	0.19796700	0.18182100

18) TS-Phen[13-9]

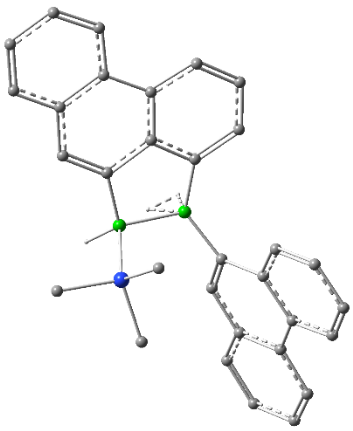

Note: Selected hydrogen atoms are omitted for clarity.

Number of imaginary frequencies = 1 (556.5 cm^{-1})

E_{total} = -1588.933592 a.u

$G_{correction}$ = 0.448161 a.u

Cartesian coordinates:

B	0.67857100	1.40861400	-0.67652600
B	-0.20635700	-0.15695700	-1.14354100
P	0.12091600	2.01672600	1.07401900
C	-1.76948300	-0.27289400	-0.80633500
C	-2.65129600	0.44068000	-1.57264800
C	-2.32506700	-1.02417200	0.30317600
C	-4.05632000	0.49332900	-1.34100800
H	-2.27824100	1.02544900	-2.41850500
C	-3.72600300	-1.01200800	0.57600800
C	-1.49005100	-1.78044600	1.15792000
C	-4.89756600	1.26296200	-2.17290100
C	-4.61707300	-0.23571100	-0.26304100
C	-4.20782100	-1.75634900	1.67278700
H	-0.42296700	-1.80245500	0.95364400
C	-1.98909700	-2.49477500	2.22496800
C	-6.25449600	1.31594400	-1.95462100
H	-4.44663000	1.81512600	-2.99676000
C	-6.01135900	-0.16485800	-0.06491000
C	-3.36566600	-2.48392000	2.48368800
H	-5.27236000	-1.75955300	1.89175000
H	-1.31578000	-3.06823200	2.85811400
C	-6.81484000	0.59268400	-0.88951100
H	-6.89197600	1.91235200	-2.60338300
H	-6.47434500	-0.71569700	0.74989700
H	-3.77122000	-3.04732200	3.32097300
H	-7.88751700	0.62822600	-0.71368200
C	2.18547600	0.87186600	-0.58444400
C	3.35721500	1.54932300	-0.41457400
C	2.23987900	-0.56368000	-0.69132800
C	4.61755400	0.87243100	-0.31547500
H	3.36773600	2.64080100	-0.35717800
C	3.44901700	-1.28913600	-0.58833900
C	0.99564700	-1.19558800	-0.94485200
C	5.81086600	1.60213600	-0.13549000
C	4.67878000	-0.54740300	-0.38396500
C	3.37399300	-2.69156000	-0.69965200
H	-0.00082500	0.68379600	-2.08771500
C	0.97769900	-2.57632400	-1.05961000
C	7.02772700	0.96804400	-0.02263200
H	5.74931100	2.68911000	-0.08745700
C	5.93734200	-1.16866200	-0.26464700
C	2.16254600	-3.31731000	-0.92389200
H	4.27326200	-3.29821400	-0.61672500
H	0.03977700	-3.09721500	-1.25563300
C	7.08956400	-0.43213900	-0.08683100
H	7.93746400	1.54819200	0.11544800
H	6.00803700	-2.25302800	-0.31324100
H	2.13120900	-4.40189000	-1.01153100
H	8.04779000	-0.93897500	0.00254300
C	0.44417600	0.90081000	2.46966700
H	-0.20870500	0.02338100	2.41695800
H	1.48842200	0.57072100	2.43713900
H	0.26220200	1.42179900	3.41662100
C	1.03918200	3.52742900	1.50765400
H	2.10891400	3.29990700	1.56860300
H	0.88437000	4.28534800	0.73209700
H	0.70342900	3.92216100	2.47349800
C	-1.62502500	2.49817400	1.21655000
H	-2.26388400	1.60971300	1.19033900
H	-1.79340500	3.03523100	2.15696400
H	-1.89465400	3.14656700	0.37556400
H	-0.27653400	-0.14450400	-2.46541500
H	0.50931600	2.42676800	-1.33314900

19)9-Phen

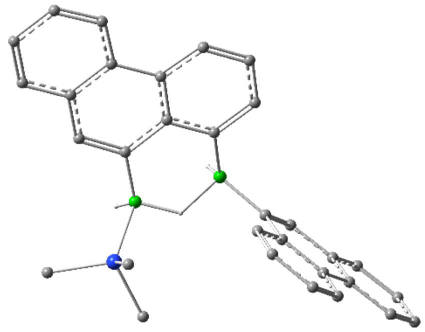

Note: Selected hydrogen atoms are omitted for clarity.

Number of imaginary frequencies = 0

E_{total} = -1588.978263 a.u

$G_{correction}$ = 0.447819 a.u

Cartesian coordinates:

B	0.99063400	1.33414700	-1.33675400
B	-0.49964900	-0.56348200	-1.34460900
P	0.81978000	2.93457400	-0.22636800
C	-1.97269300	-0.48460800	-0.75075700
C	-3.01774600	-0.65273500	-1.61389100
C	-2.28544700	-0.26164600	0.64784600
C	-4.38806900	-0.63899400	-1.20861700
H	-2.81221900	-0.80920700	-2.67390500
C	-3.63433300	-0.21517100	1.10105500
C	-1.25465100	-0.06914700	1.59437600
C	-5.41754400	-0.83640200	-2.15099600
C	-4.71426900	-0.41497500	0.15206000
C	-3.88055500	0.02360800	2.46857900
H	-0.22071700	-0.12964400	1.25887300
C	-1.52444300	0.16100000	2.92657200
C	-6.74010200	-0.81573300	-1.77069100
H	-5.14135700	-1.00739500	-3.19070100
C	-6.07650700	-0.39750100	0.51271000
C	-2.85317200	0.20945300	3.36752800
H	-4.90376900	0.06232200	2.83326600
H	-0.70701000	0.29234000	3.63319100
C	-7.06834600	-0.59346100	-0.42494200
H	-7.52632100	-0.97062300	-2.50585000
H	-6.36490600	-0.22931500	1.54701900
H	-3.07605900	0.38787800	4.41698100
H	-8.11128200	-0.57646300	-0.11678500
C	2.21687400	0.41310700	-0.92272600
C	3.50466600	0.86550200	-0.87665700
C	1.95721900	-0.95692100	-0.54434300
C	4.59578800	0.05253200	-0.43911500
H	3.74145600	1.88628400	-1.18886700

C	3.00111300	-1.79800300	-0.07555200
C	0.62815500	-1.44306100	-0.66236300
C	5.90910300	0.56532500	-0.40539200
C	4.35492600	-1.28047500	-0.01522200
C	2.67352800	-3.11370600	0.30942500
H	-0.13468200	0.84157900	-1.05537100
C	0.35899900	-2.74193800	-0.26264800
C	6.96247100	-0.20266500	0.03549300
H	6.07373100	1.58970400	-0.73885400
C	5.45291300	-2.04196600	0.43400700
C	1.37727700	-3.57358100	0.22332100
H	3.44621400	-3.78246700	0.68061000
H	-0.65882400	-3.12673700	-0.33426700
C	6.72834300	-1.51927900	0.46101400
H	7.97028600	0.20600900	0.05485600
H	5.30112700	-3.06596500	0.76600100
H	1.14518500	-4.59109400	0.53210500
H	7.55578900	-2.13217400	0.81137200
C	1.08093200	2.72231300	1.55620900
H	2.03603700	2.21459300	1.72926700
H	1.09147000	3.69654800	2.05756500
H	0.27340600	2.11350100	1.97757800
C	1.97599500	4.24606600	-0.72077500
H	3.00156100	3.95381200	-0.47249100
H	1.90683500	4.40717700	-1.80177700
H	1.73894900	5.17981400	-0.19824400
C	-0.83510500	3.65135300	-0.41481300
H	-1.58514100	2.93476100	-0.06132800
H	-0.92921700	4.58301400	0.15366200
H	-1.02115000	3.85162800	-1.47560000
H	-0.48053800	-0.56758200	-2.55242100
H	0.94760200	1.76603800	-2.46771700

20) TS-Phen[9-14]

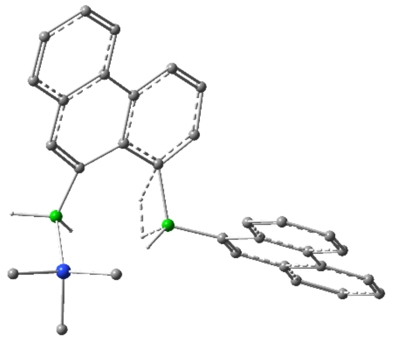

Note: Selected hydrogen atoms are omitted for clarity.

Number of imaginary frequencies = 1 (1405.9 cm^{-1})

E_{total} = -1590.097970 a.u

$G_{correction}$ = 0.464023 a.u

Cartesian coordinates:

B	-0.70848500	0.79664000	-0.90299600
H	-0.13799900	1.57959600	-0.20934600
C	2.60154600	0.66606900	-0.91865300
C	3.91156400	0.50962900	-0.54859700
C	1.77259400	-0.53080000	-0.95954900
C	4.38369600	-3.13314000	0.03025400
C	4.51786400	-0.72809800	-0.19393800
C	2.38247300	-1.81172000	-0.75899100
C	0.36448300	-0.51571000	-1.24321300
C	5.69455900	-3.17213400	0.45485400
H	3.82997600	-4.06666900	-0.01970500
C	5.86091500	-0.78822800	0.23208100
C	3.75910500	-1.91449700	-0.30918100
C	1.64575000	-2.98408800	-1.02119200
C	-0.29981400	-1.70225000	-1.52472800
C	6.44601800	-1.99058500	0.55388500
H	6.14660700	-4.12606200	0.71677300
H	6.42540500	0.14112600	0.30072300
C	0.33809400	-2.93932000	-1.43974300
H	2.12715400	-3.95239400	-0.91785500
H	-1.35810300	-1.66662400	-1.77783700
H	7.48124800	-2.02838700	0.88504200
H	-0.20206500	-3.85492300	-1.66704300
C	-2.17734300	0.34297500	-0.51507800
C	-3.23119900	0.51079800	-1.36402100
C	-2.44317100	-0.21523500	0.79520100
C	-6.19499000	-0.71955600	0.54846700
C	-4.57535100	0.15689500	-1.02700400
C	-3.76394900	-0.56687400	1.18763600
C	-1.38784700	-0.42114700	1.71039600
C	-7.20376500	-0.52962200	-0.37225200
H	-6.44794700	-1.14089200	1.51790000
C	-5.62140900	0.34429900	-1.95253000
C	-4.85888800	-0.38372300	0.25223100
C	-3.96475000	-1.08763300	2.48203900
H	-0.37260000	-0.17486600	1.40337000
C	-1.61240500	-0.93495100	2.96816700
C	-6.91811500	0.00803600	-1.63598400
H	-8.22564200	-0.79935600	-0.11549400
H	-5.38029100	0.76267700	-2.92913600
C	-2.91642900	-1.26766100	3.35794600
H	-4.96672700	-1.35614400	2.80699100
H	-0.78135200	-1.08559000	3.65337800
H	-7.71658800	0.15669700	-2.35923600
H	-3.10359600	-1.67238500	4.35002800
H	-0.80886100	1.43814200	-2.01606600
H	-0.19066500	0.58667400	-2.06723300
B	2.19079100	2.15636200	-1.37969800
H	1.20227200	2.29789400	-2.05865300
H	3.13722800	2.71431500	-1.91438900
P	1.96229400	3.32998400	0.18453400
C	0.87536200	4.74021600	-0.17778500
H	1.25569000	5.26523900	-1.06091000
H	-0.12999600	4.36864800	-0.40485600
H	0.82400400	5.43768200	0.66567300
C	1.35214500	2.63814800	1.75720100
H	1.52566900	3.35165900	2.57109000
H	0.28231200	2.41724300	1.69957700
H	1.89107400	1.70890200	1.97661400
C	3.54837400	4.08150100	0.67030000
H	4.22877900	3.29974200	1.02629200
H	4.00451000	4.57023200	-0.19690400
H	3.40697900	4.81688900	1.47064600
H	4.56446900	1.38611700	-0.55845400
H	-3.06757900	0.94049000	-2.35485300

21) 14-Phen

14-Phen comprises two components: phenanthren-9-ylborane (**14-Phen**) and its PMe_3-coordinated version (**10-Phen**). Structures and energies of these compounds are given seperately (see below).

22) 14-Phen

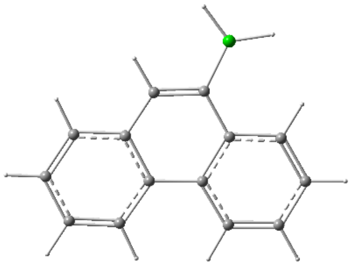

Number of imaginary frequencies = 0

E_{total} = -564.562886 a.u

$G_{correction}$ = 0.168253 a.u

Cartesian coordinates:

C	-3.66940400	-0.11249500	-0.00000200
C	-2.82323000	0.97122200	-0.00000500
C	-1.42402100	0.79518700	-0.00000300
C	-0.86847600	-0.51039700	0.00000100
C	-1.76084000	-1.60022600	0.00000600
C	-3.12648100	-1.40641800	0.00000400
C	-0.55671600	1.92258000	-0.00000300
C	0.57660300	-0.66192100	0.00000100
C	1.40453100	0.49385000	0.00000000
C	0.81406600	1.82339900	0.00000000
C	2.80306100	0.32037300	-0.00000200
H	3.43860400	1.20189600	-0.00000300
C	3.37454600	-0.93265900	-0.00000300
C	2.55809200	-2.07035000	-0.00000200
C	1.18776900	-1.93200400	-0.00000100
H	-1.01662700	2.91153100	-0.00000500
H	-4.74721100	0.02823400	-0.00000400
H	-3.21715400	1.98645800	-0.00000900
H	-1.38080200	-2.61793600	0.00001200
H	-3.78891200	-2.26902500	0.00000800
H	4.45696500	-1.03722400	-0.00000400
H	3.00236200	-3.06287700	-0.00000300
H	0.57313700	-2.82811600	-0.00000200
B	1.59601900	3.14199700	0.00000800
H	2.78861000	3.21147600	0.00002200
H	0.97392900	4.16474900	0.00000100

23) 10-Phen

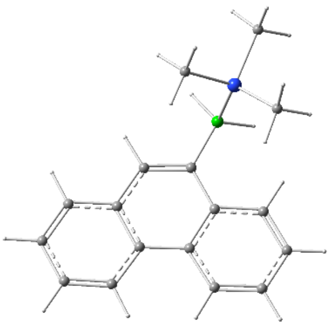

Number of imaginary frequencies = 0

E_{total} = -1025.581973 a.u

$G_{correction}$ = 0.273721 a.u

Cartesian coordinates:

B	-1.90364800	-0.46557500	-1.38891100
H	-2.07694900	-1.56049500	-1.90298800
H	-2.41398700	0.41415300	-2.05745900
P	-3.06008500	-0.63872500	0.16812800
C	-2.53044000	-1.97002500	1.28613500
H	-2.43663200	-2.90269300	0.71858400
H	-1.55042600	-1.72393300	1.70898700
H	-3.24962800	-2.11266400	2.10057100
C	-3.26660400	0.80958600	1.25061300
H	-3.93162200	0.57246200	2.08928500
H	-2.29374100	1.12486800	1.64264300
H	-3.69654900	1.63929300	0.67920600
C	-4.74993400	-1.08202400	-0.33171000
H	-5.15299500	-0.29391900	-0.97696300
H	-4.72260200	-2.01419500	-0.90602500
H	-5.40518600	-1.20981900	0.53708300
C	-0.38436400	-0.25662000	-0.91732900
C	0.44062100	-1.34205000	-0.82260100
C	0.16735600	1.02810800	-0.52874600
C	1.79315900	-1.27810600	-0.36659000
H	0.06270800	-2.32519900	-1.11206000
C	1.50529900	1.14783500	-0.05325100
C	-0.61793600	2.19913000	-0.61447700
C	2.58661100	-2.44186200	-0.29993000
C	2.34317300	-0.03350500	0.02976200
C	1.97545100	2.42154100	0.32660500
H	-1.62814200	2.11122300	-1.00756600
C	-0.13229500	3.43211500	-0.23891400
C	3.88808900	-2.39046000	0.14459200
H	2.14456200	-3.38762900	-0.61168600
C	3.67885400	-0.01000800	0.47969500
C	1.17909400	3.54256300	0.24154400
H	2.99133700	2.53237000	0.69734200
H	-0.76100500	4.31608600	-0.32150600
C	4.43700300	-1.16020000	0.53772400
H	4.48953300	-3.29560900	0.19000600
H	4.13358800	0.92851600	0.78669300
H	1.57278800	4.51108700	0.54187700
H	5.46564200	-1.11125800	0.88786100

24) TS-Phen[9-15]

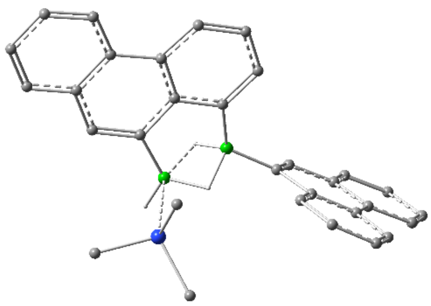

Note: Selected hydrogen atoms are omitted for clarity.

Number of imaginary frequencies = 1 (344.4 cm^{-1})

E_{total} = -1588.950255 a.u

$G_{correction}$ = 0.446707 a.u

Cartesian coordinates:

B	0.75866900	0.39939600	-1.68532300
B	-0.49377500	-0.67427400	-0.70644100
P	1.35385500	2.76657300	-0.62587300
C	-2.05777100	-0.55223500	-0.49473400
C	-2.91166100	-1.27549300	-1.27403300
C	-2.62670300	0.30044800	0.52782800
C	-4.33198500	-1.24403300	-1.11669900
H	-2.51110600	-1.91775200	-2.06016700
C	-4.03476800	0.38939300	0.70784300
C	-1.79142700	1.08065400	1.35756700
C	-5.16263000	-2.02892400	-1.94165000
C	-4.91117700	-0.41235400	-0.12576100
C	-4.53306500	1.26392500	1.69476000
H	-0.71109000	0.99225900	1.24479900
C	-2.30483800	1.92653500	2.31582900
C	-6.53112600	-2.00422400	-1.79861600
H	-4.69535400	-2.65944700	-2.69703600
C	-6.31492500	-0.40823600	-0.00053600
C	-3.69301700	2.02081600	2.48230800
H	-5.60623900	1.35286300	1.84234300
H	-1.63564800	2.51407400	2.94174100
C	-7.10908200	-1.18553500	-0.81667300
H	-7.16194000	-2.61516200	-2.44005800
H	-6.79184400	0.21291000	0.75350100
H	-4.10954700	2.68769600	3.23363700
H	-8.18976200	-1.16347100	-0.69607300
C	2.07705100	-0.22939900	-1.12390600
C	3.35335100	-0.03197800	-1.56359700
C	1.86319000	-1.06655500	0.02534800
C	4.47610500	-0.59126800	-0.87315300
H	3.54959200	0.58078500	-2.44603400
C	2.92805200	-1.63505500	0.75208000
C	0.50691000	-1.29703700	0.35787600
C	5.78753900	-0.34934100	-1.33249100
C	4.28080200	-1.38085500	0.29632100
C	2.59317000	-2.42141700	1.87487500
H	-0.15220400	0.64595200	-0.82009500
C	0.22506900	-2.07519100	1.46419700
C	6.88005900	-0.86112900	-0.67033200
H	5.91811600	0.25617100	-2.22889400
C	5.41842700	-1.88952600	0.95242400
C	1.27275000	-2.62869400	2.22224400
H	3.37531100	-2.87797400	2.47761100
H	-0.80871200	-2.27296600	1.74789000
C	6.69021500	-1.63756300	0.48318600
H	7.88513000	-0.66604700	-1.03676700
H	5.29633600	-2.49529800	1.84730800
H	1.04106000	-3.23933700	3.09271700
H	7.55029600	-2.04422700	1.01020600
C	1.98549700	2.56419500	1.09074300
H	2.79078600	1.82073100	1.10440600
H	2.37407200	3.51230400	1.48503700
H	1.18594300	2.21127000	1.75328800
C	2.80302200	3.56984200	-1.42556500
H	3.65295300	2.87753300	-1.42552300
H	2.56687300	3.82720400	-2.46369000
H	3.09695500	4.48167400	-0.88993200
C	0.20907100	4.19433800	-0.41864900
H	-0.66568900	3.88079300	0.16318200
H	0.69702200	5.03282800	0.09508600
H	-0.14253700	4.53414600	-1.39865100
H	-0.19192300	-1.05534900	-1.84905600
H	0.54421900	0.83701000	-2.76807800

25) 15-Phen

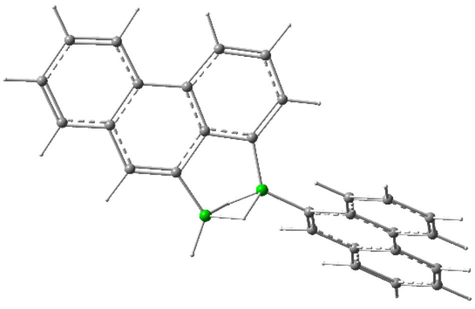

Number of imaginary frequencies = 0

E_{total} = -1127.982986 a.u

$G_{correction}$ = 0.344806 a.u

Cartesian coordinates:

B	-0.14792000	-0.47684900	-0.34740000
H	0.21408300	-0.39105800	-1.66048600
C	2.33080200	-1.04081800	-0.89608000
C	3.56528200	-1.45192500	-1.29851700
C	2.23905200	-0.05310000	0.14204800
C	5.87902800	0.54526500	0.87461300
C	4.75782600	-0.91937900	-0.70751900
C	3.37051400	0.50264500	0.76718500
C	0.91897900	0.31402300	0.49615000
C	7.10452300	0.09648000	0.42852000
H	5.84652700	1.29142500	1.66504700
C	6.02295300	-1.36092000	-1.14466200
C	4.67825400	0.05695500	0.32639800
C	3.13880900	1.45413100	1.78067100
C	0.73629300	1.24818100	1.50077900
C	7.18115600	-0.86486400	-0.59003900

H	8.01645000	0.49156900	0.87018900
H	6.06494800	-2.10805200	-1.93616500
C	1.85198400	1.81452400	2.13808700
H	3.97534800	1.91770100	2.29918500
H	-0.26744100	1.54942600	1.79895800
H	8.15004500	-1.21493900	-0.93770600
H	1.70473600	2.54952100	2.92636200
C	-1.71301500	-0.40186400	-0.24271600
C	-2.42472500	-1.49489300	0.15774300
C	-2.43309800	0.81780000	-0.53847000
C	-5.96797600	-0.32061000	0.23412400
C	-3.84347500	-1.48124900	0.32453700
C	-3.84698600	0.87842000	-0.39928000
C	-1.74574500	1.96369900	-0.99261700
C	-6.61441900	-1.46126200	0.66032800
H	-6.55845400	0.57052200	0.03881900
C	-4.52376700	-2.63664700	0.75841900
C	-4.57069500	-0.29546000	0.05280700
C	-4.50017600	2.08655900	-0.71534200
H	-0.66256100	1.92103600	-1.09758600
C	-2.40985300	3.13149400	-1.29391200
C	-5.88956000	-2.63286400	0.92543600
H	-7.69388100	-1.45043900	0.79216400
H	-3.94270400	-3.53541900	0.96084200
C	-3.80233500	3.19140500	-1.15221300
H	-5.58042200	2.15764100	-0.61969300
H	-1.85651200	4.00080500	-1.64089300
H	-6.40507800	-3.52938500	1.26126100
H	-4.33590200	4.10889800	-1.38921100
B	0.90711600	-1.47335800	-1.38530500
H	0.18758100	-1.78248600	-0.31984000
H	0.64108800	-2.27749900	-2.22133800
H	3.67836800	-2.20073300	-2.08377300
H	-1.90997500	-2.43185500	0.37852800

26) TS-Phen[12-17]

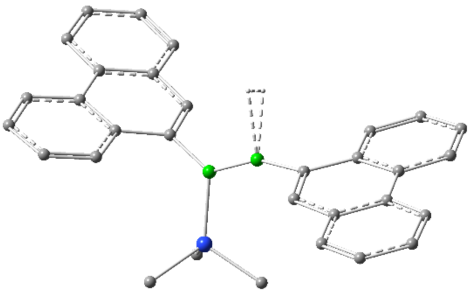

Note: Selected hydrogen atoms are omitted for clarity.

Number of imaginary frequencies = 1 (681.1 cm^{-1})

E_{total} = -1588.869768 a.u

$G_{correction}$ = 0.439833 a.u

Cartesian coordinates:

B	0.63431100	-0.34199000	0.36479000
P	0.41840200	-1.27962000	2.00198600
C	0.79732400	-3.06779000	2.04715300
H	1.83060700	-3.23815500	1.72598600
H	0.66710300	-3.46515400	3.06115800
H	0.12673000	-3.59954900	1.36280700
C	-1.25125700	-1.18512600	2.71620100
H	-1.54192100	-0.13719600	2.84272100
H	-1.97708200	-1.66916600	2.05528400
H	-1.26528000	-1.68655400	3.69066800
C	1.50678400	-0.60492000	3.29943300
H	1.30055500	0.46346100	3.42614700
H	1.35517700	-1.12310700	4.25350700
H	2.55160300	-0.71822000	2.98934600
B	-0.71322400	0.13140000	-0.21637700
H	-0.19300000	0.83652100	-1.78625600
H	-0.09772900	1.42969900	-1.29547700
C	2.12747800	-0.00555200	-0.03265500
C	2.62716800	1.25091400	0.17383700
C	3.03081800	-0.99768600	-0.58604600
C	3.97980500	1.61910300	-0.10620700
H	1.97542100	2.02034300	0.59358700
C	4.39570900	-0.69346700	-0.84980100
C	2.55798500	-2.29231400	-0.89104900
C	4.43051600	2.93442500	0.12969300
C	4.88702300	0.64889600	-0.60228100
C	5.22430100	-1.71329300	-1.36085300
H	1.49639000	-2.48927400	-0.74252900
C	3.38807900	-3.26947000	-1.39440500
C	5.73683700	3.29290600	-0.11313900
H	3.71745800	3.66452600	0.51114200
C	6.21923300	1.04306800	-0.83884600
C	4.73966700	-2.97690300	-1.62136100
H	6.27222700	-1.50626100	-1.56293800
H	2.99553400	-4.25726800	-1.62583700
C	6.63846400	2.33497300	-0.60133500
H	6.07039200	4.31145500	0.07227300
H	6.93997200	0.32380300	-1.21965700
H	5.40573300	-3.74051600	-2.01703000
H	7.67254100	2.61102200	-0.79499400
C	-2.24784500	0.19628500	-0.37640900
C	-2.92122300	-0.97272600	-0.61744400
C	-3.02874100	1.40948700	-0.21392200
C	-4.34377700	-1.05194300	-0.70969700
H	-2.35362000	-1.89338800	-0.76450300
C	-4.44409700	1.38903300	-0.34631200
C	-2.39341400	2.63102700	0.08835200
C	-4.97831900	-2.28852100	-0.94617500
C	-5.12142300	0.12866900	-0.59603900
C	-5.14797600	2.60210100	-0.20818200
H	-1.31372000	2.62245400	0.23855900
C	-3.10618000	3.80195600	0.22229000
C	-6.34661200	-2.37132000	-1.06606700
H	-4.35863400	-3.17996100	-1.03541700
C	-6.51950600	0.01354200	-0.72584100
C	-4.49766800	3.78681200	0.06234200
H	-6.22967700	2.61426800	-0.31279200
H	-2.59245000	4.73141800	0.45693100
C	-7.12062800	-1.20663800	-0.95425400
H	-6.82623600	-3.33009300	-1.24828300
H	-7.14888100	0.89599000	-0.64546100
H	-5.06930100	4.70666000	0.16272500
H	-8.20253800	-1.26366300	-1.04942400

27) 17-Phen

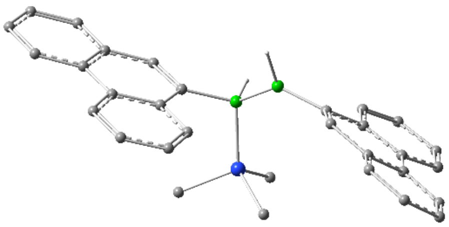

Note: Selected hydrogen atoms are omitted for clarity.

Number of imaginary frequencies = 0

E_{total} = -1588.954464 a.u

$G_{correction}$ = 0.446595 a.u

Cartesian coordinates:

B	-0.68102000	-1.11034800	-0.41937900
P	-0.13263600	-1.47230300	1.44340500
C	1.07005300	-0.35829900	2.24283400
H	0.67971600	0.66483700	2.26746400
H	1.26205900	-0.68804800	3.27053800
H	2.01325400	-0.36114000	1.68535800
C	0.58254400	-3.12847400	1.68546500
H	-0.11041600	-3.87995400	1.29198400
H	1.53213200	-3.21237500	1.14823700
H	0.75633700	-3.31903000	2.75077600
C	-1.54843300	-1.47155000	2.59016600
H	-2.29991900	-2.18788100	2.24109400
H	-1.22283600	-1.75008300	3.59906400
H	-2.01069000	-0.47944400	2.62158900
B	0.43062800	-0.22335500	-1.36028500
H	-0.04117600	0.41535100	-2.27288800
H	-0.71393500	-2.25050900	-0.87196200
C	-2.17163400	-0.49841700	-0.38729500
C	-3.24836100	-1.27597400	-0.70597700
C	-2.44068700	0.88027400	-0.02298200
C	-4.59923500	-0.80805700	-0.69989900
H	-3.08881800	-2.31959600	-0.98512200
C	-3.76768600	1.39647900	0.01588700
C	-1.38083600	1.75039800	0.31507000
C	-5.66100400	-1.66958800	-1.04415000
C	-4.87935900	0.53171500	-0.33174000
C	-3.95842200	2.74132900	0.39296400
H	-0.36165200	1.36675700	0.27639100
C	-1.59261300	3.06183200	0.68005200
C	-6.96581900	-1.23194700	-1.02819000
H	-5.42425100	-2.69505100	-1.32609100
C	-6.22421000	0.95303300	-0.32334200
C	-2.90039700	3.56140600	0.72119000
H	-4.96418100	3.15247100	0.42719500
H	-0.75039400	3.70393300	0.92896100
C	-7.24732500	0.09341000	-0.66323900
H	-7.77511400	-1.90714300	-1.29695900
H	-6.47299500	1.97435800	-0.04586200
H	-3.08358000	4.59464600	1.00719400
H	-8.27603800	0.44633400	-0.64885200
C	1.96481400	-0.00332600	-1.16417700
C	2.36307400	1.30467000	-1.08597200
C	2.94996000	-1.03009600	-0.90201400
C	3.66142200	1.69759700	-0.63742000
H	1.65785000	2.09958800	-1.34017600
C	4.27295900	-0.69139500	-0.50575400
C	2.62235800	-2.38897600	-1.09181700
C	3.99258200	3.06103900	-0.50090600
C	4.62717500	0.70581000	-0.32748900
C	5.20125500	-1.73383800	-0.31014900
H	1.61149300	-2.63768800	-1.41425700
C	3.55048100	-3.38861900	-0.89339000
C	5.23803200	3.44693200	-0.06048700
H	3.23677200	3.80485300	-0.74979900
C	5.89175700	1.13022100	0.12539000
C	4.85237200	-3.05431500	-0.49679800
H	6.22245100	-1.50029800	-0.02002000
H	3.27673800	-4.42911900	-1.05320600
C	6.19269100	2.46953200	0.25826000
H	5.48173100	4.50164500	0.04287900
H	6.65133100	0.39652800	0.38285400
H	5.59371900	-3.83558100	-0.34576300
H	7.17686300	2.76784600	0.61208900

28) TS-Phen[17-9]

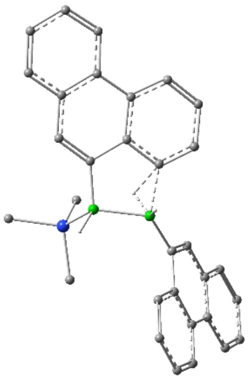

Note: Selected hydrogen atoms are omitted for clarity.

Number of imaginary frequencies = 1 (1143.1 cm^{-1})

E_{total} = -1588.907706 a.u

$G_{correction}$ = 0.445823 a.u

Cartesian coordinates:

B	0.78866100	-1.39727500	-0.13736200
P	0.28908200	-1.78236300	1.69192000
C	1.30347600	-3.08553000	2.45339600
H	2.34213400	-2.74372100	2.52146300
H	0.94216100	-3.33480700	3.45764500
H	1.27148200	-3.98174100	1.82400600
C	-1.42444000	-2.36986900	1.80263200
H	-1.66453100	-2.69730500	2.82029700
H	-2.10398000	-1.56030600	1.51200100
H	-1.56626100	-3.20375500	1.10630700
C	0.39970600	-0.38248300	2.84864400
H	0.13421000	-0.69642000	3.86446100
H	1.42225400	0.01189100	2.85256800
H	-0.28523300	0.41476600	2.53728000

B	-0.21095900	-0.05565500	-0.97008200
H	0.59156700	-2.45966600	-0.69285200
H	0.19764200	-0.31453900	-2.06340700
C	2.26939800	-0.77345200	-0.22702400
C	3.48792400	-1.37549800	-0.11629900
C	2.26203400	0.65489300	-0.46845400
C	5.94345800	1.43817000	-0.49806200
C	4.71895500	-0.64586900	-0.22136500
C	3.43358300	1.43567500	-0.54282600
C	0.98578200	1.24799000	-0.62026600
C	7.13960200	0.76196100	-0.38432700
H	5.95839300	2.51444000	-0.65445200
C	5.95622800	-1.31263700	-0.10828100
C	4.71067300	0.76230800	-0.42185200
C	3.27102800	2.82685200	-0.70790500
H	0.39591800	0.28596700	0.08816600
C	0.85063900	2.61664200	-0.73982500
C	7.14763600	-0.62711000	-0.18788500
H	8.07822700	1.30737400	-0.44941000
H	5.95010500	-2.39152400	0.04336300
C	2.01497600	3.39956100	-0.79460900
H	4.14354200	3.47457900	-0.75680800
H	-0.12964200	3.07873700	-0.83930200
H	8.09220900	-1.15911700	-0.10015800
H	1.92900800	4.47714200	-0.91826300
C	-1.74140200	0.23236200	-0.68749600
C	-2.15507600	1.22097700	0.16303900
C	-2.76605000	-0.61922600	-1.26395100
C	-5.87484600	0.89708100	0.30256600
C	-3.52344500	1.45486600	0.50323200
C	-4.14365000	-0.42723000	-0.96127700
C	-2.40657000	-1.67962400	-2.12464000
C	-6.20061100	1.91641300	1.17231400
H	-6.67589000	0.28873500	-0.10981400
C	-3.87891300	2.49207100	1.39025300
C	-4.53694200	0.63665700	-0.05563300
C	-5.08900700	-1.29285000	-1.54811500
H	-1.35343200	-1.83302500	-2.34814700
C	-3.35174500	-2.51282200	-2.68063800
C	-5.19405000	2.72364900	1.72355600
H	-7.24225800	2.09436800	1.42966800
H	-3.08542200	3.11161700	1.80755200
C	-4.70789100	-2.31392000	-2.39097300
H	-6.14671000	-1.15981600	-1.33525100
H	-3.04618700	-3.32013300	-3.34237700
H	-5.45349000	3.52795200	2.40829600
H	-5.46186100	-2.96492500	-2.82806500
H	3.55821600	-2.45261300	0.05308300
H	-1.41968700	1.88589100	0.62298900

Mechanistic pathways for the hydrogenation of 7-Pyr

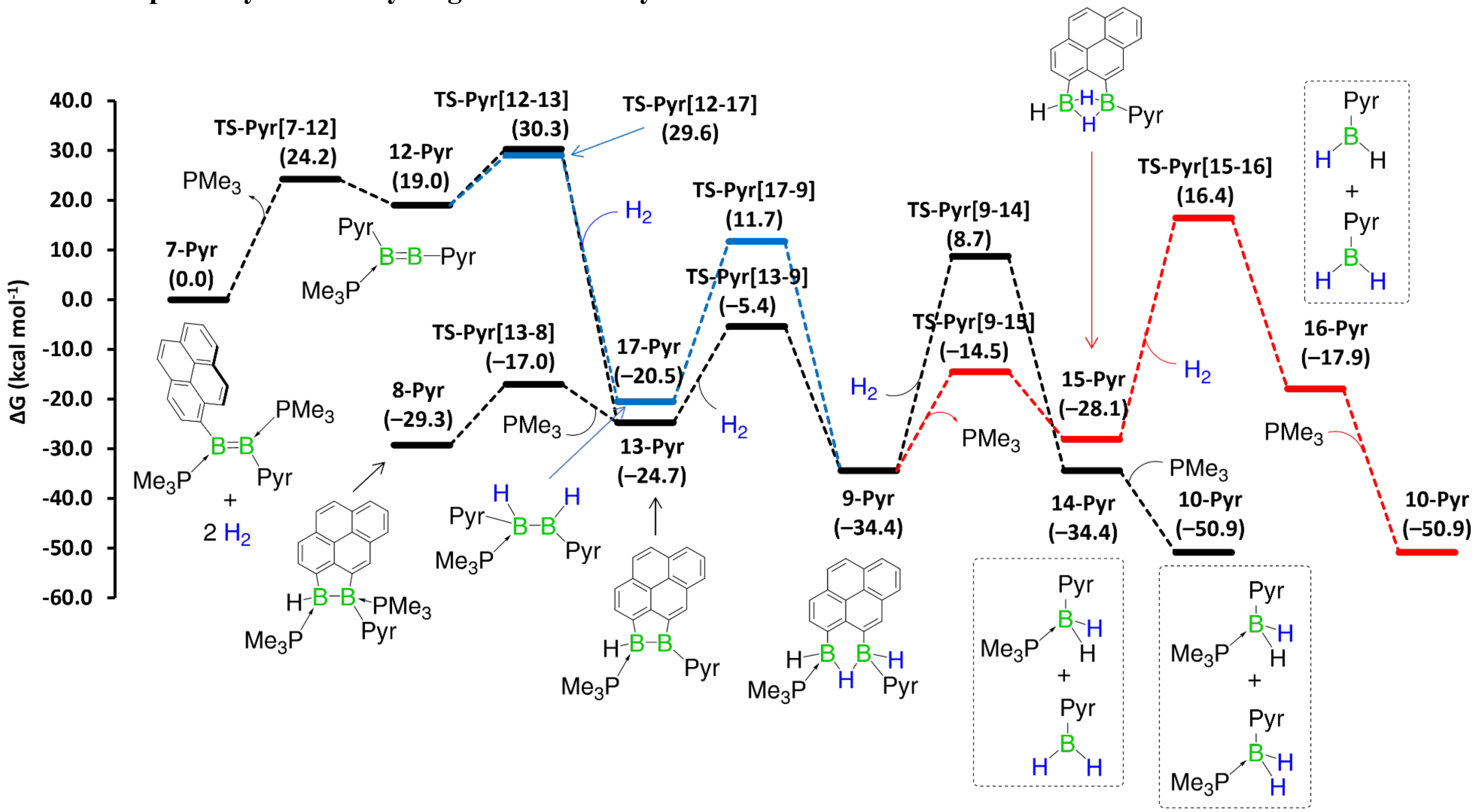

Figure S80. Computed mechanism for the formation of the borane **10-Pyr** from the hydrogenation of diborene **7-Pyr**.

Structures, energies and cartesian coordinates involving 7-Pyr in the hydrogenation:

Note: Structure and energy details of PMe_3 and H_2 are provided above in the structures, energies and cartesian coordinates involving diborene **7a-Phen** in the hydrogenation.

29) 7-Pyr

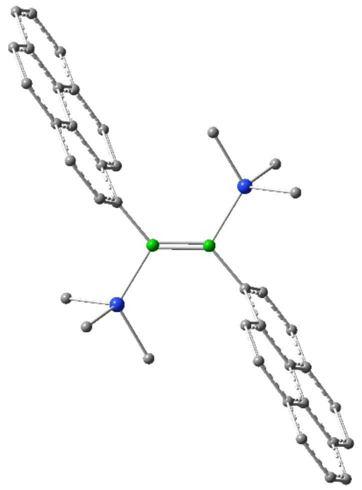

Note: Hydrogen atoms are omitted for clarity.

Number of imaginary frequencies = 0

E_{total} = -2201.094321 a.u

$G_{correction}$ = 0.560255 a.u

Cartesian coordinates:

B	0.45796400	-0.58748200	-0.25430100
B	-0.45803900	0.58761700	0.25477600
P	-0.21189100	-1.75025900	-1.61589700
P	0.21187700	1.75048500	1.61625100
C	-1.84106800	-1.39124600	-2.34580200
H	-2.63764900	-1.58727200	-1.62106200
H	-1.90095500	-0.34375800	-2.65811800
H	-1.98954600	-2.03796100	-3.21870700
C	0.89659600	-1.81484800	-3.06856800
H	0.94197000	-0.82611700	-3.53790300
H	1.90654400	-2.09084600	-2.74551700
H	0.54306800	-2.54744200	-3.80408900
C	-0.35045100	-3.51880100	-1.16296500
H	0.62887600	-3.89838700	-0.85186400
H	-1.05193500	-3.62777900	-0.32795100
H	-0.70812200	-4.11206400	-2.01341200
C	0.35109900	3.51893500	1.16314400
H	1.05260200	3.62759400	0.32810800
H	-0.62809600	3.89886200	0.85204400
H	0.70900700	4.11212000	2.01354600
C	1.84079800	1.39099500	2.34649800
H	1.90024800	0.34350200	2.65886700
H	2.63759000	1.58670300	1.62190000
H	1.98933400	2.03769300	3.21940600
C	-0.89687100	1.81562000	3.06869600
H	-1.90672300	2.09165300	2.74537900
H	-0.94245800	0.82701300	3.53827900
H	-0.54339300	2.54835400	3.80410200
C	1.94327100	-1.00382500	0.12830900
C	3.09304700	-0.27817200	-0.27922400
C	2.16298600	-2.12827000	0.94426200
C	4.39311200	-0.65042300	0.17301900
C	2.99131900	0.87167100	-1.13292400
C	3.42227300	-2.51516500	1.37782100
H	1.29503700	-2.69807000	1.28049800
C	5.53629400	0.11466600	-0.20027800
C	4.55803500	-1.78462300	1.01385400
C	4.07787800	1.60512900	-1.48848100
H	1.99221400	1.14591400	-1.47171300
H	3.53962400	-3.38537400	2.02371300
C	5.39045600	1.25760600	-1.03427500
C	6.83066300	-0.25750500	0.26143000
C	5.87067500	-2.13808200	1.46107500
H	3.97200200	2.47307000	-2.13876100
C	6.52529900	1.99702600	-1.38703700
C	7.93771400	0.51089800	-0.11694500
C	6.95842200	-1.41009400	1.10237400
H	5.97581800	-3.01232400	2.10263700
C	7.78403700	1.62516100	-0.93212600
H	6.40671100	2.87042000	-2.02689600
H	8.92555300	0.22101500	0.23854200
H	7.95320600	-1.68716900	1.44858700
H	8.65586700	2.21035900	-1.21655800
C	-1.94330700	1.00397400	-0.12793100
C	-3.09310200	0.27823800	0.27941800
C	-2.16298600	2.12846700	-0.94383200
C	-4.39312000	0.65041000	-0.17302400
C	-2.99143200	-0.87161400	1.13311100
C	-3.42223700	2.51531700	-1.37753300
H	-1.29502900	2.69834100	-1.27992100
C	-5.53629900	-0.11481900	0.20000200
C	-4.55799800	1.78465400	-1.01380800
C	-4.07798800	-1.60520300	1.48841400
H	-1.99237400	-1.14575700	1.47211300
H	-3.53955300	3.38556900	-2.02337500
C	-5.39050800	-1.25780300	1.03394700
C	-6.83061800	0.25725600	-0.26192600
C	-5.87058900	2.13802000	-1.46124400
H	-3.97215900	-2.47314500	2.13869900
C	-6.52534400	-1.99736600	1.38643300
C	-7.93766400	-0.51128700	0.11617800
C	-6.95833200	1.40989900	-1.10280200
H	-5.97569800	3.01230000	-2.10276000
C	-7.78403100	-1.62559800	0.93130300
H	-6.40679100	-2.87079200	2.02625600
H	-8.92546400	-0.22147800	-0.23947700
H	-7.95307900	1.68690000	-1.44918400
H	-8.65585600	-2.21090500	1.21552200

30) TS-Pyr[7-12]

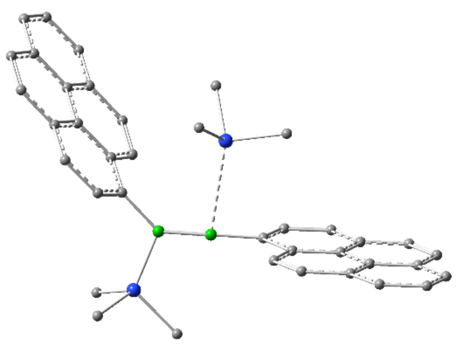

Note: Hydrogen atoms are omitted for clarity.

Number of imaginary frequencies = 1 (36.6 cm^{-1})

E_{total} = -2201.053140 a.u

$G_{correction}$ = 0.557714 a.u

Cartesian coordinates:

B	0.67020200	-0.90069800	-0.66994500
B	-0.64821800	-1.59956300	-0.30818400
P	-0.01185300	1.53295800	0.72469000
P	-0.56048700	-3.36936900	-0.95585600
C	-0.46311100	1.23078800	2.48993600
H	-1.49677100	0.87066900	2.54978300
H	0.18784300	0.45116300	2.90454700
H	-0.36711800	2.13954000	3.09950200
C	1.52399800	2.53334300	0.96456400
H	2.30517400	1.91614100	1.42531000
H	1.89857200	2.88207300	-0.00527100
H	1.34322500	3.40507500	1.60755800
C	-1.20377100	2.89086100	0.32862200
H	-1.01072300	3.27367300	-0.68015500
H	-2.22710400	2.49818100	0.34688800
H	-1.13037200	3.72082500	1.04451100
C	-0.80691100	-4.72904300	0.24432900
H	-0.04646900	-4.66353600	1.03020700
H	-1.79580000	-4.63462500	0.70655100
H	-0.73829300	-5.70738000	-0.24692000
C	1.02764800	-3.78563700	-1.74560900
H	1.22348900	-3.10302100	-2.57927400
H	1.84310400	-3.69747800	-1.01892800
H	1.00066400	-4.81292700	-2.12610900
C	-1.81062100	-3.75622700	-2.23260300
H	-2.80900400	-3.57070300	-1.81899500
H	-1.66681600	-3.08674900	-3.08785500
H	-1.74856300	-4.79895300	-2.56740400
C	1.99223700	-0.20002800	-1.03245700
C	3.14003100	-0.25176700	-0.20038400
C	2.08364900	0.52158600	-2.23569400
C	4.32696600	0.43902700	-0.57436900
C	3.13636100	-0.95889600	1.04765500
C	3.23473300	1.19378500	-2.61051100
H	1.20646900	0.55952400	-2.88105500
C	5.47011800	0.41575200	0.27336200
C	4.37337200	1.16599500	-1.79579200
C	4.22415600	-0.97661600	1.86019300
H	2.21649800	-1.47506500	1.32545300
H	3.26951900	1.74931400	-3.54683400
C	5.43119900	-0.29414200	1.50403100
C	6.65504800	1.10636500	-0.10389200
C	5.57965900	1.84862000	-2.15284300
H	4.19799900	-1.51446200	2.80709100
C	6.56460600	-0.30134800	2.32466900
C	7.76530700	1.07124000	0.74674700
C	6.67048200	1.81970600	-1.34577800
H	5.59977700	2.39730100	-3.09329400
C	7.71822600	0.37434500	1.94778900
H	6.52898300	-0.84638800	3.26678100
H	8.67024800	1.60143000	0.45373500
H	7.58291400	2.34377600	-1.62657800
H	8.59027400	0.35846500	2.59749000
C	-2.00821300	-1.20850000	0.39183000
C	-2.97818800	-0.35378900	-0.19322800
C	-2.30885600	-1.74298800	1.65622700
C	-4.18260800	-0.03663200	0.50255300
C	-2.79196000	0.21863200	-1.49644300
C	-3.48015100	-1.45283800	2.33998600
H	-1.57074800	-2.39165800	2.13013100
C	-5.14740500	0.83476800	-0.08209400
C	-4.43408500	-0.59312900	1.78653800
C	-3.70976100	1.04801700	-2.05625400
H	-1.87016400	-0.03531300	-2.01911700
H	-3.66442100	-1.88213400	3.32472600
C	-4.91882300	1.39209700	-1.37037900
C	-6.34700700	1.14966200	0.61743200
C	-5.64823400	-0.25952600	2.46752300
H	-3.54423400	1.46845300	-3.04772900
C	-5.87882100	2.24573400	-1.92647700
C	-7.27894000	2.00758100	0.02266700
C	-6.56319500	0.57383400	1.91126400
H	-5.82036000	-0.69492200	3.45124400
C	-7.04478900	2.54889800	-1.23514600
H	-5.69768800	2.66937100	-2.91340300
H	-8.19478000	2.24514400	0.56206100
H	-7.48379900	0.82145500	2.43791700
H	-7.78043100	3.21401800	-1.68220800

31) 12-Pyr

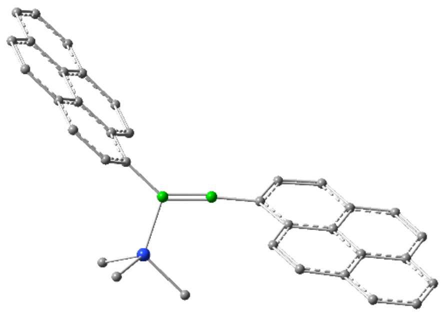

Note: Hydrogen atoms are omitted for clarity.

Number of imaginary frequencies = 0

E_{total} = -1740.069627 a.u

$G_{correction}$ = 0.452760 a.u

Cartesian coordinates:

B	-0.60812300	-0.04533100	0.19243700
B	0.62734200	-0.93491000	0.29529800
P	0.26028500	-2.24866100	1.59411900
C	0.36718800	-3.98871300	1.04420400
H	-0.36271700	-4.16234000	0.24545400
H	1.37011800	-4.18672700	0.65021200
H	0.16903900	-4.67642700	1.87536600
C	-1.37602000	-2.14963600	2.38516700
H	-1.51808900	-1.16283000	2.83714300
H	-2.17211600	-2.32205000	1.65259800
H	-1.44730500	-2.91311300	3.16827300
C	1.43826800	-2.19698900	2.98789900
H	2.45746500	-2.29847100	2.59769500
H	1.35803200	-1.22684900	3.49023200
H	1.24659100	-3.00143100	3.70837900
C	-1.89371800	0.75723900	0.16569800
C	-3.14510300	0.20261700	-0.22115100
C	-1.85758000	2.11578500	0.53958900
C	-4.32157500	0.99929100	-0.16936900
C	-3.26958400	-1.15458300	-0.66199200
C	-2.98898000	2.90961400	0.53786800
H	-0.90393400	2.54512900	0.84412200
C	-5.58616800	0.43549400	-0.49982900
C	-4.23940900	2.36818300	0.20914600
C	-4.47504600	-1.69515800	-0.97773500
H	-2.35198700	-1.73657600	-0.76093700
H	-2.92764800	3.95822900	0.82528600
C	-5.67888000	-0.92664300	-0.89343800
C	-6.76199500	1.23279900	-0.44126100
C	-5.43712900	3.14879900	0.25312000
H	-4.54523000	-2.72551300	-1.32378800
C	-6.93237600	-1.46304900	-1.20948300
C	-7.99468000	0.65563400	-0.76587100
C	-6.64333600	2.60638800	-0.05463100
H	-5.35746100	4.19514700	0.54374900
C	-8.07673800	-0.67877700	-1.14425600
H	-6.99707700	-2.50699900	-1.51248500
H	-8.89227800	1.27046800	-0.72066100
H	-7.54885400	3.21007900	-0.01592600
H	-9.04292700	-1.11069200	-1.39427200
C	2.08003500	-0.98481200	-0.32357600
C	3.08500800	-0.03108100	-0.02341000
C	2.42368300	-2.02538900	-1.20116000
C	4.37779100	-0.12911300	-0.61600000
C	2.84991900	1.05263300	0.88622900
C	3.67928500	-2.13669300	-1.78008700
H	1.66052400	-2.76188000	-1.45575300
C	5.38276000	0.83651900	-0.32016000
C	4.67593400	-1.19549400	-1.50743900
C	3.80584300	1.97302600	1.17199000
H	1.86274800	1.11027500	1.34361500
H	3.89885100	-2.95515000	-2.46540900
C	5.10568600	1.90405300	0.57702500
C	6.67060100	0.73799800	-0.91844600
C	5.97935000	-1.27089500	-2.09499800
H	3.60140600	2.78634900	1.86754900
C	6.10650100	2.84252900	0.85449900
C	7.64070400	1.69897300	-0.61296800
C	6.93335200	-0.34733200	-1.81593200
H	6.18703200	-2.09383800	-2.77801300
C	7.35922400	2.73939000	0.26381800
H	5.88722100	3.65775700	1.54267200
H	8.62409300	1.61867400	-1.07415100
H	7.92150900	-0.41328200	-2.26912200
H	8.12574000	3.47753700	0.48936700

32) TS-Pyr[12-13]

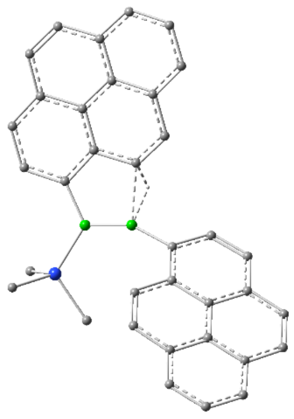

Note: Selected hydrogen atoms are omitted for clarity.

Number of imaginary frequencies = 1 (333.1 cm^{-1})

E_{total} = -1740.052762 a.u

$G_{correction}$ = 0.453864 a.u

Cartesian coordinates:

B	0.09987300	0.40918000	-0.70956900
B	0.82734200	1.80035100	-0.54292400
P	-0.06258700	3.46404700	-0.58338400
C	-0.12805500	4.40094500	0.98231900
H	-0.60296400	5.37841600	0.83571000
H	-0.69867400	3.83183200	1.72427900
H	0.88644300	4.54619800	1.36853200
C	-1.79350300	3.36355100	-1.12057600
H	-1.84932100	2.83385000	-2.07699500
H	-2.38988100	2.81479300	-0.38337300
H	-2.20720200	4.37181600	-1.23673500
C	0.70091400	4.63524300	-1.75400700
H	1.74319100	4.82123700	-1.47446600
H	0.68823200	4.19737800	-2.75755400
H	0.15615700	5.58660800	-1.76523200
C	-1.15442300	-0.47255900	-0.87254600
C	-2.30661400	-0.27390000	-0.06068400
C	-1.23490500	-1.47909800	-1.85738300
C	-3.50410800	-1.00482500	-0.30639400
C	-2.30439700	0.64483900	1.03868700
C	-2.37970700	-2.21980100	-2.07910300
H	-0.37016400	-1.65997600	-2.49786500
C	-4.67271400	-0.75850000	0.47118000
C	-3.54317400	-1.98711200	-1.33252300
C	-3.41530300	0.88262900	1.78509800
H	-1.35910200	1.13334400	1.27707800
H	-2.39932700	-2.97785300	-2.86157500
C	-4.64695400	0.20530800	1.51676900
C	-5.87271900	-1.47811300	0.20787800
C	-4.75821900	-2.69929400	-1.57125900
H	-3.37995900	1.57506800	2.62576500

C	-5.80739200	0.43545400	2.26514200
C	-7.01054800	-1.21299900	0.97987100
C	-5.87590400	-2.45471400	-0.83855000
H	-4.76787400	-3.44740500	-2.36310600
C	-6.97554200	-0.26639100	1.99555900
H	-5.77979700	1.17200300	3.06694300
H	-7.92643800	-1.76483000	0.77335300
H	-6.79816000	-3.00099400	-1.03099600
H	-7.86877000	-0.07547300	2.58639100
C	2.36600100	1.69376900	-0.31845900
C	2.78439900	0.34386600	-0.32165800
C	3.35276300	2.64024600	0.00638100
C	4.05799100	-0.06153500	0.09449800
C	1.81096600	-0.64716000	-0.67811400
C	4.64767800	2.26962500	0.35811400
H	3.10095600	3.70274900	0.02314500
C	4.34765900	-1.44670800	0.24145400
C	5.02656200	0.92223800	0.43993100
C	2.07612500	-1.98054700	-0.54555400
H	1.12032100	-0.33259900	-1.54409100
H	5.37950300	3.03484200	0.61696600
C	3.34497700	-2.41754500	-0.04190400
C	5.62928100	-1.85193900	0.69592800
C	6.31424500	0.48001900	0.87897700
H	1.35071700	-2.73140200	-0.85987800
C	3.64921500	-3.77499700	0.12340800
C	5.88525600	-3.21968200	0.84593600
C	6.60643000	-0.84353400	0.99629900
H	7.06244800	1.23230300	1.12646600
C	4.90727500	-4.16735700	0.56176200
H	2.88504500	-4.51893100	-0.09571700
H	6.86720600	-3.53693800	1.19463200
H	7.58941900	-1.16650100	1.33555900
H	5.12939200	-5.22441100	0.68713300

33) 13-Pyr

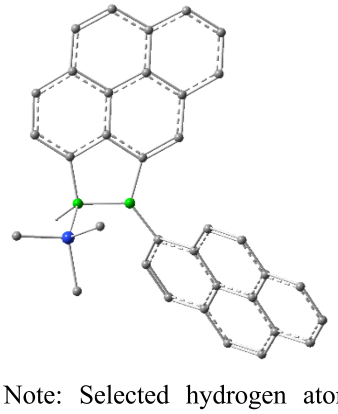

Note: Selected hydrogen atoms are omitted for clarity.

Number of imaginary frequencies = 0

E_{total} = -1740.140683 a.u

$G_{correction}$ = 0.454131 a.u

Cartesian coordinates:

B	0.29073600	0.91263800	-0.83697100
B	1.17052000	2.39707500	-0.71719800
P	0.48173600	3.30748900	0.83001700
C	0.37119800	2.26221400	2.31524600
H	0.06278500	2.84802300	3.18858600
H	-0.35964800	1.46057000	2.15246400
H	1.34883400	1.80852600	2.51482900
C	-1.20070600	3.94478800	0.57513600
H	-1.20880200	4.60482800	-0.29937400
H	-1.88513700	3.11081300	0.38287200
H	-1.54669400	4.50110800	1.45361000
C	1.44861600	4.75151400	1.37549700
H	2.44456400	4.42309300	1.69254500
H	1.56132200	5.44956500	0.53860800
H	0.95834400	5.26367500	2.21172500
C	-1.24009700	0.71538100	-1.07332700
C	-2.10594800	-0.08291400	-0.28422300
C	-1.80823700	1.43651900	-2.13839500
C	-3.49688700	-0.15205200	-0.58749400
C	-1.63938900	-0.80352000	0.86726100
C	-3.14982300	1.34209600	-2.46930600
H	-1.16077100	2.08782600	-2.72548100
C	-4.37480000	-0.93035500	0.22026800
C	-4.01842600	0.55575700	-1.70485400
C	-2.47581500	-1.53985000	1.64302400
H	-0.57832000	-0.75737800	1.10675100
H	-3.54861500	1.89681800	-3.31787600
C	-3.87274300	-1.63449500	1.34822100
C	-5.76019200	-1.00674500	-0.09527700
C	-5.41567100	0.45900300	-2.00122000
H	-2.09376100	-2.07931800	2.50864600
C	-4.75169800	-2.39264300	2.12988500
C	-6.60264900	-1.77896400	0.71201800
C	-6.24880700	-0.28796300	-1.23378600
H	-5.79561900	1.00479700	-2.86373300
C	-6.10188100	-2.46357400	1.81227600
H	-4.35990300	-2.92921800	2.99271900
H	-7.66149900	-1.83626300	0.46405400
H	-7.31022800	-0.35508100	-1.46806300
H	-6.77154000	-3.05896800	2.42866100
C	2.65795300	1.84143000	-0.42922300
C	2.65297600	0.43329700	-0.41278000
C	3.88777200	2.47274700	-0.21637800
C	3.82318400	-0.32269500	-0.21150500
C	1.37666600	-0.20107500	-0.64078700
C	5.05929200	1.74523300	0.00251500
H	3.94623700	3.56270800	-0.23608700
C	3.74602700	-1.73801700	-0.24417200
C	5.05663100	0.34420900	0.00940400
C	1.32486300	-1.57068400	-0.68734100
H	1.03107700	3.22190900	-1.60788000
H	6.00275800	2.26721700	0.16344200
C	2.49443000	-2.37641200	-0.48899900
C	4.92282200	-2.50833900	-0.04213700
C	6.22557600	-0.45940700	0.21521300
H	0.38081800	-2.08645300	-0.87471400
C	2.45412200	-3.77721300	-0.52344600
C	4.82793200	-3.90310800	-0.08335000
C	6.15984400	-1.81714200	0.19223000
H	7.17692400	0.04245200	0.38877400
C	3.60631300	-4.52591700	-0.32198600
H	1.50244800	-4.27231200	-0.71119100
H	5.72599700	-4.49971600	0.07148400

H	7.05709200	-2.41477700	0.34800800
H	3.55434400	-5.61196900	-0.35204700

34) TS-Pyr[13-8]

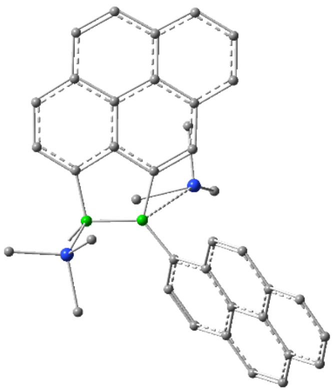

Note: Selected hydrogen atoms are omitted for clarity.

Number of imaginary frequencies = 1 (32.5 cm^{-1})

E_{total} = -2201.117872 a.u

$G_{correction}$ = 0.556747 a.u

Cartesian coordinates:

B	0.12606300	-0.84525400	0.52373300
B	0.93747100	-2.35244100	0.78451100
P	0.05766300	-3.64638700	-0.33533100
C	-0.19238900	-3.10020800	-2.05265200
H	-0.59426700	-3.91194300	-2.66957100
H	-0.89526000	-2.25844100	-2.07626600
H	0.76444300	-2.77005400	-2.47339900
C	-1.59942000	-4.08467900	0.26773900
H	-1.52404100	-4.44765700	1.29876100
H	-2.24168500	-3.19670500	0.26144800
H	-2.05104700	-4.86193200	-0.35902200
C	0.92758900	-5.23831400	-0.49871000
H	1.88756600	-5.07719400	-1.00142800
H	1.12037200	-5.65027000	0.49805100
H	0.33653300	-5.95559000	-1.08006300
C	-1.37953300	-0.51415900	0.76947400
C	-2.27107600	0.02799300	-0.18909100
C	-1.90339400	-0.84973800	2.03077300
C	-3.64392800	0.22906700	0.13772200
C	-1.85354400	0.33947100	-1.52800200
C	-3.22340200	-0.61376500	2.37535000
H	-1.23745700	-1.31185700	2.76042400
C	-4.55156200	0.73775600	-0.83502200
C	-4.11811000	-0.07844400	1.44217800
C	-2.71878800	0.81677500	-2.45899700
H	-0.80648700	0.19268600	-1.78721100
H	-3.58700600	-0.86451300	3.37124900
C	-4.09819400	1.03617900	-2.14889100
C	-5.91856500	0.94654700	-0.50017900
C	-5.49696100	0.14575200	1.75374500
H	-2.37381500	1.04767800	-3.46598300
C	-5.00620700	1.53063700	-3.09164200
C	-6.79144700	1.44569800	-1.47341000
C	-6.35833100	0.63657000	0.82723100
H	-5.83996900	-0.09096600	2.76003600
C	-6.33825100	1.73353500	-2.75468800
H	-4.65149300	1.75660700	-4.09617200
H	-7.83598700	1.60622700	-1.21046600
H	-7.40573400	0.80307100	1.07469000
H	-7.03126700	2.12053300	-3.49825000
C	2.40429100	-2.00705200	0.20466900
C	2.44839100	-0.66864900	-0.23256600
C	3.58054900	-2.75335700	0.07624700
C	3.61689500	-0.08468900	-0.75814600
C	1.22909600	0.09002800	-0.08309900
C	4.74575400	-2.20328100	-0.46172100
H	3.60295600	-3.78988100	0.41789500
C	3.59784100	1.28291100	-1.13290000
C	4.79326800	-0.86900200	-0.88635900
C	1.23569400	1.41719100	-0.43016300
H	0.86838800	-2.86142900	1.89416700
H	5.64632100	-2.81165100	-0.54751000
C	2.40709100	2.05021200	-0.96354100
C	4.77485700	1.88152800	-1.65773400
C	5.96120700	-0.24067300	-1.43026700
H	0.34051800	2.02885300	-0.29851100
C	2.42902500	3.40586300	-1.31957800
C	4.74122100	3.23572800	-2.00600400
C	5.95018500	1.06793700	-1.79876500
H	6.86790200	-0.83487000	-1.53908600
C	3.58108300	3.98554100	-1.83512100
H	1.52547200	3.99917300	-1.18671800
H	5.63938700	3.70133400	-2.40964200
H	6.84682400	1.53307400	-2.20642900
H	3.57783300	5.03859500	-2.10750600
P	0.88692400	1.69196800	3.27272700
C	2.55025000	2.33119100	2.76330100
H	3.27408500	2.29677300	3.58904600
H	2.93683000	1.72262100	1.93714300
H	2.46942700	3.36451700	2.40588200
C	0.76281500	2.52970200	4.92375000
H	1.65282700	2.35834700	5.54468400
H	0.63790900	3.60948100	4.78202400
H	-0.11754900	2.16181100	5.46324000
C	1.41759800	0.01517800	3.85907500
H	0.59460300	-0.47179600	4.39628700
H	1.68049200	-0.61930000	3.00237900
H	2.28632700	0.07155300	4.52963900

35) 8-Pyr

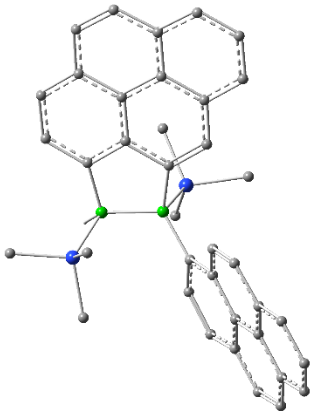

Note: Selected hydrogen atoms are omitted for clarity.

Number of imaginary frequencies = 0

E_{total} = -2201.143475 a.u

$G_{correction}$ = 0.562751 a.u

Cartesian coordinates:

B	0.23718400	-1.04141300	0.68029500
B	0.98318900	-2.33293300	-0.31358400
P	0.06327400	-2.53799200	-2.00862900
C	-0.12993800	-1.01416100	-2.98277100
H	-0.56014500	-1.23896300	-3.96559300
H	-0.78871900	-0.31117900	-2.46049000
H	0.84913300	-0.54172900	-3.12046300
C	-1.61101600	-3.24941500	-1.92596300
H	-1.58805200	-4.16599900	-1.32556300
H	-2.29903600	-2.54154800	-1.45254400
H	-1.97438700	-3.48753200	-2.93242700
C	0.93962500	-3.66047000	-3.14972100
H	1.92168400	-3.24060400	-3.39302900
H	1.08615000	-4.63179500	-2.66376100
H	0.37375600	-3.80251000	-4.07793100
C	-1.37559000	-0.87589200	0.67032300
C	-2.15050700	0.19236300	0.13806700
C	-2.11438900	-1.95663600	1.19534700
C	-3.58088000	0.18437900	0.22582200
C	-1.55624900	1.30139200	-0.55783500
C	-3.49377700	-1.98158100	1.29349800
H	-1.56969000	-2.84481100	1.51302700
C	-4.35252900	1.26503900	-0.29726800
C	-4.25854400	-0.90589000	0.83396500
C	-2.28904400	2.32191900	-1.07234400
H	-0.47860400	1.30932300	-0.67811200
H	-3.99998500	-2.84703400	1.72148300
C	-3.71225100	2.35510000	-0.94707600
C	-5.77264300	1.26075300	-0.18199500
C	-5.68492900	-0.88885100	0.93515000
H	-1.79734700	3.14097700	-1.59600700
C	-4.48321900	3.40796000	-1.45187200
C	-6.50518200	2.33679100	-0.69724300
C	-6.41292200	0.15026500	0.45508900
H	-6.17460600	-1.73763000	1.41144200
C	-5.86602700	3.39860700	-1.32362400
H	-3.98037200	4.23654900	-1.94857400
H	-7.58995700	2.32717500	-0.60022100
H	-7.49876400	0.15810700	0.53837600
H	-6.45199100	4.22562800	-1.71862100
C	2.41371000	-1.67521900	-0.65827400
C	2.46811600	-0.33017400	-0.24535600
C	3.55955000	-2.20773400	-1.26461100
C	3.61887100	0.46443700	-0.44134400
C	1.30565700	0.16979100	0.45054900
C	4.70145000	-1.43786700	-1.47849500
H	3.56715700	-3.25532300	-1.57227700
C	3.62878100	1.80474200	0.02326600
C	4.75591400	-0.09414800	-1.08300500
C	1.35020500	1.46156100	0.90715700
H	1.01719800	-3.49229000	0.09692300
H	5.57673400	-1.87832800	-1.95701800
C	2.48457900	2.31941900	0.69711500
C	4.77911400	2.61447100	-0.17848900
C	5.90172700	0.74479000	-1.27388200
H	0.49852300	1.88911900	1.44429300
C	2.52211700	3.64644100	1.14593000
C	4.76605500	3.93418600	0.28697000
C	5.90982900	2.03576800	-0.84718900
H	6.77520400	0.32407200	-1.77138000
C	3.64615900	4.43817500	0.93961700
H	1.65271500	4.05298700	1.66153900
H	5.64378000	4.56076400	0.13413300
H	6.78859500	2.66122000	-1.00055800
H	3.65020700	5.46638200	1.29583900
P	0.77855300	-1.52499600	2.53957000
C	0.25192600	-0.31356900	3.80018000
H	0.56106100	-0.63023400	4.80318200
H	0.69920000	0.66154600	3.58085500
H	-0.83891200	-0.21219200	3.77362400
C	0.24387500	-3.11716400	3.26741100
H	0.81364900	-3.30631100	4.18482500
H	-0.82028000	-3.09200100	3.51993500
H	0.42845400	-3.93314200	2.56031500
C	2.58659600	-1.61549700	2.75681100
H	3.00283200	-2.39676300	2.11191800
H	3.05494700	-0.66033900	2.49966800
H	2.81524000	-1.85352500	3.80205600

36) TS-Pyr[13-9]

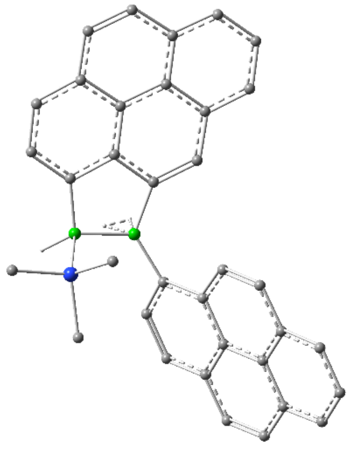

Note: Selected hydrogen atoms are omitted for clarity.

Number of imaginary frequencies = 1 (640.0 cm^{-1})

E_{total} = -1741.296435 a.u

$G_{correction}$ = 0.470105 a.u

Cartesian coordinates:

B	-0.98638300	2.41422900	0.71195100
B	-0.29252600	0.86805400	1.24034500
P	-0.10147400	3.00851600	-0.89831000
C	1.27299000	0.62217100	1.38116700
C	1.97834400	1.36526500	2.34419400
C	2.04035400	-0.18934700	0.50863200
C	3.35354800	1.31642000	2.47906600
H	1.41953100	2.01976100	3.01471900
C	3.46477000	-0.23801400	0.62520500
C	1.44249900	-0.96757400	-0.54290800
C	4.12613600	0.51880300	1.62865000
C	4.24004900	-1.04057300	-0.26182900
H	0.36038700	-0.94960500	-0.64453300
C	2.18039300	-1.72737000	-1.39220400
C	5.65728000	-1.08864100	-0.13437400
C	5.55118400	0.45188200	1.73697400
C	3.60508800	-1.79712300	-1.28419100
H	1.69304100	-2.30868900	-2.17389100
C	6.28593900	-0.31756400	0.89519700
H	6.03386600	1.03836700	2.51765500
H	7.37027100	-0.36324100	0.98466900
C	-2.48741100	1.95444600	0.35441000
C	-3.61949300	2.67836700	-0.03801700
C	-2.62095000	0.55582900	0.45218000
C	-4.82608100	2.04603600	-0.33693900
H	-3.56719500	3.76670600	-0.10385500
C	-3.82920000	-0.10521400	0.15139000
C	-1.45821100	-0.17038100	0.89822200
C	-4.95699500	0.65268000	-0.25874800
C	-3.89399200	-1.51743700	0.26348400
H	-0.72487400	1.49876000	2.61371200
C	-1.54126000	-1.52985500	1.00994700
C	-5.10584400	-2.19145900	-0.04648900
C	-6.16641100	-0.05372600	-0.56202800
C	-2.74567800	-2.24556700	0.68894600
H	-0.68398200	-2.11464500	1.35139000
C	-6.23468400	-1.40798500	-0.46290700
H	-7.03776200	0.51970700	-0.87672700
H	-7.16079600	-1.93129700	-0.69768200
C	-0.08836300	1.83280800	-2.28413100
H	-1.11088600	1.48935300	-2.47815600
H	0.30820500	2.30813500	-3.18853900
H	0.53454500	0.96490200	-2.03942400
C	-0.88523500	4.49668800	-1.59545200
H	-1.90667200	4.25601600	-1.91025700
H	-0.93057300	5.27730200	-0.82796900
H	-0.32476000	4.86979900	-2.46040500
C	1.63466700	3.48122700	-0.64359100
H	2.22837600	2.59468900	-0.39571400
H	2.04590400	3.95135600	-1.54402100
H	1.69749900	4.18430000	0.19455500
H	-0.60117200	0.76531700	2.89298000
H	-0.85825300	3.39844400	1.42898000
H	-5.69137800	2.63641700	-0.63907300
C	-2.84183500	-3.63932200	0.79014600
H	-1.97012300	-4.20545600	1.11576700
C	-4.02808500	-4.29538200	0.48294400
H	-4.08010800	-5.37867200	0.56851000
C	-5.14995200	-3.58501400	0.06904800
H	-6.07549600	-4.10829900	-0.16727100
H	3.85159800	1.90581300	3.24830800
C	6.39614300	-1.88480300	-1.01731600
H	7.47960800	-1.91835900	-0.91273500
C	5.76420200	-2.62112100	-2.01071300
H	6.35497500	-3.23459100	-2.68722100
C	4.38222300	-2.57855800	-2.14556300
H	3.88610000	-3.15499300	-2.92502200

37) 9-Pyr

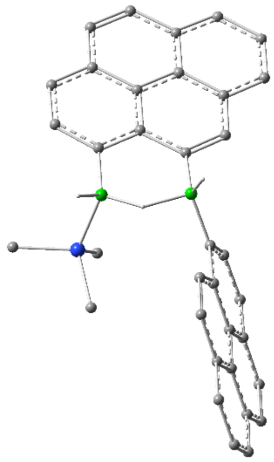

Note: Selected hydrogen atoms are omitted for clarity.

Number of imaginary frequencies = 0

E_{total} = -1741.346318 a.u

$G_{correction}$ = 0.473773 a.u

Cartesian coordinates:

B	-0.62480800	2.14000200	-0.87095400

P	0.18540700	2.61132700	0.84742600
C	-0.28654400	4.30898500	1.29043300
H	-1.37337500	4.36979300	1.41202500
H	0.19368300	4.60869400	2.22860700
H	0.01769400	4.99444700	0.49219100
C	2.00031000	2.60359000	0.84342000
H	2.38794000	3.08868600	1.74618500
H	2.36618200	1.57094500	0.80519400
H	2.36679000	3.13567000	-0.04141100
C	-0.32801400	1.60257500	2.26406800
H	0.10597700	1.99878500	3.18902100
H	-1.42054300	1.61482200	2.34350800
H	0.00544800	0.56822700	2.12425600
B	-0.21923600	-0.30098500	-1.29527800
H	-0.30954300	3.05173500	-1.60819300
H	-0.06252900	-0.33973100	-2.49294400
C	-2.16974200	1.85514300	-0.67787700
C	-3.09635200	2.88979600	-0.49972900
C	-2.63112100	0.52410200	-0.60869500
C	-4.43699400	2.64200000	-0.23720200
C	-4.00014800	0.25624300	-0.33204700
C	-1.71094000	-0.56766800	-0.83164100
C	-4.91173200	1.33013200	-0.13864100
C	-4.46237500	-1.08469600	-0.24201300
H	0.04423300	1.16535700	-1.27800700
C	-2.18368000	-1.84751100	-0.73964300
C	-3.54990600	-2.15512900	-0.44017400
H	-1.51227900	-2.69055000	-0.91875600
C	1.01015900	-0.80118600	-0.41587700
C	0.78936100	-1.51715800	0.76799800
C	2.35617100	-0.53319800	-0.77420400
C	4.24136100	-2.12232400	2.07953800
C	1.82159000	-1.96335300	1.58191000
C	3.42697000	-0.96431900	0.06340400
C	2.69626600	0.21600800	-1.95187200
C	5.52617300	-1.83290000	1.75619500
H	4.00895200	-2.68876000	2.98030900
C	3.15204700	-1.69458100	1.25323300
C	4.77745500	-0.65191700	-0.26587100
H	1.89311100	0.54214000	-2.60775500
C	3.98267400	0.51846500	-2.26407500
C	5.84047800	-1.08340200	0.57615200
H	6.34755000	-2.16263800	2.39066800
C	5.07073700	0.09905600	-1.43564600
H	4.21150300	1.08635000	-3.16502600
H	-2.75484700	3.92331300	-0.58060500
H	-5.13611400	3.46764400	-0.10665400
C	-6.28390700	1.03003800	0.14353400
C	-6.72240400	-0.25147100	0.23214200
H	-7.76826800	-0.46647500	0.44753600
C	-5.82887700	-1.35614300	0.04343700
H	-6.97282400	1.86187300	0.28599800
C	-4.02081300	-3.47055500	-0.34649000
H	-3.32229700	-4.29187700	-0.50106300
C	-5.35645800	-3.72922700	-0.06465000
H	-5.70316800	-4.75818700	0.00328700
C	-6.25457400	-2.68614800	0.12897800
H	-7.30111100	-2.89328400	0.34810700
H	-0.23836300	-1.73322000	1.05927400
H	1.60376700	-2.52340100	2.49077300
C	7.15736300	-0.76165600	0.23204900
C	7.43397400	-0.02780400	-0.91526500
H	8.46427800	0.21230300	-1.16752300
C	6.40317300	0.40013800	-1.74118900
H	6.62015400	0.97463200	-2.64049900
H	7.96702400	-1.09582600	0.87912800

38) TS-Pyr[9-14]

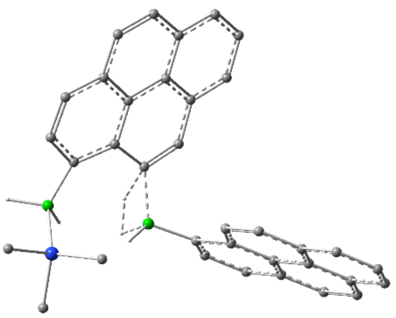

Note: Selected hydrogen atoms are omitted for clarity.

Number of imaginary frequencies = 1 (1405.3 cm^{-1})

E_{total} = -1742.463448 a.u

$G_{correction}$ = 0.489149 a.u

Cartesian coordinates:

B	-0.28354600	-1.23021600	-1.45060200
H	-0.70010900	-2.04254900	-0.68369300
C	-3.40425100	-0.83783100	-0.37815300
C	-4.53766500	-0.63422300	0.42916600
C	-2.51835300	0.25915000	-0.54519300
C	-4.37923100	2.96283900	1.35742700
C	-4.84095000	0.56608600	1.04080100
C	-2.88101600	1.54149200	-0.00435600
C	-1.25047900	0.19482500	-1.27086900
C	-3.64533200	4.06862300	1.08448900
H	-5.26234900	3.02401000	1.99217100
C	-4.03767900	1.68380900	0.81273500
C	-2.12083000	2.71526600	-0.29079600
C	-0.58214400	1.34533000	-1.58511700
C	-2.49795700	3.98336600	0.23509200
H	-3.91857200	5.03999100	1.49351600
C	-0.98254700	2.63353100	-1.13107600
H	0.34701200	1.28782300	-2.15297100
C	1.27515700	-0.95804400	-1.54336700
C	1.96213100	-1.05355500	-2.75756300
C	2.02662600	-0.65821600	-0.38338100
C	3.33467200	-0.86421000	-2.85598700
C	3.43547700	-0.47348200	-0.46489900
C	1.40612600	-0.51949600	0.90417500
C	4.09547600	-0.57814100	-1.72059900
C	4.19440300	-0.18210700	0.70407700
H	0.32490400	-0.63062700	0.97330100
C	2.12769700	-0.24228300	2.02016200
C	3.54708600	-0.06723400	1.96402000
H	1.63431800	-0.13828500	2.98560500
H	-0.63942100	-1.72750300	-2.58324200
H	-1.11310200	-0.81041600	-2.34715000

B	-3.33762200	-2.28721800	-1.07719400
H	-2.61684600	-2.43789000	-2.03347800
H	-4.46318500	-2.68233400	-1.34548800
P	-2.80750800	-3.66475700	0.22419800
C	-2.10259200	-5.12267200	-0.59918400
H	-2.81510200	-5.48915900	-1.34640400
H	-1.18123700	-4.83192600	-1.11598700
H	-1.88233600	-5.92272900	0.11626200
C	-1.63265300	-3.24163900	1.55201100
H	-1.63217100	-4.02662500	2.31701700
H	-0.61902500	-3.13096700	1.15549200
H	-1.93436600	-2.29344400	2.01254600
C	-4.24279200	-4.30359700	1.14556300
H	-4.64896800	-3.51281200	1.78612100
H	-5.02154000	-4.61572400	0.44188500
H	-3.95782500	-5.15479400	1.77442300
H	-5.23236400	-1.46729300	0.54776900
H	1.40141900	-1.29100500	-3.66246400
C	-1.74316200	5.11429600	-0.09850300
H	-2.04241000	6.07953700	0.30752000
C	-0.63673500	5.01913700	-0.93242700
H	-0.06563700	5.91101400	-1.17925200
C	-0.25495500	3.78685500	-1.44435100
H	0.61946900	3.69965800	-2.08706300
C	5.51466900	-0.38950000	-1.77919300
H	6.00340400	-0.47352900	-2.74896700
C	6.23583700	-0.11563300	-0.66365200
H	7.31437100	0.02511800	-0.71901200
C	5.60314800	-0.00188600	0.61755100
C	6.32677000	0.28136100	1.78055200
H	7.40487000	0.41830100	1.70961800
C	5.68534900	0.38901200	3.00875100
H	6.26417000	0.61068200	3.90255100
C	4.31048800	0.21788100	3.10200100
H	3.80845000	0.30610800	4.06435600
H	3.83403600	-0.94540500	-3.82083900
H	-5.73015300	0.66666000	1.66243000

39) 14-Pyr

14-Pyr comprises two components: pyren-1-ylborane (**14-Pyr**) and its PMe_3-coordinated version (**10-Pyr**). Structures and energies of these compounds are given seperately (see below).

40) 14-Pyr

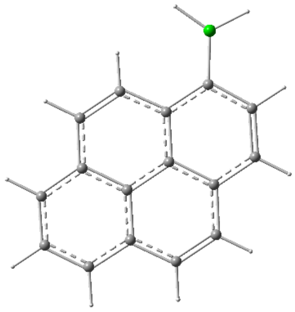

Number of imaginary frequencies = 0

E_{total} = -640.746262 a.u

$G_{correction}$ = 0.181386 a.u

Cartesian coordinates:

C	-3.67473400	-0.62869100	-0.00000100
C	-2.75140400	-1.66553200	-0.00000100
C	-1.37688900	-1.39981700	0.00000000
C	-0.92879100	-0.05308200	0.00000000
C	-1.88114900	1.00209800	0.00000100
C	-3.24510500	0.69303700	0.00000000
C	-0.39773000	-2.44054500	0.00000100
C	0.46501400	0.23580600	0.00000000
C	1.42264100	-0.81787600	0.00000000
C	0.93206300	-2.16378800	0.00000100
C	2.81404900	-0.51092000	-0.00000100
C	3.18966600	0.84688900	0.00000000
C	2.26841900	1.87589600	-0.00000100
C	0.89660100	1.59270500	0.00000000
C	-0.08309700	2.63578600	0.00000000
C	-1.41066200	2.35382500	0.00000100
H	-2.14745800	3.15565300	0.00000300
H	0.26588200	3.66692800	-0.00000100
H	-0.74371600	-3.47316800	0.00000100
H	-4.73893000	-0.85149800	-0.00000200
H	-3.08703700	-2.70135600	-0.00000100
H	-3.96909100	1.50635100	0.00000000
H	1.65274300	-2.97656600	0.00000100
H	4.25243200	1.08446000	0.00000000
H	2.59376800	2.91488400	-0.00000100
B	3.93493000	-1.55210400	0.00000000
H	3.74397800	-2.73171400	0.00000400
H	5.06942800	-1.16818600	-0.00000300

41) 10-Pyr

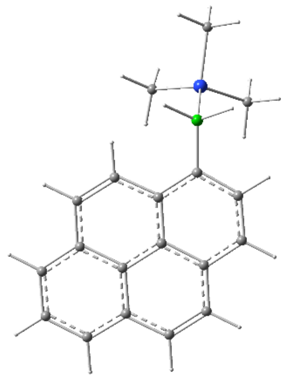

Number of imaginary frequencies = 0

E_{total} = -1101.764452 a.u

$G_{correction}$ = 0.286391 a.u

Cartesian coordinates:

C	-4.54784500	-1.78685300	0.39822300
C	-3.36589400	-2.38524600	-0.01879600
C	-2.22154100	-1.61616100	-0.25859900
C	-2.27669900	-0.20789000	-0.07130400
C	-3.49315500	0.39599600	0.35554000
C	-4.61277100	-0.41154300	0.58412000
C	-0.98844400	-2.19562600	-0.69636300
C	-1.12145300	0.59185000	-0.30750200
C	0.10312900	-0.00678700	-0.72826400
C	0.11047700	-1.43034200	-0.91989800
C	1.25969500	0.78185900	-0.94533600
C	1.13341600	2.16412800	-0.74216100
C	-0.05070500	2.76477200	-0.34095500
C	-1.19582700	1.99764100	-0.11090600
C	-2.43279100	2.57984500	0.31473900
C	-3.53160000	1.81643500	0.53887200
H	-4.46766200	2.26888800	0.86326700
H	-2.46927500	3.65945200	0.45599800
H	-0.95502300	-3.27254700	-0.85783900
H	-5.42889000	-2.39846000	0.57982500
H	-3.31642000	-3.46317800	-0.16667000
H	-5.54054000	0.05610500	0.91081300
H	1.03002000	-1.89063800	-1.27431400
H	2.00808400	2.79240700	-0.91434800
H	-0.10131500	3.84428100	-0.19933200
B	2.69695000	0.18852300	-1.34218200
H	2.72203200	-0.79500500	-2.05860000
H	3.45820600	1.05052500	-1.75394000
P	3.62062800	-0.37184300	0.27741500
C	5.33502000	-0.85861000	-0.07647800
H	5.86402100	-1.15855300	0.83498700
H	5.33317100	-1.69342700	-0.78575700
H	5.85972900	-0.01651900	-0.54028800
C	2.91604700	-1.78193200	1.18554900
H	1.88077500	-1.56333300	1.46929500
H	2.91995700	-2.67114500	0.54576300
H	3.49924200	-1.99085100	2.08992200
C	3.74816300	0.95202300	1.51584500
H	4.22305700	1.82869400	1.06123900
H	2.74488200	1.23723400	1.85071700
H	4.33843300	0.62974600	2.38098300

42) TS-Pyr[9-15]

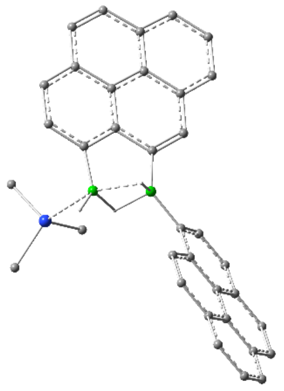

Note: Selected hydrogen atoms are omitted for clarity.

Number of imaginary frequencies = 1 (345.4 cm^{-1})

E_{total} = -1741.312545 a.u

$G_{correction}$ = 0.471732 a.u

Cartesian coordinates:

B	-0.64692800	1.81958000	-0.95054500
P	-1.08865300	3.34296700	1.18850300
C	-0.21815100	4.82040500	1.86291100
H	-0.42173000	5.69194900	1.23166700
H	-0.53947300	5.04168600	2.88897200
H	0.86284000	4.64492500	1.86019400
C	-0.80232100	2.11687100	2.53094400
H	-1.09943800	2.51006300	3.51152200
H	-1.38423400	1.20927900	2.32673100
H	0.25853000	1.84185200	2.56721700
C	-2.84779000	3.78064500	1.50190800
H	-2.99452900	4.10740100	2.53946500
H	-3.16651600	4.58382700	0.82841600
H	-3.48140600	2.90639100	1.31156500
B	-0.00385100	0.00333300	-0.53906600
H	0.02692800	2.59683600	-1.54441600
H	0.03502300	0.37531100	-1.71647000
C	-2.15974400	1.47944800	-1.12114800
C	-3.18234800	2.30912200	-1.59817900
C	-2.49679600	0.18509800	-0.68930600
C	-4.50458000	1.88388000	-1.61592400
C	-3.83390500	-0.26136500	-0.69899000
C	-1.41566500	-0.67539300	-0.27846600
C	-4.86003800	0.60374500	-1.15961300
C	-4.13042100	-1.57510900	-0.25405700
H	-0.08690000	1.26705300	0.05415900
C	-1.72041800	-1.94135100	0.13210700
C	-3.07363100	-2.42733100	0.17017200
H	-0.93143200	-2.63699900	0.42289200
C	1.36517900	-0.48177100	0.09323400
C	1.32512200	-1.15625200	1.32157900
C	2.63783900	-0.24479900	-0.48438200
C	4.92490300	-1.85486200	2.04763800
C	2.46523000	-1.60934500	1.96736200

C	3.81978100	-0.70377300	0.17057000
C	2.80314100	0.46114200	-1.72403900
C	6.14479200	-1.63324100	1.49863900
H	4.82930900	-2.38727500	2.99295800
C	3.72721400	-1.39895000	1.40778900
C	5.10417800	-0.46800800	-0.39937700
H	1.91740000	0.82116000	-2.24019200
C	4.02621200	0.68963900	-2.26610000
C	6.27884500	-0.93060700	0.25761200
H	7.04973700	-1.98427200	1.99247000
C	5.22212700	0.23488300	-1.62816800
H	4.11795700	1.22785200	-3.20858500
H	-2.93928300	3.31432300	-1.94372400
H	-5.28765700	2.54552200	-1.98460500
C	-6.20794900	0.11880200	-1.14277500
C	-6.49933400	-1.13486200	-0.70397000
H	-7.52897700	-1.48998200	-0.69698000
C	-5.47460800	-2.02922100	-0.24799100
H	-7.00076200	0.77869700	-1.49256000
C	-3.39353500	-3.72026300	0.59935400
H	-2.59164500	-4.38264700	0.92251900
C	-4.71291000	-4.16113300	0.61298300
H	-4.93855300	-5.16986300	0.95136000
C	-5.74479300	-3.32960700	0.19489300
H	-6.77454500	-3.68391400	0.20399900
H	0.35744300	-1.32732000	1.79180400
H	2.38865100	-2.13202500	2.92013700
C	7.52794200	-0.68577200	-0.32238900
C	7.63161200	0.00204400	-1.52523200
H	8.61124700	0.18325000	-1.96154900
C	6.49167300	0.45929900	-2.17272200
H	6.57205600	0.99901000	-3.11516800
H	8.42252400	-1.04319200	0.18552700

43) 15-Pyr

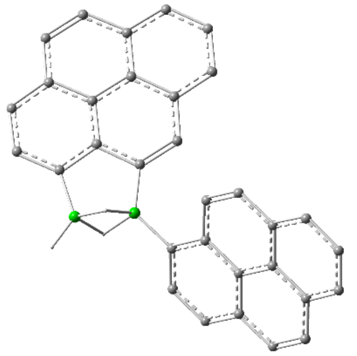

Number of imaginary frequencies = 0

E_{total} = -1280.345602 a.u

$G_{correction}$ = 0.370028 a.u

Cartesian coordinates:

B	0.29457200	-1.64914600	0.00252900
H	0.39217200	-2.11568400	-1.27130700
C	2.67518600	-2.39819500	-0.79504300
C	3.88066400	-2.93559600	-1.25471900
C	2.69105300	-1.09542100	-0.27217300
C	5.06251000	-2.20032400	-1.19418700
C	3.87170000	-0.33948500	-0.19560100
C	1.43330800	-0.57812100	0.19151100
C	5.08687700	-0.89927000	-0.66768100
C	3.82064900	0.96466000	0.35498300
C	1.39644300	0.67847500	0.72703600
C	2.58113200	1.48569200	0.82381900
H	0.45926200	1.10176200	1.09291100
C	-1.22082300	-1.55249300	0.40323400
C	-1.74446800	-2.43907000	1.35183000
C	-2.08800100	-0.58684200	-0.15746200
C	-3.06710300	-2.38583200	1.76502300
C	-3.45084700	-0.52820800	0.25123900
C	-1.64901600	0.34131200	-1.16111300
C	-3.94104900	-1.43705400	1.22896100
C	-4.33271500	0.43432200	-0.31612200
H	-0.60989100	0.30553100	-1.48478200
C	-2.48922600	1.25859600	-1.70412100
C	-3.86014900	1.34186400	-1.30223900
H	-2.13156500	1.95235200	-2.46351400
B	1.22570600	-2.99052000	-0.73837200
H	0.75901600	-2.81004200	0.49268600
H	0.85892700	-4.06934000	-1.08284800
H	3.90442200	-3.94370000	-1.66647200
H	-1.09137800	-3.19520300	1.78738400
H	5.99272100	-2.63523000	-1.55765400
C	6.27119900	-0.09688300	-0.57457700
H	7.21100100	-0.51334600	-0.93416400
C	6.23091600	1.15716800	-0.04964900
H	7.13902700	1.75496600	0.01482500
C	5.00900600	1.73453200	0.43544800
C	2.56436400	2.77701400	1.36575600
H	1.62211400	3.18540000	1.72791100
C	3.72975100	3.53083000	1.44292900
H	3.69540200	4.53162000	1.86679700
C	4.93950700	3.01965600	0.98433000
H	5.84705400	3.61814300	1.04970200
H	-3.43901200	-3.08733400	2.51034300
C	-5.31432300	-1.35673600	1.62890500
H	-5.67173800	-2.05957800	2.37993500
C	-6.15093100	-0.43529000	1.09017800
H	-7.19339500	-0.38255700	1.40051100
C	-5.69224400	0.49134900	0.09826300
C	-6.53984300	1.44576500	-0.47337700
H	-7.57954100	1.48587800	-0.15215600
C	-6.06843400	2.32988300	-1.43627000
H	-6.74214300	3.06502100	-1.87031500
C	-4.74323300	2.27996200	-1.84850700
H	-4.37500400	2.97210500	-2.60420900

44) TS-Pyr[12-17]

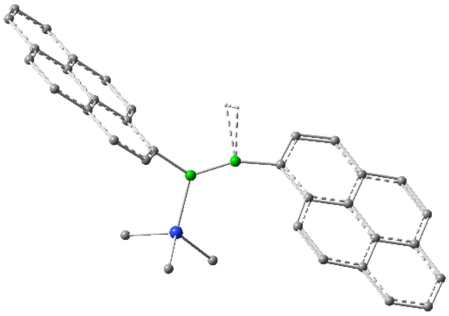

Note: Selected hydrogen atoms are omitted for clarity.

Number of imaginary frequencies = 1 (617.8 cm^{-1})

E_{total} = -1741.235731 a.u

$G_{correction}$ = 0.465150 a.u

Cartesian coordinates:

B	0.55463100	-0.56756000	0.14976800
P	0.06279200	-1.73705900	1.56831600
C	-0.05902300	-3.51478200	1.16516400
H	0.89859800	-3.86590300	0.76586300
H	-0.30621300	-4.09771900	2.06062500
H	-0.83449300	-3.67288300	0.40768700
C	-1.50482000	-1.36360100	2.40784700
H	-1.51768800	-0.31449300	2.71973600
H	-2.35287400	-1.54365400	1.73841100
H	-1.60742200	-2.00439900	3.29113900
C	1.30687100	-1.70930700	2.90063100
H	1.37102700	-0.69996300	3.32080200
H	1.04481000	-2.41700500	3.69591500
H	2.28663200	-1.97660400	2.48971800
B	-0.53739500	0.44835700	-0.26398500
H	0.28247000	1.27857300	-1.65913100
H	0.54565500	1.65679000	-1.03783300
C	-1.91432100	1.12925800	-0.33686600
C	-3.10779100	0.36522100	-0.45233800
C	-2.04900000	2.52690300	-0.22345000
C	-4.38068300	0.99810500	-0.35659000
C	-3.08501100	-1.05019400	-0.68091300
C	-3.27875800	3.15362600	-0.17810700
H	-1.14675500	3.13383900	-0.14734100
C	-5.57619500	0.22457800	-0.40462800
C	-4.46634800	2.40876900	-0.21356000
C	-4.22258600	-1.79109300	-0.73038900
H	-2.11059600	-1.51202800	-0.84352000
H	-3.33952500	4.23708700	-0.08217300
C	-5.50997300	-1.18588600	-0.57141500
C	-6.84440700	0.85953700	-0.28918300
C	-5.75303800	3.02208200	-0.10853000
H	-4.17724700	-2.86256900	-0.92281600
C	-6.69576200	-1.92756100	-0.60941500
C	-8.00547200	0.07780700	-0.32988500
C	-6.89177200	2.28219900	-0.13951500
H	-5.79835800	4.10482200	0.00098600
C	-7.92929400	-1.29979300	-0.48693800
H	-6.63793500	-3.00706400	-0.74134800
H	-8.97328700	0.56879400	-0.23953800
H	-7.86700200	2.75922500	-0.05439500
H	-8.84180200	-1.89090400	-0.51840500
C	2.03648800	-0.80219900	-0.35515600
C	3.12983400	0.04235000	-0.03826200
C	2.30426700	-1.92556300	-1.15463700
C	4.43596400	-0.24542300	-0.53353000
C	2.97443900	1.20145200	0.79427300
C	3.56949200	-2.22003100	-1.64016000
H	1.47321700	-2.57914000	-1.42409200
C	5.53299400	0.60620600	-0.21595700
C	4.65557800	-1.39141400	-1.34554500
C	4.01816900	2.01425400	1.09900600
H	1.97465700	1.41275900	1.17268000
H	3.72868300	-3.09705300	-2.26712400
C	5.33499000	1.75119000	0.60366500
C	6.83447700	0.31538200	-0.71396300
C	5.97424000	-1.65983800	-1.83419300
H	3.87249500	2.88739700	1.73405000
C	6.42587300	2.57459800	0.90469800
C	7.89620800	1.16635200	-0.38759300
C	7.01658100	-0.84505000	-1.53422900
H	6.12045700	-2.54093500	-2.45783500
C	7.69159200	2.28346000	0.41261700
H	6.26698600	3.45001500	1.53284400
H	8.88978700	0.93893700	-0.77125300
H	8.01604100	-1.05744000	-1.91115400
H	8.52879000	2.93386800	0.65612100

45) 17-Pyr

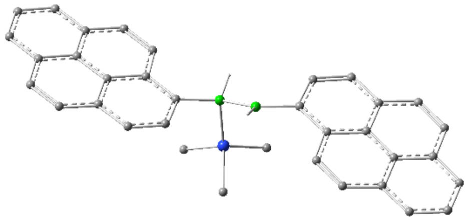

Note: Selected hydrogen atoms are omitted for clarity.

Number of imaginary frequencies = 0

E_{total} = -1741.321264 a.u

$G_{correction}$ = 0.470977 a.u

Cartesian coordinates:

B	-0.64733500	-0.40255300	-0.06459300
P	-0.21659600	-0.33101000	1.83557200
C	0.32205600	1.29911300	2.44243700
H	-0.45902000	2.04246400	2.24948900
H	0.52352000	1.26664900	3.51933800
H	1.23680500	1.60478100	1.92000500
C	1.10601000	-1.46192900	2.36599100
H	0.85756900	-2.48538700	2.06510300
H	2.05569700	-1.18338700	1.89706500
H	1.21814100	-1.42399700	3.45572500
C	-1.62501400	-0.75724900	2.90674800
H	-1.95924100	-1.77687000	2.68639600
H	-1.34118000	-0.69199400	3.96356100

H	-2.45603500	-0.07042400	2.71358100
B	0.73205600	0.24971300	-0.82599800
H	0.68459000	1.32921800	-1.36915300
H	-0.68628500	-1.61517500	-0.21131300
C	2.12786800	-0.44858000	-0.80927300
C	3.35047300	0.25964200	-0.65767800
C	2.19676600	-1.84827600	-0.92344200
C	4.58684600	-0.44593200	-0.59363000
C	3.39315100	1.68842300	-0.52293900
C	3.39627300	-2.54008100	-0.91124300
H	1.26627800	-2.40340700	-1.03390000
C	5.81093400	0.25418800	-0.39423500
C	4.60636100	-1.86164900	-0.73096800
C	4.55924800	2.35759700	-0.33433500
H	2.45755700	2.23973900	-0.58841100
H	3.41056800	-3.62359200	-1.02312900
C	5.80983200	1.66802700	-0.25572700
C	7.04189400	-0.45607500	-0.33264600
C	5.85982200	-2.55093700	-0.67144300
H	4.56365000	3.44316900	-0.24553500
C	7.02224500	2.33827100	-0.05707100
C	8.23076500	0.25380300	-0.13375700
C	7.02368100	-1.88070600	-0.47954300
H	5.85484600	-3.63418400	-0.78309100
C	8.21901700	1.63651300	0.00295800
H	7.01365700	3.42214500	0.04769800
H	9.17014700	-0.29521600	-0.08963700
H	7.97140100	-2.41520000	-0.43439000
H	9.15295600	2.17207800	0.15642800
C	-2.04745900	0.38321400	-0.22813700
C	-3.32190400	-0.23914000	-0.29126400
C	-2.03020100	1.78662900	-0.25628200
C	-4.51413200	0.54362600	-0.37224200
C	-3.47728100	-1.66715900	-0.27323500
C	-3.17704900	2.56208600	-0.33370700
H	-1.06436600	2.29307100	-0.23000700
C	-5.79423100	-0.08184500	-0.42318500
C	-4.43732500	1.96298600	-0.39170000
C	-4.69447700	-2.26596900	-0.32314700
H	-2.57896500	-2.27674600	-0.22614100
H	-3.10733600	3.64960700	-0.35513700
C	-5.89938600	-1.49907100	-0.39702800
C	-6.97817800	0.70575400	-0.49676200
C	-5.64278000	2.73157700	-0.46895200
H	-4.77602300	-3.35235600	-0.31206600
C	-7.16441700	-2.09608800	-0.44458900
C	-8.22249700	0.06743500	-0.54227800
C	-6.85883700	2.13298800	-0.51956300
H	-5.55740000	3.81749900	-0.48645500
C	-8.31281300	-1.31869300	-0.51674100
H	-7.23443200	-3.18280400	-0.42509200
H	-9.12371700	0.67645100	-0.59817600
H	-7.77070000	2.72565800	-0.57806300
H	-9.28890300	-1.79739200	-0.55370200

46) TS-Pyr[17-9]

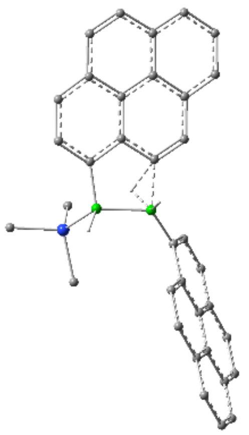

Note: Selected hydrogen atoms are omitted for clarity.

Number of imaginary frequencies = 1 (1135.7 cm^{-1})

E_{total} = -1741.270520 a.u

$G_{correction}$ = 0.471559 a.u

Cartesian coordinates:

B	-0.62837100	1.88957400	-0.40302000
P	-0.17591400	2.68372700	1.30144700
C	-0.70440100	4.41782300	1.45312900
H	-1.79855000	4.46789000	1.42204900
H	-0.35260300	4.86060100	2.39204300
H	-0.30472400	4.99212200	0.61005400
C	1.61891300	2.69431300	1.57015900
H	1.87883400	3.25594100	2.47436200
H	1.97792300	1.66302900	1.66711900
H	2.11165100	3.14858000	0.70329500
C	-0.88000300	1.86392200	2.76471800
H	-0.60583500	2.40119100	3.67962600
H	-1.97231000	1.83351600	2.68134300
H	-0.50294900	0.83685600	2.83505500
B	-0.10718800	0.10168100	-0.59467900
H	-0.01058600	2.58460000	-1.18646500
H	-0.32229500	0.12511100	-1.77004900
C	-2.21639700	1.76110500	-0.61028100
C	-3.17096500	2.73725000	-0.91948900
C	-2.69186600	0.43995200	-0.45812800
C	-4.52322900	2.42773000	-1.05129900
C	-4.05519200	0.10647600	-0.55401300
C	-1.72222800	-0.58247400	-0.17116400
C	-4.99661100	1.12156400	-0.86172300
C	-4.46305400	-1.23105800	-0.30584000
H	-0.89277600	0.29454700	0.38067700
C	-2.10760900	-1.85589600	0.11942300
C	-3.49679800	-2.22059900	0.04212100
H	-1.38029000	-2.63192300	0.35813300
C	1.19484400	-0.54700000	0.02031200
C	1.15273100	-1.29972200	1.20403300
C	2.47033500	-0.31308700	-0.55108500
C	4.75137900	-2.10297400	1.85652100
C	2.29107500	-1.80925300	1.81301400
C	3.64829400	-0.82933500	0.06167300

C	2.62711200	0.47085800	-1.74343400
C	5.96944600	-1.87189500	1.30551400
H	4.65841800	-2.68802800	2.77083600
C	3.55514700	-1.59084400	1.25921500
C	4.92915700	-0.58119800	-0.51040800
H	1.73395700	0.87835200	-2.21197600
C	3.84683300	0.71097200	-2.28930300
C	6.10281500	-1.10149500	0.10459400
H	6.87342100	-2.26698900	1.76689500
C	5.04226400	0.19236200	-1.69772800
H	3.93814500	1.30713500	-3.19639200
H	-2.85097100	3.76944400	-1.06890800
H	-5.23917900	3.21226100	-1.29577100
C	-6.37753400	0.75057200	-0.95053700
C	-6.77628400	-0.52994600	-0.72859500
H	-7.82975100	-0.79783800	-0.79769300
C	-5.84058600	-1.56536700	-0.39111700
H	-7.10746700	1.52034100	-1.19780000
C	-3.93871100	-3.52661400	0.28670800
H	-3.20807600	-4.29027800	0.54889600
C	-5.28750600	-3.85014500	0.19420900
H	-5.60817000	-4.87165000	0.38620100
C	-6.23113400	-2.88552200	-0.13915800
H	-7.28579600	-3.14832200	-0.20673500
H	0.18516200	-1.49723900	1.66882300
H	2.21006600	-2.39148000	2.73075900
C	7.34886200	-0.84442700	-0.47731400
C	7.44848700	-0.08993200	-1.63998600
H	8.42575700	0.09899300	-2.07878400
C	6.30955700	0.42537500	-2.24474400
H	6.38894400	1.01789900	-3.15508800
H	8.24372300	-1.24577300	-0.00369600

Mechanistic pathways computed for the hydrogenation of III

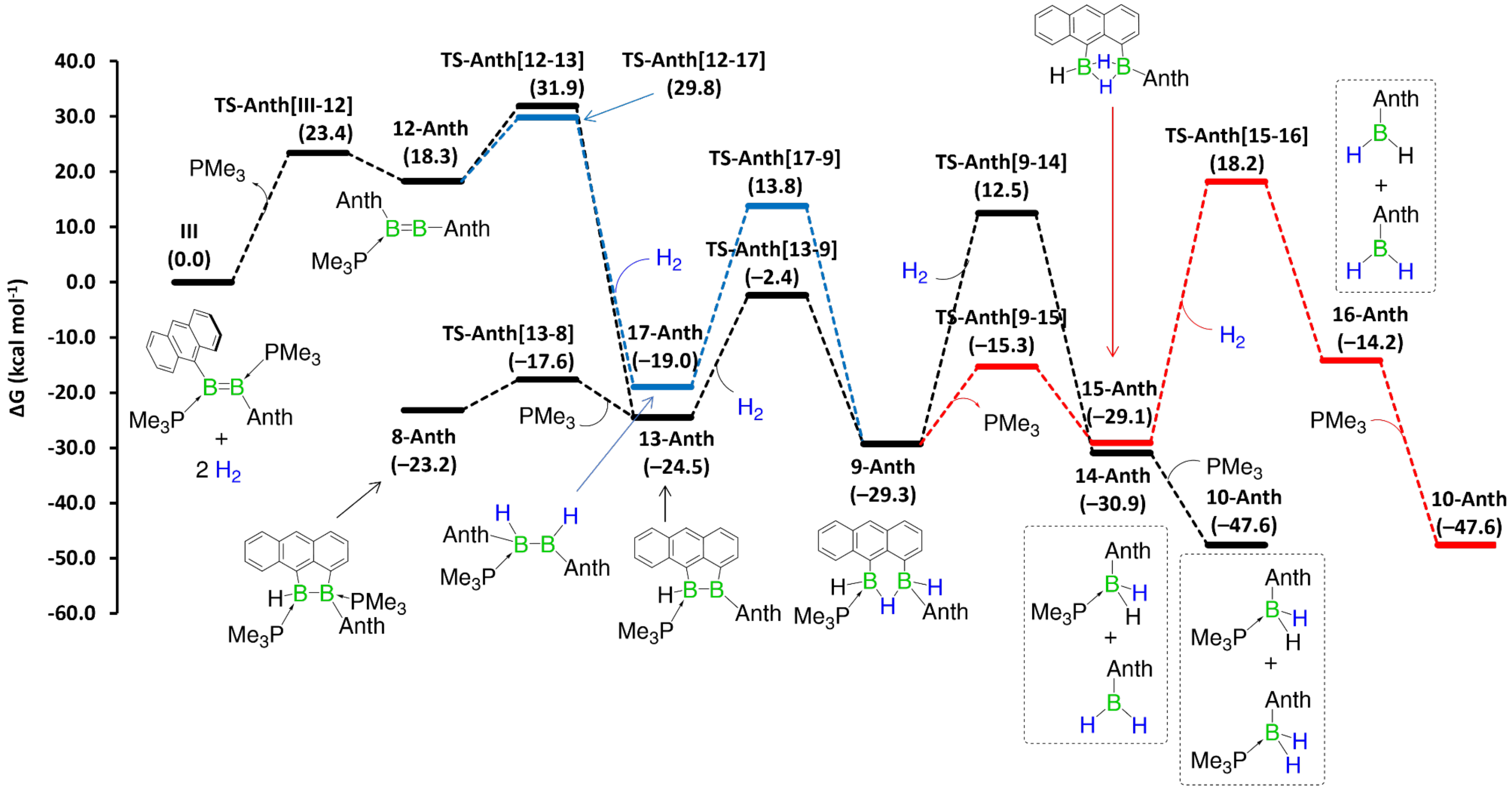

Figure S81. Computed mechanism for the formation of the borane **10-Anth** from the hydrogenation of diborene **III**.

Structures, energies and cartesian coordinates involving III in the hydrogenation:

47) Diborene III

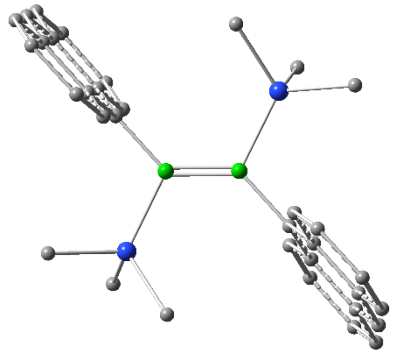

Note: Hydrogen atoms are omitted for clarity.

Number of imaginary frequencies = 0

E_{total} = -2048.711788 a.u

$G_{correction}$ = 0.536451 a.u

Cartesian coordinates:

B	0.61151500	-0.01210000	-0.49544200
B	-0.61151900	-0.01213500	0.49534400
C	2.18697000	0.01827800	-0.24613500
C	2.93238700	-1.16578100	-0.01040800
C	2.90087500	1.24554700	-0.25684100
C	2.30694200	-2.45087100	0.01383200
C	4.35282300	-1.12215600	0.22899000
C	4.32131900	1.28912100	-0.02833500
C	2.23830900	2.49190000	-0.47767700
C	3.01363000	-3.59606100	0.25473700
H	1.23132800	-2.48256100	-0.16000600
C	5.05805800	-2.33717500	0.47628500
C	5.01394300	0.10397700	0.21464600
C	4.99619100	2.54523000	-0.05083800
H	1.15750900	2.46445000	-0.61861700
C	2.91579600	3.67895700	-0.49483700
C	4.41321800	-3.53945700	0.49006000
H	2.50575900	-4.55828100	0.26569600
H	6.13129200	-2.27921900	0.65576400
H	6.08970500	0.13658600	0.39237900
C	4.31925000	3.70740700	-0.28168800
H	6.07196700	2.55243500	0.12245700
H	2.37971200	4.61047900	-0.66447200
H	4.96621000	-4.45675500	0.68082800
H	4.84901800	4.65755800	-0.29589600
C	-2.18698300	0.01828000	0.24608700
C	-2.93243200	-1.16576200	0.01036800
C	-2.90087700	1.24555300	0.25691300
C	-2.30700900	-2.45086200	-0.01393100
C	-4.35287800	-1.12210900	-0.22896300
C	-4.32133000	1.28915600	0.02847400
C	-2.23829000	2.49188500	0.47780900
C	-3.01372500	-3.59603500	-0.25484100
H	-1.23139000	-2.48257400	0.15986800
C	-5.05814300	-2.33710900	-0.47626800
C	-5.01398000	0.10403400	-0.21454100
C	-4.99618800	2.54527100	0.05108300
H	-1.15748400	2.46441200	0.61870400
C	-2.91576400	3.67894700	0.49507100
C	-4.41332300	-3.53940100	-0.49010600
H	-2.50587100	-4.55826200	-0.26584500
H	-6.13138400	-2.27913100	-0.65569600
H	-6.08975000	0.13666400	-0.39221800
C	-4.31922600	3.70742600	0.28197900
H	-6.07197200	2.55249800	-0.12216500
H	-2.37966300	4.61045400	0.66474600
H	-4.96633800	-4.45668400	-0.68087400
H	-4.84898300	4.65758200	0.29627000
P	0.19205300	-0.10050000	-2.35754900
P	-0.19200500	-0.10071400	2.35742200
C	-1.37711300	0.66094000	-2.88445800
H	-2.22567800	0.14904800	-2.41927800
H	-1.39640600	1.71267500	-2.57885300
H	-1.47248800	0.59665200	-3.97495500
C	1.43005000	0.66959600	-3.45889200
H	1.52066000	1.73870100	-3.23886000
H	2.40731500	0.20145100	-3.29709200
H	1.14316300	0.54109400	-4.50920100
C	0.08092800	-1.79534900	-3.04148200
H	1.03252100	-2.31552500	-2.88296200
H	-0.71079500	-2.34685200	-2.52102000
H	-0.14039000	-1.77426300	-4.11600000
C	-1.42983500	0.66951400	3.45886100
H	-1.52030900	1.73864000	3.23887600
H	-2.40717400	0.20151100	3.29709600
H	-1.14290900	0.54093100	4.50915000
C	1.37731000	0.66045900	2.88427000
H	2.22576300	0.14848100	2.41897600
H	1.39673000	1.71221400	2.57874000
H	1.47276900	0.59607400	3.97475300
C	-0.08109500	-1.79561900	3.04125300
H	-1.03276500	-2.31565300	2.88273000
H	0.71053700	-2.34720300	2.52073900
H	0.14025600	-1.77462400	4.11576600

48) TS-Anth[III-12]

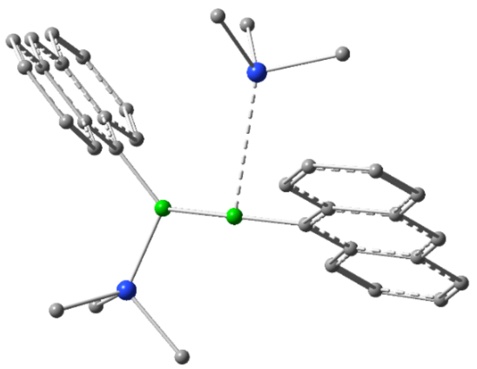

Note: Hydrogen atoms are omitted for clarity.

Number of imaginary frequencies = 1 (32.1 cm^{-1})

E_{total} = -2048.672049 a.u

$G_{correction}$ = 0.533963 a.u

Cartesian coordinates:

B	-0.79141500	-0.41041000	-0.62818900
B	0.67872200	-0.72099500	-0.94511100
C	-2.25965300	-0.10042800	-0.31152600
C	-3.14022400	-1.12715100	0.12381100
C	-2.77395000	1.21623100	-0.45622700
C	-2.70128200	-2.47134200	0.30982100
C	-4.52016700	-0.83785200	0.40534200
C	-4.13818600	1.50846200	-0.11682100
C	-1.96036700	2.29659300	-0.90451200
C	-3.56176800	-3.45948700	0.69784100
H	-1.64285600	-2.68250200	0.15032000
C	-5.39054500	-1.89378500	0.80540500
C	-4.97965600	0.47302100	0.29161600
C	-4.60943500	2.85055000	-0.20346200
H	-0.93113200	2.07755100	-1.18634500
C	-2.44392400	3.57412300	-0.97512900
C	-4.93107700	-3.17148000	0.93828500
H	-3.19543700	-4.47399100	0.83934500
H	-6.43439800	-1.65232000	1.00194500
H	-6.02177400	0.69461600	0.52547300
C	-3.78554200	3.86000500	-0.61107900
H	-5.64492100	3.05064700	0.06899600
H	-1.79822900	4.37924200	-1.31954800
H	-5.60463100	-3.96904500	1.24330500
H	-4.15462800	4.88143700	-0.66831200
C	2.06213400	-0.26504300	-0.31506900
C	2.72661400	-1.06269700	0.64897900
C	2.67052500	0.95887100	-0.69040400
C	2.15735000	-2.28078400	1.13065100
C	3.98725500	-0.65479500	1.21286800
C	3.92386400	1.37408400	-0.11508500
C	2.05791700	1.84380000	-1.62904100
C	2.78376200	-3.04769100	2.07178500
H	1.17785300	-2.55843800	0.74301200
C	4.61585100	-1.48341800	2.18905200
C	4.55744400	0.55309000	0.81603800
C	4.48246600	2.63184300	-0.48999900
H	1.11143000	1.52734400	-2.06524600
C	2.61716800	3.04550200	-1.96055900
C	4.03733200	-2.64692700	2.60595100
H	2.31799100	-3.96454700	2.42689900
H	5.57077000	-1.15738600	2.60018300
H	5.50819000	0.86630100	1.24938300
C	3.84898800	3.44895200	-1.38063300
H	5.42888400	2.92753500	-0.03827300
H	2.11978900	3.70012700	-2.67332600
H	4.52727000	-3.26513600	3.35519100
H	4.28472000	4.40857600	-1.65051100
P	-0.08212700	1.17833200	1.84506500
P	0.75778400	-1.86043300	-2.44657300
C	0.90488100	2.74355500	1.86951500
H	1.93501000	2.53154600	1.55596500
H	0.47968900	3.46363800	1.15985300
H	0.92632000	3.20052500	2.86853500
C	-1.60487700	1.76859000	2.71582500
H	-2.12955900	2.50127900	2.09091300
H	-2.28516200	0.92645400	2.89280900
H	-1.36758000	2.23741800	3.68047700
C	0.73909600	0.29430500	3.24783400
H	0.22587200	-0.65442300	3.44292400
H	1.77796400	0.06375200	2.98161000
H	0.73598000	0.89613100	4.16693400
C	1.73965600	-1.23424100	-3.85653400
H	1.29157700	-0.30855700	-4.23291500
H	2.75861500	-1.01308400	-3.51952900
H	1.78423300	-1.97056600	-4.66826900
C	-0.86888800	-2.23146200	-3.17799200
H	-1.50239100	-2.75771500	-2.45541400
H	-1.36858700	-1.30358500	-3.47456400
H	-0.74207800	-2.86680600	-4.06195600
C	1.48701700	-3.51872600	-2.18323400
H	2.49956900	-3.41698800	-1.77726200
H	0.87815800	-4.07341400	-1.46033500
H	1.53342700	-4.08268600	-3.12302400

49) 12-Anth

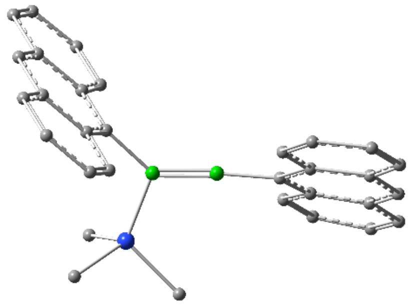

Note: Hydrogen atoms are omitted for clarity.

Number of imaginary frequencies = 0

E_{total} = -1587.687135 a.u

$G_{correction}$ = 0.427887 a.u

Cartesian coordinates:

B	-0.62687000	-0.42582700	0.44807200
B	0.81202300	-0.11092000	0.03833600
C	-2.05406900	-0.03705800	-0.13049400
C	-2.68897600	1.17014700	0.25048100
C	-2.74480300	-0.90265300	-1.01246500
C	-2.05821400	2.11245700	1.11962900
C	-4.00909800	1.49397500	-0.22502700
C	-4.06796700	-0.58146500	-1.48040300
C	-2.16273800	-2.12079800	-1.47849700
C	-2.67300800	3.27441200	1.49157500
H	-1.05339100	1.87685700	1.46926900
C	-4.62067800	2.71447100	0.18647800
C	-4.66948100	0.60695500	-1.07248500
C	-4.74010200	-1.48863600	-2.35130700
H	-1.14477700	-2.34343300	-1.16032300
C	-2.83430500	-2.96536900	-2.31612900
C	-3.97684500	3.58159700	1.02019000
H	-2.16240800	3.97351300	2.15057000
H	-5.61983800	2.93785500	-0.18615500
H	-5.67311000	0.84847500	-1.42485700
C	-4.14737400	-2.64898400	-2.75564300
H	-5.74250100	-1.22753000	-2.68909500
H	-2.36019300	-3.88210100	-2.66029100
H	-4.45649200	4.50955500	1.32390600
H	-4.67215300	-3.33045400	-3.42170900

P	-0.62511500	-1.44849900	2.03434100
C	0.99474700	-1.63775500	2.84115300
H	1.68904100	-2.19414200	2.20196700
H	1.42316400	-0.65275000	3.05238000
H	0.87030000	-2.18396900	3.78317700
C	-1.69259900	-0.75811400	3.34429800
H	-1.32535700	0.23035400	3.63885900
H	-2.71413300	-0.65016800	2.96375400
H	-1.70468600	-1.41720200	4.22078900
C	-1.24468500	-3.16242400	1.88878000
H	-2.25755600	-3.15214200	1.47128600
H	-0.59707900	-3.73302800	1.21398700
H	-1.26692800	-3.65386900	2.86915200
C	2.26243100	0.19135700	-0.26496900
C	3.23244200	-0.84837300	-0.30568800
C	2.68763300	1.53268900	-0.48023700
C	2.87515600	-2.22255300	-0.19636600
C	4.62502700	-0.53443600	-0.46338900
C	4.07312800	1.82669600	-0.70796200
C	1.76769300	2.61791600	-0.48737300
C	3.82104500	-3.21009300	-0.17771100
H	1.81261300	-2.46905100	-0.16222600
C	5.58560000	-1.58699400	-0.42444000
C	5.00876000	0.79097200	-0.66621400
C	4.46851600	3.17318700	-0.95213400
H	0.71403100	2.40624900	-0.30194700
C	2.18100400	3.89998800	-0.72298500
C	5.19982600	-2.88723400	-0.27120500
H	3.51777600	-4.25267800	-0.11298900
H	6.63824700	-1.32566700	-0.52477000
H	6.06554600	1.02336400	-0.80303800
C	3.54949800	4.18282100	-0.96685400
H	5.52364300	3.37552000	-1.13136700
H	1.45716200	4.71165000	-0.72054600
H	5.94265200	-3.68106600	-0.24497600
H	3.86202100	5.20687500	-1.15760100

50) TS-Anth[12-13]

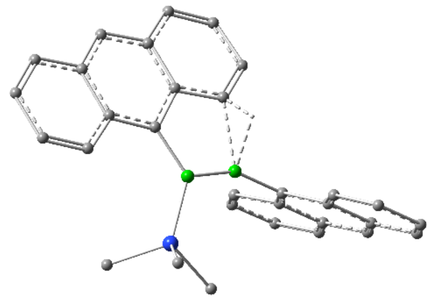

Note: Selected hydrogen atoms are omitted for clarity.

Number of imaginary frequencies = 1 (336.5 cm^{-1})

E_{total} = -1587.666038 a.u

$G_{correction}$ = 0.428328 a.u

Cartesian coordinates:

B	0.91021500	-0.78932300	0.03211200
B	-0.45891500	-0.06103100	-0.21222200
C	2.17080500	0.05488800	-0.35796100
C	3.56458800	-0.17503100	-0.24861000
C	1.78983600	1.33928700	-0.80916800
C	4.09894700	-1.45977700	0.04969800
C	4.50634700	0.90572900	-0.43600100
C	2.69178200	2.42372700	-0.95125100
C	0.39829600	1.56417100	-1.03450200
C	5.44184600	-1.67897800	0.18641000
H	3.40622000	-2.29511500	0.12791900
C	5.89832400	0.64333900	-0.26783600
C	4.05543200	2.19086100	-0.74722200
C	2.15341100	3.72236000	-1.20133600
H	-0.16067500	0.69926100	-1.52578300
C	-0.09086400	2.82299200	-1.28524500
C	6.35725400	-0.60552100	0.03662500
H	5.81458300	-2.67888300	0.39770700
H	6.59366100	1.47185000	-0.39883800
H	4.76756700	3.01397600	-0.80716100
C	0.80338000	3.92433800	-1.32195700
H	2.84303000	4.56225500	-1.27951000
H	-1.14695500	2.97023100	-1.50550200
H	7.42355400	-0.78518700	0.15394100
H	0.41386400	4.92363900	-1.50018500
P	0.79968400	-2.32876100	1.11198300
C	-0.89021600	-2.56596600	1.74261300
H	-1.19014200	-1.70082200	2.34249500
H	-1.59223700	-2.67535500	0.90964600
H	-0.92906300	-3.46796300	2.36416800
C	1.14661300	-3.95167200	0.33856100
H	0.46307400	-4.08123400	-0.50849200
H	2.17214900	-4.00551100	-0.04011000
H	0.98988100	-4.76612400	1.05631300
C	1.80887100	-2.34014900	2.63501300
H	2.87512500	-2.26981800	2.39781700
H	1.53563100	-1.46875800	3.23978600
H	1.62713800	-3.25372400	3.21364800
C	-2.01773900	0.04745700	-0.12548100
C	-2.62435400	0.87022200	0.85577200
C	-2.85824000	-0.67496800	-1.00983500
C	-1.84781300	1.60599700	1.80161200
C	-4.05529400	0.98843800	0.93773200
C	-4.29016600	-0.55980800	-0.92135100
C	-2.33199400	-1.54623700	-2.01206300
C	-2.43573500	2.40151000	2.74382400
H	-0.76375700	1.50625700	1.75523500
C	-4.63326600	1.83213100	1.93089700
C	-4.85102200	0.27279000	0.04515000
C	-5.11058900	-1.29791900	-1.82371000
H	-1.24841500	-1.64450700	-2.07669100
C	-3.14911700	-2.24186500	-2.85787700
C	-3.84941600	2.52236100	2.80863200
H	-1.81887200	2.94844100	3.45355700
H	-5.71915900	1.90924800	1.97146100
H	-5.93637200	0.36359300	0.10635400
C	-4.56044800	-2.11640900	-2.76641800
H	-6.19148100	-1.19221400	-1.73832900
H	-2.71779800	-2.89806700	-3.61083000
H	-4.30167400	3.16278600	3.56259400
H	-5.19738800	-2.67485200	-3.44869600

51) 13-Anth

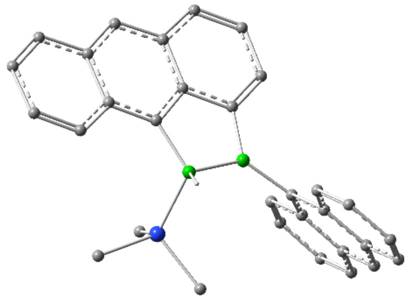

Note: Selected hydrogen atoms are omitted for clarity.

Number of imaginary frequencies = 0

E_{total} = -1587.757978 a.u

$G_{correction}$ = 0.430441 a.u

Cartesian coordinates:

B	0.81176600	-0.99343700	-0.04616900
B	-0.38231600	0.18430100	-0.50238900
C	2.16604500	-0.15774500	-0.32658400
C	3.52589200	-0.50308700	-0.22512800
C	1.85629000	1.13507900	-0.78678000
C	3.94702500	-1.81526200	0.14431500
C	4.54930600	0.47254200	-0.52610200
C	2.85243600	2.10187900	-1.12017600
C	0.45879400	1.42222000	-0.94359200
C	5.26908000	-2.14090700	0.25591300
H	3.17722800	-2.56693100	0.31117100
C	5.91788600	0.09567700	-0.38525300
C	4.19471300	1.75320200	-0.95876900
C	2.40447500	3.36054300	-1.61169100
H	0.71612500	-2.10127300	-0.55231200
C	0.08764300	2.65169800	-1.44546600
C	6.27022600	-1.16558400	-0.00073500
H	5.56327800	-3.15168500	0.53048800
H	6.68121100	0.84239400	-0.60200000
H	4.97826700	2.47562000	-1.19095800
C	1.06714100	3.62270200	-1.77500600
H	3.14737000	4.11470200	-1.87103300
H	-0.96683900	2.88941700	-1.58744200
H	7.31979300	-1.43445600	0.09689100
H	0.75273000	4.58892600	-2.16443500
P	0.51785600	-1.38721200	1.82085000
C	-1.12282000	-2.12701700	2.07959200
H	-1.90570100	-1.39748200	1.84498300
H	-1.23977500	-2.98239800	1.40436400
H	-1.24002100	-2.46197000	3.11630400
C	1.65041800	-2.57791600	2.61238400
H	1.64197700	-3.52000700	2.05281600
H	2.67116300	-2.18114800	2.61985200
H	1.33518000	-2.77374700	3.64419400
C	0.58566200	0.05620900	2.92603600
H	1.53855300	0.57823700	2.78096200
H	-0.23295400	0.74714500	2.69440200
H	0.50040000	-0.25140300	3.97438000
C	-1.94933300	0.12025100	-0.45058800
C	-2.69385900	0.95893200	0.41544700
C	-2.64597800	-0.86400000	-1.19340100
C	-2.07745900	1.98365500	1.19994800
C	-4.11466200	0.77966700	0.56917600
C	-4.06887300	-1.01924000	-1.06157400
C	-1.97082500	-1.72529200	-2.10989200
C	-2.79547900	2.76099800	2.06460300
H	-1.00967800	2.15878300	1.08003300
C	-4.83046100	1.61016500	1.48133300
C	-4.76660800	-0.20261900	-0.17280700
C	-4.73822200	-2.01005700	-1.83713800
H	-0.89391100	-1.61988000	-2.22620400
C	-2.64416300	-2.66576800	-2.83713500
C	-4.19465600	2.57047200	2.21204000
H	-2.29548200	3.53829900	2.63811300
H	-5.90426500	1.45492100	1.57969300
H	-5.84363300	-0.33185200	-0.05964000
C	-4.04944500	-2.81105600	-2.70081800
H	-5.81732500	-2.10825400	-1.72465400
H	-2.10271300	-3.30789200	-3.52804600
H	-4.75441400	3.19543900	2.90410300
H	-4.57278700	-3.56168800	-3.28889200

52) TS-Anth[13-8]

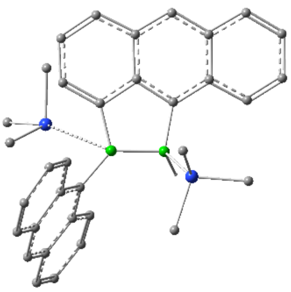

Note: Selected hydrogen atoms are omitted for clarity.

Number of imaginary frequencies = 1 (38.6 cm^{-1})

E_{total} = -2048.734751 a.u

$G_{correction}$ = 0.531295 a.u

Cartesian coordinates:

B	-1.01891100	0.81915700	-0.97442900
B	0.25283500	0.33855500	0.10528900
C	-2.30274500	0.22461000	-0.19566100
C	-3.67052900	0.19181900	-0.52156800
C	-1.90664400	-0.38190600	1.01030800
C	-4.16353100	0.69994000	-1.76027000
C	-4.62355700	-0.39265200	0.39462200
C	-2.82982000	-0.98618800	1.91698700
C	-0.49454700	-0.38831800	1.26969500
C	-5.49464700	0.67755500	-2.06647700
H	-3.43829700	1.08555700	-2.47500500
C	-6.00568800	-0.38550200	0.04181300
C	-4.18828400	-0.96010000	1.59534400
C	-2.29510500	-1.58866600	3.09101500
H	-0.88655200	0.53165000	-2.15379400
C	-0.03592100	-1.00385100	2.41640700
C	-6.43163300	0.13672400	-1.14509500

H	-5.84381000	1.06240500	-3.02225700
H	-6.71749000	-0.81800500	0.74435500
H	-4.91873100	-1.40203800	2.27423400
C	-0.94315700	-1.60001800	3.32958100
H	-2.98116900	-2.05401700	3.79856100
H	1.03211100	-1.03042600	2.63601900
H	-7.48975400	0.12873600	-1.39752500
H	-0.55999900	-2.07608800	4.22991100
P	-0.98881900	2.74779200	-1.04460200
C	0.58834100	3.34314900	-1.72438100
H	1.40611300	3.10798000	-1.03437100
H	0.78438300	2.82965500	-2.67269500
H	0.55839600	4.42522100	-1.89417300
C	-2.24239800	3.55799000	-2.09212400
H	-2.17104100	3.17016800	-3.11447200
H	-3.24683400	3.35308300	-1.70659800
H	-2.07941400	4.64219900	-2.10985200
C	-1.17707100	3.58341600	0.56043300
H	-2.09157600	3.22847900	1.04939500
H	-0.32130300	3.35625700	1.20615500
H	-1.23864400	4.66952800	0.42850300
C	1.80232000	0.57489300	0.03656000
C	2.47620600	1.36906900	0.99677300
C	2.55101700	0.05105200	-1.04585500
C	1.80148300	1.93957100	2.12104100
C	3.87744500	1.66705900	0.85060800
C	3.96105100	0.30674300	-1.15948400
C	1.94901100	-0.78207300	-2.03634900
C	2.44658700	2.74328000	3.01852800
H	0.74990200	1.70091200	2.27207100
C	4.51688700	2.50940800	1.80724500
C	4.58638700	1.12311600	-0.21810900
C	4.69253800	-0.28070100	-2.23235300
H	0.88393700	-0.99527900	-1.96570300
C	2.68142800	-1.33086100	-3.05143100
C	3.82629400	3.03802500	2.85823300
H	1.90426000	3.15361100	3.86741700
H	5.57728400	2.72108000	1.67453500
H	5.65183300	1.33475300	-0.31598500
C	4.07490000	-1.07983500	-3.15070500
H	5.76123000	-0.07921400	-2.29627800
H	2.19395800	-1.97157400	-3.78288400
H	4.32801900	3.67994000	3.57870600
H	4.64655500	-1.52670200	-3.96113300
P	0.86619300	-4.06147400	-0.57966700
C	1.59295400	-5.76064400	-0.74256500
H	2.46028100	-5.73064500	-1.41213200
H	1.91259300	-6.16781600	0.22656300
H	0.85549000	-6.44187600	-1.18242300
C	-0.32459200	-4.40560600	0.79640800
H	-0.81352800	-3.47551500	1.10787000
H	-1.10512500	-5.08929800	0.44258700
H	0.16819200	-4.85479900	1.67000000
C	2.23077000	-3.28123300	0.40006400
H	2.49513800	-3.88043800	1.28260100
H	3.12200300	-3.16570000	-0.22929300
H	1.92686500	-2.28070000	0.73139100

53) 8-Anth

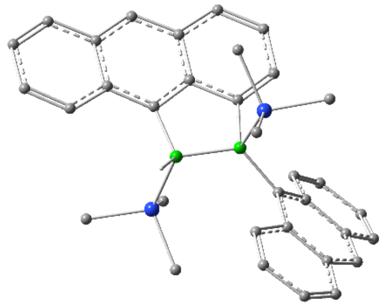

Note: Selected hydrogen atoms are omitted for clarity.

Number of imaginary frequencies = 0

E_{total} = -2048.751630 a.u

$G_{correction}$ = 0.539377 a.u

Cartesian coordinates:

B	-0.88286800	-1.05592100	-0.16117900
B	0.34522900	0.09715300	0.45505900
C	-2.22951900	-0.17705500	-0.04344500
C	-3.58537700	-0.55393900	-0.14127700
C	-1.93498400	1.17062800	0.23679300
C	-3.98389100	-1.91179600	-0.32143700
C	-4.62374500	0.44285700	-0.03299200
C	-2.95677200	2.16603000	0.36834800
C	-0.54920000	1.46904400	0.47341600
C	-5.29974200	-2.26822300	-0.42338500
H	-3.20311700	-2.66985800	-0.35455600
C	-5.98414300	0.03269400	-0.15669900
C	-4.28771800	1.77883400	0.20178300
C	-2.55714700	3.49244400	0.69831500
H	-0.95352100	-2.19216000	0.29844000
C	-0.23816600	2.76640100	0.82499400
C	-6.31514300	-1.27779900	-0.35014700
H	-5.57651800	-3.31244600	-0.55282500
H	-6.75947600	0.79510800	-0.08376100
H	-5.08259200	2.52153000	0.28747900
C	-1.23613300	3.77457500	0.91964800
H	-3.31827300	4.26724400	0.78696500
H	0.79702700	3.04876300	1.02286300
H	-7.35936400	-1.57071000	-0.43775400
H	-0.93904000	4.78822700	1.18533800
P	-0.58679100	-1.38683100	-2.06233100
C	0.95984300	-2.24650800	-2.49469100
H	1.82295600	-1.63693500	-2.20889600
H	1.01681600	-3.20309900	-1.96417000
H	0.99246100	-2.43430200	-3.57435400
C	-1.84969900	-2.42721700	-2.87442400
H	-1.87982100	-3.41202000	-2.39487900
H	-2.83834800	-1.96482700	-2.78517600
H	-1.60921400	-2.55769800	-3.93642200
C	-0.56276400	0.10779400	-3.10032400
H	-1.46349900	0.70339500	-2.91557800
H	0.31826900	0.71487200	-2.86665500
H	-0.52753600	-0.16834700	-4.16048600
C	1.91607100	0.08706600	-0.00830900

C	2.56996300	1.15492800	-0.69209600
C	2.73929400	-1.04067100	0.29733100
C	1.85828400	2.21294300	-1.34232000
C	4.00828800	1.19428300	-0.82686300
C	4.17303200	-1.00084400	0.16090200
C	2.18167200	-2.29357300	0.69733100
C	2.48787300	3.23211500	-2.00070400
H	0.77581100	2.19375600	-1.32329200
C	4.63568200	2.29776400	-1.47824700
C	4.78092000	0.14400000	-0.34441100
C	4.95431800	-2.13984600	0.51487900
H	1.09927400	-2.38172300	0.72599400
C	2.95482700	-3.38588000	0.98305800
C	3.90339900	3.29485700	-2.04998200
H	1.89617500	4.00089300	-2.49307200
H	5.72397100	2.30324200	-1.53137900
H	5.86765000	0.19128400	-0.42234600
C	4.36865200	-3.30404200	0.91811000
H	6.03744100	-2.06158500	0.42612700
H	2.48034400	-4.32608600	1.25821200
H	4.39552100	4.12090400	-2.55866800
H	4.97641900	-4.17142900	1.16625800
P	0.33042400	-0.14561000	2.47267500
C	0.60615200	-1.76025800	3.29480400
H	1.63975500	-2.09420100	3.17745400
H	0.38482500	-1.65205400	4.36342300
H	-0.06358000	-2.51738900	2.87241600
C	-1.25577100	0.33160300	3.24390300
H	-1.50500900	1.37304800	3.02287700
H	-2.06044700	-0.31143200	2.87104600
H	-1.17550400	0.20608900	4.32997300
C	1.55257300	0.92712800	3.30431600
H	1.46567400	0.84364800	4.39388400
H	2.56429800	0.63509500	3.00128300
H	1.38827400	1.96963800	3.01262200

54) TS-Anth[13-9]

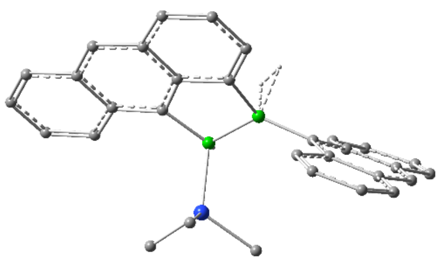

Note: Selected hydrogen atoms are omitted for clarity.

Number of imaginary frequencies = 1 (610.6 cm^{-1})

E_{total} = -1588.908774 a.u

$G_{correction}$ = 0.445978 a.u

Cartesian coordinates:

B	-0.82465200	-1.05061900	-0.18221200
B	0.37632900	0.12039400	-0.77782600
P	-0.48250700	-1.35146400	1.70659200
C	1.95704900	0.07919500	-0.51827100
C	2.75879100	-0.98920700	-1.01508600
C	2.60743300	1.07208400	0.26461800
C	4.18997900	-0.97807500	-0.85486200
C	4.03702400	1.05197600	0.45568500
C	1.89735600	2.11370000	0.94410000
C	4.98074200	-2.02751100	-1.40721000
C	4.79533300	0.04970800	-0.13758300
C	4.66610800	2.05397200	1.25243600
H	0.81897700	2.16473500	0.83823600
C	2.52913000	3.04624800	1.71800600
C	4.40600100	-3.08064800	-2.05432500
H	6.06158000	-1.96887200	-1.28446300
C	2.20898200	-2.13756300	-1.66847900
C	3.93929100	3.02655900	1.87119900
H	5.74911400	2.01059700	1.36198700
H	1.94418200	3.81516900	2.21793600
C	2.99425400	-3.14133500	-2.16356100
H	5.01869200	-3.87881000	-2.46683500
H	1.13148500	-2.25170300	-1.73316700
H	4.43033900	3.78009600	2.48257200
H	2.52879700	-4.00206500	-2.63880400
C	-2.18327400	-0.20938100	-0.39266300
C	-3.53302300	-0.59057600	-0.26598800
C	-1.90631500	1.11505200	-0.78093200
C	-4.58169100	0.38238100	-0.46200500
C	-2.93425600	2.08545100	-0.99965700
C	-0.52474000	1.42940200	-0.99840500
C	-5.93752100	-0.03034600	-0.30339500
C	-4.26144700	1.69805900	-0.80927700
C	-2.53658600	3.38839700	-1.41515900
H	0.08814800	-0.67589500	-2.08219100
C	-0.20625300	2.69413400	-1.43144100
C	-6.25444700	-1.32241100	0.00359000
H	-6.72103700	0.71391000	-0.44332400
C	-3.91657800	-1.93226500	0.02965800
C	-1.21508200	3.67692800	-1.62638800
H	-3.30359800	4.14548000	-1.57626900
H	0.83227900	2.96357400	-1.62624300
C	-5.22842600	-2.29199200	0.16175400
H	-7.29534600	-1.61823400	0.11569100
H	-3.12823900	-2.67751900	0.12246100
H	-0.92770800	4.67281500	-1.95909200
H	-5.49460000	-3.32451300	0.37780000
C	-0.53048300	0.13001600	2.75951100
H	-1.47205100	0.66539700	2.59284000
H	-0.46014400	-0.15063800	3.81661100
H	0.30394800	0.79687900	2.51712100
C	-1.65524800	-2.48383400	2.52250300
H	-2.66914500	-2.07189100	2.48233100
H	-1.64827000	-3.45249500	2.01058200
H	-1.36953100	-2.63318700	3.57054500
C	1.13355000	-2.12022000	2.03000700
H	1.94085700	-1.44326500	1.73083500
H	1.23648300	-2.35720000	3.09519000
H	1.22032200	-3.04277500	1.44525100
H	0.48695800	-0.08335700	-2.42912300
H	-0.81157900	-2.19531600	-0.61331900
H	-5.06470400	2.42129500	-0.95739800
H	5.87910900	0.05350700	-0.01672500

55) 9-Anth

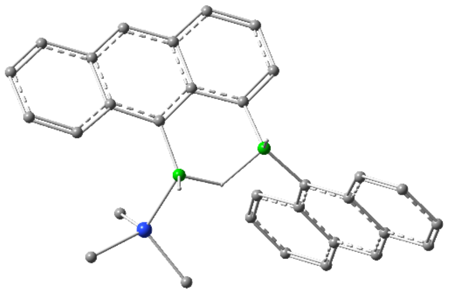

Note: Selected hydrogen atoms are omitted for clarity.

Number of imaginary frequencies = 0

E_{total} = -1588.955220 a.u

$G_{correction}$ = 0.449536 a.u

Cartesian coordinates:

B	1.05003500	-0.54678600	-1.19575200
P	1.19234000	-2.40421200	-0.52494100
C	2.25981800	-3.50665100	-1.50395300
H	3.31077800	-3.22255800	-1.39136100
H	2.13198700	-4.54112600	-1.16469400
H	1.98419100	-3.44043200	-2.56224300
C	-0.43463200	-3.20984400	-0.57657000
H	-0.34359700	-4.27907900	-0.35577800
H	-1.10436500	-2.74634800	0.15659700
H	-0.86723700	-3.08046500	-1.57500500
C	1.79897300	-2.57202800	1.17743500
H	1.76811600	-3.62057000	1.49405500
H	2.82977800	-2.20655300	1.23549700
H	1.17862400	-1.97407200	1.85264700
B	-0.75343400	0.97641500	-0.62155000
H	1.01312200	-0.66595300	-2.40005000
H	-0.60910600	1.38870300	-1.74216300
C	2.09375700	0.45218200	-0.52425300
C	3.49080100	0.32788700	-0.69146000
C	1.60940300	1.45964900	0.33534000
C	4.05978300	-0.57163300	-1.64130600
C	4.39621300	1.12630100	0.09141500
C	2.51041200	2.26336500	1.11507500
C	0.19440100	1.70062600	0.41480100
C	5.41320700	-0.71386900	-1.77965200
H	3.38843300	-1.11713600	-2.30232400
C	5.79999700	0.94190400	-0.06972900
C	3.88315000	2.05940000	0.99371600
C	1.96925200	3.24183900	2.00009100
H	-0.12885700	-0.36892800	-0.78416700
C	-0.26667000	2.65618700	1.28638300
C	6.29850900	0.04368000	-0.96962900
H	5.81690800	-1.39477700	-2.52616300
H	6.46997400	1.54631400	0.54098400
H	4.57032500	2.65041100	1.60083600
C	0.62006100	3.42642900	2.08653100
H	2.65754000	3.83794200	2.59826100
H	-1.33848200	2.84381100	1.35848900
H	7.37310100	-0.08060500	-1.08310500
H	0.21426000	4.17465300	2.76486800

C	-2.21491100	0.46755800	-0.23053600
C	-2.42829800	-0.33803300	0.91718600
C	-3.33810000	0.78588900	-1.03805200
C	-1.37082200	-0.68673700	1.81580400
C	-3.73106400	-0.86824500	1.22170800
C	-4.64689400	0.27556700	-0.71220100
C	-3.25170400	1.62954700	-2.18993800
C	-1.57718200	-1.49367100	2.90060300
H	-0.38008800	-0.27435800	1.63962400
C	-3.90832200	-1.71483100	2.35492400
C	-4.80652900	-0.54831300	0.39791400
C	-5.76670400	0.61085100	-1.52850300
H	-2.28548900	2.04156900	-2.46160200
C	-4.34683500	1.93429800	-2.94711500
C	-2.86364200	-2.02615300	3.17438400
H	-0.74959700	-1.72272300	3.56976700
H	-4.90662500	-2.10475900	2.55035000
H	-5.79533400	-0.94584200	0.63062200
C	-5.62696100	1.41826300	-2.61755300
H	-6.73974500	0.20602100	-1.25268300
H	-4.23803900	2.58246600	-3.81394200
H	-3.01152700	-2.66980400	4.03839700
H	-6.48797100	1.67075500	-3.23224400

56) TS-Anth[9-14]

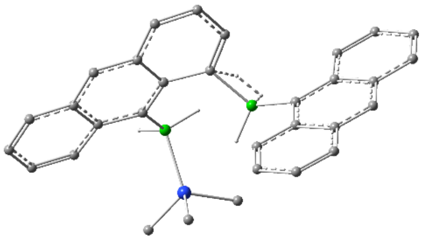

Note: Selected hydrogen atoms are omitted for clarity.

Number of imaginary frequencies = 1 (1338.5 cm^{-1})

E_{total} = -1590.073543 a.u

$G_{correction}$ = 0.464106 a.u

Cartesian coordinates:

B	-0.92687500	0.51744600	-0.61178300
H	-0.20619600	1.13880700	0.10606700
C	2.36098700	-0.00731600	-0.85819500
C	3.68276700	-0.38820500	-0.48808800
C	1.38890500	-1.03476400	-0.98484300
C	4.72978200	0.56979000	-0.31682900
C	4.04351300	-1.76135800	-0.26775300
C	1.80446200	-2.42011400	-0.91054500
C	-0.03488900	-0.82567800	-1.20449700
C	5.98413700	0.21748900	0.09857000
H	4.52491200	1.60920500	-0.55069600
C	5.35942700	-2.10065300	0.16038200
C	3.10022500	-2.74829000	-0.52747800
C	0.91010500	-3.47336500	-1.26055600
C	-0.83617800	-1.88144400	-1.57846400
C	6.30684300	-1.13907800	0.35516900

H	6.74854700	0.98275400	0.21766100
H	5.58826600	-3.15307600	0.32467400
H	3.38266800	-3.79870600	-0.44900000
C	-0.36813600	-3.21334600	-1.64668500
H	1.28930100	-4.49394600	-1.22388200
H	-1.89485100	-1.69900100	-1.75870300
H	7.30799200	-1.40695800	0.68500900
H	-1.04250400	-4.01339300	-1.94108100
C	-2.38230600	0.16507400	-0.07294600
C	-3.58145900	0.38875600	-0.78782700
C	-2.46887500	-0.34470200	1.24476200
C	-3.60516700	0.83466900	-2.14705100
C	-4.85971900	0.15124900	-0.16325300
C	-3.74488100	-0.57495100	1.86600500
C	-1.30488800	-0.66781200	2.01036700
C	-4.77439700	1.04287100	-2.82186500
H	-2.66713800	1.00140600	-2.67031200
C	-6.05941800	0.39013400	-0.89556400
C	-4.90947600	-0.31077400	1.14894400
C	-3.80007400	-1.07061400	3.20120600
H	-0.32360200	-0.54625700	1.55599200
C	-1.39463600	-1.14945800	3.28591200
C	-6.02501800	0.82441700	-2.18739200
H	-4.74888100	1.37689100	-3.85669400
H	-7.00882800	0.20876300	-0.39304300
H	-5.87870000	-0.47936500	1.61987900
C	-2.66022100	-1.34803300	3.89706700
H	-4.78048900	-1.22720200	3.64965500
H	-0.48903000	-1.39286500	3.83721500
H	-6.94777300	0.99921100	-2.73570800
H	-2.71499200	-1.72892800	4.91435700
H	-1.01685300	1.33385300	-1.60184700
H	-0.52384100	0.44988400	-1.83309500
B	2.09132300	1.53009300	-1.27790100
H	1.07820500	1.72227100	-1.90822300
H	3.01981200	2.01130300	-1.90135900
P	1.94121000	2.78415900	0.25179400
C	0.60355700	3.99467100	0.01181900
H	0.76878100	4.51919900	-0.93590800
H	-0.36217400	3.48288600	-0.03814600
H	0.58421400	4.72482400	0.82872500
C	1.68465500	2.08879400	1.91600000
H	1.73281700	2.88497300	2.66792300
H	0.71151600	1.59228300	1.98466400
H	2.46838300	1.35210000	2.12561500
C	3.40599500	3.84916600	0.48372600
H	4.24382600	3.26517200	0.87860400
H	3.70197800	4.28418700	-0.47697600
H	3.18051400	4.65690100	1.18955200

57) 14-Anth

14-Anth comprises two components: anthracen-9-ylborane (**14-Anth**) and its PMe_3-coordinated version (**10-Anth**). Structures and energies of these compounds are given seperately (see below).

58) 14-Anth

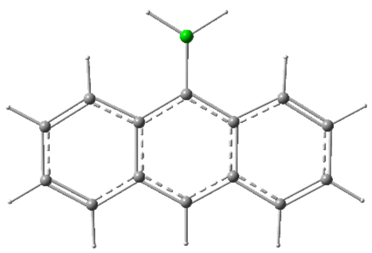

Number of imaginary frequencies = 0

E_{total} = -564.550720 a.u

$G_{correction}$ = 0.168161 a.u

Cartesian coordinates:

C	0.00000000	1.29388900	0.00000000
C	1.22895000	0.56226400	0.00000000
C	-1.22895100	0.56226500	0.00000100
C	2.51161500	1.18514700	0.00000000
C	1.21929800	-0.87535600	0.00000100
C	-1.21929800	-0.87535600	0.00000100
C	-2.51161500	1.18514600	0.00000000
C	3.66765000	0.45409300	-0.00000100
H	2.57190400	2.26815900	0.00000200
C	2.44092300	-1.60793200	0.00000100
C	0.00000000	-1.54737800	0.00000100
C	-2.44092300	-1.60793200	0.00000000
C	-3.66765100	0.45409300	-0.00000100
C	3.64161900	-0.96289900	-0.00000100
H	4.62527800	0.96997100	-0.00000200
H	2.38708400	-2.69562000	0.00000200
H	0.00000000	-2.63847700	0.00000200
C	-3.64161900	-0.96289900	-0.00000200
H	-2.38708500	-2.69562000	0.00000000
H	-4.62527900	0.96997000	-0.00000100
H	4.57302300	-1.52344500	-0.00000100
H	-4.57302300	-1.52344600	-0.00000400
H	-2.57190400	2.26815900	0.00000100
B	0.00000000	2.82654900	0.00000000
H	-1.00961400	3.46237400	0.00002200
H	1.00961300	3.46237400	-0.00002200

59) 10-Anth

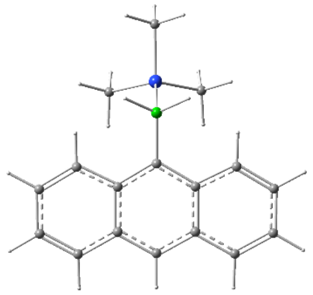

Number of imaginary frequencies = 0

E_{total} = -1025.569801 a.u

$G_{correction}$ = 0.273773 a.u

Cartesian coordinates:

C	-0.09587500	-0.00080700	-0.73122000
C	-0.78138100	-1.20963200	-0.43902000
C	-0.76357200	1.21797000	-0.43937700
C	-0.20752800	-2.49621800	-0.68904200
C	-2.10291500	-1.19759600	0.13655000
C	-2.08495600	1.22566900	0.13645700
C	-0.17078400	2.49579800	-0.69030500
C	-0.86465500	-3.65699900	-0.39273400
H	0.77745200	-2.54200600	-1.14378000
C	-2.76053300	-2.42809200	0.43129700
C	-2.72105900	0.01872600	0.41145400
C	-2.72402000	2.46590700	0.43125400
C	-0.81048400	3.66630000	-0.39414900
C	-2.16262900	-3.62741100	0.17987500
H	-0.39396700	-4.61471800	-0.60517800
H	-3.75888300	-2.38212500	0.86533600
H	-3.71838500	0.02612900	0.85319600
C	-2.10844600	3.65610500	0.17919200
H	-3.72272600	2.43499400	0.86579800
H	-0.32579400	4.61688500	-0.60713100
H	-2.67574500	-4.55872000	0.40940300
H	-2.60753300	4.59499800	0.40876400
H	0.81468700	2.52640700	-1.14531400
B	1.40641200	-0.01203300	-1.31750200
H	1.74399000	0.97097000	-1.94804800
H	1.73072600	-1.00310700	-1.94218000
P	2.68169700	-0.01713800	0.15294900
C	2.61700500	1.43281700	1.24911600
H	2.79036200	2.34454000	0.66750100
H	1.62794700	1.50181300	1.71461300
H	3.37817300	1.35970800	2.03458100
C	2.56499000	-1.43769000	1.28268300
H	3.32825800	-1.37430600	2.06688500
H	1.57381900	-1.45915800	1.74834500
H	2.70408500	-2.36871000	0.72282600
C	4.38862200	-0.05364700	-0.46896500
H	4.53111200	-0.95188600	-1.07936000
H	4.55992200	0.82264300	-1.10341700
H	5.11431100	-0.05433200	0.35204700

60) TS-Anth[9-15]

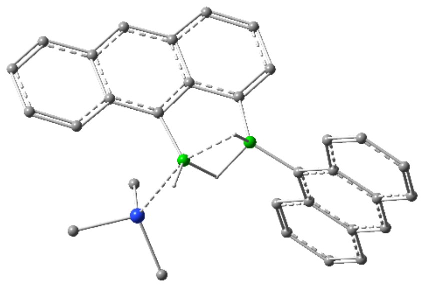

Note: Selected hydrogen atoms are omitted for clarity.

Number of imaginary frequencies = 1 (322.2 cm^{-1})

E_{total} = -1588.931311 a.u

$G_{correction}$ = 0.447946 a.u

Cartesian coordinates:

B	-0.80355000	-0.11938200	-1.23350000
P	-1.61831200	2.37843900	-0.87386800
C	-3.11240300	3.00036300	-1.75181900
H	-3.96484200	2.34521700	-1.54101400
H	-3.35980300	4.02170800	-1.43384600
H	-2.93853500	2.99825900	-2.83357300
C	-0.46696300	3.79775600	-1.09406700
H	-0.92061000	4.73849200	-0.75572700
H	0.45510200	3.62111000	-0.52755800
H	-0.19996100	3.89364000	-2.15210200
C	-2.13778600	2.59386900	0.87791900
H	-2.49341500	3.61544900	1.06488400
H	-2.94510500	1.89006600	1.11214700
H	-1.30155900	2.38359900	1.55569700
B	0.70958700	-0.74072100	-0.18678300
H	-0.73310600	-0.00954700	-2.41405400
H	0.33635000	-1.43756200	-1.13634300
C	-1.94763700	-0.72033300	-0.34722600
C	-3.32254900	-0.80253300	-0.64677300
C	-1.49878200	-1.17396600	0.90336700
C	-3.85617600	-0.41109100	-1.90795700
C	-4.23630800	-1.29102500	0.35471700
C	-2.39403500	-1.65083700	1.90690100
C	-0.08491700	-1.15781100	1.11641400
C	-5.19646100	-0.49447800	-2.16933000
H	-3.16742100	-0.05077600	-2.67110300
C	-5.62495800	-1.35770000	0.04375000
C	-3.75569800	-1.68718500	1.60948100
C	-1.83596800	-2.06333700	3.15324700
H	0.14600300	0.45391100	-0.58821000
C	0.39923300	-1.56736000	2.33173500
C	-6.09437800	-0.97234600	-1.17964900
H	-5.58076000	-0.19789700	-3.14281700
H	-6.30641100	-1.72871700	0.80835600
H	-4.46419200	-2.04301300	2.35889900
C	-0.48413900	-2.01578700	3.35560400
H	-2.50472100	-2.42063800	3.93543000
H	1.47302000	-1.56599400	2.52083800
H	-7.15715700	-1.03259500	-1.40241300
H	-0.07029200	-2.33469600	4.31014900
C	2.25472100	-0.36695600	-0.19473500
C	2.69824100	0.82716800	0.41898100
C	3.21749600	-1.21835000	-0.78352800
C	1.80092100	1.71460500	1.08965100
C	4.08693900	1.20111800	0.39220500
C	4.60997900	-0.84866000	-0.79876900
C	2.87442400	-2.47721100	-1.36693300
C	2.22938000	2.87792700	1.66524100
H	0.75089300	1.43655900	1.16917800
C	4.49809900	2.42512000	0.99631800
C	5.00690400	0.35616700	-0.22368700
C	5.56402400	-1.72081200	-1.40010200
H	1.83502700	-2.79498800	-1.35199300
C	3.81675000	-3.29219600	-1.92586800
C	3.59959100	3.24590400	1.61289200
H	1.51664700	3.52449300	2.17459600
H	5.55473500	2.68689800	0.95521500
H	6.06033000	0.63789000	-0.24650200

C	5.18359400	-2.90933300	-1.94944000
H	6.60897200	-1.41303400	-1.40287800
H	3.52011000	-4.24587300	-2.35665800
H	3.92713000	4.17586200	2.07164700
H	5.92123500	-3.56803600	-2.40206600

61) 15-Anth

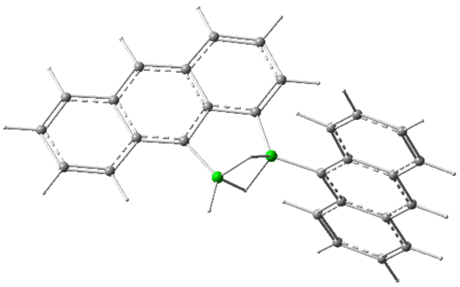

Number of imaginary frequencies = 0

E_{total} = -1127.961798 a.u

$G_{correction}$ = 0.343419 a.u

Cartesian coordinates:

B	0.30064200	-0.00225800	-0.21190600
H	-0.18795300	-0.96749500	-1.02291400
C	-2.28905800	-0.00399500	-0.56240600
C	-3.64684100	-0.00573300	-0.92862000
C	-1.95792100	0.00348900	0.79939600
C	-4.07868400	-0.01312600	-2.28531700
C	-4.65354600	0.00027600	0.10638000
C	-2.93513700	0.00940600	1.83287600
C	-0.56227200	0.00501100	1.09553200
C	-5.40722800	-0.01448800	-2.60486800
H	-3.32198100	-0.01768500	-3.06758900
C	-6.02754600	-0.00139300	-0.27202500
C	-4.27889300	0.00763500	1.45566300
C	-2.46899500	0.01682100	3.17924700
C	-0.16217600	0.01232500	2.40858500
C	-6.39624900	-0.00852200	-1.58616000
H	-5.71546100	-0.02015600	-3.64794300
H	-6.77886100	0.00319500	0.51655200
H	-5.05723100	0.01207700	2.21987000
C	-1.12604300	0.01821100	3.45451900
H	-3.19843600	0.02139800	3.98834500
H	0.89908700	0.01371600	2.65870500
H	-7.44865000	-0.00968400	-1.85993200
H	-0.78787000	0.02392000	4.48821000
B	-0.99299900	-0.00891700	-1.44754100
H	-0.18802400	0.95412200	-1.03374200
H	-0.91218500	-0.01568400	-2.63535800
C	1.87427600	-0.00195900	-0.28497700
C	2.59086100	-1.21812200	-0.28820400
C	2.58925100	1.21511200	-0.29676600
C	1.94387500	-2.48795600	-0.18671000
C	4.02683900	-1.21564400	-0.38146900
C	4.02522200	1.21393100	-0.39012300
C	1.94053200	2.48475900	-0.20426000
C	2.65179600	-3.65584400	-0.19773400
H	0.86166800	-2.52627200	-0.07187600
C	4.73054900	-2.45475800	-0.40170800
C	4.70499300	-0.00061400	-0.44444800
C	4.72723900	2.45381900	-0.41929800
H	0.85828800	2.52244300	-0.08953300
C	2.64686400	3.65349800	-0.22370400
C	4.06659700	-3.64288500	-0.31523000
H	2.13002100	-4.60605400	-0.11089400
H	5.81646000	-2.42654100	-0.48158100
H	5.79275400	-0.00018600	-0.52288400
C	4.06167000	3.64162900	-0.34128100
H	5.81317800	2.42651000	-0.49909500
H	2.12379900	4.60359000	-0.14360600
H	4.61476700	-4.58189500	-0.32800700
H	4.60856600	4.58126500	-0.36084600

62) TS-Anth[12-17]

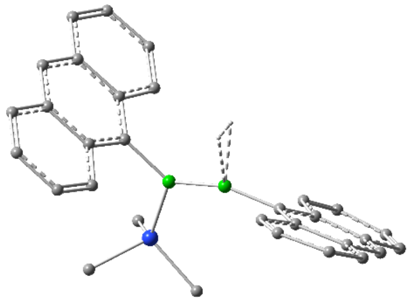

Note: Selected hydrogen atoms are omitted for clarity.

Number of imaginary frequencies = 1 (844.5 cm^{-1})

E_{total} = -1588.851964 a.u

$G_{correction}$ = 0.440471 a.u

Cartesian coordinates:

B	-0.64596800	-0.21013200	0.37289600
P	-0.53639800	-0.93803200	2.12468700
C	-1.51596500	-0.01965000	3.35976400
H	-2.56152700	0.02729500	3.03654600
H	-1.46440900	-0.51342000	4.33736200
H	-1.13450100	1.00251100	3.45159500
C	1.13303100	-1.00639200	2.83929700
H	1.76964000	-1.68411400	2.26013600
H	1.58299300	-0.00848700	2.83216700
H	1.07508400	-1.36900200	3.87200200
C	-1.16592600	-2.64313500	2.30809100
H	-0.55821400	-3.33034800	1.70931600
H	-1.13145400	-2.95722600	3.35831300
H	-2.20115400	-2.69381300	1.95346600
B	0.72738100	0.09233700	-0.26929400
H	0.14187200	1.12837200	-1.61038400
H	0.19198700	0.41306600	-1.91584000
C	-2.12667200	0.03094000	-0.15398900
C	-2.78860100	1.26635600	0.05578500
C	-2.83300300	-0.98989500	-0.83663000
C	-2.14800000	2.36282400	0.71118200
C	-4.14452100	1.46588200	-0.38592600
C	-4.19185000	-0.79265100	-1.26989800
C	-2.23160300	-2.25186500	-1.13146800

C	-2.78680000	3.55282600	0.91829200
H	-1.11639700	2.22245000	1.03163800
C	-4.77952200	2.72110400	-0.15228700
C	-4.81634700	0.42903200	-1.02958700
C	-4.87524300	-1.85175500	-1.93661200
H	-1.19001400	-2.39144700	-0.84591700
C	-2.91468900	-3.24440600	-1.77517900
C	-4.12517100	3.73687800	0.48123500
H	-2.26835200	4.36934800	1.41656900
H	-5.80513300	2.84847400	-0.49726700
H	-5.84662300	0.57824600	-1.35526800
C	-4.26113400	-3.04551300	-2.17993200
H	-5.90414500	-1.67982500	-2.25107900
H	-2.42429400	-4.19127300	-1.99106000
H	-4.62250500	4.68945500	0.65051000
H	-4.79476500	-3.84363500	-2.69139000
C	2.26514000	0.18057000	-0.41070500
C	3.06028300	-0.99610500	-0.35589200
C	2.92343800	1.42969600	-0.57104600
C	2.48655000	-2.29774900	-0.23822700
C	4.49482300	-0.91636700	-0.42626100
C	4.35233400	1.49384100	-0.71261300
C	2.21124800	2.66452200	-0.58800500
C	3.25991200	-3.42026600	-0.13610400
H	1.39931100	-2.37000000	-0.25270000
C	5.27098200	-2.10739100	-0.31328100
C	5.10269300	0.32200500	-0.62023300
C	4.98184000	2.75468400	-0.92205700
H	1.13598200	2.64336100	-0.41471400
C	2.84653400	3.85922800	-0.78568500
C	4.67614900	-3.32519100	-0.15878500
H	2.78979000	-4.39833900	-0.05812600
H	6.35578400	-2.01784800	-0.35520000
H	6.18914500	0.37647200	-0.69982800
C	4.25169600	3.90691000	-0.97050800
H	6.06443500	2.77424300	-1.04141600
H	2.27336700	4.78364500	-0.78824100
H	5.27981100	-4.22590200	-0.07388000
H	4.74355500	4.86338400	-1.13201900

63) 17-Anth

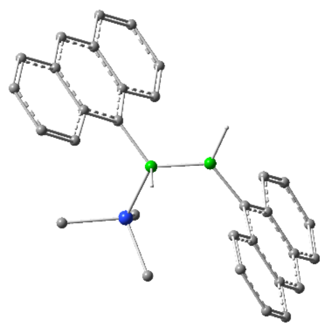

Note: Selected hydrogen atoms are omitted for clarity.

Number of imaginary frequencies = 0

E_{total} = -1588.933453 a.u

$G_{correction}$ = 0.444278 a.u

Cartesian coordinates:

B	0.57639600	-0.44675900	-0.01254100
P	0.05459900	-0.23934800	1.84763500
C	1.30253500	-0.91469300	2.98742200
H	2.25556300	-0.39549100	2.83868100
H	0.98401700	-0.79128100	4.02887100
H	1.45500000	-1.97973300	2.78157400
C	-1.48003100	-1.10445500	2.29997400
H	-2.33785200	-0.63387900	1.80858200
H	-1.42792500	-2.15160100	1.98293400
H	-1.62300900	-1.06388100	3.38608500
C	-0.20331200	1.45825700	2.45542200
H	-0.99416800	1.94995100	1.87594800
H	-0.49626100	1.43664200	3.51171200
H	0.71990000	2.03894400	2.35390800
B	-0.58531700	-0.07087500	-1.16182900
H	0.27922500	-1.63224100	-0.18491100
H	-0.21946200	0.02641300	-2.31028400
C	2.14336800	-0.13116300	-0.22059800
C	3.13051700	-1.15118200	-0.25410300
C	2.58433100	1.20703800	-0.37997400
C	2.82027200	-2.53414100	-0.06169100
C	4.51979900	-0.83183600	-0.47718700
C	3.97105800	1.52458800	-0.59999100
C	1.67765500	2.30909900	-0.32874800
C	3.77808600	-3.50807300	-0.09021500
H	1.78453200	-2.81292500	0.11047200
C	5.49146500	-1.87488200	-0.51080900
C	4.90558400	0.49489800	-0.64923400
C	4.36684800	2.88511100	-0.76091900
H	0.62753100	2.09642600	-0.15202200
C	2.08675900	3.60366600	-0.48172100
C	5.13814900	-3.17806000	-0.32246800
H	3.49705500	-4.54797800	0.06258400
H	6.53029500	-1.59865600	-0.68866800
H	5.95706200	0.73006000	-0.81879500
C	3.45687700	3.90000800	-0.70471800
H	5.42285800	3.09320100	-0.93012400
H	1.35854700	4.41156800	-0.43702200
H	5.89039200	-3.96327600	-0.34863600
H	3.77470100	4.93263800	-0.82987700
C	-2.13767100	-0.07647200	-0.89933700
C	-2.80485700	1.13587900	-0.61828300
C	-2.88297300	-1.27582400	-0.86746900
C	-2.13768900	2.39335000	-0.73301800
C	-4.18265400	1.14413400	-0.20912100
C	-4.26459500	-1.26845600	-0.46420900
C	-2.30240400	-2.53119000	-1.22415100
C	-2.76516400	3.56931800	-0.43072300
H	-1.11576400	2.41258300	-1.11308800
C	-4.80226400	2.38927500	0.10500800
C	-4.87682400	-0.06236700	-0.12386400
C	-4.97586500	-2.50304800	-0.40802800
H	-1.26886300	-2.55334400	-1.56713400
C	-3.01881700	-3.69312700	-1.16841100
C	-4.11663400	3.56555700	0.00608400
H	-2.23671000	4.51377600	-0.53951800
H	-5.84474900	2.37843900	0.42097700
H	-5.91837200	-0.06156500	0.19898900
C	-4.37426600	-3.68046900	-0.74501400
H	-6.01829300	-2.48078700	-0.09241700

H	-2.55490700	-4.63344500	-1.45708300
H	-4.60622600	4.50651700	0.24655500
H	-4.93220100	-4.61312100	-0.70038000

64) TS-Anth[17-9]

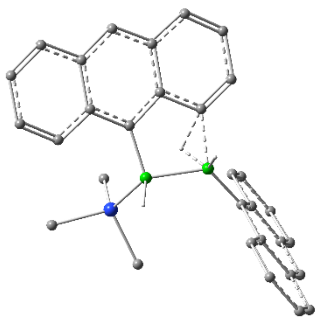

Note: Selected hydrogen atoms are omitted for clarity.

Number of imaginary frequencies = 1 (1117.5 cm^{-1})

E_{total} = -1588.883671 a.u

$G_{correction}$ = 0.446796 a.u

Cartesian coordinates:

B	0.82099600	-0.96130600	0.11809200
P	0.51144000	-1.01905900	2.03511400
C	1.60631800	-2.14096700	2.96327500
H	2.64636700	-1.80796800	2.87751800
H	1.32325300	-2.16359800	4.02224800
H	1.52511900	-3.15332500	2.55206400
C	-1.16237000	-1.62532600	2.39320900
H	-1.29634100	-1.79470600	3.46744200
H	-1.90391000	-0.89810800	2.04374800
H	-1.32480300	-2.56437000	1.85152200
C	0.66654300	0.56529600	2.91528400
H	0.52693300	0.42193400	3.99271600
H	1.66204500	0.98753000	2.73616500
H	-0.08986700	1.27301000	2.55735100
B	-0.43660200	0.03309900	-0.83395400
H	0.70532000	-2.13319800	-0.18806600
H	0.00060400	-0.39944000	-1.85498200
C	2.17865600	-0.20052000	-0.29661000
C	3.51294300	-0.64626900	-0.21131500
C	1.96021600	1.08019800	-0.84711900
C	3.84115600	-1.94897200	0.26597900
C	4.59544000	0.21308300	-0.62483000
C	3.01740400	1.94735000	-1.25777300
C	0.60071800	1.50331200	-0.99608800
C	5.13711600	-2.37320300	0.35863000
H	3.02151600	-2.61378200	0.53409600
C	5.93347600	-0.26392300	-0.50558600
C	4.32790400	1.49136700	-1.12591400
C	2.66360500	3.23857000	-1.75208000
H	0.18247600	0.74026100	0.01304900
C	0.29526300	2.76220400	-1.43716800
C	6.19999000	-1.51407000	-0.02558900
H	5.36053300	-3.37539900	0.71774200
H	6.74350600	0.39583000	-0.81459600
H	5.15850500	2.13420800	-1.41945700
C	1.35417200	3.62861300	-1.83076300
H	3.46005700	3.91530600	-2.05859700
H	-0.73791000	3.09061000	-1.53098700
H	7.22766700	-1.86139900	0.05467400
H	1.10822100	4.61827200	-2.21023900
C	-2.00800500	0.04411100	-0.60307600
C	-2.71434400	0.99280200	0.18257700
C	-2.75256400	-1.03651100	-1.15287600
C	-2.06933600	2.08377700	0.84750200
C	-4.13805400	0.87992300	0.38390100
C	-4.17496100	-1.13861500	-0.95409500
C	-2.13507000	-2.08764600	-1.90121100
C	-2.75120400	2.97501100	1.62914500
H	-1.00131800	2.23046600	0.71898600
C	-4.82049700	1.83635500	1.19222500
C	-4.83494400	-0.17456900	-0.19722500
C	-4.89500700	-2.23126400	-1.51984800
H	-1.06087400	-2.06042200	-2.05516300
C	-2.85385200	-3.12710900	-2.42094100
C	-4.15343400	2.85575400	1.80433800
H	-2.21476100	3.78860300	2.11288900
H	-5.89724000	1.72270600	1.31389600
H	-5.91182000	-0.25505700	-0.04463400
C	-4.25808600	-3.20141600	-2.23539200
H	-5.97170800	-2.27132600	-1.35817200
H	-2.34486100	-3.90656000	-2.98375300
H	-4.68732400	3.57436400	2.42188200
H	-4.81812500	-4.03158900	-2.66013700

Mechanistic pathways for the hydrogenation of diborene II

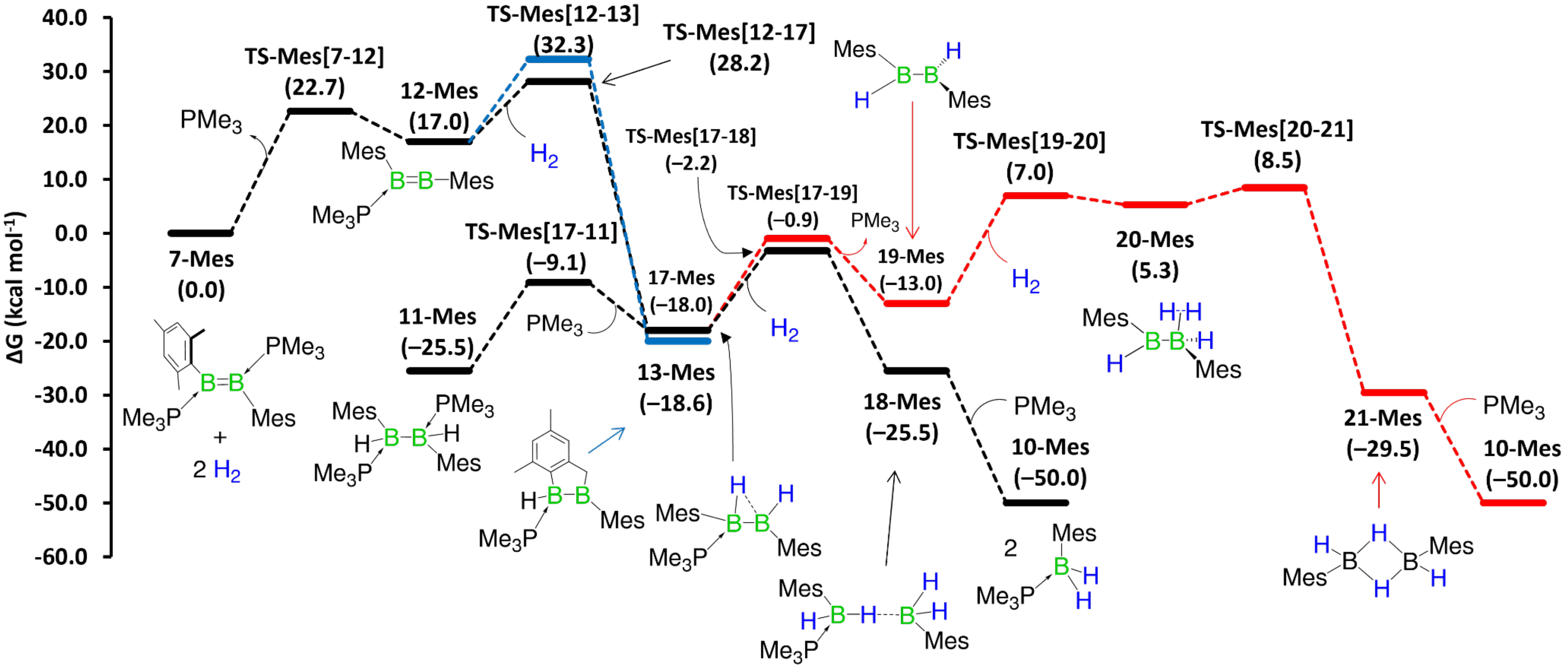

Figure S82. Computed mechanism for the formation of the borane **10-Mes** from the hydrogenation of diborene **II**.

Structures, energies and cartesian coordinates involving diborene II in the hydrogenation:

65) Diborene II

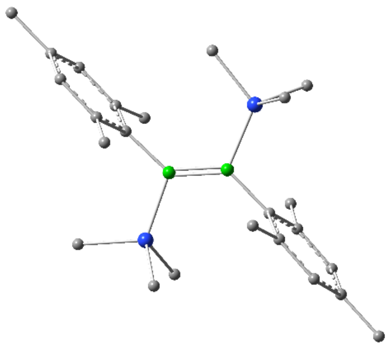

Note: Hydrogen atoms are omitted for clarity.

Number of imaginary frequencies = 0

E_{total} = -1670.310847 a.u

$G_{correction}$ = 0.512063 a.u

Cartesian coordinates:

C	-2.87792600	0.21629900	1.20735400
C	-4.24929800	-0.04692000	1.19361900
C	-2.14405200	0.32904100	-0.00067100
C	-4.96051200	-0.19220000	0.00553600
H	-4.77926300	-0.14507400	2.14423300
C	-2.87806500	0.19025600	-1.20489400
C	-4.25024600	-0.07230900	-1.18504300
H	-4.78017100	-0.19070100	-2.13331800
B	-0.55683500	0.55153300	-0.00127300
B	0.62793600	-0.48957200	0.01173300
C	2.21479900	-0.33864600	0.01252500
C	2.94494800	-0.21232000	1.21494500
C	2.95187300	-0.28587700	-1.19360300
C	4.32709000	-0.00724900	1.19739300
C	4.33190400	-0.08050200	-1.18288700
C	5.04295100	0.06717400	0.00644200
H	4.86158200	0.09798300	2.14412000
H	4.87155900	-0.03303400	-2.13168600
C	-2.21313500	0.36641500	2.54896600
H	-1.14010800	0.15194800	2.48508100
H	-2.31626400	1.39043400	2.93723200
H	-2.66582100	-0.29862100	3.29606800
C	-2.21447500	0.30918200	-2.55031800
H	-2.34108900	1.31601700	-2.97471800
H	-1.13716800	0.12064000	-2.47709800
H	-2.65009700	-0.39197900	-3.27433800
C	-6.43763300	-0.44843100	0.01080600
H	-6.74041400	-1.05032800	0.87574000
H	-7.00922500	0.48841500	0.05885100
H	-6.75999000	-0.97400000	-0.89549200
C	2.25964400	-0.41386400	-2.52121600
H	1.65147600	-1.32720900	-2.58214000
H	1.55958700	0.41536800	-2.69496300
H	2.97940900	-0.43307700	-3.34857900
C	2.24931100	-0.25986300	2.54629900
H	1.65197900	-1.17422700	2.66738900
H	2.96683000	-0.21544600	3.37469400
H	1.53907500	0.57033200	2.66295600
C	6.52899100	0.26698300	-0.00375900
H	7.06151900	-0.66934500	-0.21877500
H	6.83510000	0.98739900	-0.77229600
H	6.89391000	0.63257700	0.96274100
P	-0.09037200	2.39557000	-0.03006900
P	0.03851400	-2.31725400	0.01891500
C	-1.00217400	-2.84461800	1.43021300
H	-0.47843800	-2.66993700	2.37590100
H	-1.92857200	-2.25820400	1.42724900
H	-1.25266100	-3.90974900	1.34765300
C	1.39884800	-3.53653000	0.02843400
H	2.03098800	-3.39869500	-0.85550900
H	2.02508600	-3.39048000	0.91525900
H	1.00641700	-4.56007200	0.03195700
C	-0.99621400	-2.85970400	-1.39132000
H	-1.92601300	-2.27864300	-1.39313000
H	-0.47238100	-2.68753000	-2.33749900
H	-1.24055400	-3.92573000	-1.30247400
C	-0.76036500	3.39189400	1.35685100
H	-0.34329800	3.03435700	2.30479300
H	-0.51564100	4.45443900	1.23555200
H	-1.85038800	3.28044600	1.38765500
C	-0.73209100	3.33805900	-1.46704800
H	-0.29743600	2.94295900	-2.39189100
H	-1.82160400	3.22767900	-1.51484100
H	-0.48701000	4.40404700	-1.38202500
C	1.67973300	2.82786300	-0.02182200
H	2.18769600	2.39332900	-0.88868800
H	1.78352900	3.91919100	-0.05206700
H	2.16678900	2.44517200	0.88061800

66) Dihydrogen molecule (**H_2**)

Note: Hydrogen atoms are omitted for clarity.

Number of imaginary frequencies = 0

E_{total} = -1.169141 a.u

$G_{correction}$ = -0.001363 a.u

Cartesian coordinates:

H	0.00000000	0.00000000	0.37120900
H	0.00000000	0.00000000	-0.37120900

67) **TS-Mes[II-12)**

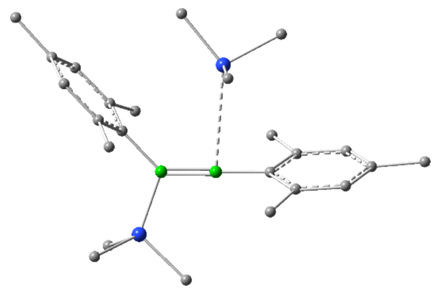

Note: Hydrogen atoms are omitted for clarity.

Number of imaginary frequencies = 1 (31.6 cm^{-1})

E_{total} = -1670.272851 a.u

$G_{correction}$ = 0.510308 a.u

Cartesian coordinates:

C	-2.55497700	-0.41108900	1.26622700
C	-3.71323000	-1.19027700	1.23592800
C	-2.05039200	0.18267800	0.07836900
C	-4.41111100	-1.42931100	0.04662900
H	-4.07981400	-1.63273300	2.16087000
C	-2.75436000	-0.06394900	-1.12431500
C	-3.91017800	-0.86089000	-1.12263500
H	-4.42752600	-1.04194500	-2.06364800
B	-0.73317900	1.07827300	0.10903100
B	0.76556600	0.75966600	0.12284000
C	2.26127900	0.44170300	0.13661900
C	2.87555400	-0.13675500	1.28709700
C	3.07022300	0.66789800	-1.01303700
C	4.21890300	-0.50855500	1.24609400
C	4.41377900	0.28345200	-1.01221800
C	5.00433200	-0.31569300	0.10268500
H	4.67177300	-0.95730400	2.12811400
H	5.01525100	0.45404500	-1.90294500
C	-1.81975200	-0.25112100	2.57626700
H	-0.85059800	-0.75740800	2.52770200
H	-1.60149000	0.79820300	2.79609700
H	-2.39097800	-0.67173300	3.41018300
C	-2.29662300	0.50569600	-2.45114400
H	-2.84546200	1.42012700	-2.70972700
H	-1.23365400	0.75763300	-2.42175200
H	-2.47065900	-0.20604400	-3.26584500
C	-5.63897600	-2.30890400	0.03672500
H	-6.30859700	-2.07167000	0.87079900
H	-6.20503600	-2.19802200	-0.89290500
H	-5.36977200	-3.36828800	0.13412200
C	2.51190700	1.35252300	-2.23914500
H	1.42755400	1.21974100	-2.30029200
H	2.70507300	2.43289300	-2.20216700
H	2.97689500	0.97544500	-3.15579200
C	2.07762200	-0.39919100	2.53802500
H	1.36064400	0.40517300	2.72470200
H	1.48350700	-1.31380900	2.42261100
H	2.72743800	-0.52217800	3.40978200
C	6.44369000	-0.76899200	0.07585300
H	6.51186600	-1.85050500	-0.09839500
H	7.00855000	-0.27328700	-0.71873900
H	6.94315800	-0.56658700	1.02881200
P	-0.96424000	2.94047200	0.19319700
P	0.33948000	-2.25485100	-0.20952000
C	-1.10820900	-3.41896500	-0.18108300
H	-1.29623100	-3.74525200	0.84592100
H	-1.99436900	-2.87968100	-0.52577100
H	-0.94688800	-4.30026400	-0.81291800
C	1.74825200	-3.42328200	0.13671900
H	2.68890000	-2.86686800	0.08299400
H	1.65275000	-3.82433000	1.15063400
H	1.78225100	-4.25910900	-0.57144400
C	0.55721400	-2.04939500	-2.04191600
H	-0.27317700	-1.45878900	-2.43465800
H	1.48388600	-1.49892200	-2.22666000
H	0.59835900	-3.01081400	-2.56691600
C	-1.75431000	3.55612400	1.74812700
H	-1.09567200	3.33388900	2.59097400
H	-1.95636200	4.63149000	1.70999900
H	-2.69313000	3.01468300	1.89332300
C	-2.05026200	3.72052700	-1.08250300
H	-1.62321200	3.54929000	-2.07281000
H	-3.03204400	3.24150300	-1.04218600
H	-2.16514900	4.79513600	-0.90991700
C	0.59526100	3.90701700	0.07880100
H	1.01490900	3.79858100	-0.92348400
H	0.40681500	4.96481700	0.28052400
H	1.31588900	3.52139400	0.80329800

68) 12-Mes

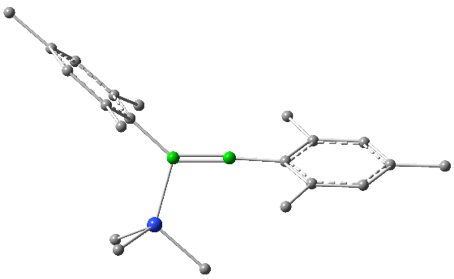

Note: Hydrogen atoms are omitted for clarity.

Number of imaginary frequencies = 0

E_{total} = -1209.290325 a.u

$G_{correction}$ = 0.405695 a.u

Cartesian coordinates:

C	2.88952600	-1.42784500	0.60224100
C	4.23683600	-1.73790000	0.48335200
C	2.33071200	-0.36925900	-0.16855100
C	5.08155900	-1.02357100	-0.37126300
H	4.65181100	-2.55555900	1.07547000
C	3.18691000	0.34806600	-1.04088100
C	4.53904100	0.01531700	-1.11990600
H	5.18602200	0.58006300	-1.79324100
B	0.84537300	-0.06164200	-0.06098800
B	-0.62380600	0.32444100	0.09365300
C	-2.04494500	-0.33722700	-0.17641600
C	-2.64288400	-1.19150300	0.77976700
C	-2.75776700	-0.09836000	-1.37068600

C	-3.90044400	-1.74607400	0.54881100
C	-4.01482200	-0.67536400	-1.57508800
C	-4.61092900	-1.49496100	-0.62410200
H	-4.33853600	-2.40307000	1.30333500
H	-4.54000300	-0.48032000	-2.51256600
C	2.03189400	-2.23462900	1.53020500
H	1.36888600	-1.58757200	2.11877400
H	1.38026900	-2.92338700	0.97611100
H	2.64045900	-2.83715300	2.21358500
C	2.69001900	1.48935200	-1.87614000
H	1.60224700	1.44188800	-2.01436100
H	2.92007200	2.45697700	-1.40733300
H	3.17350300	1.49872600	-2.86019200
C	6.53185200	-1.38168700	-0.46871600
H	7.06680600	-0.72676700	-1.16417400
H	7.02551600	-1.31046100	0.50874900
H	6.66310100	-2.41548500	-0.81243100
C	-2.19506500	0.76584300	-2.46358700
H	-2.43273100	1.82864700	-2.31271600
H	-1.10268100	0.68970900	-2.50838300
H	-2.60751500	0.48631500	-3.44045400
C	-1.92564700	-1.55667300	2.04767600
H	-1.04689500	-2.18136600	1.83936100
H	-1.54797300	-0.67600400	2.58184100
H	-2.57905800	-2.11657900	2.72719300
C	-5.97190700	-2.08545900	-0.84016400
H	-6.27046100	-2.03713800	-1.89334100
H	-6.01040800	-3.13614300	-0.52784100
H	-6.73861200	-1.55464700	-0.25972300
P	-0.61606700	2.05157700	0.83909800
C	-1.37042900	3.38644500	-0.16445700
H	-0.83604000	3.46988000	-1.11770500
H	-2.41906500	3.14010000	-0.36724400
H	-1.32871200	4.34842200	0.36148300
C	1.02316000	2.73551500	1.24845000
H	1.57259100	2.04893300	1.90010200
H	1.60456100	2.90477600	0.33615500
H	0.89711500	3.69399700	1.76501500
C	-1.54267400	2.21232100	2.40984000
H	-2.55993700	1.82838400	2.27244500
H	-1.04705600	1.62320300	3.18898500
H	-1.59807900	3.25989300	2.73098500

69) Trimethylphosphine (**PMe$_3$**)

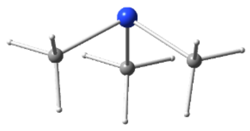

Note: Hydrogen atoms are omitted for clarity.

Number of imaginary frequencies = 0

E_{total} = -460.969973 a.u

$G_{correction}$ = 0.082962 a.u

Cartesian coordinates:

P	0.00006600	-0.00004500	-0.61397300
C	1.55876600	-0.44729400	0.28280200
H	1.87401000	-1.45809900	0.00008700
H	2.36192600	0.24261500	-0.00007900
H	1.43248900	-0.41089800	1.37339000
C	-1.16686100	-1.12607200	0.28275100
H	-2.19974800	-0.89357900	-0.00034500
H	-0.97103100	-2.16667300	0.00020800
H	-1.07260200	-1.03456900	1.37335300
C	-0.39198800	1.57343300	0.28274200
H	0.32586600	2.35178700	0.00011600
H	-1.39101000	1.92407000	-0.00021400
H	-0.36039100	1.44562000	1.37329900

70) **TS-Mes[12-13]**

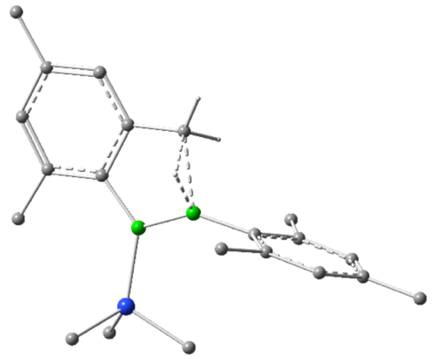

Note: Selected hydrogen atoms are omitted for clarity.

Number of imaginary frequencies = 1 (977.8 cm^{-1})

E_{total} = -1209.263224 a.u

$G_{correction}$ = 0.405364 a.u

Cartesian coordinates:

C	-2.60718400	-0.72125500	-1.17829400
C	-3.99575900	-0.84530200	-1.28336500
C	-1.98386400	-0.68203800	0.08726200
C	-4.81037600	-0.93823100	-0.16121300
H	-4.45274000	-0.86847200	-2.27493500
C	-2.81673800	-0.78255900	1.22783800
C	-4.19762500	-0.90180200	1.09157600
H	-4.81906900	-0.96842300	1.98716000
B	-0.42516000	-0.48929700	0.24513300
B	0.78144000	0.53322500	0.02583900
C	2.16516100	-0.15578000	-0.08022900
C	2.03557800	-1.57241600	-0.04732100
C	3.49418700	0.35174100	-0.14442500
C	3.14590500	-2.42074300	0.06864700
C	4.57362800	-0.51288300	-0.09003900
C	4.42855000	-1.90710600	0.03396700
H	2.99294800	-3.49891400	0.14630700
H	5.58388700	-0.09856200	-0.13091400
C	-1.80921200	-0.65088100	-2.45018900
H	-0.88768400	-0.07002600	-2.32143000
H	-1.50718600	-1.65203300	-2.79117300
H	-2.39326700	-0.20751800	-3.26561700
C	-2.23201200	-0.75152700	2.61186600
H	-1.59228600	-1.62418700	2.80129500
H	-1.59387100	0.12743900	2.77082200
H	-3.01781800	-0.74656600	3.37621300

C	-6.29749500	-1.08306000	-0.28112300
H	-6.63719800	-0.92806800	-1.31105500
H	-6.62879900	-2.08348400	0.02706200
H	-6.82591700	-0.36547700	0.35879100
C	3.75653000	1.82017000	-0.27992700
H	3.39030400	2.20281800	-1.24235100
H	3.26614600	2.40082400	0.51381500
H	4.82786500	2.04421000	-0.22763500
C	0.67263000	-2.08128900	-0.04211100
H	0.08533400	-1.36386600	1.03969700
C	5.63933400	-2.78735900	0.10348700
H	6.24317300	-2.71992100	-0.81141100
H	6.29765800	-2.50316000	0.93500400
H	5.36547700	-3.83921200	0.24126000
P	0.30518600	2.38174800	0.06591300
C	0.70376700	3.23770200	1.62958400
H	0.22672600	2.69870300	2.45547000
H	1.78403500	3.24513800	1.80594700
H	0.33570300	4.27060500	1.60961500
C	-1.49976300	2.58763700	-0.04692300
H	-1.85530800	2.27183100	-1.03317600
H	-2.00592400	1.97997800	0.70921500
H	-1.75552800	3.64299300	0.10347100
C	0.89237000	3.53977100	-1.22365100
H	1.95253000	3.77222500	-1.09370100
H	0.74838400	3.08849100	-2.21148200
H	0.31786300	4.47221500	-1.17116000
H	0.13743500	-2.00450400	-0.99402700
H	0.51226600	-3.07766600	0.38287000

71) 13-Mes

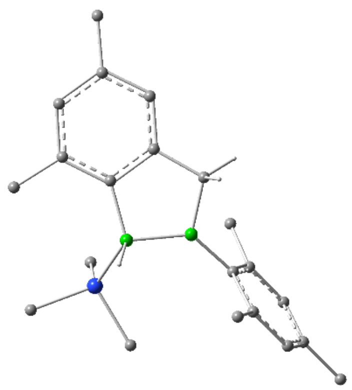

Note: Selected hydrogen atoms are omitted for clarity.

Number of imaginary frequencies = 0

E_{total} = -1209.348925 a.u

$G_{correction}$ = 0.407530 a.u

Cartesian coordinates:

C	-2.34002700	-1.04545000	-1.00464700
C	-3.71217700	-1.05736300	-1.26541500
C	-1.87149400	-0.80399500	0.29832100
C	-4.64844200	-0.86743700	-0.25344400
H	-4.05725800	-1.22363800	-2.28823200
C	-2.81467700	-0.62232600	1.33051700
C	-4.17583400	-0.65797500	1.04422900
H	-4.89401700	-0.51039300	1.85372900
B	-0.33025900	-0.67665200	0.53993300
B	0.72985300	0.66948300	0.77319500
C	2.15205900	-0.02565900	0.42755000
C	2.01533700	-1.42688000	0.32528200
C	3.44612900	0.50779600	0.29471300
C	3.10220400	-2.25227000	0.05182800
C	4.52627000	-0.33444500	0.01837600
C	4.37610100	-1.71388500	-0.12189500
H	2.95853200	-3.33261400	-0.02157300
H	5.52323800	0.10008900	-0.08587800
C	-1.37620300	-1.29712500	-2.13385300
H	-0.37981000	-0.87084400	-1.94880800
H	-1.22572100	-2.37280600	-2.29870800
H	-1.74481300	-0.88192200	-3.08041500
C	-2.34259500	-0.40483900	2.73861000
H	-1.92326700	-1.32582200	3.16744200
H	-1.54341900	0.34768000	2.78488400
H	-3.15616200	-0.08103300	3.39736200
C	-6.12093500	-0.90255300	-0.53325500
H	-6.58116200	-1.82161000	-0.14697000
H	-6.64320100	-0.06395500	-0.05642600
H	-6.32912800	-0.86045300	-1.60804800
C	3.70460300	1.97798300	0.44691500
H	3.47004200	2.53270900	-0.47379000
H	3.10205200	2.40979100	1.25589600
H	4.75893400	2.17898900	0.67068000
C	0.62094200	-1.93892800	0.57144800
H	0.61747600	1.08843500	1.92429500
C	5.54836600	-2.58684100	-0.45751000
H	5.67093900	-2.69832900	-1.54357000
H	6.48630800	-2.17115600	-0.07105100
H	5.43150100	-3.59551600	-0.04442400
P	0.13379800	2.22191300	-0.22327500
C	0.85276000	3.85278300	0.18601300
H	0.84409500	3.98693700	1.27363600
H	1.88320600	3.93664700	-0.16770700
H	0.25589100	4.64851300	-0.27517300
C	-1.63773300	2.53991500	0.05489900
H	-2.24030200	1.69223800	-0.28688800
H	-1.81706600	2.68360600	1.12665800
H	-1.94587000	3.44545800	-0.48039800
C	0.29733900	2.11498800	-2.03475900
H	1.31601900	1.81102800	-2.30037200
H	-0.39965300	1.36468700	-2.42358900
H	0.07305800	3.07912100	-2.50520500
H	0.33443000	-2.78617600	-0.07042600
H	0.53822100	-2.33549600	1.60301100

72) TS-Mes[12-17]

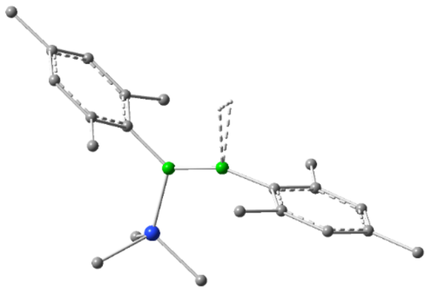

Note: Selected hydrogen atoms are omitted for clarity.

Number of imaginary frequencies = 1 (805.0 cm^{-1})

E_{total} = -1210.455314 a.u

$G_{correction}$ = 0.418045 a.u

Cartesian coordinates:

C	2.82184100	-0.67024900	-1.16372100
C	4.14075700	-1.12555700	-1.11719900
C	2.13750100	-0.32437000	0.02272400
C	4.82714600	-1.26446700	0.08603400
H	4.64631500	-1.38665500	-2.04932600
C	2.82555500	-0.49746900	1.24437100
C	4.14452200	-0.95462800	1.25925800
H	4.65319100	-1.08000700	2.21747500
B	0.64019300	0.20645900	-0.01385200
C	-2.27085000	-0.65889200	0.04113200
C	-2.99566100	-0.80021300	-1.16392600
C	-2.99694100	-0.61619300	1.25592200
C	-4.38879300	-0.87600000	-1.14051600
C	-4.38733700	-0.69404200	1.24404900
C	-5.10231700	-0.82441200	0.05360500
H	-4.93347100	-0.98033400	-2.08034000
H	-4.93364800	-0.65519900	2.18845300
C	2.13490900	-0.60518800	-2.49854500
H	1.30020400	-1.31672900	-2.55063400
H	1.69493400	0.38045100	-2.69669900
H	2.82873300	-0.83621900	-3.31552000
C	2.14136800	-0.24575500	2.55836000
H	1.68949500	0.75268700	2.61245000
H	1.31592800	-0.95247100	2.71709200
H	2.83964700	-0.34726400	3.39757800
C	6.25472700	-1.72126600	0.11667700
H	6.48582500	-2.26306800	1.04127300
H	6.48521600	-2.38200000	-0.72708000
H	6.95089000	-0.87342400	0.05986900
C	-2.28999500	-0.50667200	2.57631900
H	-1.70519000	-1.41183800	2.79086500
H	-1.57083700	0.32199200	2.59341300
H	-3.00194100	-0.36928300	3.39815600
C	-2.29036500	-0.89095500	-2.48655700
H	-1.72266400	-1.82808200	-2.57133600
H	-3.00148500	-0.85925900	-3.31992100
H	-1.55644200	-0.08600500	-2.61922000
C	-6.59693500	-0.92543100	0.07256100
H	-7.04221400	-0.17591800	0.73782300
H	-7.02550600	-0.78958000	-0.92617500
H	-6.92325400	-1.90743900	0.43938300
P	0.40718400	2.08307400	-0.14052700
C	1.13044300	3.06968700	1.22045400
H	2.19791500	2.83948700	1.31362100
H	1.01377000	4.14394300	1.03188800
H	0.63083600	2.81562100	2.16215300
C	-1.32150600	2.65183400	-0.19442000
H	-1.83758400	2.22377400	-1.05946600
H	-1.85362900	2.34845400	0.71259400
H	-1.34345900	3.74519400	-0.27172100
C	1.15447500	2.87709600	-1.60995400
H	0.66978600	2.49740800	-2.51654200
H	1.03644500	3.96686900	-1.57059000
H	2.22287200	2.63690600	-1.65345700
B	-0.72502300	-0.50981100	0.03078700
H	-0.16479400	-2.11534100	0.53039500
H	-0.16476500	-2.16717700	-0.25300200

73) 17-Mes

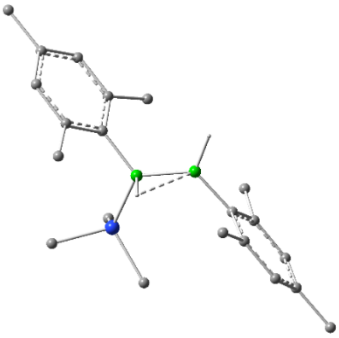

Note: Selected hydrogen atoms are omitted for clarity.

Number of imaginary frequencies = 0

E_{total} = -1210.538144 a.u

$G_{correction}$ = 0.427148 a.u

Cartesian coordinates:

C	2.64988300	1.24331300	-0.36365600
C	4.01595600	1.49012100	-0.51347400
C	2.14248200	-0.07571000	-0.38257000
C	4.93226600	0.45951700	-0.69736200
H	4.37428500	2.52147500	-0.49628500
C	3.07469500	-1.11731100	-0.59898100
C	4.43492800	-0.83920300	-0.74935300
H	5.12956600	-1.66443800	-0.91963300
B	0.56990400	-0.35958700	-0.18848400
C	-2.16482800	0.27184200	-0.95385000
C	-2.80734700	1.34792700	-0.31149900
C	-2.95109700	-0.82359500	-1.36205500
C	-4.17563200	1.29758200	-0.04815500
C	-4.31679400	-0.84938200	-1.07955700
C	-4.95103900	0.19955400	-0.41723400
H	-4.65158900	2.14216300	0.45466900
H	-4.90580100	-1.71403500	-1.39269900
C	1.73660000	2.42952500	-0.22946700

H	0.99737500	2.45706400	-1.04130300
H	1.17119900	2.42718800	0.71123800
H	2.30042200	3.36874800	-0.26570400
C	2.64094600	-2.55222200	-0.72081000
H	3.50430800	-3.21437600	-0.85287400
H	2.08736400	-2.90318600	0.15878200
H	1.97550400	-2.70037800	-1.58087400
C	6.40025400	0.73566400	-0.82304000
H	6.58911500	1.72672400	-1.25158000
H	6.90137700	0.70796500	0.15418100
H	6.89765200	-0.00655600	-1.45812500
C	-2.32277500	-1.98391300	-2.07998300
H	-1.60650200	-1.64590700	-2.84127300
H	-1.76365700	-2.63977300	-1.39805600
H	-3.07733500	-2.60192500	-2.57971300
C	-2.02139200	2.57009600	0.06371800
H	-1.77407600	3.17394100	-0.82115400
H	-2.57233900	3.21771600	0.75536900
H	-1.06128300	2.30732500	0.52834000
C	-6.42601700	0.16884500	-0.15036600
H	-6.99405700	0.60555900	-0.98278200
H	-6.79156600	-0.85578000	-0.01663000
H	-6.68607100	0.74010800	0.74832600
P	0.12532300	-0.58251800	1.69193400
C	1.13529600	-1.85130000	2.52676600
H	2.19933700	-1.62737900	2.39263900
H	0.90859900	-1.88059600	3.59902800
H	0.92327500	-2.83520800	2.09423200
C	-1.58156700	-1.11699100	2.02801800
H	-2.29426800	-0.34448000	1.71972600
H	-1.79993700	-2.03084300	1.46452800
H	-1.70409200	-1.32175000	3.09824200
C	0.36123500	0.86579800	2.77429900
H	-0.30431100	1.68346100	2.47824300
H	0.14347500	0.59613500	3.81439500
H	1.40021000	1.20832900	2.71055000
B	-0.61001800	0.27167700	-1.19133500
H	0.21569500	-1.47225700	-0.61082300
H	-0.24748000	0.53133800	-2.32122700

74) TS-Mes[17-11]

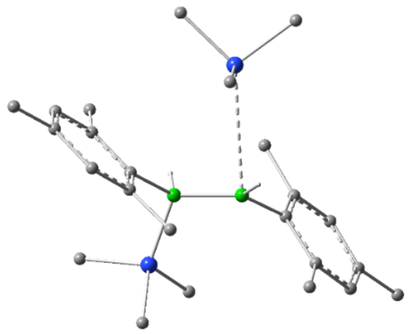

Note: Selected hydrogen atoms are omitted for clarity.

Number of imaginary frequencies = 1 (27.9 cm^{-1})

E_{total} = -1671.515338 a.u

$G_{correction}$ = 0.531490 a.u

Cartesian coordinates:

C	2.93247500	1.00196500	-0.81778200
C	4.32334700	0.96203300	-0.96766500
C	2.33506100	0.54023000	0.37924000
C	5.16318700	0.45156400	0.02436200
H	4.76404500	1.33462100	-1.89123100
C	3.19041600	0.06084300	1.40666400
C	4.57308600	0.00205300	1.20923200
H	5.20715300	-0.38841900	2.00353600
B	0.78727900	0.38529800	0.61110700
B	-0.19734100	-0.61374200	-0.37200300
C	-1.79126300	-0.72739700	-0.10211000
C	-2.72407300	-0.50314200	-1.15406000
C	-2.32988900	-1.00905100	1.17815300
C	-4.10171300	-0.55633700	-0.91547500
C	-3.71703700	-1.06829200	1.38095100
C	-4.62456800	-0.84416100	0.34849900
H	-4.78927800	-0.36880000	-1.73869400
H	-4.09437600	-1.28451500	2.37963900
C	2.10586300	1.55094400	-1.96313300
H	1.85264000	0.76469800	-2.68463600
H	1.16365100	1.97930400	-1.61616400
H	2.66012100	2.32377400	-2.50634200
C	2.61737700	-0.42576600	2.72183000
H	1.98595700	0.33944200	3.18578900
H	1.98180800	-1.31147900	2.59618500
H	3.40888300	-0.69301500	3.42796900
C	6.66245800	0.43087600	-0.15564900
H	6.93896500	0.33792600	-1.21071500
H	7.11980400	1.35554600	0.21895800
H	7.12132300	-0.40005600	0.38978400
C	-1.46543100	-1.18886900	2.41135400
H	-1.86605400	-1.97250700	3.06425700
H	-0.43242900	-1.43579800	2.17667900
H	-1.43472800	-0.26177800	2.99534100
C	-2.27307300	-0.16170700	-2.56040700
H	-1.63201600	0.72464600	-2.56915000
H	-1.69044200	-0.96997300	-3.01340100
H	-3.13243500	0.03217800	-3.20966600
C	-6.11517700	-0.93276100	0.57334200
H	-6.65339800	-0.17745700	-0.00902200
H	-6.50719400	-1.91215100	0.27013600
H	-6.37132500	-0.79180900	1.62792700
P	-1.19904700	2.91995600	-0.03566800
P	0.54217900	-2.40427600	-0.59226500
C	0.48872200	-3.59051200	0.81361900
H	1.10411500	-3.22264100	1.63767500
H	-0.54209800	-3.68504800	1.16275300
H	0.85790800	-4.57207600	0.50273600
C	2.29353800	-2.43933200	-1.13433800
H	2.41489700	-1.76640500	-1.98569300
H	2.93687500	-2.07534800	-0.33077300
H	2.58769600	-3.45349200	-1.41946100
C	-0.36486000	-3.31310300	-1.90665300
H	-1.43381400	-3.26910300	-1.68328200
H	-0.19103400	-2.81950400	-2.86543300
H	-0.04078700	-4.35559900	-1.97096800
C	-0.67283000	4.69234800	0.20272700
H	-0.27960200	5.08854700	-0.73856800

H	-1.50014100	5.32974400	0.53608200
H	0.12933900	4.73685000	0.94555100
C	-2.05437900	2.61748100	1.58534100
H	-2.58492700	1.66354300	1.52765700
H	-1.31081200	2.54468400	2.38344500
H	-2.76714900	3.41407200	1.82780100
C	-2.70015300	3.17736700	-1.10822500
H	-3.25729400	2.23815200	-1.15969800
H	-3.35668800	3.95868400	-0.70806800
H	-2.39703200	3.45439900	-2.12228900
H	0.06442500	-0.28937600	-1.51287000
H	0.36227300	0.89891400	1.61081700

75) 11-Mes

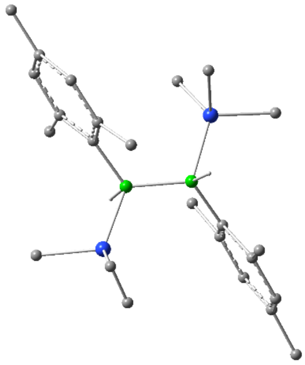

Note: Selected hydrogen atoms are omitted for clarity.

Number of imaginary frequencies = 0

E_{total} = -1671.544680 a.u

$G_{correction}$ = 0.534780 a.u

Cartesian coordinates:

C	-2.77143900	-0.75701100	-1.03411900
C	-4.13220300	-0.53943600	-1.26927600
C	-2.17891300	-0.44697100	0.21206100
C	-4.97661800	-0.02284800	-0.29449400
H	-4.54380500	-0.78515000	-2.25095900
C	-3.05687200	0.05034800	1.21176000
C	-4.41266700	0.25576800	0.94779900
H	-5.05381300	0.64616600	1.74163300
B	-0.58807200	-0.53514200	0.51443400
B	0.48167800	0.57507500	-0.37287400
C	2.09227500	0.41506100	-0.22351400
C	2.89020300	0.07980700	-1.35519000
C	2.77770400	0.52598300	1.00718000
C	4.25616700	-0.16700900	-1.22616600
C	4.15527800	0.28730000	1.09994500
C	4.91751500	-0.07011200	-0.00179000
H	4.83290100	-0.43505300	-2.11449300
H	4.63901700	0.37964200	2.07584000
C	-1.97986500	-1.36718500	-2.15718700
H	-0.98177200	-0.92441600	-2.24638600
H	-1.85193000	-2.45137500	-2.01682000
H	-2.49276700	-1.24170300	-3.11813900
C	-2.57727900	0.39926000	2.59532500
H	-2.16005900	-0.46893900	3.12136400
H	-1.77798500	1.15297700	2.58364200
H	-3.39780600	0.79611700	3.20482500
C	-6.42737100	0.24029600	-0.56596900
H	-7.05560500	-0.03741900	0.28922100
H	-6.61660300	1.30394500	-0.76526400
H	-6.78254100	-0.31955000	-1.43869900
C	2.09213300	0.87886000	2.30163000
H	2.47742900	1.82365500	2.71083600
H	1.00888900	0.96052600	2.19852300
H	2.28734100	0.11437100	3.06687500
C	2.31307900	-0.02344800	-2.74245400
H	1.49635600	-0.75314300	-2.80989400
H	1.89300000	0.93071700	-3.08533100
H	3.08502300	-0.32102400	-3.46216400
C	6.38982000	-0.33227200	0.10080400
H	6.73840800	-0.27591800	1.13802100
H	6.65138800	-1.32601600	-0.28502500
H	6.97110600	0.39360500	-0.48296500
P	0.10664600	-2.37058600	0.37895800
P	-0.03705700	2.44974600	-0.17807000
C	0.06874300	3.39168200	1.38768600
H	-0.48579500	2.88953200	2.18752100
H	1.11534900	3.48782400	1.69492600
H	-0.34924700	4.39585200	1.24744900
C	-1.74770400	2.75928800	-0.72444900
H	-1.91192900	2.28272800	-1.69689700
H	-2.46089800	2.32919600	-0.01221500
H	-1.92742500	3.83721500	-0.81556400
C	0.95127900	3.49586300	-1.30308700
H	2.01610400	3.38381400	-1.06968800
H	0.78593300	3.17114700	-2.33625000
H	0.67195600	4.55236900	-1.21314300
C	-1.15365100	-3.61305500	0.83259000
H	-1.95420200	-3.64188100	0.08609700
H	-0.71488600	-4.61287400	0.93170200
H	-1.59993700	-3.31876200	1.79027600
C	1.40912500	-2.65437900	1.62456300
H	2.27243600	-2.01456900	1.40566900
H	1.02634400	-2.40560200	2.61982100
H	1.72898000	-3.70312000	1.61264800
C	0.91171700	-3.06219200	-1.11259900
H	1.82732000	-2.49155300	-1.31000900
H	1.18499500	-4.10963300	-0.93654900
H	0.26378900	-3.00817700	-1.99138700
H	0.16608200	0.58937500	-1.55742400
H	-0.39575000	-0.50354600	1.72096800

76) TS-Mes[17-18]

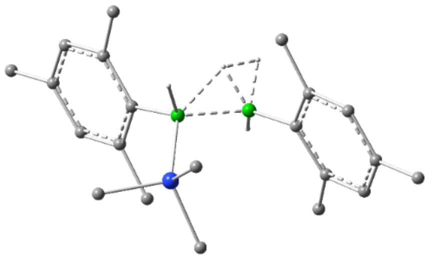

Note: Selected hydrogen atoms are omitted for clarity.

Number of imaginary frequencies = 1 (965.7 cm^{-1})

E_{total} = -1211.701396 a.u

$G_{correction}$ = 0.445057 a.u

Cartesian coordinates:

C	-2.80969100	-0.88774300	-0.58634500
C	-4.17125900	-0.90909800	-0.89993600
C	-2.20606200	0.26063900	-0.03232500
C	-4.99109700	0.19172200	-0.68774400
H	-4.60004100	-1.81652500	-1.33027800
C	-3.04483400	1.38684000	0.16908600
C	-4.39994700	1.33526900	-0.15569300
H	-5.01789000	2.22097400	0.00730600
B	-0.63448200	0.39529600	0.29038400
C	2.01604100	0.51259900	-0.98228300
C	2.76020800	-0.61612500	-1.38967300
C	2.71989800	1.55718800	-0.34130600
C	4.12044400	-0.71728000	-1.08612100
C	4.08288200	1.43404000	-0.06796800
C	4.80408300	0.29446200	-0.41810900
H	4.66697200	-1.60995800	-1.39769700
H	4.59855800	2.25467600	0.43536400
P	0.13454600	-0.93254700	1.50299900
B	0.44515800	0.54896900	-1.22577200
H	0.19731100	1.69900300	-1.77595600
H	-0.08475700	-0.13352400	-2.05638200
H	-0.19464600	1.56518100	-0.86721300
H	-0.45530600	1.28447100	1.10779100
C	-6.45419500	0.16020700	-1.01182000
H	-6.75740400	1.03909300	-1.59419700
H	-6.72037100	-0.73190400	-1.58949900
H	-7.06900100	0.15736900	-0.10187400
C	-2.02481900	-2.14136000	-0.84754600
H	-1.02784400	-1.92396900	-1.24911300
H	-1.89628500	-2.74077200	0.06635700
H	-2.53971900	-2.78728000	-1.56781800
C	-2.52234300	2.68425700	0.72669900
H	-2.16104400	2.57626800	1.75772100
H	-1.67902500	3.08648300	0.14795600
H	-3.30694000	3.44936500	0.72961400
C	2.12976300	-1.72345800	-2.18990700
H	2.83481700	-2.54859500	-2.34429400
H	1.23019700	-2.13744400	-1.71826000
H	1.81472000	-1.36725000	-3.17934900
C	6.27107100	0.18573200	-0.12861200
H	6.87300900	0.66060900	-0.91509700
H	6.53506400	0.67828100	0.81471800
H	6.59388600	-0.85975800	-0.06500500
C	2.02562700	2.82919000	0.05495300
H	1.59401900	3.34289900	-0.81649100
H	1.19806900	2.65256000	0.75576200
H	2.71981200	3.52982500	0.53156700
C	0.89336400	-2.52806400	1.03838700
H	0.23957600	-3.13257800	0.40533600
H	1.83172100	-2.33327000	0.50690600
H	1.12246500	-3.09027400	1.95157500
C	1.47032500	-0.17542500	2.48114200
H	2.30340000	0.10115600	1.82404800
H	1.09906200	0.72595600	2.97940300
H	1.82942900	-0.88303600	3.23751800
C	-1.11845900	-1.35956100	2.75598100
H	-0.66928400	-1.91698600	3.58583100
H	-1.55916200	-0.43189400	3.13967000
H	-1.92093300	-1.95645400	2.31086700

77) 18-Mes

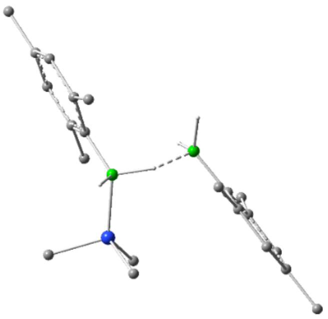

Note: Selected hydrogen atoms are omitted for clarity.

Number of imaginary frequencies = 0

E_{total} = -1211.738818 a.u

$G_{correction}$ = 0.445358 a.u

Cartesian coordinates:

C	-2.89953500	-0.71722300	-0.70203100
C	-4.21079600	-0.53261000	-1.14728700
C	-2.32406000	0.16398600	0.23356600
C	-4.99279200	0.52846700	-0.70839300
H	-4.62684800	-1.23484700	-1.87202800
C	-3.11057000	1.26645800	0.65156300
C	-4.41303200	1.42770000	0.18336400
H	-4.99381700	2.28923000	0.51906300
B	-0.85306800	0.07081800	0.83909700
C	2.03563800	0.97118500	-0.81581200
C	2.77322700	0.12100200	-1.67082700
C	2.73157100	1.60627200	0.23867100
C	4.12994500	-0.11129800	-1.43519500
C	4.08852900	1.35523900	0.44650600
C	4.80675900	0.49126500	-0.37751000
H	4.67984900	-0.77561500	-2.10475400
H	4.60181600	1.85099100	1.27288500
P	0.14117600	-1.57984700	1.19817000
B	0.48257300	1.19872100	-1.01883000

H	0.04640200	2.32235100	-0.95676700
H	-0.11106000	0.46808900	-1.76445600
H	-0.00509100	0.85183000	0.33235200
H	-0.80702300	0.44157000	2.00089500
C	-6.40468600	0.71122100	-1.17620600
H	-6.59107300	1.74273500	-1.49927900
H	-6.64110600	0.04860600	-2.01601400
H	-7.12430600	0.49605600	-0.37518600
C	-2.12742300	-1.86489000	-1.28480400
H	-1.14827700	-1.53615800	-1.65671900
H	-1.96145400	-2.67521100	-0.56082500
H	-2.66326900	-2.31181900	-2.12939800
C	-2.56790900	2.31282300	1.58490700
H	-2.39236500	1.91730100	2.59384000
H	-1.60793500	2.71487300	1.23537000
H	-3.26551300	3.15278700	1.67590100
C	2.14252600	-0.53177200	-2.87078100
H	2.86312600	-1.16996900	-3.39465300
H	1.27373900	-1.14877200	-2.60908900
H	1.77708100	0.21361000	-3.58766900
C	6.27016800	0.25353100	-0.15882600
H	6.87836000	1.00398600	-0.68126400
H	6.53512000	0.31130000	0.90321700
H	6.58095600	-0.72833000	-0.53368300
C	2.02384800	2.54309500	1.17434600
H	1.48344200	3.32600000	0.62790500
H	1.27154100	2.02516400	1.78688500
H	2.72840600	3.02944700	1.85801800
C	0.96626000	-2.65172800	-0.02415600
H	0.27469700	-3.05124900	-0.76904700
H	1.75034100	-2.07319700	-0.52688000
H	1.43895500	-3.48670600	0.50663300
C	1.52880700	-1.13163500	2.28542800
H	2.24408800	-0.50956600	1.73145000
H	1.16165100	-0.56914200	3.14960500
H	2.04129800	-2.03555600	2.63376800
C	-0.88706600	-2.68444200	2.21437900
H	-0.29242900	-3.51789800	2.60438900
H	-1.29559500	-2.11024500	3.05327200
H	-1.72533500	-3.08153400	1.63294900

78) 10-Mes

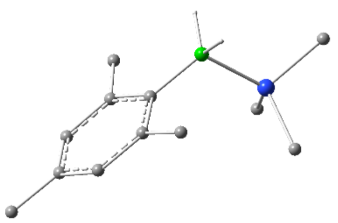

Note: Selected hydrogen atoms are omitted for clarity.

Number of imaginary frequencies = 0

E_{total} = -836.371913 a.u

$G_{correction}$ = 0.262216 a.u

Cartesian coordinates:

B	0.80733000	-1.42877000	0.00000000
H	1.25996500	-1.96326700	0.99810400
H	1.25996500	-1.96326700	-0.99810400
P	-1.01934000	-2.11596300	0.00000000
C	-2.05574500	-1.66920400	-1.42938800
H	-1.59311500	-2.03820400	-2.35126500
H	-3.05598400	-2.10788900	-1.33444400
H	-2.15058900	-0.57937000	-1.49213400
C	-1.01195100	-3.93411600	0.00000000
H	-0.47818300	-4.29173500	0.88711400
H	-2.02963200	-4.34052500	0.00000000
H	-0.47818300	-4.29173500	-0.88711400
C	-2.05574500	-1.66920400	1.42938800
H	-1.59311500	-2.03820400	2.35126500
H	-2.15058900	-0.57937000	1.49213400
H	-3.05598400	-2.10788900	1.33444400
C	0.83304100	0.17888100	0.00000000
C	0.84527400	0.91674100	1.20556300
C	0.84527400	0.91674100	-1.20556300
C	0.84527400	2.31295100	1.18961900
C	0.84527400	2.31295100	-1.18961900
C	0.84248900	3.03495300	0.00000000
H	0.85258400	2.85476700	2.13784200
H	0.85258400	2.85476700	-2.13784200
C	0.88904800	0.23205500	2.54366700
H	0.03296000	-0.43458800	2.70678800
H	1.78513900	-0.39281200	2.64560100
H	0.89541000	0.96312100	3.36025200
C	0.88904800	0.23205500	-2.54366700
H	0.03296000	-0.43458800	-2.70678800
H	0.89541000	0.96312100	-3.36025200
H	1.78513900	-0.39281200	-2.64560100
C	0.87502800	4.53385000	0.00000000
H	0.38159200	4.94903100	0.88654000
H	1.90566500	4.91379400	0.00000000
H	0.38159200	4.94903100	-0.88654000

79) TS-Mes[17-19]

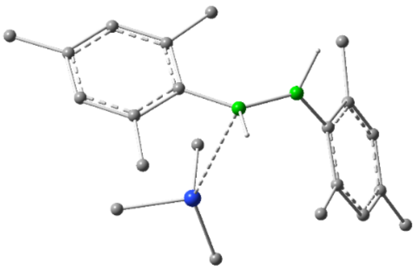

Note: Selected hydrogen atoms are omitted for clarity.

Number of imaginary frequencies = 1 (17.5 cm^{-1})

E_{total} = -1210.509618 a.u

$G_{correction}$ = 0.425846 a.u

Cartesian coordinates:

C	-2.31927000	1.55890500	-0.04600300
C	-3.52368300	1.50551200	-0.75195900
C	-2.02919200	0.60033200	0.96375400
C	-4.47234900	0.50965800	-0.50667900

H	-3.72767800	2.25311200	-1.51528400
C	-3.00409100	-0.40738500	1.21768600
C	-4.19182000	-0.43748700	0.48298000
H	-4.91958600	-1.22087400	0.68395600
B	-0.69394600	0.63254000	1.76755500
C	2.06214300	0.98301500	0.88537000
C	2.74462900	1.75557700	-0.08956400
C	2.63890500	-0.25970500	1.27399200
C	3.90010200	1.25378500	-0.70378000
C	3.81149700	-0.70631600	0.66471800
C	4.44378400	0.02037100	-0.35163200
H	4.39297300	1.85201300	-1.46753100
H	4.24955500	-1.64795600	0.98961600
C	-1.34188600	2.66035400	-0.37887000
H	-1.20063200	3.34994100	0.45920300
H	-0.35963800	2.24586500	-0.62351400
H	-1.67309300	3.24425000	-1.24227100
C	-2.76812300	-1.49967600	2.23494600
H	-3.62706000	-2.17387000	2.29283200
H	-1.88557700	-2.09230800	1.97061800
H	-2.58645300	-1.08984200	3.23305300
C	-5.74763600	0.43299900	-1.30936200
H	-6.58071200	0.06454400	-0.70272200
H	-6.02624300	1.40901200	-1.71721400
H	-5.63423400	-0.25571200	-2.15605400
C	2.03773000	-1.09624000	2.38209800
H	1.88247800	-0.51171500	3.29444700
H	1.06557300	-1.49675900	2.08225600
H	2.68764800	-1.93953700	2.63269900
C	2.24461200	3.11114200	-0.53820000
H	1.73622400	3.64573400	0.26862400
H	3.07215100	3.73199300	-0.89412100
H	1.53087500	3.02461800	-1.36657300
C	5.67166200	-0.52453000	-1.03933200
H	6.22790700	0.26328500	-1.55552700
H	6.34763600	-1.00824500	-0.32663200
H	5.39903600	-1.27945600	-1.78751500
P	-0.04450700	-1.74439400	-0.61893700
C	-1.54315300	-2.61967500	-1.29063300
H	-2.41753900	-1.97645200	-1.15701700
H	-1.43720000	-2.86865000	-2.35264800
H	-1.71426700	-3.54178500	-0.72672000
C	1.30077600	-2.91856000	-1.14818500
H	2.26902200	-2.44944000	-0.95117600
H	1.24235300	-3.83840700	-0.55830100
H	1.23512900	-3.17482900	-2.21190200
C	0.15739100	-0.44059800	-1.92880600
H	1.07160400	0.12718300	-1.73616200
H	0.20759500	-0.87585700	-2.93333700
H	-0.69502200	0.24190200	-1.88260600
B	0.71536400	1.48345800	1.50536100
H	-0.69988500	0.06418500	2.82863700
H	0.69540500	2.62111400	1.91313700

80) 19-Mes

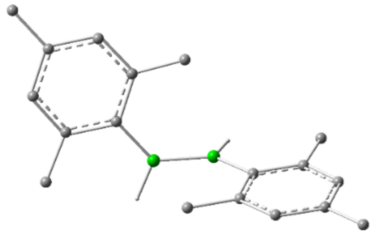

Note: Selected hydrogen atoms are omitted for clarity.

Number of imaginary frequencies = 0

E_{total} = -749.534357 a.u

$G_{correction}$ = 0.318328 a.u

Cartesian coordinates:

C	-3.13692900	0.99589200	0.68851400
C	-4.29788800	0.70014800	-0.02025700
C	-2.01442300	0.13067700	0.61066300
C	-4.40144100	-0.44070800	-0.81469700
H	-5.15096200	1.37702700	0.04538900
C	-2.11672600	-1.01579700	-0.21844200
C	-3.29699700	-1.28560700	-0.90561100
H	-3.35943400	-2.17446300	-1.53491400
B	-0.71465300	0.42179000	1.40507000
B	0.71386000	-0.41388700	1.40734400
C	2.01352200	-0.12600800	0.61161500
C	3.13869800	-0.98963300	0.69575200
C	2.11542500	1.01630300	-0.22082700
C	4.29933700	-0.69455700	-0.01054000
C	3.29822200	1.28618600	-0.90697300
C	4.40157300	0.44307400	-0.81250900
H	5.15578500	-1.36706000	0.06169300
H	3.36107300	2.17459100	-1.53646100
C	-0.96535700	-1.96356800	-0.38834200
H	-0.09133900	-1.46661000	-0.83168100
H	0.75290600	-1.31353200	2.21322600
H	-0.75366400	1.32528500	2.20663900
H	-1.23039700	-2.79797200	-1.04660200
H	-0.64266300	-2.39640200	0.56865200
C	-3.11213400	2.25586900	1.50576200
H	-2.93121700	2.05243300	2.56748000
H	-2.31802700	2.93816200	1.17830100
H	-4.06407800	2.79119300	1.42488000
C	-5.67356900	-0.76095700	-1.53521300
H	-6.33792000	-1.36802000	-0.90592800
H	-6.22572700	0.14634000	-1.80364300
H	-5.48649500	-1.33300100	-2.45059600
C	0.96473500	1.96410400	-0.39518200
H	0.09044000	1.46560300	-0.83622500
H	0.64227000	2.40198700	0.55958600
H	1.23024600	2.79503200	-1.05761600
C	3.11409800	-2.24412800	1.52129300
H	2.93342400	-2.03385700	2.58174600
H	2.31972500	-2.92830400	1.19839800
H	4.06596400	-2.78007400	1.44352400
C	5.67048100	0.73471200	-1.55086600
H	6.51396300	0.84865200	-0.85841600

H	5.93101500	-0.08526000	-2.23171700
H	5.59531400	1.65271600	-2.14263200

81) TS-Mes[19-20]

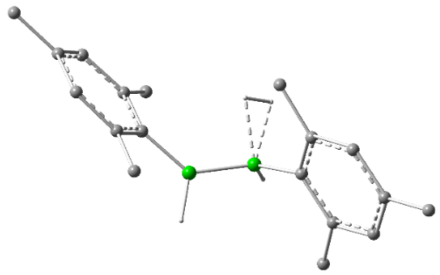

Note: Selected hydrogen atoms are omitted for clarity.

Number of imaginary frequencies = 1 (545.8 cm^{-1})

E_{total} = -750.686760 a.u

$G_{correction}$ = 0.332167 a.u

Cartesian coordinates:

C	2.86268800	1.13954600	-0.33781200
C	4.10002400	0.54677100	-0.60096700
C	1.93426000	0.49212400	0.49967900
C	4.44368600	-0.69184200	-0.07426200
H	4.81388000	1.07362200	-1.23726900
C	2.29412300	-0.75890700	1.06472500
C	3.52064200	-1.33264300	0.75744800
H	3.77561700	-2.30501900	1.18294800
B	0.52138100	1.06927100	0.79946400
C	-2.00528000	0.65691400	-0.27322800
C	-3.17558200	1.09083700	0.39043900
C	-2.01947700	-0.61303700	-0.88336000
C	-4.30897200	0.28289000	0.41163600
C	-3.17908200	-1.39191800	-0.85431200
C	-4.33595500	-0.96289700	-0.21423900
H	-5.20113000	0.63378800	0.93414400
H	-3.17168000	-2.36897700	-1.34059100
C	2.59941800	2.49775300	-0.92757800
H	3.37237900	3.21157400	-0.61618200
H	2.62418100	2.47116600	-2.02529200
H	1.63667300	2.91788300	-0.61835800
C	1.34340200	-1.48778600	1.97025500
H	1.09774600	-0.88820600	2.85663400
H	0.38877100	-1.71590000	1.47688500
H	1.76991800	-2.43633800	2.31344800
C	5.76384000	-1.33274700	-0.37396600
H	6.32570100	-1.53860900	0.54577400
H	5.63273000	-2.29515400	-0.88474200
H	6.38714800	-0.69803000	-1.01252200
C	-0.81874100	-1.16056800	-1.60305000
H	-0.68892100	-0.70414500	-2.59504700
H	0.11830300	-0.99300000	-1.05495500
H	-0.91491900	-2.23987500	-1.76256300
C	-3.21592100	2.41392300	1.10043600
H	-3.04988300	3.25106400	0.40979000
H	-4.18364200	2.57083200	1.58902900
H	-2.43946900	2.48922700	1.87306100
C	-5.57532100	-1.80485900	-0.18487100
H	-5.41593000	-2.77594500	-0.66575100
H	-5.90989500	-1.99144500	0.84332600
H	-6.40765800	-1.31066500	-0.70234500
B	-0.72271800	1.58815800	-0.20547100
H	0.16863000	1.25710000	-1.53642400
H	-0.36297800	1.73692300	-1.83474400
H	0.16398900	1.07722600	1.95514100
H	-0.88939100	2.78242200	-0.26519800

82) 20-Mes

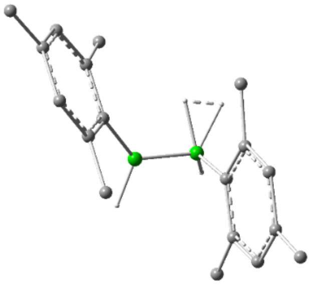

Note: Selected hydrogen atoms are omitted for clarity.

Number of imaginary frequencies = 0

E_{total} = -750.69773 a.u

$G_{correction}$ = 0.340415 a.u

Cartesian coordinates:

C	2.60991600	1.09091300	-0.10856500
C	3.71955300	0.26540500	-0.28871900
C	1.45721300	0.60119400	0.54471500
C	3.72782500	-1.05696300	0.14530900
H	4.60551300	0.66820000	-0.78259400
C	1.47364200	-0.73753000	1.00739700
C	2.59021100	-1.54023800	0.78905700
H	2.57674500	-2.57429000	1.13818100
B	0.20048800	1.49176400	0.80999500
C	-1.71883000	1.02594600	-0.34649100
C	-2.78607000	0.86164100	0.57107700
C	-1.48463300	-0.00143900	-1.28951500
C	-3.56555900	-0.29148300	0.53359500
C	-2.29009100	-1.14072700	-1.29513100
C	-3.33130800	-1.31146200	-0.38718300
H	-4.38847500	-0.39576500	1.24281800
H	-2.09957800	-1.91739100	-2.03731400
C	2.69435800	2.50199300	-0.62673600
H	3.73755100	2.80082000	-0.77648000
H	2.20483900	2.60907500	-1.60738600
H	2.23983900	3.23355800	0.05057900
C	0.27834300	-1.33442900	1.68952100
H	-0.12203900	-0.67108400	2.46646100
H	-0.54136300	-1.50934700	0.97895500
H	0.52579200	-2.29542400	2.15341900
C	4.93054900	-1.93000800	-0.04172700
H	5.49274700	-2.03784300	0.89520300
H	4.64804100	-2.94042300	-0.35991500
H	5.61736400	-1.51853400	-0.78920500

C	-0.40781200	0.11178600	-2.32831900
H	-0.55871500	0.98901900	-2.97434100
H	0.59387100	0.20016200	-1.88545400
H	-0.39659800	-0.76840100	-2.97904300
C	-3.13127800	1.93388600	1.56189200
H	-3.43419700	2.85969000	1.05585300
H	-3.95792000	1.62037100	2.20806700
H	-2.28190500	2.19411400	2.20510000
C	-4.16688400	-2.55470100	-0.38390000
H	-4.08514400	-3.09969800	-1.33057900
H	-3.85666600	-3.24077800	0.41531400
H	-5.22570100	-2.32717300	-0.21344600
B	-0.82587200	2.35317200	-0.23420700
H	0.22103300	2.40353200	-0.97133600
H	-0.48587900	2.61606300	-1.48452100
H	-0.23435500	1.49779900	1.93103200
H	-1.26275000	3.41637100	0.09402500

83) TS-Mes[20-21]

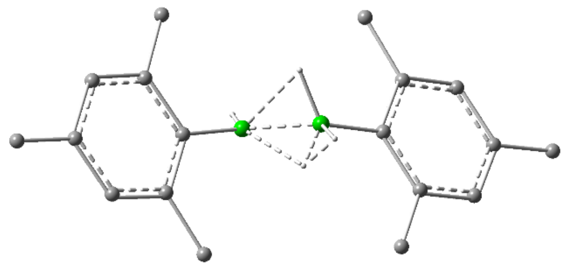

Note: Selected hydrogen atoms are omitted for clarity.

Number of imaginary frequencies = 1 (246.6 cm^{-1})

E_{total} = -750.687838 a.u

$G_{correction}$ = 0.335629 a.u

Cartesian coordinates:

H	-0.11748900	-0.92482600	0.84879400
H	0.25964600	-0.59717400	1.63340200
B	-0.62385800	0.34426900	-0.56181000
C	-2.15513900	0.12305300	-0.33737200
C	-2.75368000	-1.15048000	-0.32803800
C	-4.12667700	-1.27229200	-0.11686400
H	-4.57901600	-2.26543200	-0.12828300
C	-4.93521100	-0.16060200	0.10645900
C	-4.33516700	1.09796200	0.10190700
H	-4.95118500	1.98266500	0.27317900
C	-2.97091600	1.25539700	-0.12745100
B	0.59191100	0.08921200	0.56565500
C	2.14789600	0.01939400	0.33053600
C	2.84057000	-1.20486500	0.28051400
C	2.87932500	1.20773900	0.13418800
C	4.21536100	-1.22748000	0.04771300
C	4.25284800	1.15192200	-0.10093900
H	4.73152300	-2.18859400	0.00897200
C	4.94398800	-0.05664100	-0.14382800
H	4.80146200	2.08278700	-0.25691600
H	0.03900500	1.09962400	0.98311100
H	-0.26325600	0.97588900	-1.52140400
C	2.11137500	-2.50451300	0.46553800
H	1.66269300	-2.59195700	1.46620500
H	1.29573700	-2.61870200	-0.26258400
H	2.78185100	-3.36114200	0.34000300
C	6.42613100	-0.09211800	-0.36519000
H	6.74282100	-1.03401600	-0.82735900
H	6.75720800	0.72895900	-1.01147200
H	6.97627000	0.00332500	0.58065200
C	2.20260200	2.54948900	0.16703100
H	1.42732300	2.64075300	-0.60574900
H	1.70795800	2.73333700	1.13065400
H	2.92231600	3.35941800	0.00642300
C	-1.93702400	-2.39224000	-0.56099800
H	-1.23862900	-2.27815800	-1.39969700
H	-1.34099300	-2.66825600	0.32257200
H	-2.57779500	-3.25230800	-0.78357400
C	-6.41240300	-0.30392100	0.31360300
H	-6.96858300	-0.06294300	-0.60193300
H	-6.68333400	-1.32620900	0.59904600
H	-6.77723500	0.37355700	1.09447700
C	-2.36814200	2.63047500	-0.12361600
H	-1.70694300	2.78817300	0.74066000
H	-1.75689700	2.80465300	-1.02001200
H	-3.13914000	3.40749300	-0.08754000

84) 21-Mes

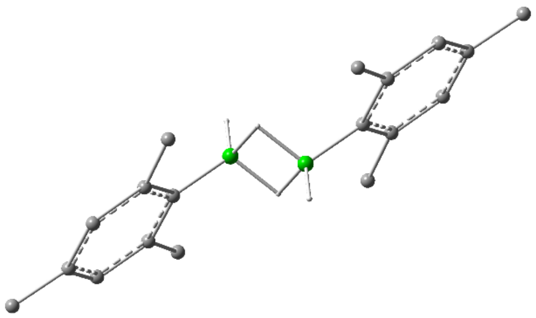

Note: Selected hydrogen atoms are omitted for clarity.

Number of imaginary frequencies = 0

E_{total} = -750.752691 a.u

$G_{correction}$ = 0.339866 a.u

Cartesian coordinates:

C	-2.92298200	-1.24485800	-0.19694900
C	-4.29494600	-1.16108400	0.03059300
C	-2.15324700	-0.07243600	-0.32497000
C	-4.94207400	0.06870300	0.12699600
H	-4.87503500	-2.07971700	0.13418500
C	-2.79913500	1.17416600	-0.22526100
C	-4.17516200	1.22432400	-0.00402100
H	-4.66542200	2.19664800	0.06772200
B	-0.61226200	-0.21930100	-0.59585300
B	0.61226600	0.21931200	0.59586400
C	2.15324900	0.07244100	0.32497900
C	2.92299400	1.24486000	0.19697600
C	2.79912500	-1.17416300	0.22524400

C	4.29495500	1.16107800	-0.03057400
C	4.17515100	-1.22433000	0.00399500
C	4.94207300	-0.06871400	-0.12700400
H	4.87505200	2.07970700	-0.13415300
H	4.66540200	-2.19665600	-0.06777000
H	-0.03769000	-0.89761100	0.39790500
H	0.03769200	0.89762200	-0.39789300
H	-0.19566400	-0.71035900	-1.60793100
H	0.19566800	0.71036800	1.60794300
C	-2.03966400	2.46491300	-0.36056800
H	-1.33190000	2.61555500	0.46614800
H	-1.46081500	2.50662400	-1.29318500
H	-2.71926700	3.32354800	-0.36339900
C	-2.26545700	-2.59232000	-0.28026500
H	-1.67369800	-2.70078700	-1.19948100
H	-1.57771400	-2.76421100	0.56086500
H	-3.00295500	-3.40164100	-0.26302000
C	2.03964200	-2.46490600	0.36052300
H	1.46079300	-2.50663100	1.29313900
H	1.33187500	-2.61552300	-0.46619500
H	2.71923600	-3.32354800	0.36333500
C	2.26548100	2.59232600	0.28031900
H	1.57772600	2.76423400	-0.56079900
H	1.67373600	2.70078500	1.19954500
H	3.00298500	3.40164100	0.26307400
C	-6.42416100	0.14642000	0.33434100
H	-6.96098100	0.14913900	-0.62362000
H	-6.79661400	-0.70953300	0.90830400
H	-6.70993800	1.06128800	0.86544300
C	6.42415800	-0.14643200	-0.33435900
H	6.96099500	-0.14894500	0.62359400
H	6.70995000	-1.06139400	-0.86528800
H	6.79657800	0.70942000	-0.90849400

References

1 S. R. Wang, M. Arrowsmith, J. Böhnke, H. Braunschweig, T. Dellermann, R. D. Dewhurst, H. Kelch, I. Krummenacher, J. D. Mattock, J. H. Müssig, T. Thiess, A. Vargas and J. Zhang, *Angew. Chem. Int. Ed.*, 2017, **56**, 8009.

2 P. Bissinger, H. Braunschweig, M. A. Celik, C. Claes, R. D. Dewhurst, S. Endres, H. Kelch, T. Kramer, I. Krummenacher and C. Schneider, *Chem. Commun.*, 2015, **51**, 15917.

3 H. Nöth and W. Meister, *Z. Naturforsch.*, 1962, **17b**, 714.

4 G. Sheldrick, *Acta Cryst.*, 2015, **A71**, 3.

5 G. Sheldrick, *Acta Cryst.*, 2008, **A64**, 112.

6 M. J. Frisch, G. W. Trucks, H. B. Schlegel, G. E. Scuseria, M. A. Robb, J. R. Cheeseman, G. Scalmani, V. Barone, G. A. Petersson, H. Nakatsuji, X. Li, M. Caricato, A. V. Marenich, J. Bloino, B. G. Janesko, R. Gomperts, B. Mennucci, H. P. Hratchian, J. V. Ortiz, A. F. Izmaylov, J. L. Sonnenberg, D. Williams-Young, F. Ding, F. Lipparini, F. Egidi, J. Goings, B. Peng, A. Petrone, T. Henderson, D. Ranasinghe, V. G. Zakrzewski, J. Gao, N. Rega, G. Zheng, W. Liang, M. Hada, M. Ehara, K. Toyota, R. Fukuda, J. Hasegawa, M. Ishida, T. Nakajima, Y. Honda, O. Kitao, H. Nakai, T. Vreven, K. Throssell, J. A. Montgomery, Jr., J. E. Peralta, F. Ogliaro, M. J. Bearpark, J. J. Heyd, E. N. Brothers, K. N. Kudin, V. N. Staroverov, T. A. Keith, R. Kobayashi, J. Normand, K. Raghavachari, A. P. Rendell, J. C. Burant, S. S. Iyengar, J. Tomasi, M. Cossi, J. M. Millam, M. Klene, C. Adamo, R. Cammi, J. W. Ochterski, R. L. Martin, K. Morokuma, O. Farkas, J. B. Foresman and D. J. Fox, Gaussian 16, Revision C.01, Gaussian, Inc., Wallingford CT, 2016.

7 Y. Zhao and D.G. Truhlar, *Theor Chem Acc.,* 2008, **120**, 215–241.

8 A. V. Marenich, C. J. Cramer and D. G. Truhlar, *J. Phys. Chem. B,* 2009, **113**, 6378-6396.

9 a) A. D. Becke, *Phys. Rev. A*, 1988, **38**, 3098; b) A. D. Becke, *J. Chem. Phys.*, 1993, **98**, 5648; (c) C. Lee, W. Yang and R. G. Parr, *Phys. Rev. B*, 1988, **37**, 785.

10 S. Grimme, J. Antony, S. Ehrlich and H. J. Krieg, *Chem. Phys.,* 2010, **132**, 154104.

11 J. Tomasi, B. Mennucci and R.Cammi, *Chem. Rev.*, 2005, **105**, 2999.

12 N. Tsukahara, H. Asakawa, K.-H. Lee, Z. Lin and M. Yamashita, *J. Am. Chem. Soc.,* 2017, **139**, 2593–2596.

13 a) J. Baker, *J. Comput. Chem.,* 1986, **7**, 385; b) C. Peng, P. Y. Ayala, H. B. Schlegel and M. J. Frisch, *J. Comput. Chem.,* 1996, **17**, 49.

14 a) A. Jayaraman, G. M. Berner, L. M. Mihichuk andA. L. L. East, *J. Mol. Catal. A: Chem.*, 2011, **351**, 143; b) A. Jayaraman and A. L. L. East, *J. Org. Chem.*, 2012, **77**, 351; c) A. Jayaraman and B. T. Sterenberg, *Organometallics* 2016, **35**, 2367.
